# Controlling Glycan Folding with Ionic Functional Groups

**DOI:** 10.1021/jacs.4c17992

**Published:** 2025-04-24

**Authors:** Nishu Yadav, Ana Poveda, Yadiel Vázquez Mena, Martin Rosenthal, Yu Ogawa, Jesús Jiménez-Barbero, Martina Delbianco

**Affiliations:** † Department of Biomolecular Systems, Max Planck Institute of Colloids and Interfaces, Am Mühlenberg 1, 14476 Potsdam, Germany; ‡ Department of Chemistry and Biochemistry, Freie Universität Berlin, Arnimallee 22, 14195 Berlin, Germany; § CICbioGUNE, Basque Research and Technology Alliance, 48160 Derio, Spain; ∥ Ikerbasque, Basque Foundation for Science, 48009 Bilbao, Spain; ⊥ Department of Inorganic & Organic Chemistry, Faculty of Science and Technology, University of the Basque Country, EHU-UPV, 48940 Leioa, Spain; # Centro de Investigación Biomedica En Red de Enfermedades Respiratorias, 28029 Madrid, Spain; ∇ CERMAV, CNRS, Univ. Grenoble Alpes, 38000 Grenoble, France; ○ Faculty of Chemistry, KU Leuven, Celestijnenlaan 200F, Box 2404, B-3001 Leuven, Belgium; ◆ Department of Sustainable and Bioinspired Materials, Max Planck Institute of Colloids and Interfaces, Am Mühlenberg 1, 14476 Potsdam, Germany

## Abstract

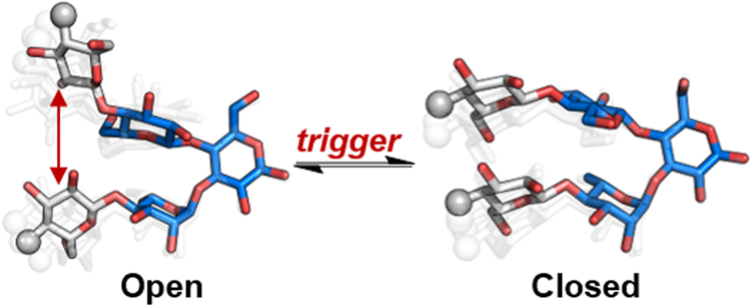

Glycans are intrinsically flexible molecules that can adopt many conformations. These molecules often carry ionic functional groups that influence glycan’s conformational preferences, dynamics, and aggregation tendencies. Inspired by these mechanisms, we have engineered a glycan sequence whose secondary structure can be precisely manipulated by using ionic groups. We strategically incorporated ionic substituents into a glycan sequence adopting a hairpin conformation. Complementary ionic groups stabilized the closed conformers, while ionic repulsions shifted the populations toward the open forms. External stimuli, such as pH variations or enzyme addition, enabled us to dynamically control the hairpin’s opening and closing. Additionally, changes in protonation states led to glycan aggregation, suggesting opportunities for the creation of responsive glycan-based materials.

## Introduction

**Figure 1:**
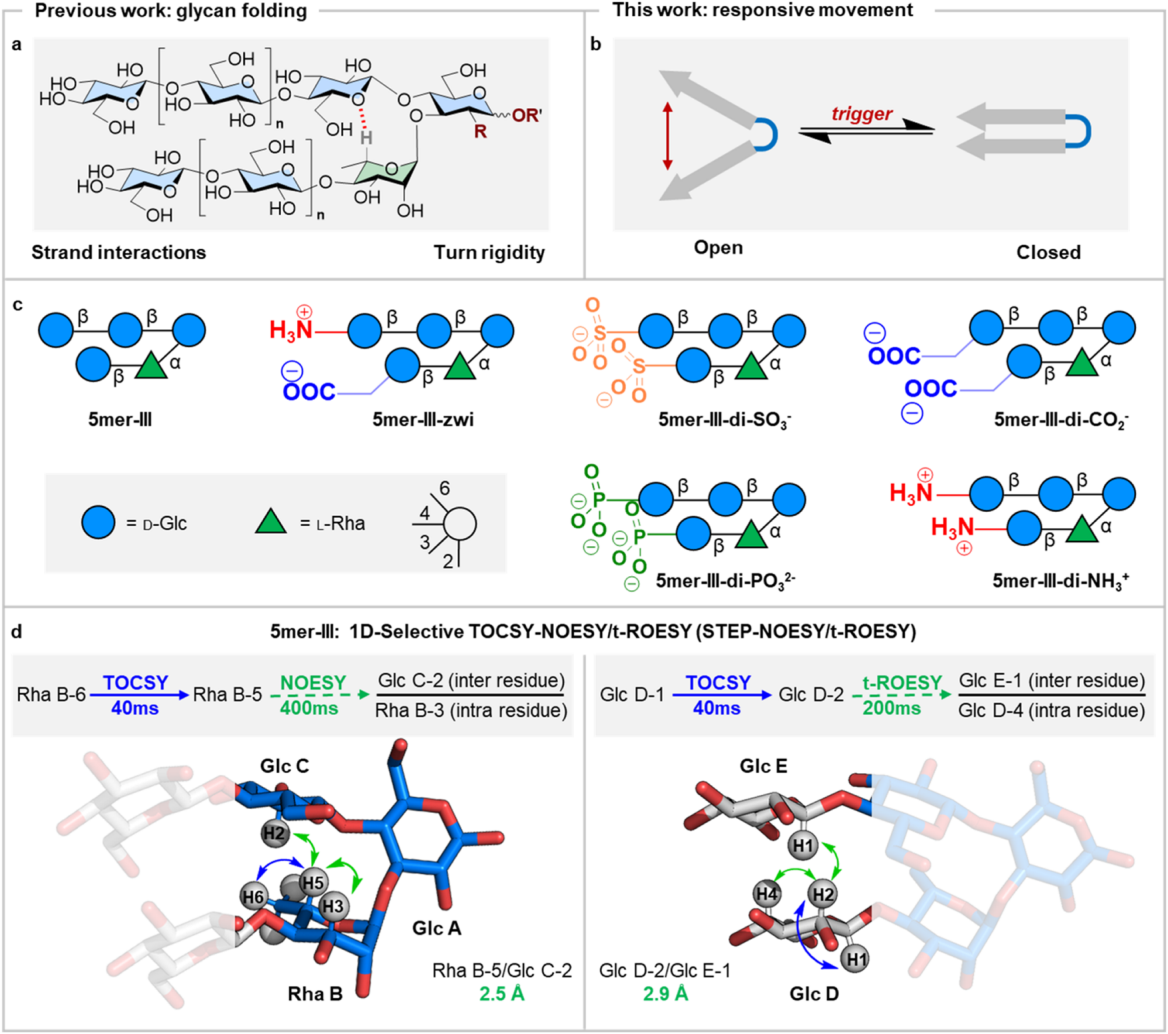
(a) Synthetic glycan hairpin that spontaneously folds into a rigid conformation. (b) Cartoon representation of a dynamic system capable of changing its major conformation between an open and closed state in response to external stimuli. (c) Oligosaccharides synthesized and studied in this work. To standardize the name and representation of the glycan hairpins, a systematic terminology was developed in our previous work and can be found in the Supporting Information (SI).^5^ (d) STEP-NOESY (left) and STEP-t-ROESY (right) NMR experiments performed to measure inter-residue distances in **5mer-III** (spectra are available in Figures S85 and S86). The values obtained for **5mer-III** serve as references to analyze the effect of ionic groups on hairpin conformational preference. The following abbreviations are used for monosaccharides: Glc = glucose (blue circle), and Rha = rhamnose (green triangle). The monosaccharide residues are represented following the Symbol Nomenclature for Glycans (SNFG).^12^

Glycans are highly dynamic molecules that oscillate between various conformations separated by low energy barriers.^1a,1b^ However, some glycans can adopt quite rigid secondary structures^2a−2e^ that can be further stabilized with unnatural substituents.^3^ Building on these natural glycan motifs, we recently designed a glycan sequence capable of spontaneous folding into a hairpin conformation (Figure 1a).^4^ This system features a rigid trisaccharide glycan turn substituted with two cellulose strands that enhance its conformational stability through weak glycan–glycan interactions.^5^ As a next step, we wondered whether this system could be expanded to achieve controllable folding, enabling us to open and close the hairpin on demand. To this end, we considered incorporating functional groups that respond to external stimuli, resulting in programmable conformational changes.

Glycans bearing ionic functional groups such as sulfates, phosphates, carboxylic acids, and amines are ubiquitous in nature.^6^ These substituents influence glycans’ conformational preferences, dynamics, and aggregation tendencies. Ionic groups enhance hydrogen bonding and electrostatic interactions, affecting glycan folding in aqueous environments.^7^ For instance, sulfate groups promote the helical conformations of some polysaccharides,^8^ while carboxylate ions can alter aggregation through ionic interactions.^9a−9c^ Additionally, the presence of ionic groups impacts glycan interactions with other molecules. Zwitterionic oligosaccharides, such as those from *Streptococcus pneumoniae* serotype 1 (Sp1) and polysaccharide A1 (PS A1) from *Bacteroides fragilis*, demonstrated that the spatial arrangement of positive and negative charges stabilize their helical secondary structures.^10a,10b^ This conformation enhances interaction with antibodies, making these oligosaccharides promising candidates for synthetic vaccines.^11^ Beyond secondary structures, ionic groups also influence the polysaccharide assembly. For example, carrageenans, which are highly sulfated glycans, undergo diverse ion-induced self-assembly processes depending on ion type and concentration, resulting in chiral supramolecular architectures.^13a,13b^ Anionic glycans found in algae promote aggregation by forming networks stabilized by ionic interactions, facilitating the assembly of glycans into microgels and acting as carbon sink in marine environments.^14^

We hypothesized that ionic groups could be harnessed to control the folding and assembly of a specifically designed glycan. Herein, we present five synthetic glycan hairpins, each carrying different ionic functionalities at the nonreducing ends of both strands. Long-range electrostatic interactions enable us to stabilize either the “closed” (via attractive interactions) or the “open” (via repulsive interactions) form of the hairpin. External stimuli, such as pH changes or enzyme addition, allow us to control folding and/or trigger aggregation, creating responsive glycans capable of switching between two major states (described as “open” and “closed” forms, Figure 1).

## Results and Discussion

All glycan hairpins discussed in this work feature a rigid trisaccharide turn motif, which contains a reducing d-Glc branching unit substituted with a β-d-Glc unit at C-4 and a α-l-Rha unit at C-3. Previous studies^2d^ have demonstrated that this trisaccharide motif displays a very major “closed” conformation. Nevertheless, there is a certain degree of molecular motion around the glycosidic linkages, which makes possible the existence of a minor population of conformers displaying an “open” geometry.^2e^ This turn motif is further extended with two Glc-based stacking strands, increasing the number of rotatable bonds that define its conformation. The neutral pentasaccharide **5mer-III** (Figure 1c) serves as a reference for conformational analysis. All hairpin analogs, carrying ionic functional groups, were synthesized with automated glycan assembly (AGA).^15a,15b^ Conformational analysis was conducted using nuclear magnetic resonance (NMR) spectroscopy,^16a,16b^ small-angle X-ray scattering (SAXS)^17^, and transmission electron microscopy in cryogenic conditions (cryo-TEM). A systematic terminology for naming and representing the glycan hairpins is detailed in our previous paper.^5^ Residues within a structure are labeled with letters from the reducing to the nonreducing end, prioritizing C-3 linked residues over C-4 linked residues (Figure 1d). Proton labeling in a monosaccharide follows this format: for example, the proton at C-1 of Rha-B is named “Rha B-1.” NMR signals of residues at the reducing-end are additionally labeled with α or β.

All proton nuclei in the molecules were assigned using various NMR experiments (^1^H NMR, correlation spectroscopy (COSY), heteronuclear single quantum coherence (HSQC), selective one-dimensional (1D) total correlation spectroscopy (TOCSY), Section S4). To confirm the spatial proximity between key residues at the termini of the putative hairpin, nuclear Overhauser effect spectroscopy experiments were employed. Estimated distances correspond to average values for the ensemble of all of the existing conformers. Thus, short interstrand distances correspond to a major population of “closed” conformations. 1D selective TOCSY-NOESY experiments^18^ were performed to examine the conformations of all modified hairpins. The results were compared with those obtained for neutral **5mer-III**. Average inter-residue distances were estimated from STEP-NOESY^18^ and STEP-t-ROESY^19^ experiments (Figure 1d, exemplified for **5mer-III**) with a confidence higher than 0.2 Å.

Selective inversion of Rha B-6, followed by TOCSY transfer of coherence to Rha B-5 and then NOESY transfer from Rha B-5, allowed us to measure the intraresidue Rha B-5/Rha B-3 (as reference) and the inter-residue Rha B-5/Glc C-2 distances, applying the isolated spin pair approximation (ISPA).^18^ This analysis confirmed the presence of a major population of “closed” conformation of the turn motif in **5mer-III**, due to the short average intraresidue Rha B-5/Glc C-2 distance of 2.5 Å (Figures 1d and S85). No NOE signal between the interstrand Glc D-2/Glc E-1 of **5mer-III** was detected in the STEP-NOESY experiment (Figure S86). While the absence of NOE could suggest the existence of a rather open hairpin conformation, it could also result from a particular molecular tumbling rate in the region of zero NOE. Zero NOE value occurs when the product of the spectrometer frequency and the effective rotational correlation time for the target proton pair is approximately 1.1.^20^ Therefore, a STEP-t-ROESY experiment was conducted to overcome this problem since NOEs in the rotating frame (from ROESY) are always positive, independent of the molecular motion.

1D selective inversion of Glc D-1 followed by TOCSY transfer of magnetization to Glc D-2 and then t-ROESY from Glc D-2 permitted us to measure the intraresidue Glc D-2/Glc D-4 (as reference) and to detect the inter-residue Glc D-2/Glc E-1 ROEs for **5mer-III** (Figure 1d). The analysis of the ROE data allowed estimation of an inter-residue Glc D-2/Glc E-1 average distance of 2.9 Å (Figures 1d and S86). Taken together, these results indicate that a substantial population of **5mer-III** adopts a geometry that can be defined by the “closed” conformation. Together with the specific signatures of the chemical shifts measured for Rha B-5^2d^ and Glc E-1,^5^ these Rha B-5/Glc C-2 (2.5 Å) and Glc D-2/Glc E-1 (2.9 Å) average distance values were employed as standards to analyze the effect of ionic substituents on the overall distribution of conformations in the hairpin.

### Stabilizing the Closed Conformation with Complementary Ionic Groups

Salt bridges are long-range noncovalent interactions widespread in peptides and proteins.^21^ Charge–charge interactions act over a much longer distance than other types of interactions. In particular, salt bridges between Glu and Lys are common stabilizers of β-hairpin peptides.^22a,22b^ On the other end, solvation of polar and ionic groups could play a detrimental role and sometimes decrease the folded population of certain peptide sequences.^23^

**Figure 2:**
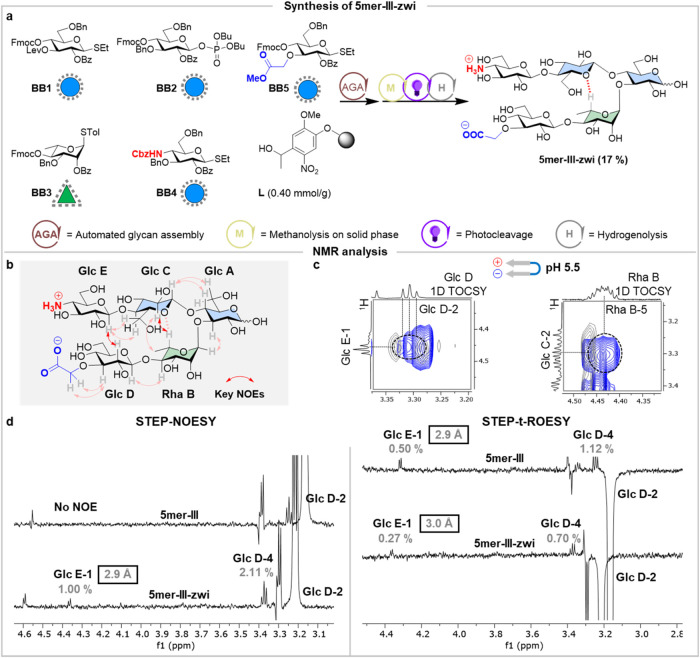
(a) **5mer-III-zwi** glycan hairpin was prepared by AGA using protected monosaccharide BBs. The overall yield is reported in parentheses. Reaction conditions for AGA and post-AGA are reported in Section S3. (b) Experimentally observed interstrand NOEs extracted from NOESY NMR experiments at pH 5.5 for **5mer-III-zwi** (red arrows, the full spectrum is available in Figure S17). (c) Superimposed two-dimensional (2D) NOESY (blue) and 2D TOCSY (gray) NMR of **5mer-III-zwi** showing the key interstrand NOEs (Glc C-2/Rha B-5 and Glc E-1/Glc D-2) with (293 K, mixing times d8 800 ms and d9 150 ms, D_2_O, 700 MHz) (for full spectra see Figure S17). (d) Comparison of inter-residue NOE distances calculated from STEP-NOESY/t-ROESY experiments for **5mer-III-zwi** and **5mer-III** (gray). The estimated confidence of the NOE-based distances is higher than 0.2 Å.

To evaluate the effect of salt bridges on the conformational distribution of our glycan hairpin, we strategically incorporated Glc residues with complementary ionic groups at the nonreducing ends of the two strands (**5mer-III-zwi**, Figure 2a). **5mer-III-zwi** was constructed from monosaccharide building blocks (BBs) using AGA (Sections S2 and S3). **BB 1**–**3** were used to assemble the turn motif, while **BB 4**–**5** bearing a masked amino group (**BB 4**) and a carboxylic acid group (**BB 5**) were placed as strands. AGA followed by on resin methanolysis, photocleavage, and global deprotection resulted in a 17% overall yield (Section S3.5.1).

To investigate the effect of the salt bridge on the hairpin conformation, NMR data for **5mer-III-zwi** and **5mer-III** were compared at pH 5.5 (i.e., the isoelectric point). This was determined by NMR spectroscopy by monitoring the pH-dependent variations of the chemical shifts of the methylene protons, next to the carboxylic acid group, and the H-4 proton, next to the amino group (Figure S12). 2D NOESY and TOCSY NMR experiments allowed assessment of the spatial proximity between key inter-residue protons of the turn and the strands (Figures 2b,c and S17). The presence of the key inter-residue NOE between Rha B-5/Glc C-2 confirmed a major closed conformation for the turn unit. Similar average distances were estimated for both **5mer-III-zwi** (2.5 Å) and **5mer-III** (2.5 Å) (Figure S85 and Table S05), indicating that the rigid turn motif is conserved.

The NOESY spectrum showed clear interstrand NOE signals between Glc E-1/Glc D-2 for **5mer-III-zwi** (Figure 2b,c). In contrast, the Glc E-1/Glc D-2 NOE was at the noise level for **5mer-III**. Given the observed overlap in the 2D spectrum, STEP-t-ROESY experiments were employed, revealing similar interstrand average distances (Glc E-1/Glc D-2 ca. 3.0 Å) for both hairpins (Figures 2d and S86). Overall, these results showed that ionic substituents do not disrupt the predominant geometry of the hairpin (i.e., closed conformation). Moreover, the difference in the NOESY results for the two 5mers suggests that the formation of a salt bridge in **5mer-III-zwi** might provide additional stabilizing interactions between the strands, reducing the local flexibility around the glycosidic linkages at this region.

### Promoting the Open Conformation with Electrostatic Repulsion

Next, we designed two hairpins with identical ionic modifications (i.e., sulfate or phosphate groups) at the terminal Glc residues of both strands. This design was intended to shift the conformational distribution of conformers toward regions defined by the open conformers, given the expected electrostatic repulsion between the ionic groups.

**Figure 3:**
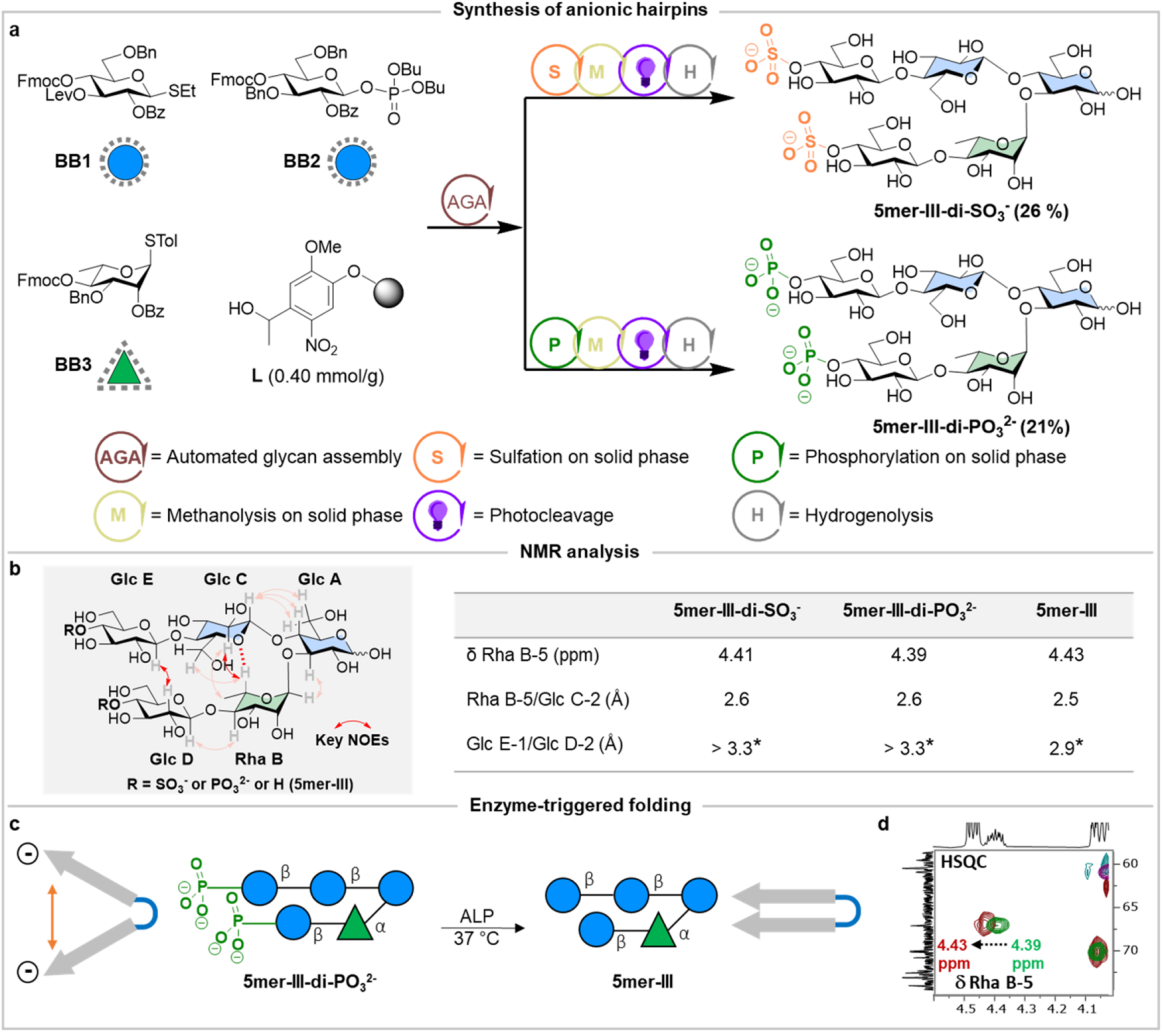
(a) Anionic glycan hairpins were prepared by AGA using protected monosaccharide BBs. Overall yields are reported in parentheses. Reaction conditions for AGA and post-AGA are reported in the Section S3. (b) Comparison of the Rha B-5 chemical shift, reflecting the presence of a nonconventional CH···O hydrogen bond in all hairpins. Still, differences in chemical shift indicate the slight destabilization of the turn unit in both **5mer-III-di-SO**_**3**_^–^ and **5mer-III-di-PO**_**3**_^**2**–^ when compared to **5mer-III**. Experimentally observed key NOEs (red arrows) with estimated average distances between Rha B-5/Glc C-2 and Glc E-1/Glc D-2 extracted from STEP-NOESY/t-ROESY NMR experiments for **5mer-III-di-SO**_**3**_^–^, **5mer-III-di-PO**_**3**_^**2–**^ and compared with **5mer-III** (full spectra are available in Figures S27, S34, S87, and S88). These values indicate the open conformation for both modified hairpins. (c) Dephosphorylation of **5mer-III-di-PO**_**3**_^**2–**^ using alkaline phosphatase (ALP). (d) Superimposed 2D HSQC before (green-purple) and after (red-cyan) dephosphorylation of **5mer-III-di-PO**_**3**_^**2–**^ showing the downfield shift of Rha B-5 from 4.39 to 4.43 ppm (for full spectrum see Figure S38). This shift indicates that phosphate cleavage triggers folding. (*) NOE quantified by STEP-t-ROESY.

The target hairpins were prepared using AGA from **BB 1–3**, following iterative cycles of glycosylation and deprotection. The solid bound 5mer, carrying free hydroxyl groups at Glc D-4 and Glc E-4, was subjected to sulfation using a sulfur trioxide pyridine complex (SO_3_.py) at 40 °C for 15 h in a dimethylformamide (DMF)/Py mixture.^24^ On resin methanolysis followed by photocleavage and global deprotection afforded the target **5mer-III-di-SO**_**3**_^**-**^ in a 26% overall yield (Figure 3a and Section S3.5.2). A similar strategy was employed to construct phosphorylated pentasaccharide **5mer-III-di-PO**_**3**_^**2–**^. AGA was followed by phosphorylation on solid phase using dibenzyl *N*,*N*-diisopropyl phosphoramidite and 5-benzylthio-1*H*-tetrazole in dichloromethane (DCM), with subsequent oxidation using a mixture of pyridine, iodine and H_2_O.^25^ On resin methanolysis, photocleavage and global deprotection yielded **5mer-III-di-PO**_**3**_^**2–**^ in a 21% overall yield (Figure 3a and Section S3.5.3).

The obtained NMR data for **5mer-III-di-SO**_**3**_^**–**^ and **5mer-III-di-PO**_**3**_^**2–**^ in solution were compared to those for the neutral **5mer-III**. First, we focused on the turn. The chemical shift deviation (Δδ) of Rha B-5 served as an experimental marker of the closed conformation.^5^ Both hairpins showed a significant downfield shift (Δδ ∼ 0.33–0.35 ppm) for this proton when compared to the value observed for the control trisaccharide **3mer-V**, which lacks the Glc C unit (δ = 4.06 ppm, taken as zero value).^4^ This is a clear indication of the presence of a significant population of conformers that display the nonconventional H-bond between Rha B and Glc C. However, these Δδ values (Δδ ∼ 0.33–0.35 ppm) suggest that this population is somehow smaller than that in the **5mer-III** hairpin (Δδ ∼ 0.37 ppm) (Figure 3b).^3^ Moreover, the weak intensity of the key inter-residue NOEs Rha B-5/Glc C-2 confirmed the destabilization of the turn motif in both **5mer-III-di-SO**_**3**_^–^ and **5mer-III-di-PO**_**3**_^**2**–^ compared to **5mer-III** (Figures S27 and S34). The NOE data confirmed a slightly larger Rha B-5/Glc C-2 average distance in **5mer-III-di-SO**_**3**_^–^ (2.6 Å) and **5mer-III-di-PO**_**3**_^**2–**^ (2.6 Å) in comparison to **5mer-III** (2.5 Å), supporting a slight shift of the conformational distribution toward open-type geometries of the turn motif (Figures 3b and S85).

We then analyzed the key interstrand NOE between Glc E-1/Glc D-2. As for **5mer-III**, no NOE signal was observed for both anionic 5mers in NOESY and STEP-NOESY experiments (Figure S87), suggesting their open conformation. Therefore, STEP-t-ROESY experiments were carried out that allowed detection of a weak ROE signal (estimated proton–proton average distance >3.3 Å) (Figure S88 and Table S05). This estimated value supports a larger average interstrand distance for both anionic 5mers compared to **5mer-III** (2.9 Å). Overall, these results indicate that anionic modifications promote the opening of the hairpin due to electrostatic repulsion.

Having demonstrated that ionic groups stabilize the open forms of the hairpin, we explored methods to trigger the transition toward the closed conformers. In nature, negatively charged phosphate esters are cleaved by phosphatases, promoting structural rearrangements.^26a−26c^ Thus, we hypothesized that treating **5mer-III-di-PO**_**3**_^**2–**^ with alkaline phosphatase (ALP) would cleave the ionic groups, triggering a transition into the closed hairpin conformation (Figure 3c and Section S4.3.1).

**5mer-III-di-PO**_**3**_^**2–**^ was incubated with ALP and the reaction progress was monitored by matrix-assisted laser desorption ionization time-of-flight (MALDI-TOF), indicating complete cleavage of the phosphate groups after 4 h (Figure S37). NMR studies were performed to compare the conformations before and after dephosphorylation. Stacked HSQC spectra revealed a downfield shift of Rha B-5 from δ 4.39 to 4.43 ppm (Figures 3d and S38), confirming the strengthening of the nonconventional H-bond, a key marker of the closed conformation. The dephosphorylation also reinstalled the interstrand NOE Glc E-1/Glc D-2 cross peak (Figure S41).

Thus, cleavage of the phosphate groups promoted a structural transition from the open to the closed hairpin conformation. This approach enabled us to induce folding on demand and could inspire new avenues for enzyme-triggered engineering of glycans.^27^

### pH Responsive Glycan Foldamers

**Figure 4:**
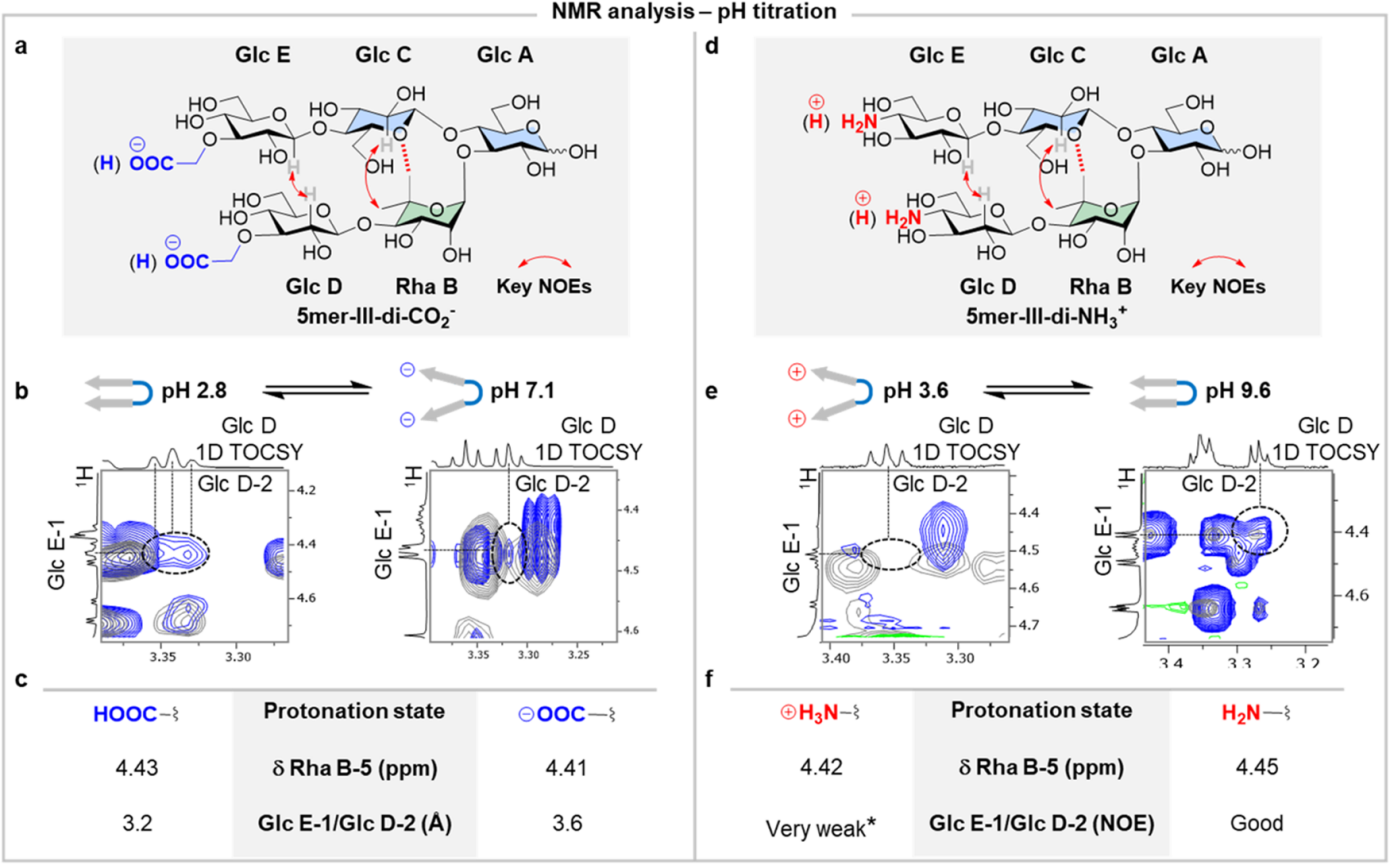
(a) Experimentally observed key NOEs extracted from NOESY NMR experiments for **5mer-III-di-CO**_**2**_^–^ (red arrows). (b) Superimposed 2D NOESY (blue) and 2D TOCSY (gray) NMR of **5mer-III-di-CO**_**2**_^**–**^ showing the key interstrand NOE signal detected at low pH (2.8) but very weak signal observed at high pH (7.1) (293 K, mixing times d8 800 ms and d9 150 ms, D_2_O, 700 MHz) (for full spectra see Figures S46 and S56). (c) Comparison of the estimated interstrand NOE (Glc E-1/Glc D-2) average distances extracted from STEP-NOESY experiments (400 ms mixing time) and Rha B-5 chemical shift values at different pH values for **5mer-III-di-CO**_**2**_**^–^**. (d) Experimentally observed key NOEs extracted from NOESY NMR experiments for **5mer-III-di-NH**_**3**_^**+**^ (red arrows). (e) Superimposed 2D NOESY (blue) and 2D TOCSY (gray) NMR of **5mer-III-di-NH**_**3**_^**+**^ showing no key interstrand NOE signal detected at low pH (3.6) but observed at high pH (9.6) (293 K, mixing times d8 800 ms and d9 150 ms, D_2_O, 700 MHz) (for full spectra see Figures S77 and S80). (f) Comparison of interstrand NOE signal and Rha B-5 chemical shift at different pH for **5mer-III-di-NH**_**3**_^**+**^. (*) Very weak NOE signal was detected in 1D t-ROESY experiments (Figure S81).

While enzyme-triggered folding facilitated the transition from the open to the closed form, it lacked reversibility. To address this, we designed two additional hairpins with conformation that can be modulated by pH changes: **5mer-III-di-CO**_**2**_^–^ and **5mer-III-di-NH**_**3**_^**+**^. These hairpins feature carboxylic acid and amino functionalities at the nonreducing ends of the strands, respectively. These groups were chosen for their ability to undergo (de)protonation in response to basic or acidic conditions, enabling conformational rearrangements. The **5mer-III-di-CO**_**2**_^**–**^ hairpin, incorporating two carboxylic acid groups, was synthesized using carboxylic ester functionalized **BB 5**. AGA, on resin methanolysis, photocleavage, and global deprotection, resulted in a 23% overall yield (Figure 4a and Section S3.5.4). Similarly, the **5mer-III-di-NH**_**3**_^**+**^ hairpin, bearing two amino functionalities, was synthesized using **BB 4**, resulting in a 13% overall yield (Figure 4a and Section S3.5.5).

NMR studies were performed to gain insights into the conformational distribution adopted by both hairpins in solution and to correlate the major folding pattern with the protonation state (Sections S4.4 and S4.5). Titration experiments were performed to assess the major foldamer conformation as a function of pH (Figure S49A). First, we examined **5mer-III-di-CO**_**2**_^**–**^. At neutral pH, both carboxylic acid groups existed in their ionic form. The Rha B-5 chemical shift at δ = 4.41 ppm (Δδ 0.35 ppm) evidenced the presence of a significant population of conformers displaying the nonconventional hydrogen bond within the turn unit. Additionally, the presence of the key inter-residue NOE between Glc C-2 and Rha B-5 confirmed the stability of the turn motif. In contrast, no NOE signal was observed for the interstrand Glc E-1/Glc D-2 strongly suggesting a substantial population of conformers displaying open-like geometries, driven by ionic repulsion between the carboxylate groups (Figures S46–S48).

Protonation of the carboxylate groups was achieved by sequential addition of 1 M HCl, resulting in a significant downfield shift of the methylene protons adjacent to the COOH (Figure S49A–C). Complete protonation occurred at pH < 3. Consequently, the chemical shift of Rha B-5 changed from δ = 4.41 ppm (neutral pH) to δ = 4.43 ppm (low pH), suggesting a more compact conformation of the turn motif likely due to reduced Coulombic repulsions between the strands (Figure 4c). The strengthening of the inter-residue key NOE at the turn (Glc C-2/Rha B-5) and the observation of the Glc E-1/Glc D-2 NOE further confirmed the transition to a distribution of conformers in which the closed hairpin geometry is highly populated (Figures 4b,c, S53, and S56).

Subsequent stepwise addition of 1 M NaOH increased the pH, leading to deprotonation of the carboxylic acid groups. At pH > 5, electrostatic repulsion between the carboxylate groups disrupted the closed conformation, reverting to the open conformers (Figures S59–S63). A STEP-NOESY experiment was performed to calculate the interstrand distance (Glc E-1/Glc D-2), revealing a shorter distance at low pH (3.2 Å) compared to high pH (3.6 Å) (Figures 3c and S89). Careful analysis of the NMR titration from high to low pH showed a linear correlation between the downfield shift of Rha B-5 and the high-field chemical shift of Glc E-1, highlighting the changes in the conformational distribution induced by pH variations (Figure S50).^5^

A similar analysis was conducted with **5mer-III-di-NH**_**3**_^**+**^. During the titration process, the protonation state of the amino group was easily monitored by observing the chemical shift of the Glc D/E-4 protons (highlighted with red dotted circles in Figure S74). At neutral pH, the amino groups were in equilibrium between protonated and deprotonated states. 2D NOESY and selective 1D t-ROESY (Figures S71–S73) revealed a clear Glc C-2/Rha B-6 NOE (strong). The Glc E-1/Glc D-2 was weak yet observable at the noise level, suggesting a conformational equilibrium where a substantial population of the closed geometry exists at pH 7.0 (Figure S72).

1 M NaOH (1 M) was gradually added until NMR confirmed that both amino groups were in their neutral form. At pH > 9, we observed a downfield shift of Rha B-5 from δ = 4.43 ppm (neutral pH 6.96) to δ = 4.45 ppm (high pH 9.60) (Table S03), along with the strengthening of key inter-residue NOEs (Glc C-2/Rha B-6 and Glc E-1/Glc D-2). Selective 1D t-ROESY (Figure S78) confirmed that the conformational equilibrium is dominated by geometries that can be defined by the closed conformation (Figures 4d,e and S77).

In contrast, at pH 3.6, when both amino groups were in their ionic form, an upfield shift of Rha B-5 (δ = 4.42 ppm) was observed, suggesting the destabilization of closed-type conformers of **5mer-III-di-NH**_**3**_^**+**^ (Figures 4d–f and S80). No interstrand NOE signal between Glc E-1/Glc D-2 was detected in the 2D NOESY NMR and only an extremely weak signal was detected in the 1D t-ROESY (Figure S81), indicating the transition toward the open conformer.

To confirm that the observed changes in the chemical shifts resulted from the different protonation states of the terminal groups, which affected the hairpin conformational distribution, control experiments were conducted on neutral **5mer-III**. The observed values for **5mer-III** remained unaffected by the pH changes (Figure S84). In summary, the introduction of ionic functionalities enabled reversible control of glycan folding through external stimuli.

### SAXS and TEM Analysis

The ability to control the distribution of glycan conformers through Coulombic interactions can induce supramolecular aggregation^13b^ and may serve as a valuable tool for creating responsive glycan materials. To assess whether pH changes affect the aggregation of our hairpin, we studied **5mer-III-di-CO**_**2**_^**–**^ at low and high pH using small-angle X-ray scattering (SAXS), a technique that can determine the size and shape of molecules in solution state.^17^ Experiments were conducted without salt, with specific amounts of HCl or NaOH added to explore the influence of intra- and intermolecular Coulombic interactions on hairpin conformation and aggregation tendency.

**Figure 5:**
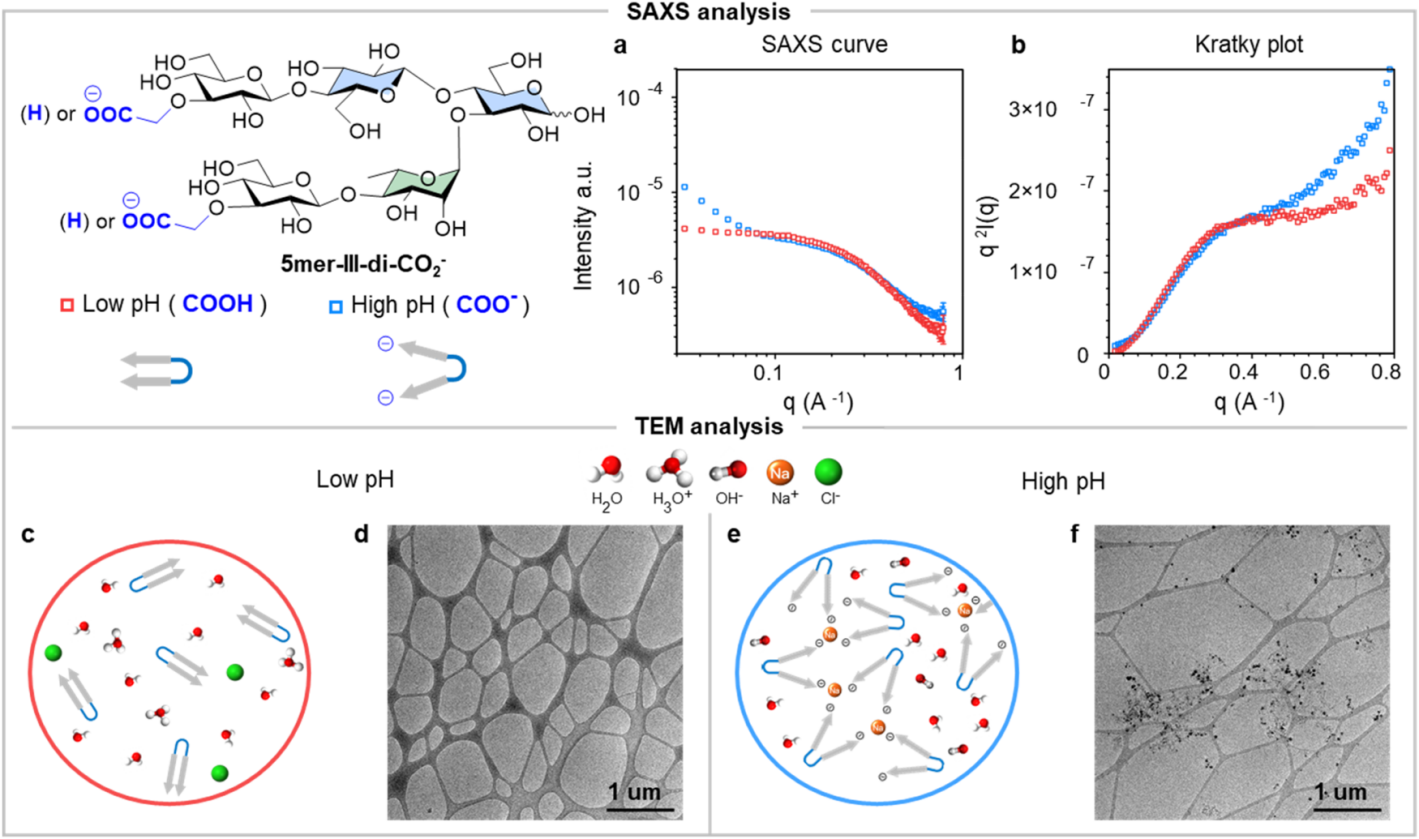
(a) Small-angle X-ray scattering intensity plots of hairpin **5mer-III-di-CO**_**2**_**^–^** (10 mg/mL) at pH 2.9 (low pH) and 11 (high pH). (b) Kratky plots of hairpin **5mer-III-di-CO**_**2**_**^–^** (10 mg/mL) in both conditions. (c) Model showing dispersed hairpins at low pH; (d) cryo TEM analysis at low pH; (e) model showing interactions between negatively charged hairpin-mediated cations at high pH; (f) cryo TEM analysis at high pH.

SAXS intensity curves at pH < 3 were characterized by a pristine tendency along *y*-axis in the low-*q* region (Figure 5a), consistent with a monodispersed oligosaccharide solution. A radius of gyration of 5.46 Å was estimated from the low-*q* region, comparable to the predicted radius of gyration for the closed conformation of the neutral glycan hairpin **5mer-III** (5.20 Å) using molecular dynamics (MD) simulations (Figure S65).^5^ In contrast, at high pH, an increase in SAXS intensity in the low-*q* region indicated aggregation (Figure 5a), a phenomenon commonly observed in polysaccharide solutions including alginates,^28^ carrageenan,^29^ and bacterial polysaccharides.^30^ The differences were more pronounced in the Kratky plot, which showed a larger increasing tendency of *q*^2^*I*(*q*) at the higher *q* range at basic pH. This result suggests a more flexible and extended conformation due to electrostatic repulsion in the deprotonated molecule (Figure 5b).

Further analysis using cryo-TEM at both low and high pH values provided additional insights. At low pH, no aggregates were detected (Figure 5c), whereas at high pH, a substantial amount of aggregates was imaged (Figure 5d). This is consistent with the SAXS results that showed aggregation at the low *q* range under low pH condition. By combining these techniques, we gained a comprehensive understanding of the conformational changes and aggregation tendency of the **5mer-III-di-CO**_**2**_^**–**^ hairpin at different pHs. This behavior may be exploited in the future to trigger the formation of supramolecular glycan materials.

## Conclusions

Responsive foldamers, based on peptide or aromatic motifs, exhibit a unique ability to dynamically respond to external stimuli.^31a−31c^ Their tunable structural properties make them valuable tools in various fields as molecular machines, sensor, probes, drug delivery systems, and responsive materials.^32a,32b^ Here, we showed that glycan sequences can also be engineered to allow control over their conformation, opening new opportunities in the glycosciences.

We have shown that the conformational distribution of a glycan hairpin can be precisely manipulated by using strategically placed ionic substituents. Complementary ionic groups stabilized the closed conformers, while ionic repulsions favored alternative geometries, in which the strands are separated. The interconversion between these states could be controlled with external stimuli such as pH changes or enzyme addition. This approach may find use in the development of glyco-drugs, allowing to change their state between active and inactive states.^33a,33b^ Additionally, the ability to control the conformation of glycans could in turn induce supramolecular aggregation, offering opportunities for the on demand formation of supramolecular glycomaterials.^34^

Lastly, ionic groups are ubiquitous in natural glycans. This work introduces advanced analytical protocols to study the effect of ionic groups on glycan conformation and aggregation. These strategies can be applied to study other glycans and biomolecules, facilitating the establishment of structure–property correlations.^35^

## Experimental Section

### Synthesis

The oligosaccharides were prepared using a home-built synthesizer designed at the Max Planck Institute of Colloids and Interface.^36^ All details concerning **BB** synthesis, AGA modules, and post-AGA manipulations can be found in Sections S2 and S3.

### NMR Analysis

All NMR experiments were carried out using mostly a Bruker Biospin AVANCE 700 (700 MHz). The STEP-NOESY/t-ROESY experiments were recorded on an AVANCE III 800 (800 MHz) spectrometer. Samples were prepared by dissolving lyophilized samples in D_2_O, using concentrations ≈1–4 mM. 2D Heteronuclear ^1^H–^13^C HSQC experiments were carried out at 700 MHz, using the hsqcedetgpsisp2.3 pulse program, 128 or 256 increments in the indirect dimension and 2k data points in the detection dimension, with either 8 or 16 scans. Homonuclear experiments (COSY, TOCSY, NOESY) were carried out using the 700 MHz instruments using 256 or 384 increments in the indirect dimension with 2k or 4k data points in the detection dimension. The number of scans was between 8 and 16, depending on the concentration employed and the intrinsic sensitivity of the experiment, which is lower for NOESY. A variety of mixing times were employed for the 2D TOCSY (mlevphpp pulse program with mixing times d9 80 ms, or 150 ms, d9 determines the duration of the spin lock and hence over how many protons the magnetization will be distributed) and 2D NOESY (noesygpphpp pulse program with mixing times d8 600 ms, or 800 ms, d8 determines the duration of mixing time. It largely depends on the relaxation behavior of the investigated molecule).

Selective 1D-TOCSY and 1D t-ROESY experiments were also carried out in the 700 and 800 MHz instruments to detect all of the protons within a particular spin system (1D-TOCSY, seldigpz pulse program with mixing times d9 = 40, 80, 120, 160, and 200 ms) and those in close distance of the selectively inverted one with 1D t-ROESY (selrogp.2 pulse program with mixing times of 200 and 300 ms). The estimation of the interstrand distances was achieved using the Bruker AVANCE III 800 instrument through the use of STEP-NOESY and STEP-t-ROESY experiments, built by concatenation of a selective 1D-TOCSY module (mixing time optimized between 40 and 60 ms, depending on the particular case) with a selective 1D-NOESY (mixing time 400 ms) or selective 1D-t-ROESY modules (mixing time 200 ms), using pulse sequences written at CIC bioGUNE, which are available from the authors upon request. The equation used to estimate the inter-residue distance is given below; where *r_ij_* is estimated distance, *r*_ref_ is reference distance, *V*_ref_ is integral of NOE peak between reference proton pair, and *V_ij_* is integral of NOE peak between target proton pair.
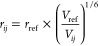


The proton integrals measured in the step-NOESY (400 ms mixing time) or STEP-t-ROESY experiments (200 ms mixing time) were employed to estimate the proton–proton distances using the isolated spin pair approximation (ISPA), using as references the Rha B3–B5 or the Glc C1–C3 distances (2.5 Å). For the application of ISPA, it was assumed that the global rotational motion of these molecules can be described as isotropic,^37^ since the shape of these pentasaccharides (at least in the closed form that produce the interstrand NOEs) is quasi-spherical. The possibility of the existence of spin diffusion was also tested. Therefore, the expected NOEs and ROEs for the closed form and for an 80:20 conformational equilibrium of the open form and a representative open geometry were guessed by using a full matrix relaxation approach (MSpin software). The estimated rotational motion correlation time for the closed form is 800 ps. Given the possibilities of the existence of open forms, additional correlation times 25% faster (600 ps) and slower (1 ns) were also employed. Thus, the expected NOEs at 100, 200, 300, 400, 600, and 800 ms were predicted, as well as the ROEs at 50, 100, 150, 200, and 300 ms. Fittingly, for all of the correlation times and molecular shapes, the ratios of the expected (B5–C2/B5–B3) and (E1–D2/D2–D4) NOESY and ROESY intensities were exactly the same (1.40–1.41 and 0.59–0.60, respectively), supporting the lack of spin diffusion for these molecular shapes. It is also noteworthy to point out that the NOE-based estimated distances correspond to average values. These structures show certain motion around the glycosidic linkages (restricted within certain regions of the corresponding Φ/Ψ maps), which may give rise to the presence of populations of “open” conformers (or several), which may even be bound by a lectin^2e^ through induced fit/conformational selection events. Given the intrinsic features of NOE/ROE, the intrinsic error in the determination of the values of the integrals, and the signal-to-noise ratio for certain cross peaks, the confidence of the estimated average distanced values is higher than 0.2 Å.

### SAXS Analysis

X-ray scattering experiments were performed at the BM26 beamline of the European Synchrotron Radiation Facility (ESRF). Sample with concentration 1.0 wt % at 25 °C were sealed in glass capillaries and mounted on a motorized sample changer. They were exposed to monochromatic X-rays of 12 keV (λ = 1.033 Å). The scattering intensity was measured by using two-dimensional pixel detectors (Pilatus1M, Dectris). The data processing was performed using pyFAI software.^38^ Intensity and Rg analyses were done using Gnuplot software.

## Data Availability

The authors declare that all data supporting the findings of this study are available within the article and in the supporting information files. Raw data for NMR analysis and SAXS can be downloaded from 10.17617/3.EAO4VX, Edmond. Data are also available from the corresponding author upon request.

## Supporting Information Available

All experimental procedures of synthesis and conformational analysis; characterization data, including HPLC, HR-MS, MALDI-TOF, NMR, SAXS, and TEM (PDF)
