# Supplementary material for: Controlling Glycan Folding with Ionic Functional Groups
Source: J Am Chem Soc. 2025 Apr 24;147(18):15126–35. doi: 10.1021/jacs.4c17992 (PMC12063165; doi:10.1021/jacs.4c17992)
Supplement: Supplementary file 1 — ja4c17992_si_001.pdf [file ja4c17992_si_001.pdf]

Supporting Information

Controlling glycan folding with ionic functional groups

Nishu Yadav,[1,2] Ana Poveda,[3] Yadiel Vázquez Mena,[1,7] Martin Rosenthal,[8] Yu Ogawa,[7,9] Jesús Jiménez-Barbero,[3,4,5,6] and Martina Delbianco[1]*

[1]Department of Biomolecular Systems, Max Planck Institute of Colloids and Interfaces, Am Mühlenberg 1, 14476, Potsdam, Germany
[2]Department of Chemistry and Biochemistry, Freie Universität Berlin, Arnimallee 22, 14195, Berlin, Germany
[3]CICbioGUNE, Basque Research and Technology Alliance, 48160, Derio, Spain
[4]Ikerbasque, Basque Foundation for Science, 48009, Bilbao, Spain
[5]Department of Inorganic & Organic Chemistry, Faculty of Science and Technology, University of the Basque Country, EHU-UPV, 48940, Leioa, Spain
[6]Centro de Investigación Biomedica En Red de Enfermedades Respiratorias, 28029, Madrid, Spain
[7]Univ. Grenoble Alpes, CNRS, CERMAV, 38000, Grenoble, France
[8]Faculty of Chemistry, KU Leuven, Celestijnenlaan 200F, Box 2404, B-3001 Leuven, Belgium
[9]Department of Sustainable and Bioinspired Materials, Max Planck Institute of Colloids and Interfaces, Am Mühlenberg 1, 14476, Potsdam, Germany

*Email: martina.delbianco@mpikg.mpg.de

Table of contents

1 General materials and methods ... 7

2 Building blocks ... 8

Figure S01 ... 8

2.1 Synthesis of BB5 ... 9

2.2 Synthesis of BB6 ... 12

3 Automated glycan assembly ... 15

3.1 General materials and methods ... 15

3.2 Preparation of stock solutions ... 15

3.3 Modules for automated synthesis ... 15

3.3.1 Module A: Resin preparation ... 15

3.3.2 Module B: Acidic wash with TMSOTf solution (20 min) ... 16

3.3.3 Module C1: Thioglycoside glycosylation (35 min-55 min) ... 16

3.3.4 Module C2: Glycosyl phosphate glycosylation (45 min) ... 17

3.3.5 Module D: Capping (30 min) ... 17

3.3.6 Module E1: Fmoc deprotection (9 min) ... 18

3.3.7 Module E2: Lev deprotection (90 min) ... 18

3.4 Post-AGA manipulations ... 18

3.4.1 Module F: On resin sulfation ... 18

3.4.2 Module G: On-resin phosphorylation ... 19

3.4.3 Module H1: On-resin methanolysis ... 19

3.4.4 Module H2: On-resin methanolysis ... 19

3.4.5 Module I1: Cleavage from solid support ... 19

3.4.6 Module I2: Micro-cleavage from solid support ... 19

3.4.7 Module J1: Hydrogenolysis ... 19

3.4.8 Module J2: Hydrogenolysis ... 19

3.4.9 Module K: Purification ... 20

3.5 Oligosaccharides synthesis ... 21

Figure S02 ... 21

Figure S03 ... 21

3.5.1 5mer-III-zwi ... 22

3.5.2 5mer-III-di-SO_3^- ... 26

3.5.3 5mer-III-di-PO_3^{2-} ... 30

3.5.4 5mer-III-di-CO_2^- ... 35

3.5.5 5mer-III-di- NH_3^+ ... 39

4 NMR studies ... 43

4.1 NMR characterization of 5mer-III-zwi ... 43

Figure S04 43
Figure S05 44
Figure S06 45
Figure S07 45
Figure S08 46
Figure S09 47
Figure S10 47
Figure S11 48
4.1.1 pH titration of 5mer-III-zwi 49
Figure S12 49
Figure S13 50
Figure S14 51
Figure S15 52
Figure S16 53
Figure S17 54
Figure S18 55
Figure S19 56
Figure S20 57
Figure S21 58
Figure S22 59
Table S01 59
4.2 NMR characterization of 5mer-III-di-SO_3^- 60
Figure S23 60
Figure S24 61
Figure S25 62
Figure S26 63
Figure S27 64
Figure S28 65
Figure S29 66
4.3 NMR characterization of 5mer-III-di-PO_3^{2-} 67
Figure S30 67
Figure S31 68
Figure S32 69
Figure S33 69
Figure S34 70
Figure S35 71
Figure S36 72

4.3.1 Analysis of enzyme triggered dephosphorylation 73
4.3.2 MALDI-TOF of 5mer-III-di-PO_3^{2-} before and after dephosphorylation 74
Figure S37 74
4.3.3 NMR Studies after enzyme triggered dephosphorylation 75
Figure S38 75
4.3.4 Inter-residue NOEs comparison before and after dephosphorylation 76
Figure S41 76
4.4 NMR characterization of 5mer-III-di-CO_2^- 77
Figure S42 77
Figure S43 78
Figure S44 79
Figure S45 79
Figure S46 80
Figure S47 81
Figure S48 81
4.4.1 pH titration of 5mer-III-di-CO_2^- 82
Figure S49 A. 82
Figure S49 B 83
Figure S49 C 84
Figure S50 85
Figure S51 86
Figure S52 87
Figure S53 88
Figure S54 89
Figure S55 89
Figure S56 90
Figure S57 91
Figure S58 92
Figure S59 93
Figure S60 94
Figure S61 95
Figure S62 96
Figure S63 97
Figure S64 98
Table S02 98
4.4.2 Examination of scattering curves at different pH 99
Figure S65 99

Figure S66 100
4.5 NMR characterization of 5mer-III-di-NH_3^+ 101
Figure S67 101
Figure S68 102
Figure S69 103
Figure S70 103
Figure S71 104
Figure S72 105
Figure S73 106
4.5.1 pH titration of 5mer-III-di-NH_3^+ 107
Figure S74 107
Figure S75 108
Figure S76 109
Figure S77 110
Figure S78 111
Figure S79 112
Figure S80 113
Figure S81 114
Figure S82 115
Figure S83 115
Table S03 115
4.6 pH titration of 5mer-III 116
Figure S84 116
4.7 Evaluation of the non-conventional H-bond at neutral pH for all 5mer 117
Table S04 117
4.8 STEP-NOESY/t-ROESY inter-residue distance estimation 118
Figure S85 119
Figure S86 120
Figure S87 121
Figure S88 121
Figure S89 122
Table S05 123
4.9 Estimation of the inter-residue NOEs and ROEs using full matrix relaxation approach (MSpin software) 124
4.9.1 Estimation for the closed form of 5mer-III 124
4.9.2 Estimation for the 80:20 conformational equilibrium of the open form of 5mer-III 127
4.10 ^{1}H and ^{13}C chemical shifts assignment for all the compounds 130

5 References 132

1 General materials and methods

All chemicals used were reagent grade and used as supplied unless otherwise noted. The automated syntheses were performed on a home-built synthesizer developed at the Max Planck Institute of Colloids and Interfaces.[1] Analysis and purification by normal and reverse phase HPLC were performed by using an Agilent 1200 series. Products were lyophilized using a Christ Alpha 2-4 LD plus freeze dryer. ^{1}H, ^{13}C, COSY, HSQC, 1D and 2D TOCSY, 1D t-ROESY, 2D NOESY NMR spectra were recorded on Bruker Biospin AVANCE700 (700 MHz) using probe (Z135421_0008 (CPP TCI 700S3 H&F-C/N-D-05 Z). The STEP-NOESY/t-ROESY experiments were recorded at Bruker AVANCE III 800 (800 MHz) spectrometer using probe (5mm CPTCI 1H-13/15N/D Z-GRD Z44909/0040). Building block spectra were recorded in $CDCl_3$ by using the solvent residual peak chemical shift as the internal standard ($CDCl_3$: 7.26 ppm ^{1}H, 77.0 ppm ^{13}C). Oligosaccharide's samples were prepared by dissolving lyophilized compounds in D_2O (concentration ≈ 1 - 4 mM) using the solvent as the internal standard in ^{1}H NMR (D_2O: 4.79 ppm ^{1}H). All the spectra were recorded at same temperature i.e. 293 K. ^{1}H NMR spectra for all the compounds were recorded without ^{13}C decoupling. Weak intensity ^{13}C resonances were derived from the respective HSQC cross peaks. Proton resonances of the oligosaccharides were assigned using a combination of ^{1}H, 2D COSY, HSQC, 1D and 2D TOCSY. Selective 1D TOCSY (HOHAHA, pulse program: seldigpzs) spectra were recorded using different mixing times to assign all the resonances (d9 = 40, 80, 120, 160, and 200 ms). 2D TOCSY (pulse program: mlevphpp) spectra were recorded using different mixing times (d9 = 80, or 150 ms). Selective 1D t-ROESY (pulse program: selrogp.2) spectra were recorded using different mixing times (p15 =200, or 300 ms). 2D NOESY (pulse program: noesygpphpp) spectra were recorded using different mixing times (d8 = 600, or 800 for NOESY). ^{1}H NMR integrals of the resonances corresponding to residues at the reducing end are reported as non-integer numbers and the sum of the integrals of α and β anomers is set to 1. High resolution mass spectra were obtained using a 6210 ESI-TOF mass spectrometer (Agilent) and a MALDI-TOF autoflexTM (Bruker). X-ray scattering experiments were performed at the D2AM beamline of the European Synchrotron Radiation Facility (ESRF). Monosaccharide were named as follows: D-glucose at position A (Glc A), L-rhamnose at position B (Rha B). Labelling of protons in a monosaccharide is done as follows: e.g. proton attached to C-1 of Rha B is named "Rha B-1". Resonances of residues at the reducing end are additionally labelled with α or β.

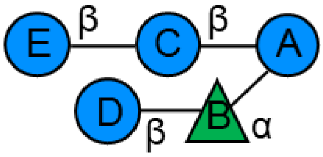

2 Building blocks

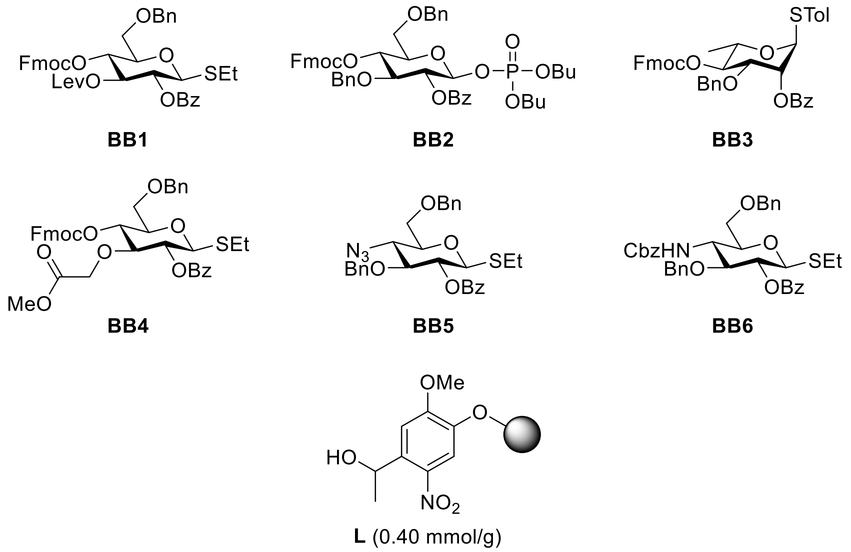

Figure S01
BBs and solid supports used in this work. Loading of **L** is reported in parenthesis.

BB1, BB2 and **BB3** were synthesized according to previously reported procedures.[3] **BB4** was synthesized according to previously reported procedure.[4] The synthesis of **BB5** and **BB6** are described herein (**section 2.1** and **2.2**). Merrifield resin equipped with photocleavable linkers **L** (loading 0.40 mmol/g) was prepared according to previously reported procedures.[5]

2.1 Synthesis of BB5

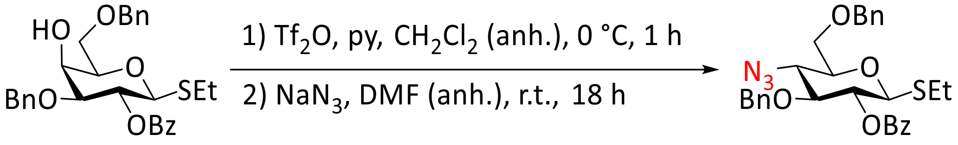

S-1 **BB5**

S-1 was synthesized according to previously reported procedures.[6]

Compound **S-1** (294 mg, 0.58 mmol) was dissolved in anhydrous CH_2Cl_2 (10.5mL) under argon atmosphere and anhydrous pyridine (236 μL, 2.93 mmol, 5 equiv.) was added. The solution was cooled to 0°C and triflic anhydride (195 μL, 1.16 mmol, 2 equiv.) was added. The reaction mixture was further stirred at 0°C for 1 h. Upon complete conversion (indicated by TLC), the reaction was quenched with ice-cold water, washed three times with ice-cold water and brine. The organic layer was separated and dried over Na_2SO_4, filtered and concentrated under reduced pressure to give the triflate intermediate as a yellow solid. The product was readily used for the next step without further purification. The solid was dissolved in anhydrous DMF (10.5 mL) under argon atmosphere and sodium azide (75 mg, 1.16 mmol, 2 equiv.) was added. The reaction mixture was stirred at r.t. for 18 h. Upon complete conversion indicated by TLC, the solvent was evaporated under reduced pressure, the residue dissolved in EtOAc and washed with saturated $NaHCO_3$ and brine. The organic layer was separated and dried over Na_2SO_4, filtered and concentrated under reduced pressure. The greenish crude was further purified via flash column chromatography (hexanes:EtOAc (9:1)) affording the desired **BB5** as a colorless oil (261 mg, 85 %).

^{1}H NMR (700 MHz, $CDCl_3$) δ 8.06 – 8.03 (m, 2H), 7.59 (ddt, *J* = 8.7, 7.3, 1.3 Hz, 1H), 7.48 – 7.45 (m, 2H), 7.36 (d, *J* = 5.0 Hz, 4H), 7.33 – 7.27 (m, 1H), 7.21 – 7.15 (m, 5H), 5.32 (dd, *J* = 10.0, 9.0 Hz, 1H), 4.75 (d, *J* = 10.8 Hz, 1H), 4.68 – 4.64 (m, 2H), 4.58 (d, *J* = 12.1 Hz, 1H), 4.51 (d, *J* = 10.0 Hz, 1H), 3.83 – 3.70 (m, 4H), 3.40 (ddd, *J* = 10.2, 4.4, 1.9 Hz, 1H), 2.78 – 2.64 (m, 2H, SCH_2CH_3), 1.23 (t, *J* = 7.5 Hz, 3H, SCH_2CH_3).

^{13}C NMR (176 MHz, $CDCl_3$) δ 165.16, 137.91, 137.05, 133.37, 129.88, 129.65, 128.52, 128.44, 128.36, 128.31, 127.96, 127.75, 83.63, 82.65, 78.56, 77.24, 77.06, 76.88, 75.18, 73.58, 72.20, 69.15, 61.88, 23.91 (S*C*H$_2$CH$_3$), 14.88 (S*C*H$_2$CH$_3$).

ESI-HRMS m/z 556.1880 $[M+Na]^+$ ($C_{29}H_{31}NaN_3O_5S$ requires 556.1876).

^{1}H NMR of BB5 (700 MHz, $CDCl_3$)

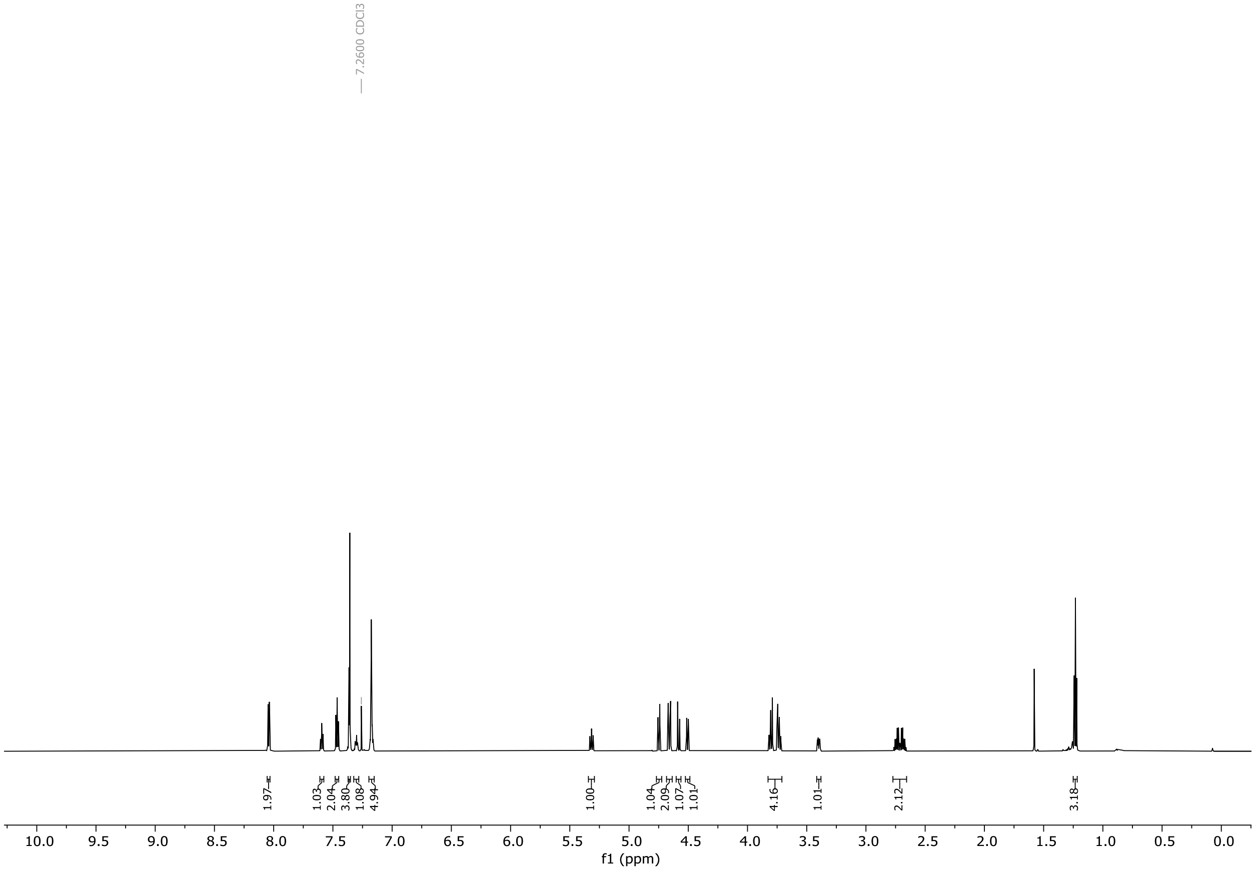

^{13}C NMR of BB5 (700 MHz, $CDCl_3$)

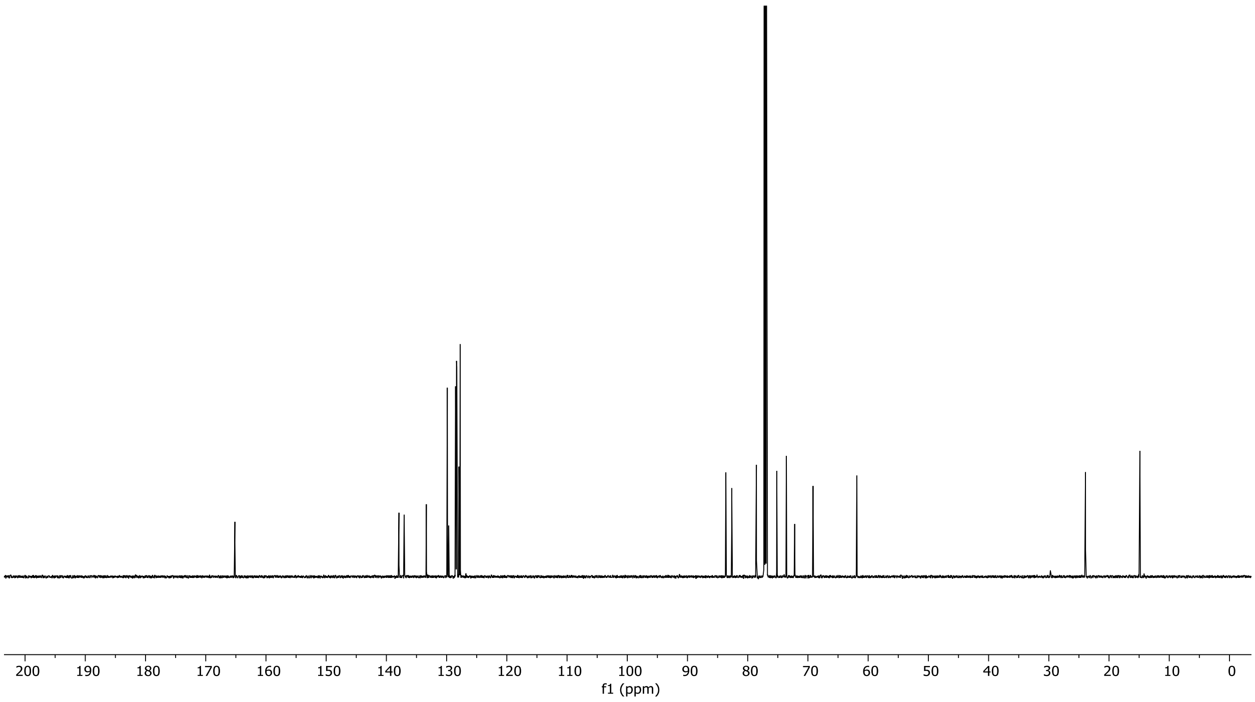

COSY NMR of BB5 ($CDCl_3$)

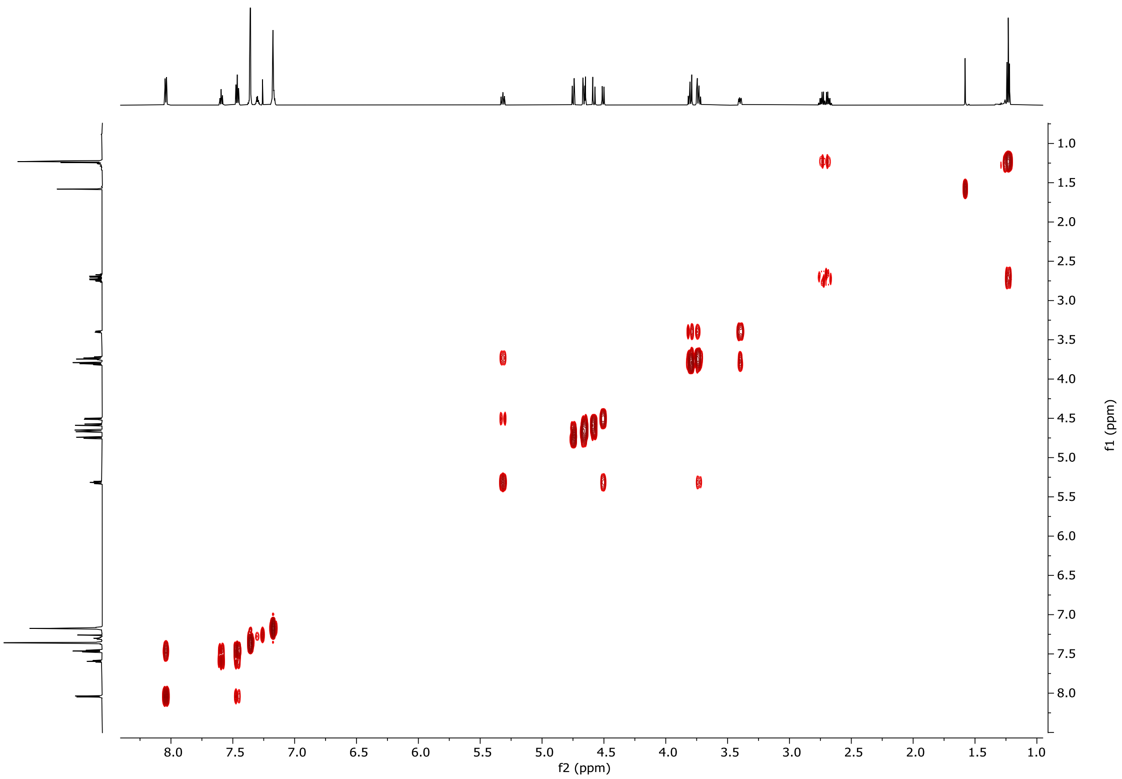

HSQC NMR of BB5 ($CDCl_3$)

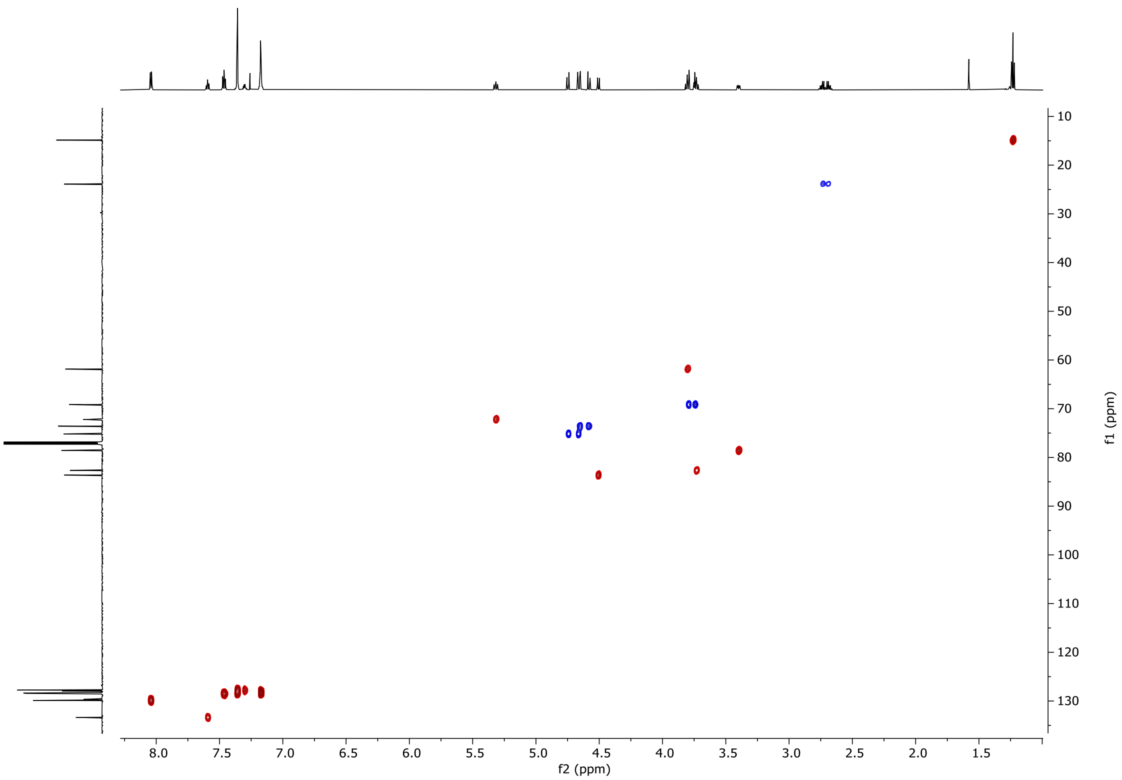

2.2 Synthesis of BB6

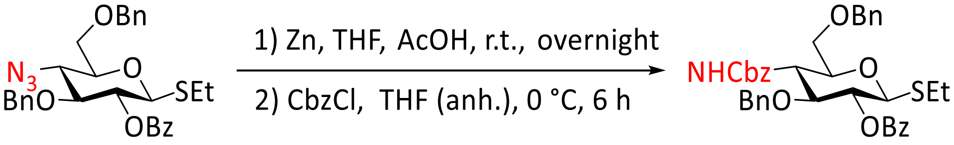

BB5 **BB6**

BB5 (400 mg, 0.75 mmol) was dissolved in THF/AcOH (10:1, 5.5 mL) and activated zinc (8.98 mmol, 12 equiv.) was added. The reaction mixture was stirred at r.t. overnight. Upon complete conversion (indicated by TLC), the reaction mixture was filtered and concentrated under reduced pressure to give the free amino intermediate that was readily used for the next step without further purification. The intermediate was dissolved in anhydrous THF (4 mL) under argon atmosphere and the reaction mixture was cooled to 0°C. After 10 min, $NaHCO_3$ (2.76 mmol, 2.0 equiv.) was added followed by CbzCl (1.66 mmol, 1.2 equiv.). The reaction mixture was stirred at 0°C for 6 h. Upon complete conversion indicated by TLC, the reaction mixture was filtered and the solvent was evaporated under reduced pressure. The crude was further purified via flash column chromatography (hexanes:EtOAc (3:1 to 0:1)). **BB6** was obtained as a white solid (350 mg, 74 %).

^{1}H NMR (700 MHz, $CDCl_3$) δ 8.11 – 8.01 (m, 2H), 7.58 (t, *J* = 7.4 Hz, 1H), 7.46 (t, *J* = 7.7 Hz, 2H), 7.39 – 7.23 (m, 10H), 7.14 (dq, *J* = 13.9, 6.7 Hz, 3H), 7.08 – 7.00 (m, 2H), 5.31 (t, *J* = 9.5 Hz, 1H), 5.05 (d, *J* = 3.8 Hz, 2H), 4.90 (d, *J* = 7.9 Hz, 1H), 4.64 – 4.43 (m, 5H), 4.21 (t, *J* = 9.6 Hz, 1H), 4.03 (dd, *J* = 10.7, 4.5 Hz, 1H), 3.78 – 3.65 (m, 2H), 3.56 (q, *J* = 9.7 Hz, 1H), 2.77 – 2.65 (m, 2H, SCH_2CH_3), 1.24 (t, *J* = 7.4 Hz, 3H, SCH_2CH_3).

^{13}C NMR (176 MHz, $CDCl_3$) δ 165.24, 155.75, 138.11, 137.54, 136.29, 133.26, 129.89, 129.81, 128.61, 128.49, 128.45, 128.42, 128.38, 128.32, 128.13, 128.11, 127.81, 127.79, 127.75, 127.71, 83.58, 79.16, 77.64, 77.26, 77.08, 76.90, 74.24, 73.54, 72.98, 69.50, 66.86, 54.05, 24.05 (S*C*H_2CH_3), 14.94 (S*C*H_2CH_3).

ESI-HRMS m/z 664.2360 $[M+Na]^+$ ($C_{37}H_{39}NNaO_7S$ requires 664.2345).

^{1}H NMR of BB6 (700 MHz, $CDCl_3$)

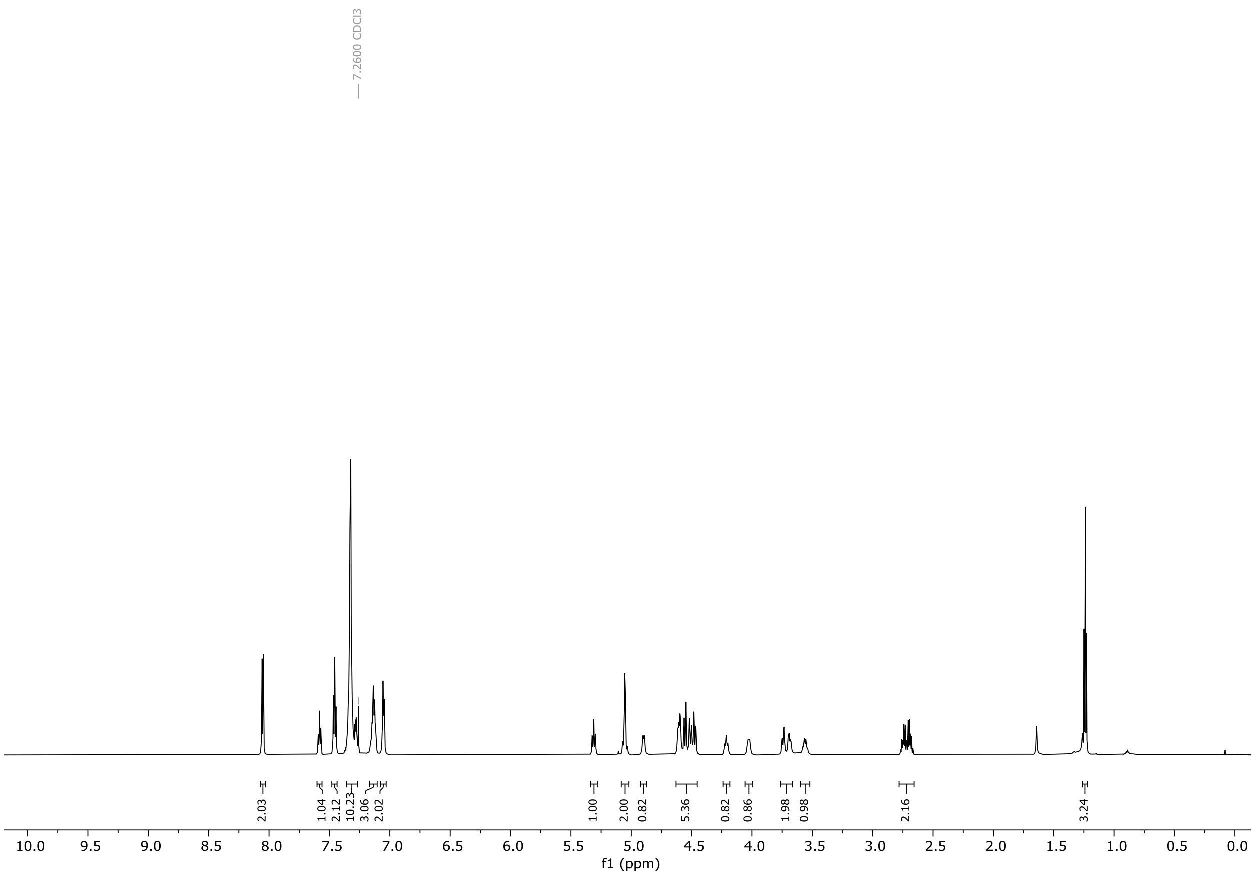

^{13}C NMR of BB6 (700 MHz, $CDCl_3$)

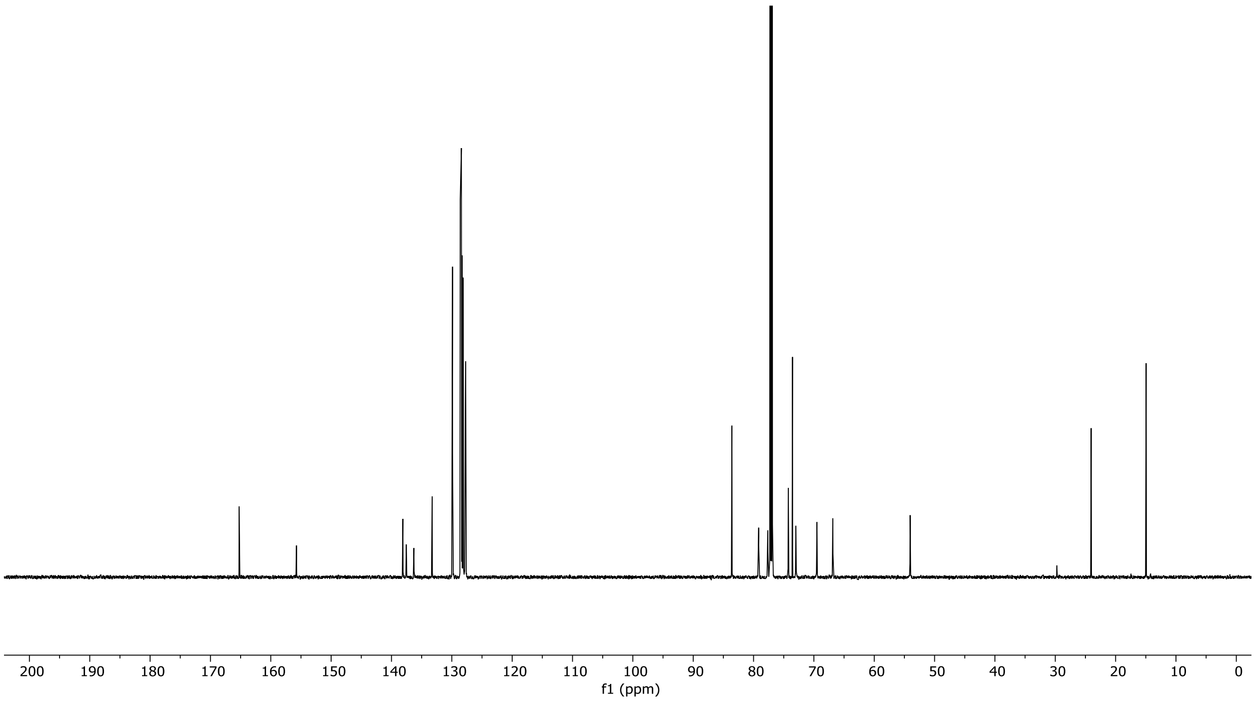

COSY NMR of BB6 ($CDCl_3$)

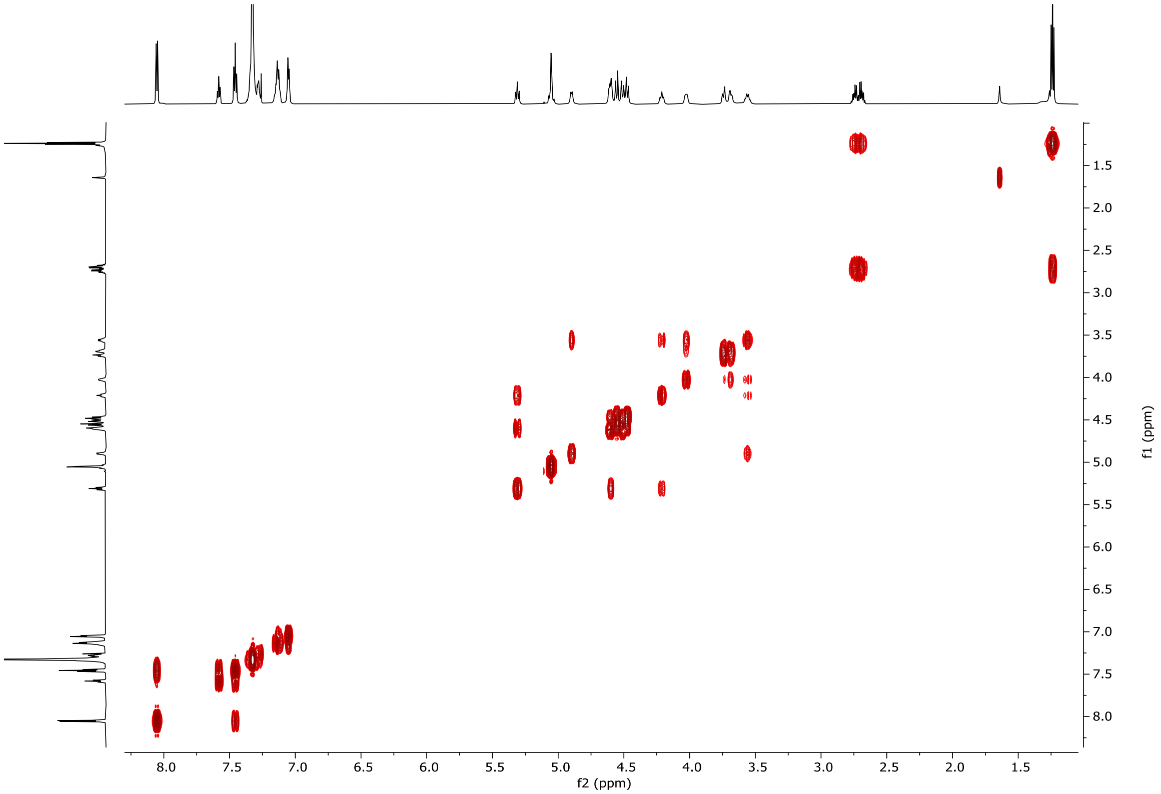

HSQC NMR of BB6 ($CDCl_3$)

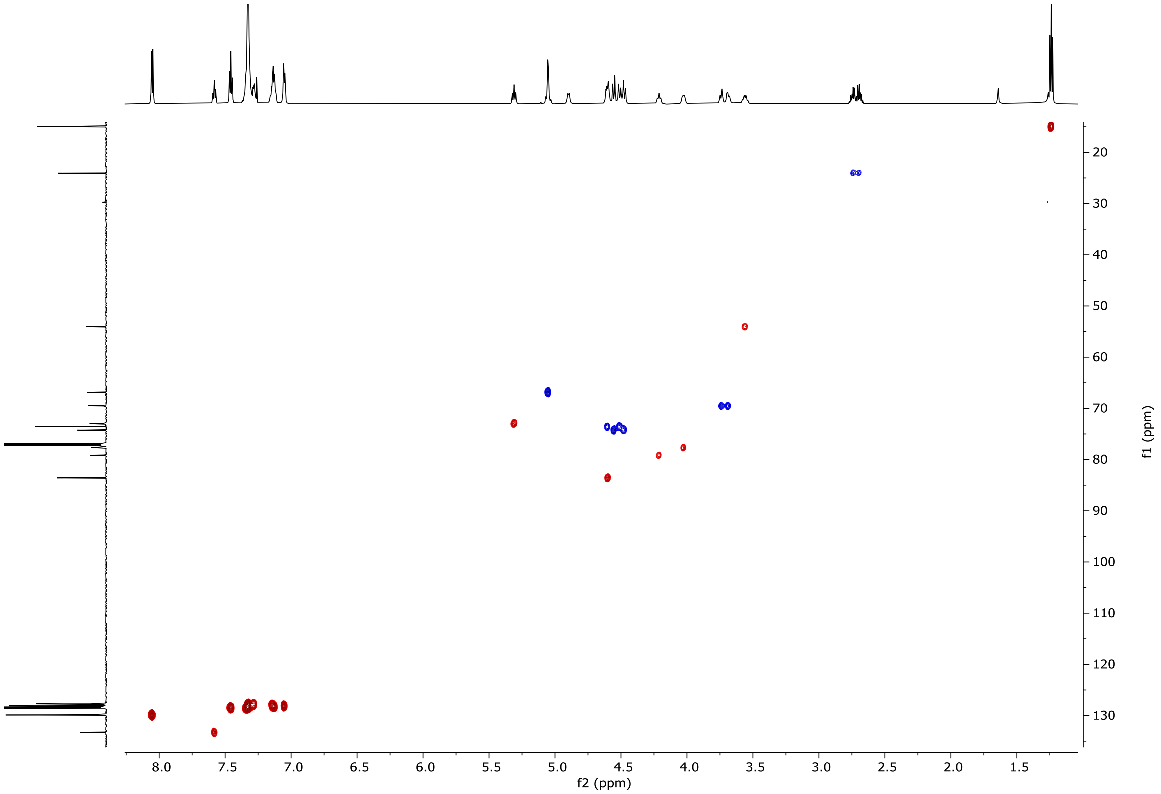

3 Automated glycan assembly

3.1 General materials and methods

The automated syntheses were performed on a home-built synthesizer developed at the Max Planck Institute of Colloids and Interfaces.[1] All solvents used were HPLC-grade. The solvents used for the building blocks, activator, TMSOTf and capping solutions were taken from an anhydrous solvent system (J.C. Meyer). The building blocks were co-evaporated three times with toluene and dried for 1 h on high vacuum before use. Oven-heated, argon-flushed flasks were used to prepare all moisture-sensitive solutions. Activator, capping, deprotection, acidic wash and building block solutions were freshly prepared and kept under argon during the automation run. All yields of products obtained by AGA were calculated on the basis of resin loading. Resin loading was determined following previously established procedures.[7]

3.2 Preparation of stock solutions

- **Building block solution**: Between 0.06 and 0.10 mmol of building block (depending on the BB, see Module C1 and C2) was dissolved in DCM (1 mL).
- **NIS/TfOH activator solution**: 1.35 g (6.0 mmol) of recrystallized NIS was dissolved in 40 mL of a 2:1 v/v mixture of anhydrous DCM and anhydrous dioxane. Then triflic acid (55 µL, 0.6 mmol) was added. The solution was kept at 0 °C (ice bath) for the duration of the automation run.
- **Fmoc deprotection solution**: A solution of $20\%_{v/v}$ piperidine in DMF was prepared.
- **Lev deprotection solution**: Hydrazine acetate (550 mg, 5.97 mmol) was dissolved in pyridine/AcOH/H_2O (40mL, v/v, 32:8:2) and sonicated for 10 min.
- **TMSOTf solution**: TMSOTf (0.45 mL, 2.49 mmol) was added to DCM (40 mL).
- **Capping solution**: A solution of $10\%_{v/v}$ acetic anhydride and $2\%_{v/v}$ methanesulfonic acid in DCM was prepared.

3.3 Modules for automated synthesis

3.3.1 Module A: Resin preparation

All automated syntheses were performed on 0.0125 mmol scale. Resin (**L**) is placed in the reaction vessel and swollen in DCM for 20 min at room temperature prior to the synthesis. During this time, all reagent lines needed for the synthesis are washed and primed. After the swelling, the resin is washed with DMF, THF, and DCM (three times each with 2 mL for 25 s).

3.3.2 Module B: Acidic wash with TMSOTf solution (20 min)

The resin is swollen in 2 mL DCM and the temperature of the reaction vessel adjusted to -20 °C. Upon reaching the low temperature, TMSOTf solution (1 mL) is added dropwise to the reaction vessel. After bubbling for 3 min, the acidic solution is drained and the resin washed with 2 mL DCM for 25 s.

Action	Cycles	Solution	Amount	T (°C)	Incubation time
**Cooling**	-	-	-	-20	(15 min)*
**Deliver**	1	DCM	2 mL	-20	-
**Deliver**	1	TMSOTf solution	1 mL	-20	3 min
**Wash**	1	DCM	2 mL	-20	25 sec

*Time required to reach the desired temperature.

3.3.3 Module C1: Thioglycoside glycosylation (35 min-55 min)

The building block solution (0.10 mmol of BB in 1 mL of DCM per glycosylation) is delivered to the reaction vessel. After the set temperature is reached, the reaction is started by dropwise addition of the NIS/TfOH activator solution (1.0 mL, 0.15 mmol). The glycosylation conditions (T_1, T_2, t_1, and t_2) are building block dependent and are reported in a table below. After completion of the reaction, the solution is drained and the resin was washed with DCM, DCM:dioxane (1:2, 3 mL for 20 s) and DCM (two times, each with 2 mL for 25 s). The temperature of the reaction vessel is increased to 25 °C for the next module. In case of a double cycle (C1*, *Double cycle), module C1 is repeated twice.

Action	Cycles	Solution	Amount	T (°C)	Incubation time
**Cooling**	-	-	-	T_1	-
**Deliver**	1	BB solution	1 mL	T_1	-
**Deliver**	1	NIS/TfOH activator solution	1 mL	T_1	-
**Reaction time (BB dependent)**	1	-	-	T_1 to T_2	t_1 t_2
**Wash**	1	DCM	2 mL	T_2	5 sec
**Wash**	1	DCM : Dioxane (1:2)	2 mL	T_2	20 sec
**Heating**	-	-	-	25	-
**Wash**	2	DCM	2 mL	> 0	25 sec

BB	Equiv.	t_1 (min)	T_1 (°C)	t_2 (min)	T_2 (°C)
**BB1**	6.5	5	-20	20	0
**BB3**	6.5	5	-20	20	0
**BB4**	6.5	5	-20	20	0
**BB6**	6.5	5	-20	20	0

3.3.4 Module C2: Glycosyl phosphate glycosylation (45 min)

The building block solution (0.06 mmol of BB in 1 mL of DCM per glycosylation) is delivered to the reaction vessel. After the set temperature is reached, the reaction is started by dropwise addition of the TMSOTf solution (1.0 mL, stoichiometric). After completion of the reaction, the solution is drained and the resin washed with DCM (six times, each with 2 mL for 25 s). The temperature of the reaction vessel is increased to 25 °C for the next module. In case of a double cycle (C2*, *Double cycle), module C2 is repeated twice.

Action	Cycles	Solution	Amount	T (°C)	Incubation time
**Cooling**	-	-	-	-30	-
**Deliver**	1	BB solution	1 mL	-30	-
**Deliver**	1	TMSOTf solution	1 mL	-30	-
**Reaction time (BB dependent)**	1	-	-	-30 to -10	5 min 40 min
**Wash**	1	DCM	2 mL	-10	5 sec
**Heating**	-	-	-	25	-
**Wash**	6	DCM	2 mL	> 0	25 sec

BB	Equiv.	t1 (min)	T1 (°C)	t2 (min)	T2 (°C)
**BB2**	5	5	-30	40	-10

3.3.5 Module D: Capping (30 min)

The resin is washed with DMF (two times with 2 mL for 25 s) and the temperature of the reaction vessel adjusted to 25 °C. A pyridine solution (2 mL, 10%$_{v/v}$ in DMF) is delivered into the reaction vessel. After 1 min, the reaction solution is drained and the resin washed with DCM (three times with 3 mL for 25 s). Capping solution (4 mL) is delivered into the reaction vessel. After 20 min, the reaction solution is drained and the resin washed with DCM (three times with 3 mL for 25 s).

Action	Cycles	Solution	Amount	T (°C)	Incubation time
**Heating**	-	-	-	25	(5 min)*
**Wash**	2	DMF	2 mL	25	25 sec
**Deliver**	1	10% Pyridine in DMF	2 mL	25	1 min
**Wash**	3	DCM	2 mL	25	25 sec
**Deliver**	1	Capping Solution	4 mL	25	20 min
**Wash**	3	DCM	2 mL	25	25 sec

*Time required to reach the desired temperature.

3.3.6 Module E1: Fmoc deprotection (9 min)

The resin is washed with DMF (three times with 2 mL for 25 s) and the temperature of the reaction vessel adjusted to 25 °C. Fmoc deprotection solution (2mL) is delivered to the reaction vessel and kept under Ar bubbling. After 5 min, the reaction solution is drained and the resin washed with DMF (three times with 3 mL for 25 s) and DCM (five times each with 2 mL for 25 s). The temperature of the reaction vessel is decreased to -20 °C for the next module.

Action	Cycles	Solution	Amount	T (°C)	Incubation time
**Wash**	3	DMF	2 mL	25	25 sec
**Deliver**	1	Fmoc depr. solution	2 mL	25	5 min
**Wash**	1	DMF	2 mL		
**Cooling**	-	-	-	-20	-
**Wash**	3	DMF	2 mL	< 25	25 sec
**Wash**	5	DCM	2 mL	< 25	25 sec

3.3.7 Module E2: Lev deprotection (90 min)

The resin is washed with DCM (three times with 2 mL for 25 s). DCM (1.3 mL) is delivered to the reaction vessel and the temperature of the reaction vessel is adjusted to 30 °C. Lev deprotection solution (2mL) is delivered to the reaction vessel, kept under pulsed Ar bubbling for 30 min. This procedure is repeated twice. The reaction solution is drained and the resin washed with DMF (three times with 3 mL for 25 s) and DCM (five times each with 2 mL for 25 s).

Action	Cycles	Solution	Amount	T (°C)	Incubation time
**Wash**	3	DMF	2 mL	25	25 sec
**Deliver**	2	Lev depr. solution	2 mL	25	30 min
**Wash**	1	DMF	2 mL	-	-
**Cooling**	-	-	-	-20	-
**Wash**	3	DMF	2 mL	< 25	25 sec
**Wash**	5	DCM	2 mL	< 25	25 sec

3.4 Post-AGA manipulations

3.4.1 Module F: On resin sulfation

The resin is suspended in 4 mL of a 0.5 M SO_3.py solution (DMF/pyridine, 1:1). The reaction is rotated for 24 h at 40 °C, after which time the resin is repeatedly washed with DMF (5 x 4 mL), MeOH (5 x 4 mL) and CH_2Cl_2 (5 x 4 mL).[8]

3.4.2 Module G: On-resin phosphorylation

Under argon, two vials sealed with a septum are prepared. Vial 1: dibenzyl *N,N*-diisopropylphosphoramidite (20 equiv. 0.32 mmol 110 microliter) and 2 ml anhydrous CH_2Cl_2. Vial 2: 5-benzylthio-1H-tetrazole (BTT, 30 equiv. 0.48 mmol, 92 mg), 2 mL anhydrous CH_2Cl_2 and 0.5 mL anhydrous ACN. The resin is first suspended in the contents of Vial 1, by withdrawing it into of a fritted syringe. The content of Vial 2 is then immediately withdrawn into the syringe. The syringe is capped and the reaction shaken for 1.5 h at room temperature. Upon completion, the reaction solution is discharged and the resin washed repeatedly with fresh CH_2Cl_2.

The resin is then subsequently subjected to oxidation by a mixture of pyridine (2.6 mL), I_2 (0.154 mmol, 40 mg, 22 equiv.), and H_2O (77.0 mmol, 1.4 mL, 11000 equiv.). The resin is suspended in the reaction solution mixture inside of a fritted syringe and gently shaken at room temperature for 2 h. The resin was repeatedly washed with pyridine (2 mL x 5) and DCM (2 mL x 3)..[9]

3.4.3 Module H1: On-resin methanolysis

The resin is suspended in THF (4 mL). MeONa in MeOH (0.5 M, 0.2 mL) is added and the suspension is gently shaken at room temperature. After micro-cleavage (see *Module I2*) indicates the complete removal of all ester groups, the resin is repeatedly washed with MeOH (3 x 2 mL) and DCM (3 x 2 mL).

3.4.4 Module H2: On-resin methanolysis

The resin is suspended in THF (4 mL). MeONa in MeOH (0.5 M, 0.4 mL) is added and the suspension is gently shaken at room temperature. After micro-cleavage (see *Module I2*) indicates the complete removal of all ester groups, the resin is repeatedly washed with MeOH (3 x 2 mL) and DCM (3 x 2 mL).

3.4.5 Module I1: Cleavage from solid support

The oligosaccharides are cleaved from the solid support using a continuous-flow photoreactor as described previously. A 15% MeOH in CH_2Cl_2 solvent system is used due to the presence of ionic functional groups.[10]

3.4.6 Module I2: Micro-cleavage from solid support

Trace amount of resin (around 20 beads) is dispersed in DCM (0.1 mL) and MeOH (0.01 mL) and irradiated with a UV lamp (6 W, 356 nm) for 10 minutes. ACN (10 µL) is then added to the resin and the resulting solution analyzed by MALDI.

3.4.7 Module J1: Hydrogenolysis

The crude compound obtained from *Module I1* is dissolved in 2 mL of EtOAc:*t*BuOH:H_2O (1.5:1.5:1). $Pd(OH)_2/C$ (10-20%$_w$, moistened with water) is added and the reaction stirred in a pressurized reactor under H_2 pressure (4 bar). The reaction progress is monitored to avoid undesired side products formation (*i.e.* degradation of reducing end).[11] Upon completion, the reaction is filtered (PTFE 0.45 µm 25 mm syringe filter, Fisher scientific) and washed with EtOAc, H_2O, and ACN (4 mL each). The filtrates are concentrated *in vacuo.*

3.4.8 Module J2: Hydrogenolysis

The crude compound obtained from *Module I1* is dissolved in 2 mL of EtOAc:*t*BuOH:H_2O (1.5:1.5:1). $Pd(OH)_2/C$ (10-20%$_w$, moistened with water) and 0.2 mL of formic acid is added to the stirred flask, the reaction purged for 5 min with N_2 balloon, and equipped with a H_2 balloon. The reaction progress is monitored to avoid undesired side products formation (*i.e.* degradation of reducing end).[11] Upon completion, the reaction is filtered (PTFE 0.45 µm 25 mm syringe filter, Fisher scientific) and washed with EtOAc, H_2O, and ACN (4 mL each). The filtrates are concentrated *in vacuo.*

3.4.9 Module K: Purification

The final compounds are analyzed using analytical reversed phase HPLC (Agilent 1200 Series, Methods A1, B1, and C1). The purification of the crudes is conducted using reversed phase HPLC (Agilent 1200 Series, Method A2, B2, C2 and D).

- **Method A1**: (Hypercarb column, ThermoFisher scientific, 150 x 4.6 mm, 3 µm) flow rate of 0.7 mL/min with H_2O (0.01 M NH_4HCO_3) and ACN as eluents [isocratic (5 min), linear gradient to 60% ACN (30 min), linear gradient to 100% ACN (5 min), isocratic 100% ACN (5 min)]. ELSD Detector: 100 °C.

- **Method A2 (Prep)**: (Hypercarb column, ThermoFisher scientific, 150 x 10 mm, 5 µm), flow rate of 3 mL/min with H_2O (0.01 M NH_4HCO_3) and ACN as eluents [isocratic (5 min), linear gradient to 30% ACN (60 min), linear gradient to 100% ACN (5 min), isocratic 100% ACN (5 min)]. ELSD Detector: 100 °C.

- **Method B1**: (Hypercarb column, ThermoFisher scientific, 150 x 4.6 mm, 3 µm) flow rate of 0.7 mL/min with H_2O (0.1% formic acid) and ACN as eluents [isocratic (5 min), linear gradient to 60% ACN (30 min), linear gradient to 100% ACN (5 min), isocratic 100% ACN (5 min)]. ELSD Detector: 60 °C.

- **Method B2 (Prep)**: (Hypercarb column, ThermoFisher scientific, 150 x 10 mm, 5 µm), flow rate of 3 mL/min with H_2O (0.1% formic acid) and ACN as eluents [isocratic (5 min), linear gradient to 60% ACN (30 min), linear gradient to 100% ACN (5 min), isocratic 100% ACN (5 min)]. ELSD Detector: 60 °C.

- **Method C1**: (Synergi Hydro RP18 column, Phenomenex, 250 x 4.6 mm), flow rate of 1.0 mL/min with H_2O (0.1% formic acid) and ACN as eluents [isocratic (5 min), linear gradient to 30% ACN (30 min), linear gradient to 100% ACN (5 min), isocratic 100% ACN (5 min)]. ELSD Detector: 60 °C.

- **Method C2 (Prep)**: (Synergi Hydro RP18 column, Phenomenex, 250 x 10 mm) flow rate of 4.0 mL/min with H_2O (0.1% formic acid) and ACN as eluents [isocratic (5 min), linear gradient to 30% ACN (30 min), linear gradient to 100% ACN (5 min), isocratic 100% ACN (5 min)]. ELSD Detector: 60 °C.

- **Method D**: Sephadex® LH-20 column with H_2O:MeOH (1:1) as eluent, isocratic.

Following final purification, all deprotected products are lyophilized on a Christ Alpha 2-4 LD plus freeze dryer prior to characterization.

3.5 Oligosaccharides synthesis

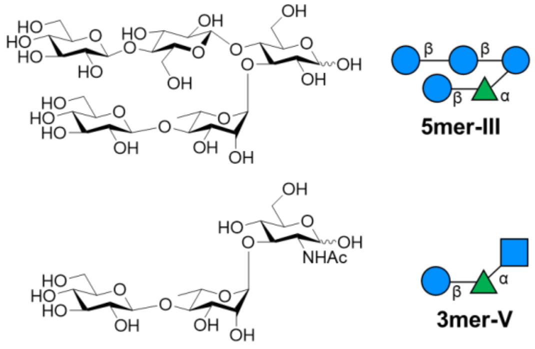

Figure S02
Oligosaccharides synthesized by AGA in previous works.[3, 12]

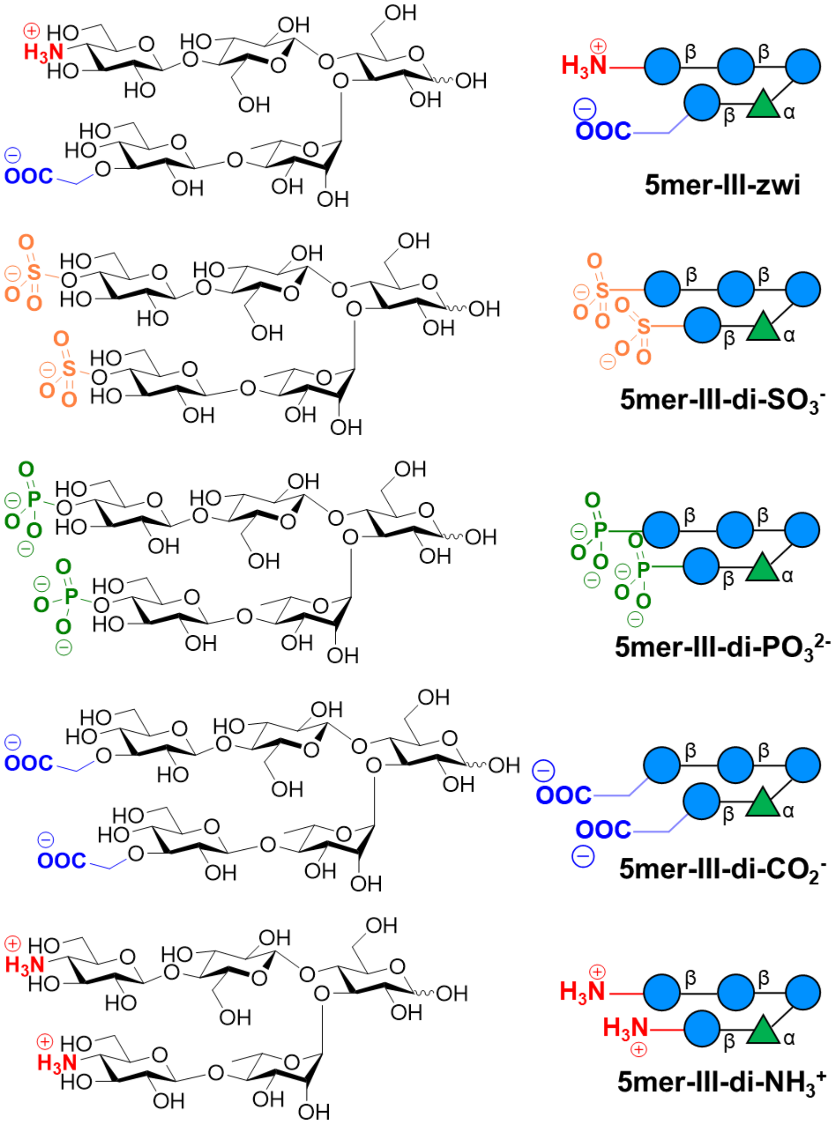

Figure S03
Oligosaccharides synthesized by AGA in this work. A systematic terminology to standardize the name and representation of the glycan hairpins discussed in the previous paper can be found in the SI.[12]

3.5.1 5mer-III-zwi

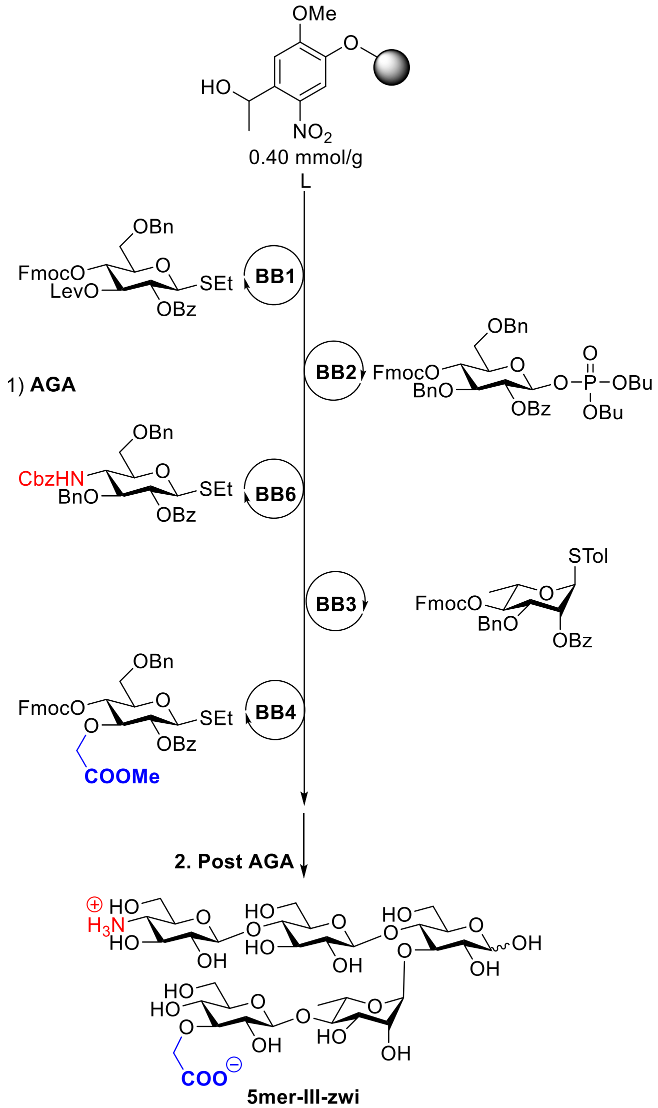

5mer-III-zwi

Step		Modules	Notes
		**A**	**L** swelling
**AGA**	**BB1**	**B**, **C1**, **D**, **E1**	**C1**: (**BB1**, -20 °C for 5 min, 0 °C for 20 min)
	**BB2**	**B**, **C2***, **D**, **E1**	**C2***: (**BB2**, -30 °C for 5 min, -10 °C for 40 min) *Double cycle
	**BB6**	**B**, **C1**, **D**, **E2**	**C1**: (**BB6**, -20 °C for 5 min, 0 °C for 20 min)
	**BB3**	**B**, **C1**, **D**, **E1**	**C1**: (**BB3**, -20 °C for 5 min, 0 °C for 20 min)
	**BB4**	**B**, **C1***, **D**, **E1**	**C1***: (**BB4**, -20 °C for 5 min, 0 °C for 20 min) *Double cycle
**Post-AGA**	**Methanolysis**	**H2**	(6 h)
	**Photocleavage**	**I1**	
	**Hydrogenolysis**	**J1**	(5.5 h)
	**Purification**	**K**	(Method B2: t_R = 19.4 min)

Automated synthesis, global deprotection, and purification afforded **5mer-III-zwi** as a white solid (1.8 mg, 17% overall yield).

^{1}H NMR (700 MHz, D_2O) δ 5.26 (d, *J* = 1.7 Hz, 0.6H, H-1β Rha), 5.20 (d, *J* = 1.7 Hz, 0.4H, H-1α Rha), 5.14 (d, *J* = 3.8 Hz, 0.4H, H-1α Glc), 4.68 (d, *J* = 7.9 Hz, 1H, H-1 Glc), 4.61 (d, *J* = 8.0 Hz, 0.6H, H-1β Glc), 4.46 (d, *J* = 7.9 Hz, 1H, H-1 Glc), 4.42 (dd, *J* = 18.7, 7.2 Hz, 2H, 1× H-1 Glc, 1× H-5 Rha), 4.26 – 4.15 (m, 2H, CH_2 next to COOH), 4.11 (dt, *J* = 9.5, 2.8 Hz, 1H), 4.04 (dt, *J* = 12.3, 2.1 Hz, 1H), 4.00 (t, *J* = 2.5 Hz, 1H), 3.96 (t, *J* = 9.7 Hz, 0H), 3.92 – 3.88 (m, 1H), 3.88 – 3.84 (m, 2H), 3.84 – 3.73 (m, 5H), 3.69 – 3.64 (m, 1H), 3.64 (s, 0H), 3.60 – 3.50 (m, 3H), 3.45 (t, *J* = 9.3 Hz, 1H), 3.42 – 3.33 (m, 3H), 3.32 – 3.26 (m, 3H), 3.05 (s, 1H), 1.27 (dd, *J* = 6.4, 3.9 Hz, 3H, CH_3-6 Rha).
^{13}C NMR (176 MHz, D_2O) δ 178.84 (COOH), 103.56 (C-1 Glc), 102.73 (C-1 Glc), 101.00 (C-1 Glc), 99.96 (C-1α Rha), 99.84 (C-1β Rha), 95.70 (C-1β Glc), 92.03 (C-1α Glc), 85.90, 81.56, 80.15, 76.58, 75.65, 75.41, 75.36, 75.17, 74.32, 74.16, 73.73, 73.52, 73.11, 73.09, 72.58, 72.37, 71.44, 70.79, 70.10, 70.05, 69.88, 69.50, 66.79, 66.73, 60.98, 60.49, 60.41, 59.50, 59.42, 52.03, 16.53 (C-6 Rha).
ESI-HRMS m/z 870.3091 $[M+H]^+$ ($C_{32}H_{56}NO_{26}$ requires 870.3091).

RP-HPLC of 5mer-III-zwi (ELSD trace, Method B1, t_R = 17.7, 18.3 min)

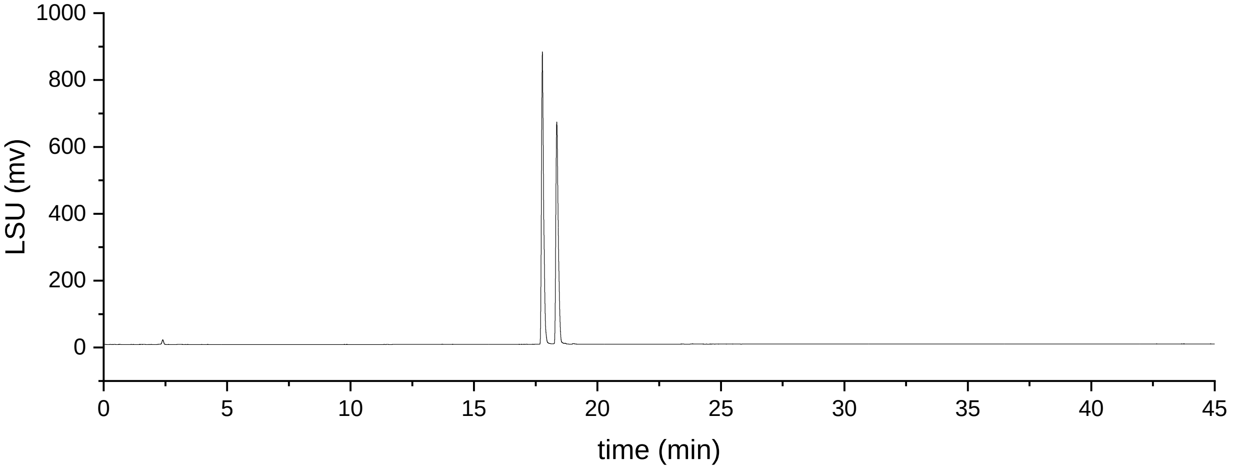

^{1}H NMR of 5mer-III-zwi (700 MHz, D_2O)

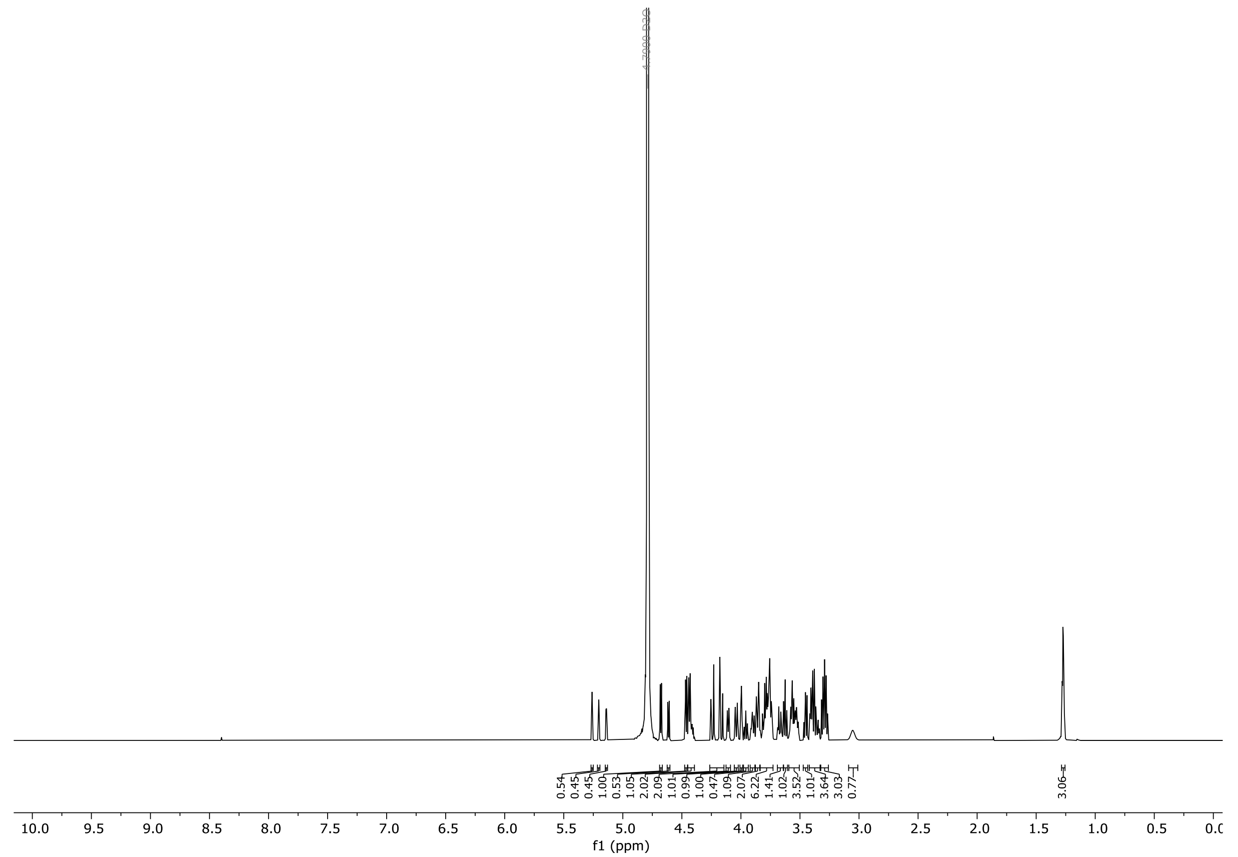

^{13}C NMR of 5mer-III-zwi (176 MHz, D_2O)

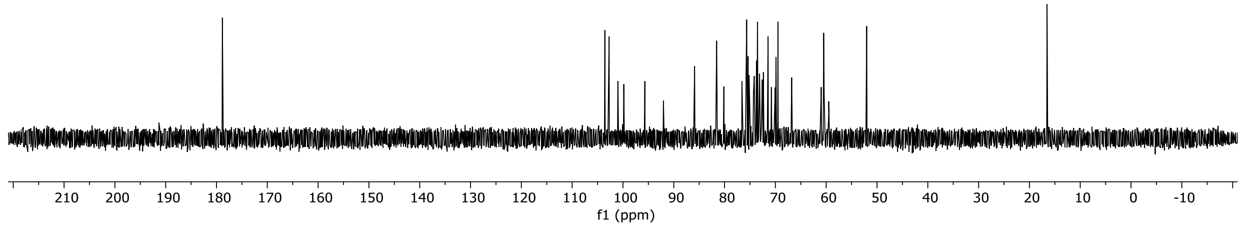

COSY NMR of 5mer-III-zwi (D_2O)

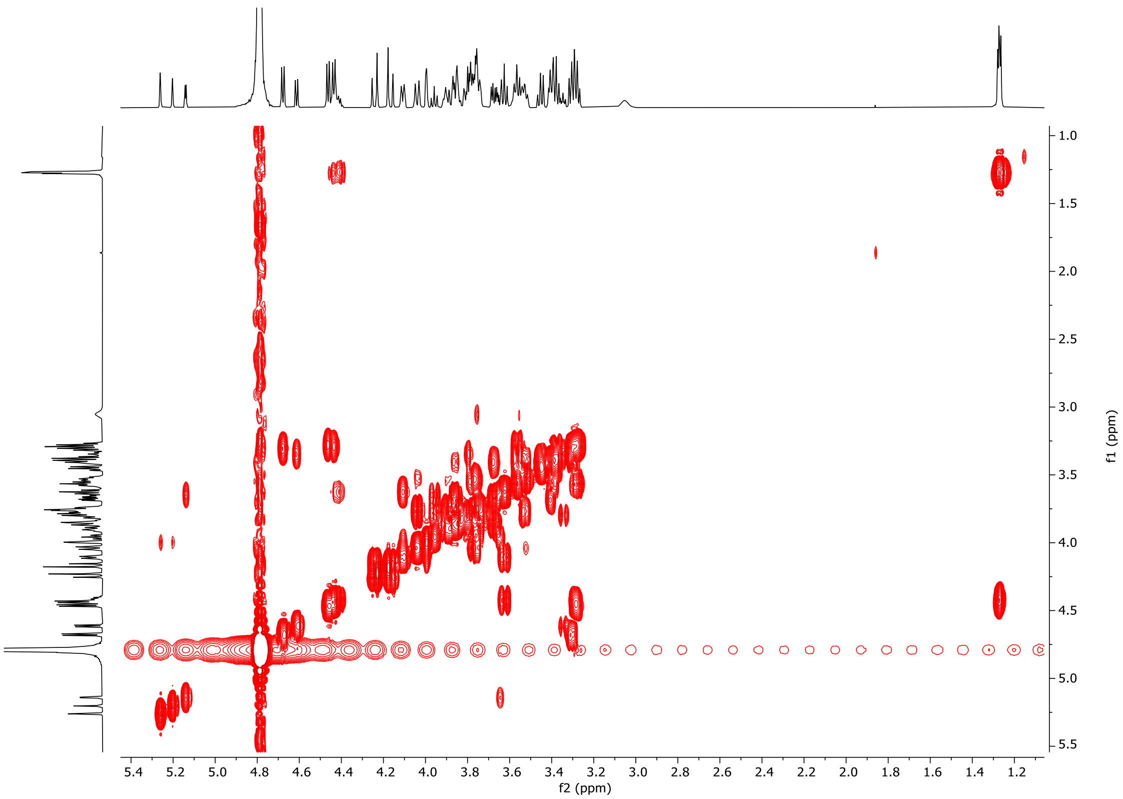

HSQC NMR of 5mer-III-zwi (D_2O)

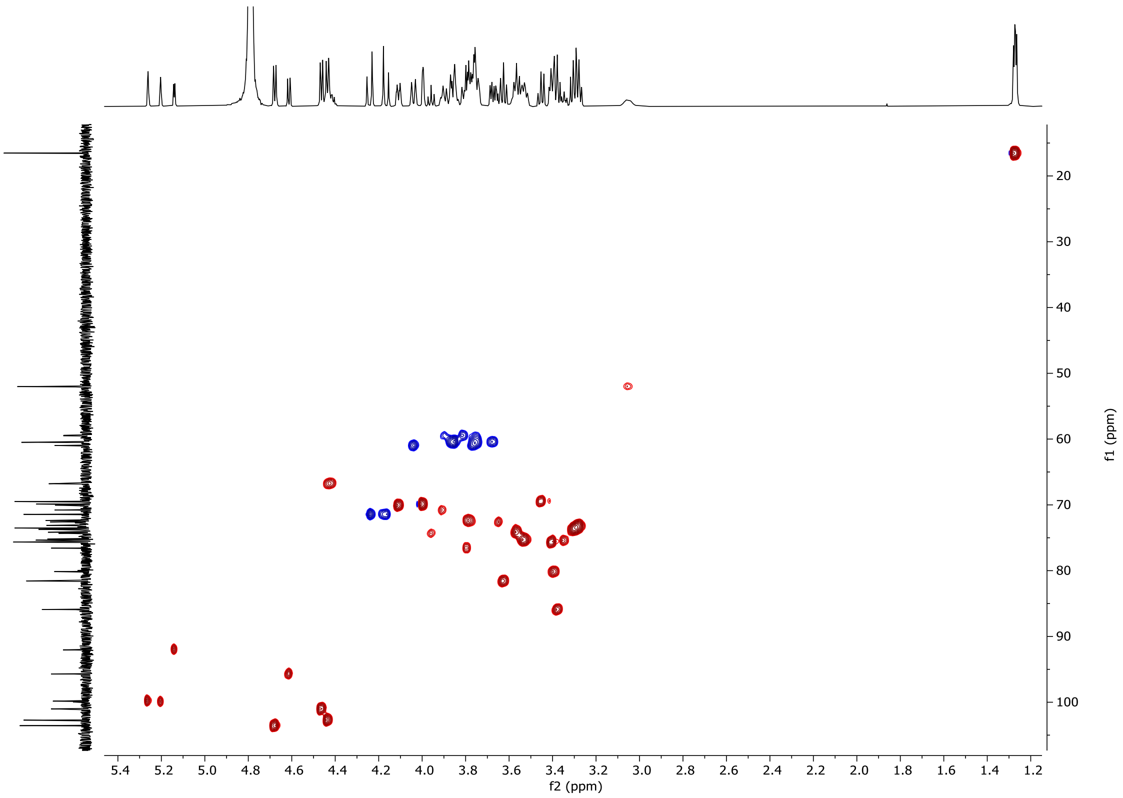

3.5.2 5mer-III-di-SO_3^-

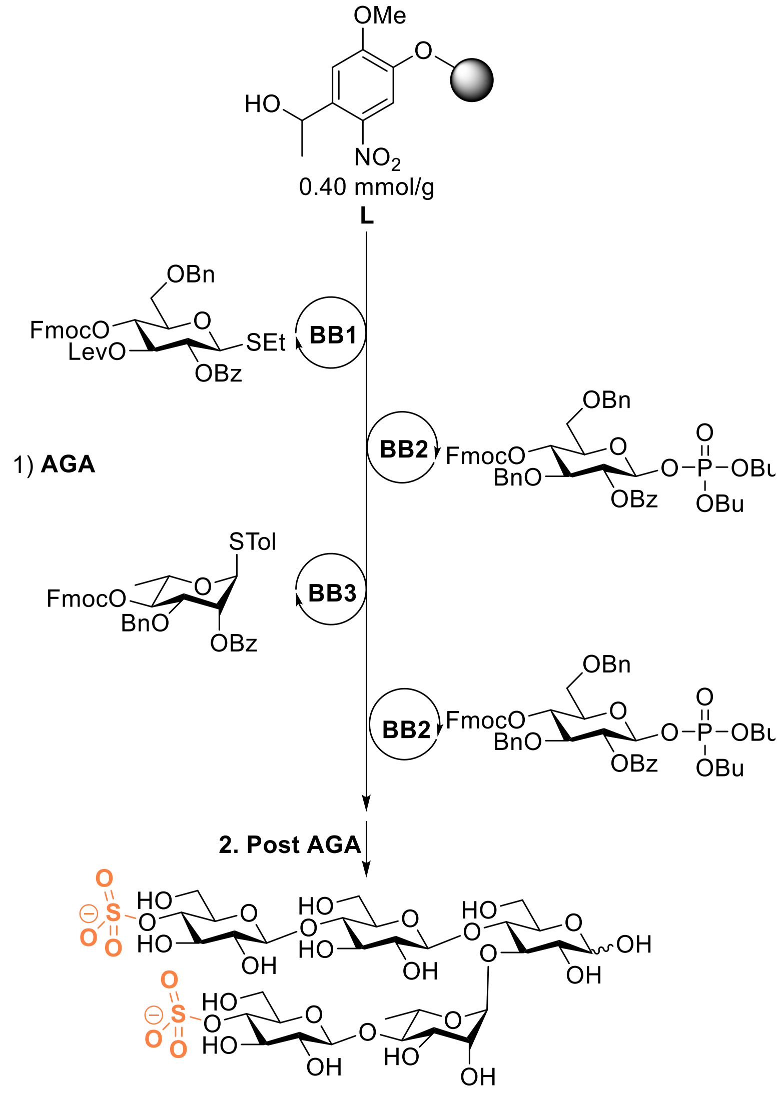

5mer-III-di-SO_3^-

Step		Modules	Notes
AGA		**A**	**L** swelling
	**BB1**	**B**, **C1**, **D**, **E1**	**C1**: (**BB1**, -20 °C for 5 min, 0 °C for 20 min)
	**BB2**	**B**, **C2***, **D**, **E2**	**C2***: (**BB2**, -30 °C for 5 min, -10 °C for 40 min) *Double cycle
	**BB3**	**B**, **C1**, **D**, **E1**	**C1**: (**BB3**, -20 °C for 5 min, 0 °C for 20 min)
	**BB2**	**B**, **C2***, **C2**, **D** **E1**	**C2***, **C2**: (**BB2**, -30 °C for 5 min, -10 °C for -40 min) *Double cycle
Post-AGA	**Sulfation**	**F**	(24 h)
	**Methanolysis**	**H1**	(9 h)
	**Photocleavage**	**I1**	
	**Hydrogenolysis**	**J1**	(7 h)
	**Purification**	**K**	(Method A2: t_R = 47.6, 49.3 min, Method D)

Automated synthesis, sulfation, global deprotection, and purification afforded **5mer-III-di-SO_3^-** as a white solid (3.2 mg, 26% overall yield).

^{1}H NMR (700 MHz, D_2O) δ 5.25 (d, *J* = 1.8 Hz, 0.6H, H-1β Rha), 5.19 (d, *J* = 1.7 Hz, 0.4H, H-1α Rha), 5.15 (d, *J* = 3.8 Hz, 0.4H, H-1α Glc), 4.69 (d, *J* = 8.1 Hz, 1H, H-1 Glc), 4.61 (d, *J* = 8.0 Hz, 0.6H, H-1β Glc), 4.48 (dd, *J* = 7.9, 2.0 Hz, 2H, 2×H-1 Glc), 4.42 (tt, *J* = 16.4, 6.4 Hz, 1H, H-5 Rha), 4.13 – 4.09 (m, 2H), 4.08 – 4.06 (m, 1H), 4.04 (dt, *J* = 12.3, 2.4 Hz, 1H), 4.00 (dt, *J* = 3.4, 1.6 Hz, 1H), 3.92 (td, *J* = 13.5, 2.5 Hz, 3.4H), 3.83 – 3.76 (m, 4H), 3.74 – 3.69 (m, 4H), 3.65 (qd, *J* = 8.7, 5.6 Hz, 2.4H), 3.60 (td, *J* = 9.0, 1.9 Hz, 1H), 3.56 – 3.52 (m, 2.6H), 3.46 (t, *J* = 9.2 Hz, 1H), 3.35 (td, *J* = 9.9, 7.9 Hz, 2.6H), 3.31 (dd, *J* = 9.5, 8.0 Hz, 1H), 1.29 (dd, *J* = 6.4, 3.8 Hz, 3H, CH_3-6 Rha).

^{13}C NMR (176 MHz, D_2O) δ 103.25 (C-1 Glc), 102.47 (C-1 Glc), 101.00 (C-1 Glc), 100.13 (C-1α Rha), 99.96 (C-1β Rha), 95.74 (C-1β Glc), 92.01(C-1α Glc), 81.17, 81.11, 80.01, 79.95, 77.37, 76.72, 76.36, 75.23, 75.20, 75.08, 74.46, 74.28, 74.18, 73.92, 73.71, 73.02, 72.46, 72.42, 70.79, 70.10, 70.05, 69.99, 67.00, 60.99, 60.94, 60.43, 59.51, 59.42, 16.61(C-6 Rha).

ESI-HRMS m/z 993.1685 $[M+Na]^+$ ($C_{30}H_{50}NaO_{31}S_2^{2-}$ requires 993.1686).

RP-HPLC of 5mer-III-di-SO_3^- (ELSD trace, Method A1, t_R = 22.4, 23.13 min)

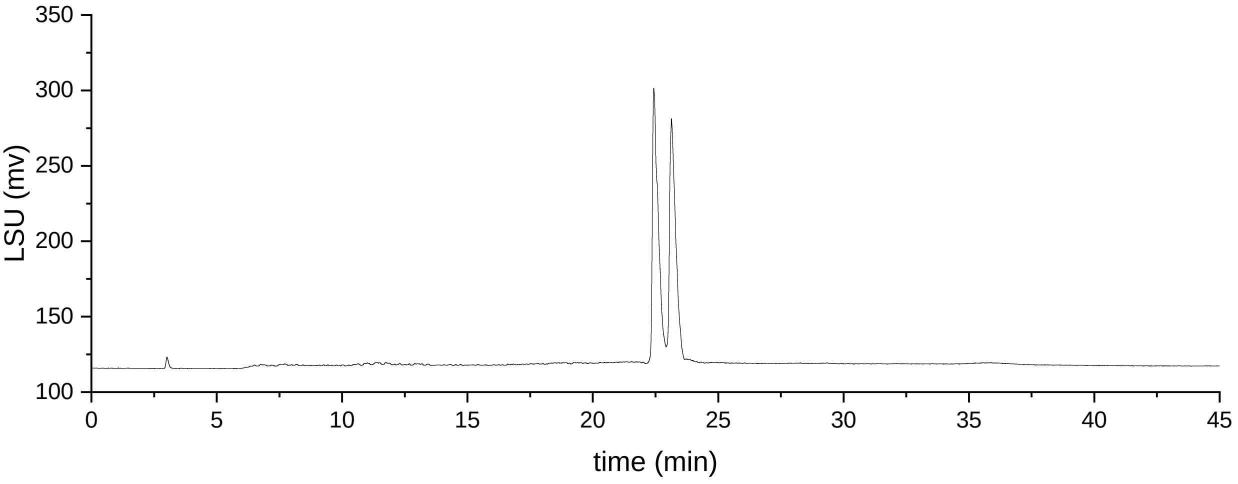

^{1}H NMR of 5mer-III-di-SO_3^- (700 MHz, D_2O)

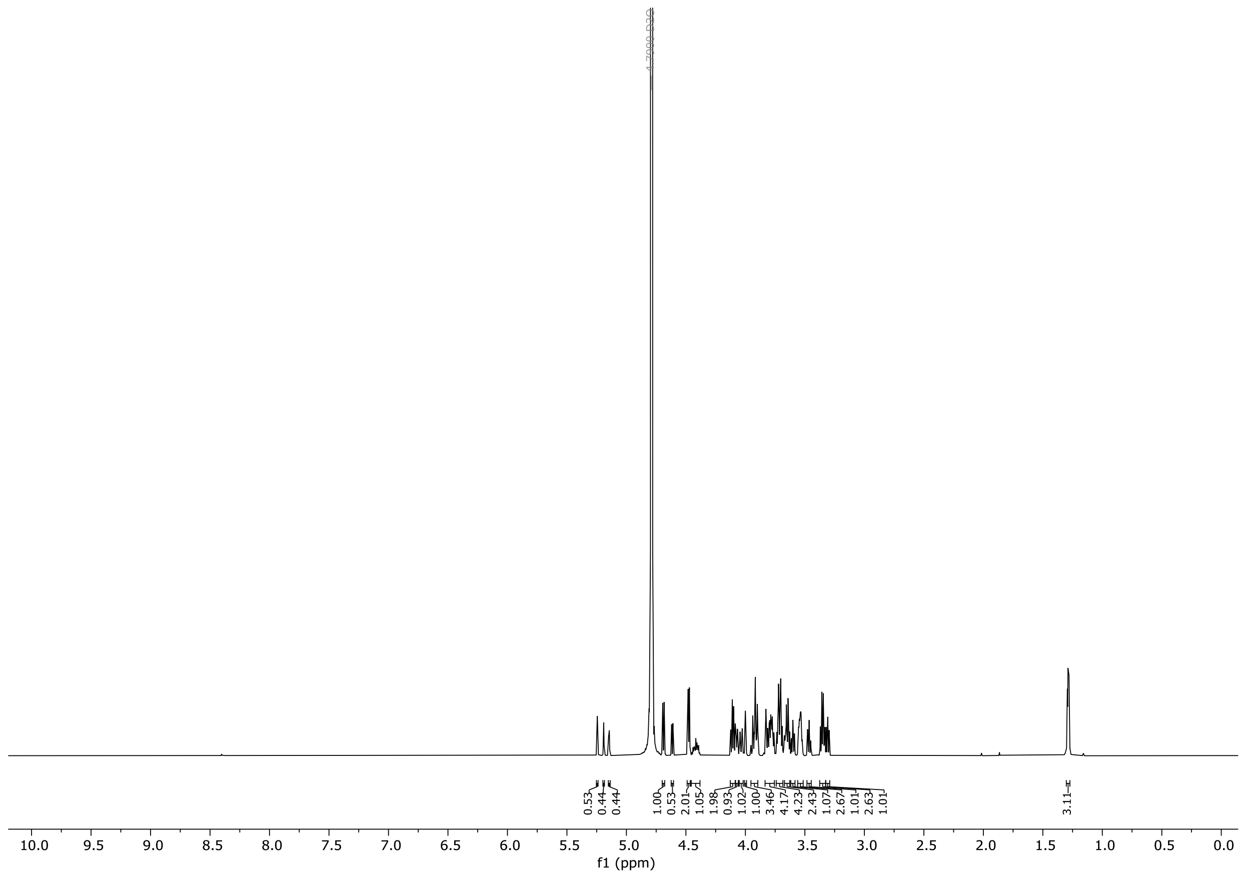

^{13}C NMR of 5mer-III-di-SO_3^- (176 MHz, D_2O)

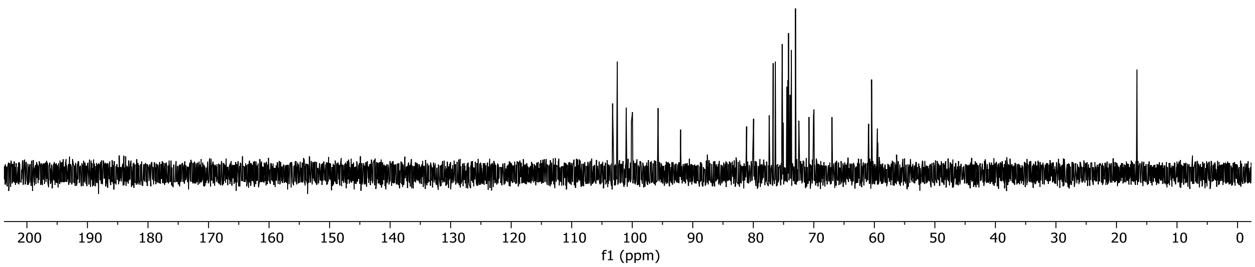

COSY NMR of 5mer-III-di-SO_3^- (D_2O)

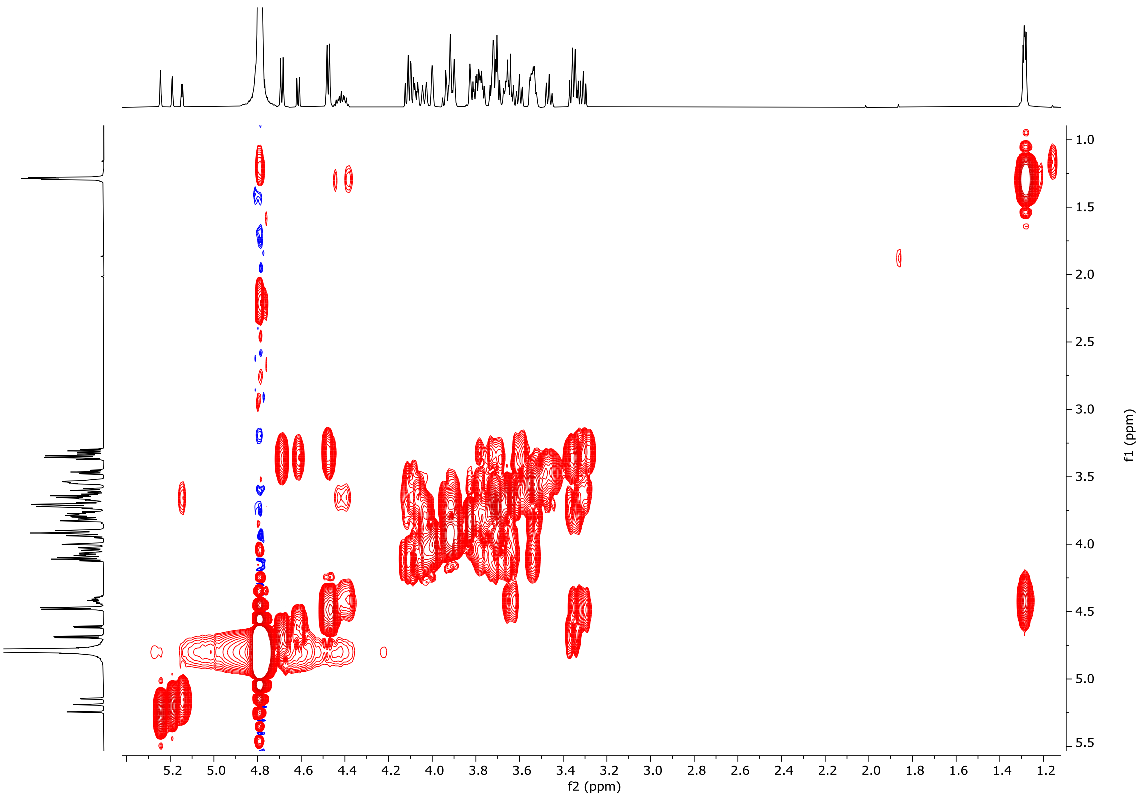

HSQC NMR of 5mer-III-di-SO_3^- (D_2O)

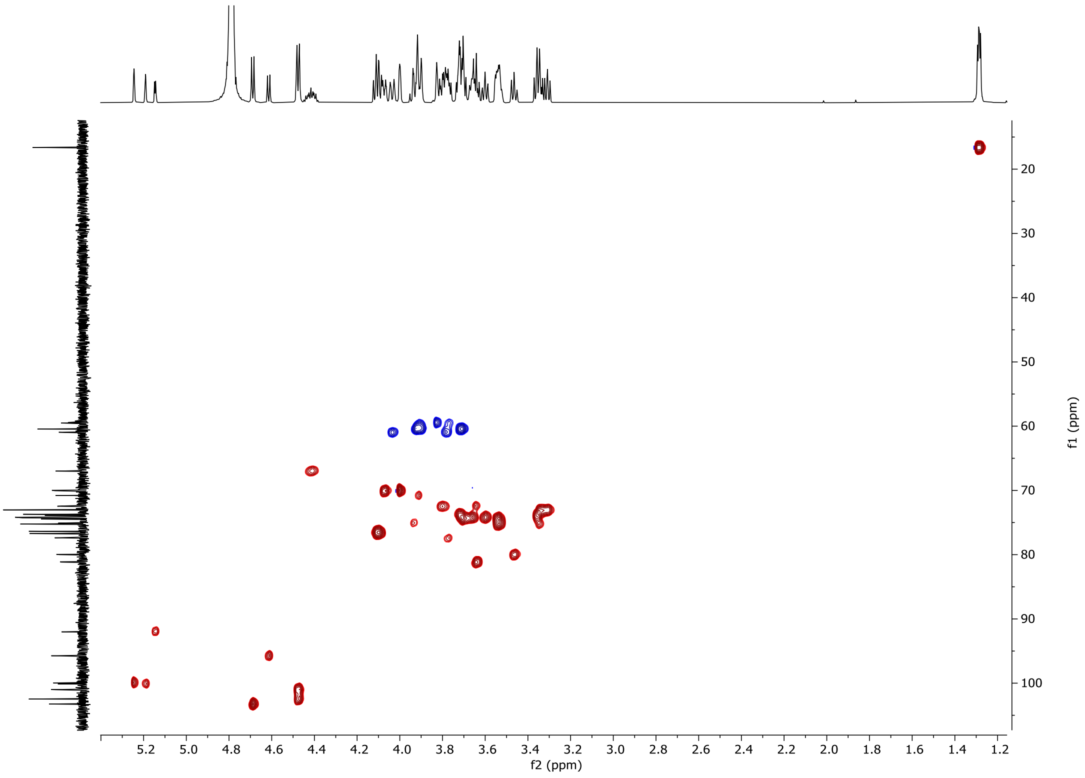

3.5.3 5mer-III-di-PO_3^{2-}

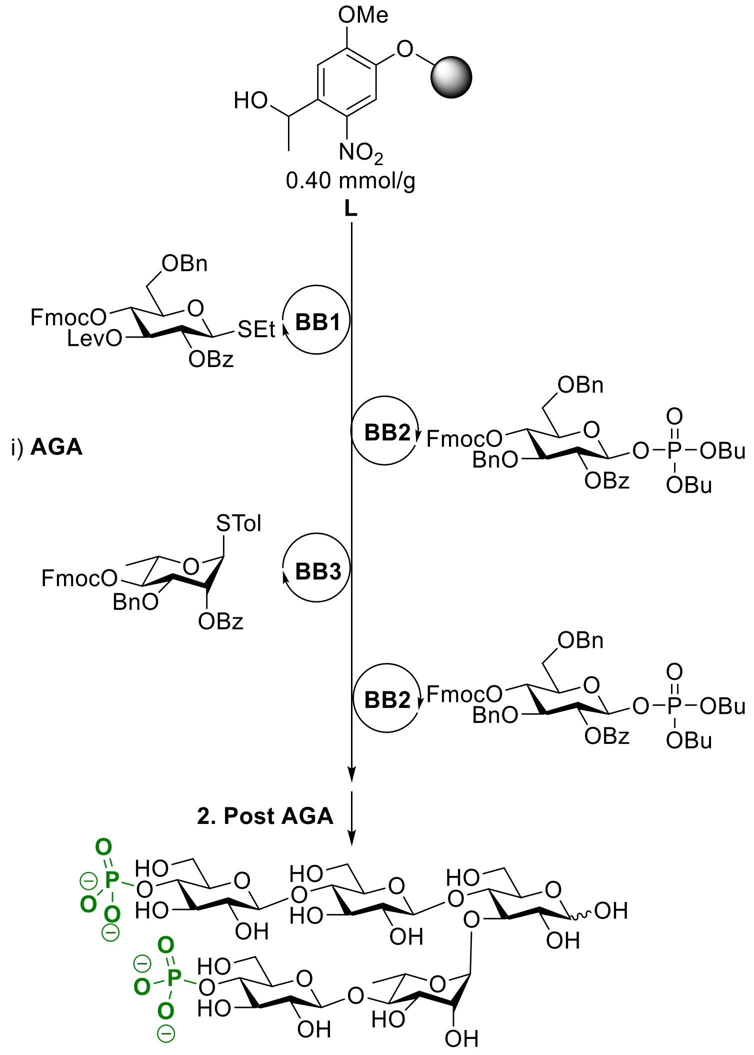

5mer-III-di-PO_3^{2-}

Step		Modules	Notes
**AGA**		**A**	**L** swelling
	**BB1**	**B**, **C1**, **D**, **E1**	**C1**: (**BB1**, -20 °C for 5 min, 0 °C for 20 min)
	**BB2**	**B**, **C2***, **D, E2**	**C2***: (**BB2**, -30 °C for 5 min, -10 °C for 40 min) *Double cycle
	**BB3**	**B**, **C1**, **D**, **E1**	**C1**: (**BB3**, -20 °C for 5 min, 0 °C for 20 min)
	**BB2**	**B**, **C2***, **C2**, **D** **E1**	**C2***, **C2**: (**BB2**, -30 °C for 5 min, -10 °C for -40 min) *Double cycle
**Post-AGA**	**Phosphorylation**	**F**	(3.5 h)
	**Methanolysis**	**H1**	(7 h)
	**Photocleavage**	**I1**	
	**Hydrogenolysis**	**J1**	(12 h)
	**Purification**	**K**	(Method D)

Automated synthesis, phosphorylation, global deprotection, and purification afforded **5mer-III-di-PO_3^{2-}** as a white solid (2.1 mg, 21% overall yield).

^{1}H NMR (700 MHz, D_2O) δ 5.24 (d, *J* = 1.8 Hz, 0.6H, H-1β Rha), 5.19 (d, *J* = 1.8 Hz, 0.4H, H-1α Rha), 5.14 (d, *J* = 3.8 Hz, 0.4H, H-1α Glc), 4.67 (d, *J* = 8.0 Hz, 1H, H-1 Glc), 4.61 (d, *J* = 8.0 Hz, 0.6H, H-1β Glc), 4.48 (d, *J* = 7.9 Hz, 1H, H-1 Glc), 4.45 (d, *J* = 7.9 Hz, 1H, H-1 Glc), 4.43 – 4.35 (m, 1H, H-5 Rha), 4.07 (dd, *J* = 9.6, 3.0 Hz, 1H), 4.05 – 4.02 (m, 1H), 4.00 (dt, *J* = 3.4, 1.6 Hz, 1H), 3.95 – 3.90 (m, 1.8H), 3.88 – 3.73 (m, 9.6H), 3.72 – 3.62 (m, 3.4H), 3.59 (td, *J* = 9.0, 1.7 Hz, 2H), 3.56 – 3.51 (m, 1.6H), 3.48 – 3.43 (m, 2H), 3.36 – 3.34 (m, 0.6H), 3.33 – 3.29 (m, 3H), 1.28 (dd, *J* = 6.4, 3.2 Hz, 3H, CH_3-6 Rha).
^{13}C NMR (176 MHz, D_2O) δ 103.41 (C-1 Glc), 102.50 (C-1 Glc), 101.01 (C-1 Glc), 100.18 (C-1α Rha), 99.97 (C-1β Rha), 95.76 (C-1β Glc), 92.00 (C-1α Glc), 81.06, 79.79, 77.63, 75.70, 75.24, 75.03, 74.04, 73.11, 72.58, 70.81, 70.15, 70.11, 70.04, 67.11, 60.48, 16.67 (C-6 Rha).
^{31}P NMR (162 MHz, D_2O) δ 2.25, 2.00.
ESI-HRMS m/z 995.2028 $[M+Na]^+$ ($C_{30}H_{54}NaO_{31}P_2$ requires 995.2022).

RP-HPLC of 5mer-III-di-PO_3^{2-} (ELSD trace, Method C1, t_R = 3.6, 3.9 min)

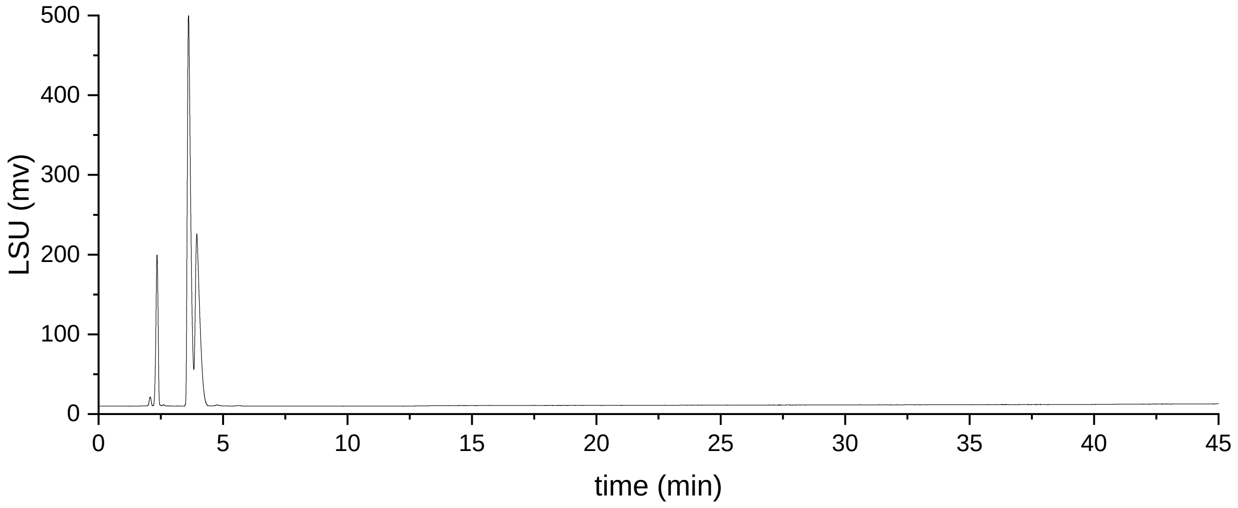

^{1}H NMR of 5mer-III-di-PO_3^{2-} (700 MHz, D_2O)

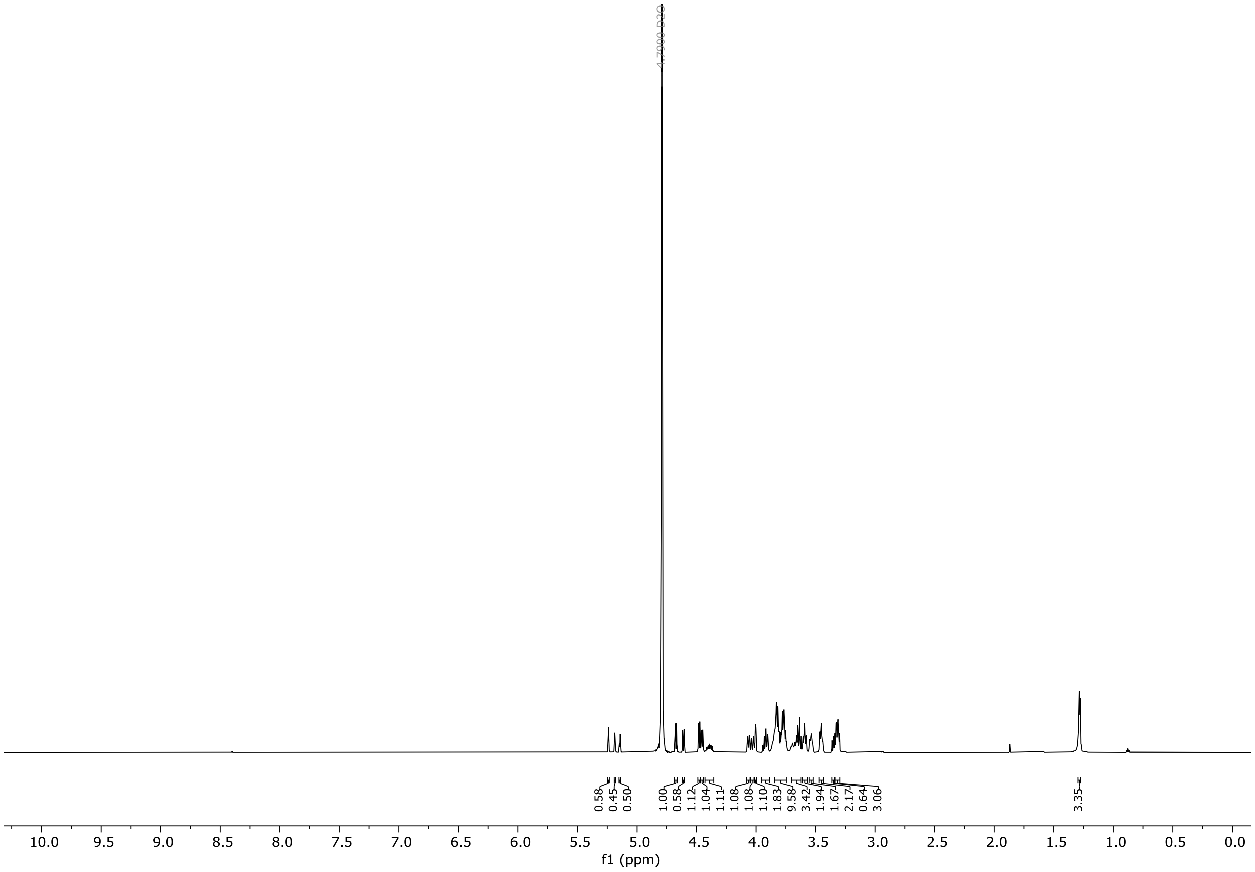

^{13}C NMR of 5mer-III-di-PO_3^{2-} (176 MHz, D_2O)

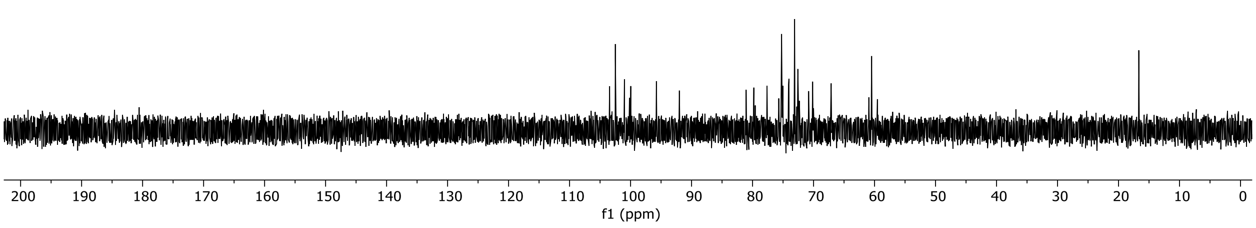

^{31}P NMR of 5mer-III-di-PO_3^{2-} (162 MHz, D_2O)

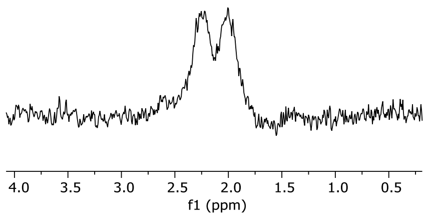

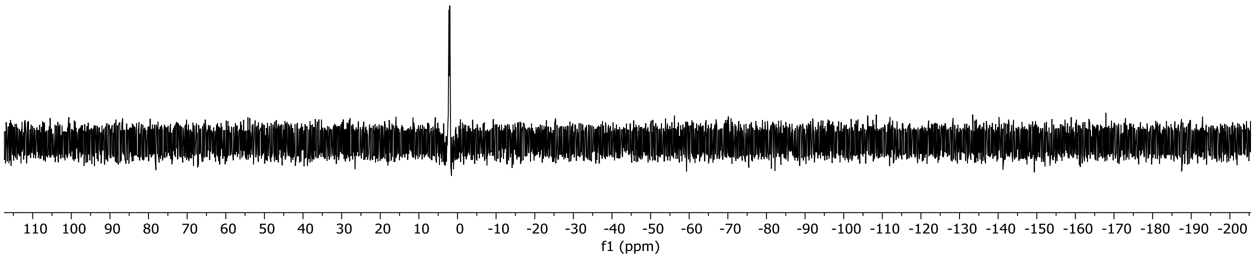

COSY NMR of 5mer-III-di-PO_3^{2-} (D_2O)

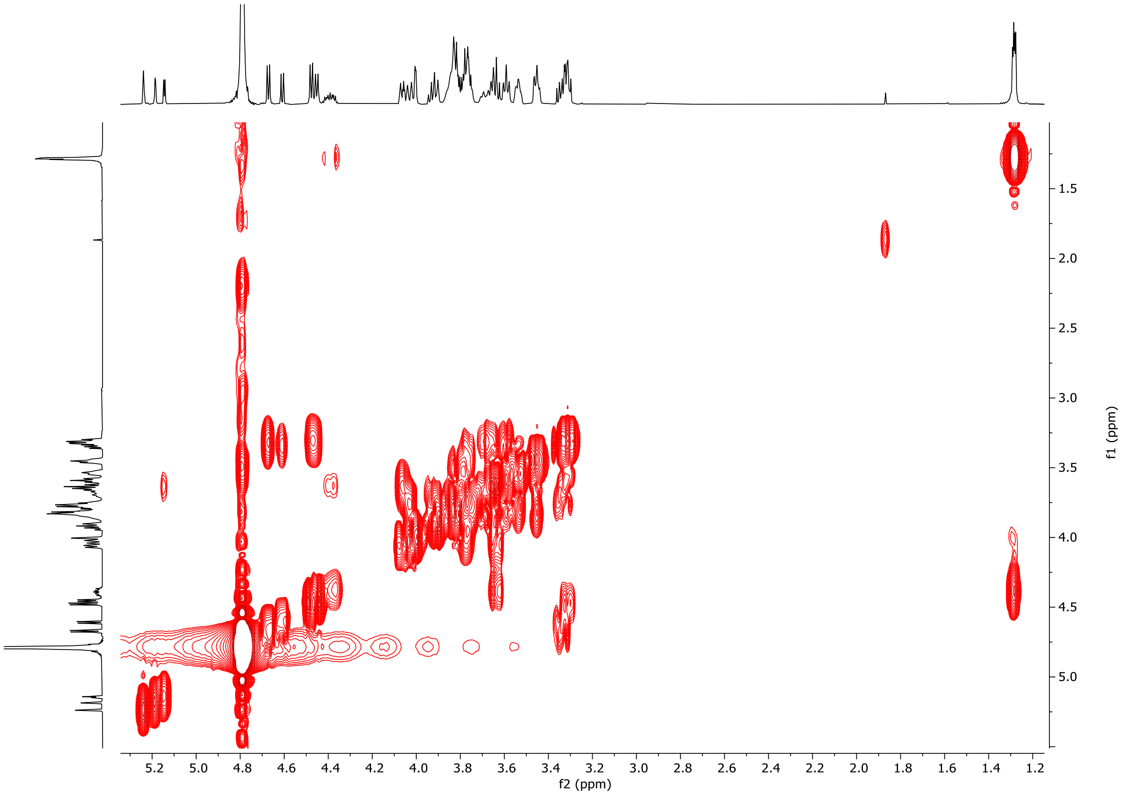

HSQC NMR of 5mer-III-di-PO_3^{2-} (D_2O)

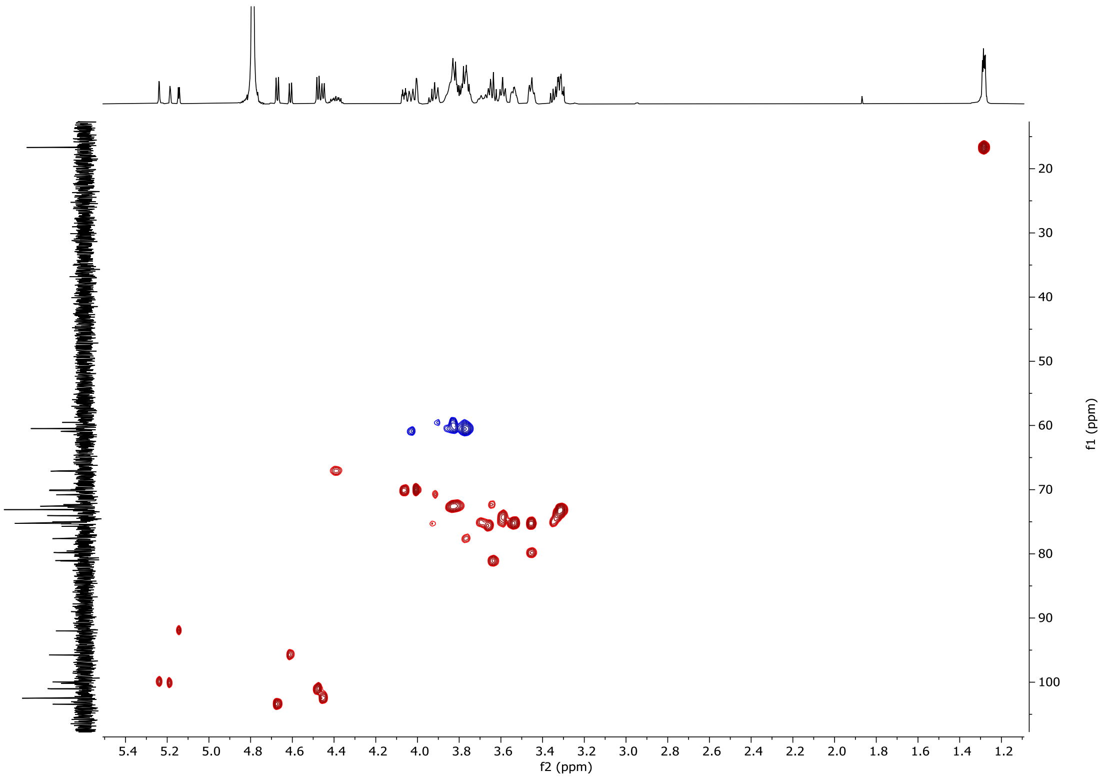

3.5.4 5mer-III-di-CO_2^-

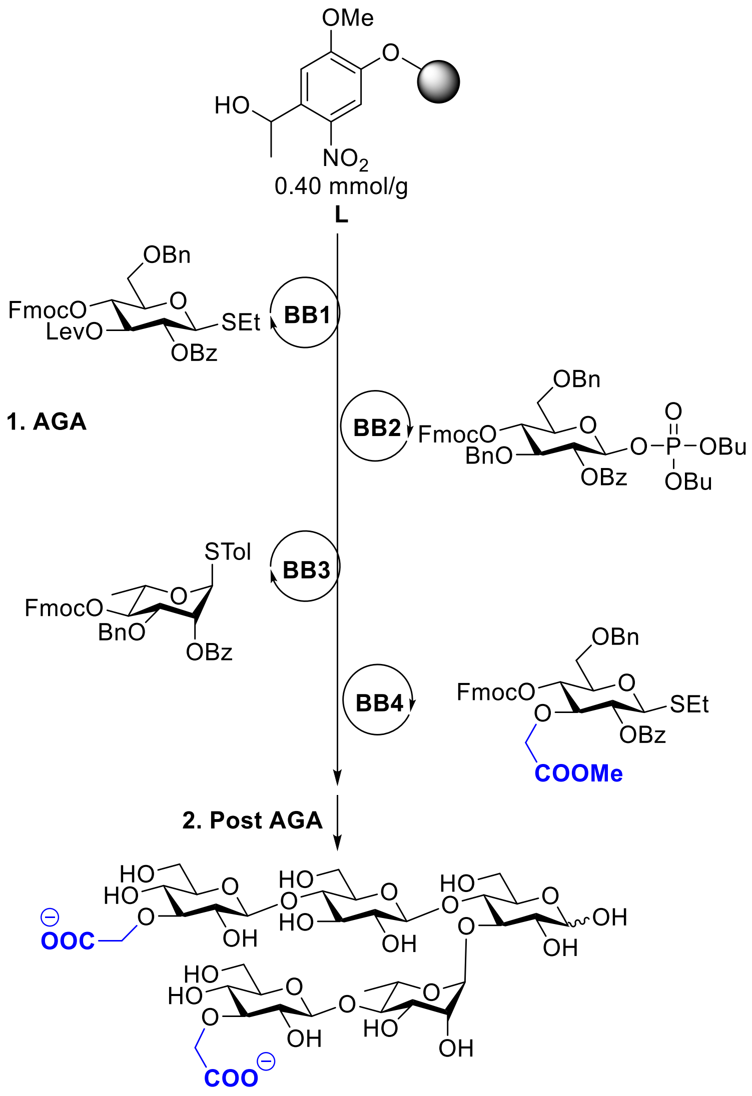

5mer-III-di-CO_2^-

Step		Modules	Notes
		**A**	**L** swelling
	**BB1**	**B**, **C1**, **D**, **E1**	**C1**: (**BB1**, -20 °C for 5 min, 0 °C for 20 min)
**AGA**	**BB2**	**B**, **C2***, **D**, **E2**	**C2***: (**BB2**, -30 °C for 5 min, -10 °C for 40 min) *Double cycle
	**BB3**	**B**, **C1**, **D**, **E1**	**C1**: (**BB3**, -20 °C for 5 min, 0 °C for 20 min)
	**BB4**	**B**, **C1***, **C1**, **D**, **E1**	**C1***, **C1**: (**BB4**, -20 °C for 5 min, 0 °C for 20 min) *Double cycle
	**Methanolysis**	**H2**	(8 h)
**Post-AGA**	**Photocleavage**	**I1**	
	**Hydrogenolysis**	**J1**	(5 h)
	**Purification**	**K**	(Method C2: t_R = 19.1 min)

Automated synthesis, global deprotection, and purification afforded **5mer-III-di-CO_2^-** as a white solid (2.7 mg, 23% overall yield).

^{1}H NMR (700 MHz, D_2O) δ 5.26 (d, *J* = 1.7 Hz, 0.6H, H-1β Rha), 5.20 (d, *J* = 1.7 Hz, 0.4H, H-1α Rha), 5.14 (d, *J* = 3.8 Hz, 0.4H, H-1α Glc), 4.68 (d, *J* = 7.8 Hz, 1H, H-1 Glc), 4.61 (d, *J* = 8.0 Hz, 0.6H, H-1β Glc), 4.47 (dd, *J* = 7.8, 5.2 Hz, 2×H-1 Glc), 4.45 – 4.38 (m, 1H, H-5 Rha), 4.21 (qd, *J* = 16.8, 7.4 Hz, 4H, 2× CH_2 next to COOH), 4.12 (ddd, *J* = 9.4, 3.4, 2.1 Hz, 1H), 4.07 (dt, *J* = 12.3, 2.3 Hz, 1H), 4.01 (dd, *J* = 3.3, 1.8 Hz, 1H), 3.95 (t, *J* = 9.7 Hz, 0.4H), 3.91 (tt, *J* = 9.4, 2.8 Hz, 1H), 3.88 – 3.84 (m, 1.4H), 3.83 – 3.74 (m, 4.6H), 3.71 – 3.65 (m, 2.4H), 3.63 (t, *J* = 9.7 Hz, 1H), 3.59 (dq, *J* = 10.2, 2.3 Hz, 1H), 3.58 – 3.51 (m, 2.6H), 3.51 – 3.48 (m, 1H), 3.47 – 3.43 (m, 1H), 3.43 – 3.39 (m, 2H), 3.39 – 3.34 (m, 3.6H), 3.32 (dd, *J* = 9.2, 7.8 Hz, 1H), 3.29 (ddd, *J* = 9.4, 7.9, 1.3 Hz, 1H), 1.28 (dd, *J* = 6.4, 3.7 Hz, 3H, CH_3-6 Rha).
^{13}C NMR (176 MHz, D_2O) δ 176.71 (COOH), 176.13 (COOH), 103.33 (C-1 Glc), 102.73 (C-1 Glc), 100.96 (C-1 Glc), 100.06(C-1α Rha), 99.94 (C-1β Rha), 95.70 (C-1β Glc), 92.02 (C-1α Glc), 85.93, 85.20, 81.20, 80.44, 76.90, 75.55, 75.45, 75.37, 75.25, 75.18, 74.63, 74.08, 73.55, 73.18, 72.87, 72.54, 72.36, 70.79, 70.11, 70.06, 69.94, 69.83, 69.56, 68.54, 66.78, 61.03, 60.41, 60.28, 59.50, 59.42, 16.54 (C-6 Rha).
ESI-HRMS m/z 929.2988 $[M+H]^+$ ($C_{34}H_{57}O_{29}$ requires 929.2986).

RP-HPLC of 5mer-III-di-CO_2^- (ELSD trace, Method C1, t_R = 18.3, 18.9 min)

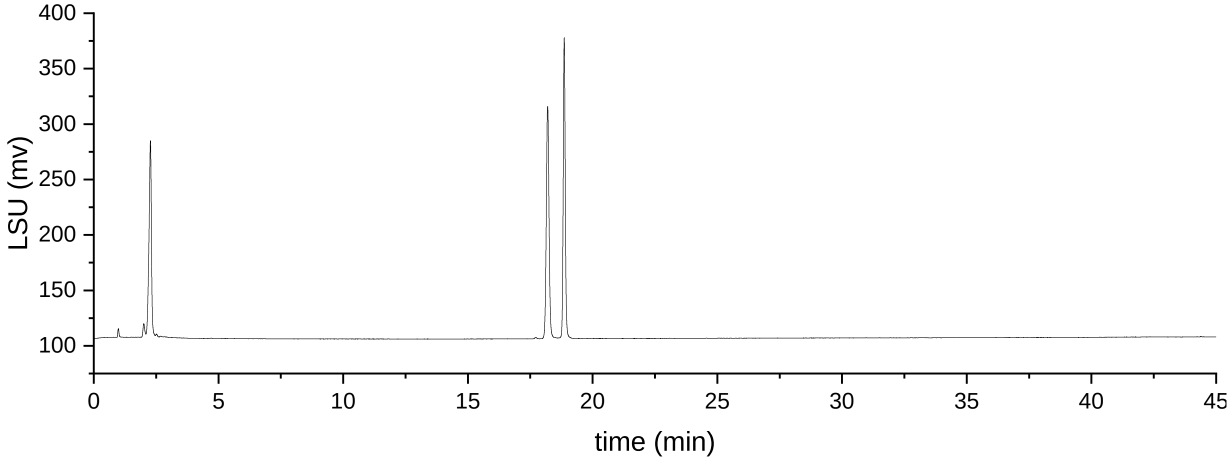

^{1}H NMR of 5mer-III-di-CO_2^- (700 MHz, D_2O)

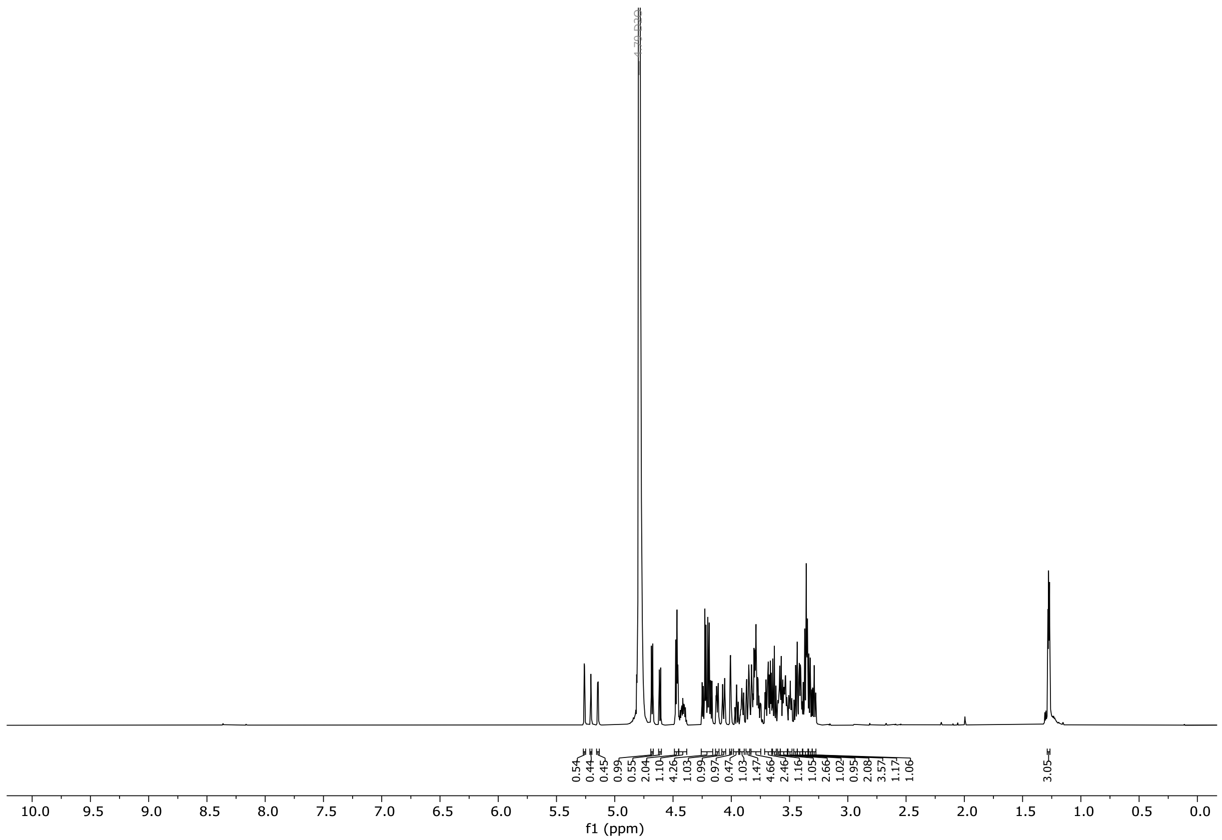

^{13}C NMR of 5mer-III-di-CO_2^- (176 MHz, D_2O)

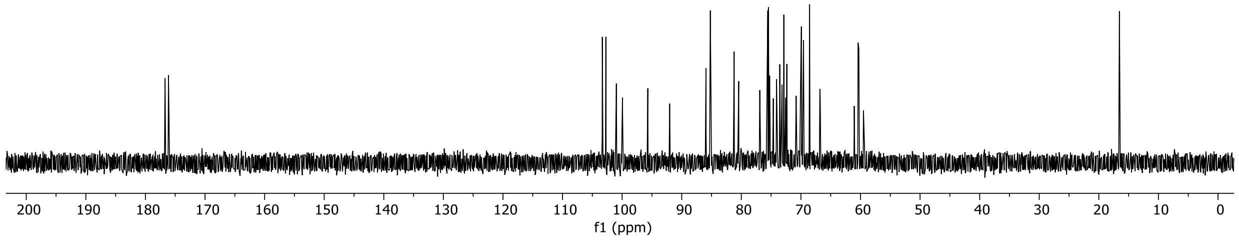

COSY NMR of 5mer-III-di-CO_2^- (D_2O)

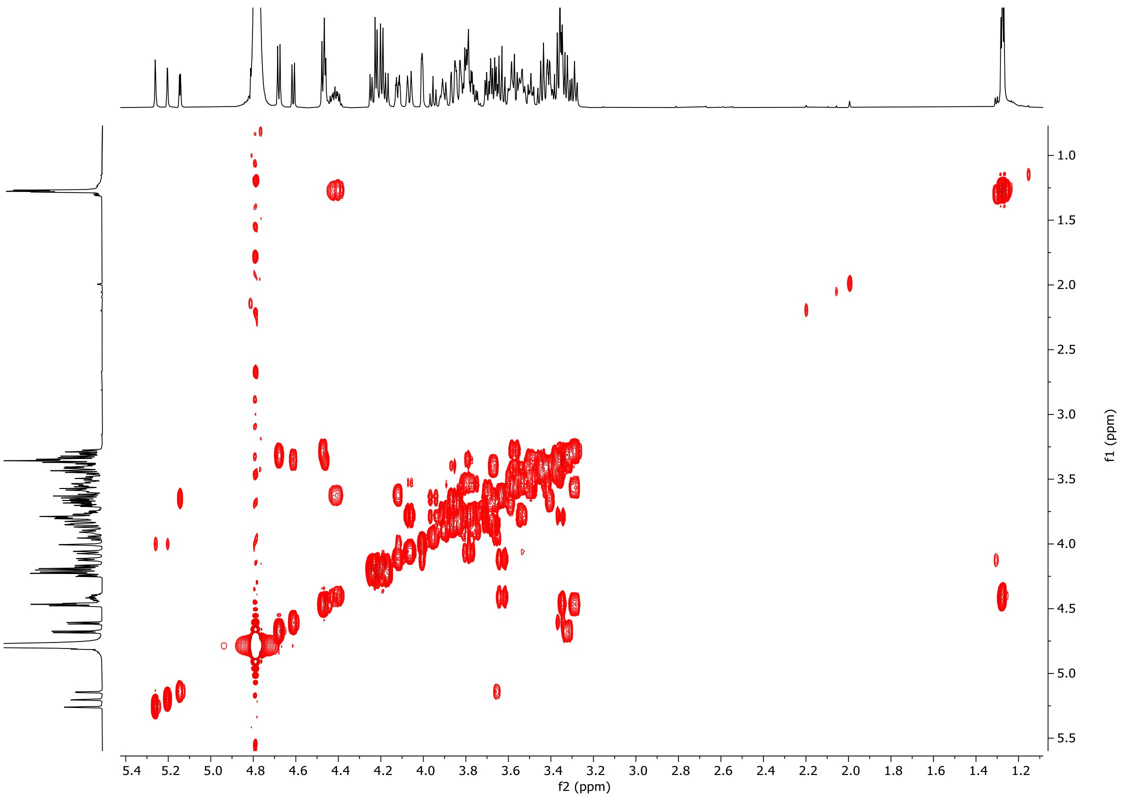

HSQC NMR of 5mer-III-di-CO_2^- (D_2O)

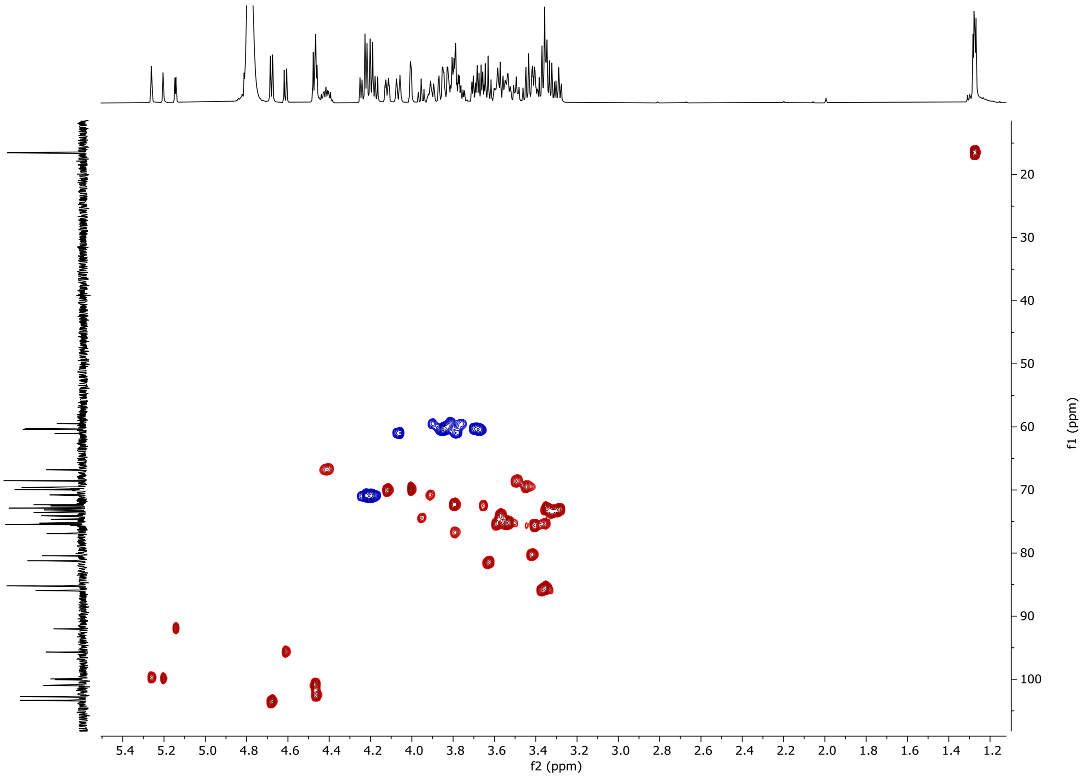

3.5.5 5mer-III-di- NH_3^+

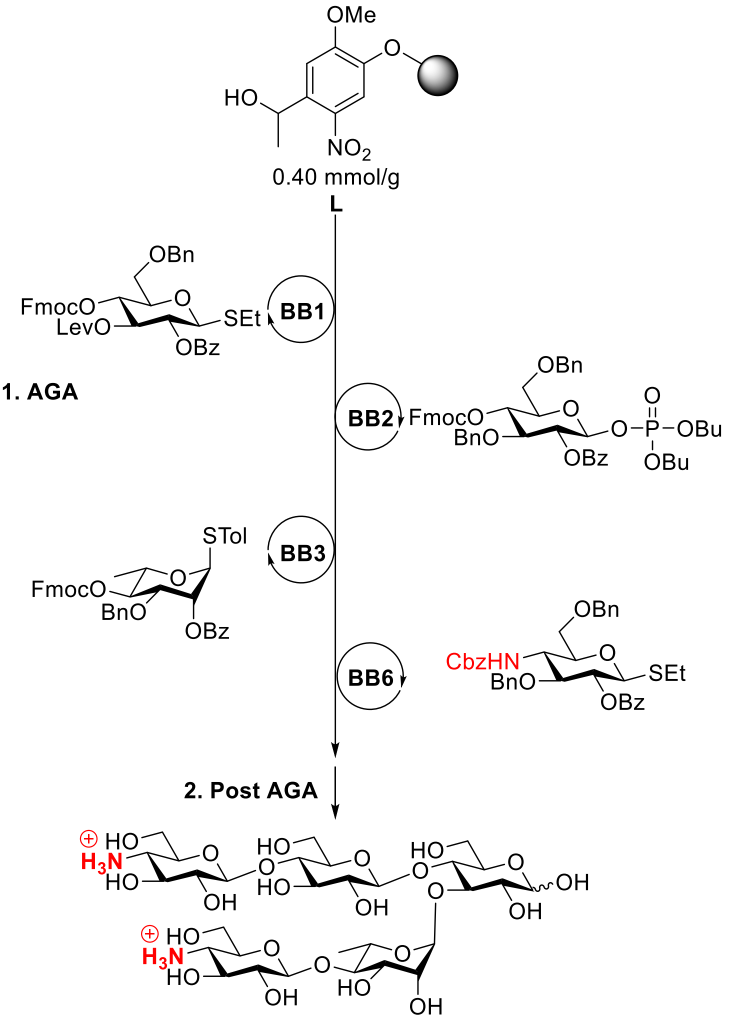

5mer-III-di-NH_3^+

Step		Modules	Notes
		**A**	**L** swelling
**AGA**	**BB1**	**B**, **C1**, **D**, **E1**	**C1**: (**BB1**, -20 °C for 5 min, 0 °C for 20 min)
	**BB2**	**B**, **C2***, **D**, **E2**	**C2***: (**BB2**, -30 °C for 5 min, -10 °C for 40 min) *Double cycle
	**BB3**	**B**, **C1**, **D**, **E1**	**C1**: (**BB3**, -20 °C for 5 min, 0 °C for 20 min)
	**BB6**	**B**, 2x**C1***, **D**, **E1**	**C1***: (**BB6**, -20 °C for 5 min, 0 °C for 20 min) *Double cycle
**Post-AGA**	**Methanolysis**	**H2**	(12 h)
	**Photocleavage**	**I1**	
	**Hydrogenolysis**	**J2**	(3 h)
	**Purification**	**K**	(Method B2: t_R = 17.2 min)

Automated synthesis, global deprotection, and purification afforded **5mer-III-di-NH$_3^+$** as a white solid (1.3 mg, 13 % overall yield).

^{1}H NMR (700 MHz, D_2O) δ 5.26 (d, *J* = 1.8 Hz, 0.6H, H-1β Rha), 5.21 (d, *J* = 1.7 Hz, 0.4H, H-1α Rha), 5.17 (d, *J* = 3.8 Hz, 0.4H, H-1α Glc), 4.69 (d, *J* = 8.0 Hz, 1H, H-1 Glc), 4.64 (d, *J* = 8.0 Hz, 0.6H, H-1β Glc), 4.54 – 4.48 (m, 1H, H-1 Glc), 4.48 – 4.40 (m, 2H, 1×H-1 Glc, 1×H-5 Rha), 4.09 (ddd, *J* = 9.5, 3.4, 1.7 Hz, 1H), 4.06 – 4.04 (m, 1H), 4.02 (t, *J* = 2.5 Hz, 1H), 3.95 (t, *J* = 9.9 Hz, 0.4H), 3.92 (t, *J* = 2.4 Hz, 1H), 3.88 (dt, *J* = 12.4, 2.7 Hz, 2H), 3.85 – 3.73 (m, 6H), 3.68 – 3.54 (m, H), 3.51 (t, *J* = 9.8 Hz, 1H), 3.46 (ddd, *J* = 10.1, 8.6, 1.4 Hz, 1H), 3.36 (dd, *J* = 9.0, 8.0 Hz, 0.6H), 3.34 – 3.28 (m, 3H), 2.99 (dt, *J* = 21.5, 9.9 Hz, 2H), 1.29 (dd, *J* = 6.3, 3.7 Hz, 3H, CH_3-6 Rha).
^{13}C NMR (176 MHz, D_2O) δ 103.52 (C-1 Glc), 102.71 (C-1 Glc), 101.07 (C-1 Glc), 100.23 (C-1α Rha), 100.10 (C-1β Rha), 95.74 (C-1β Glc), 92.02 (C-1α Glc), 81.28, 79.83, 77.47, 75.29, 75.17, 74.48, 74.12, 73.48, 73.13, 72.56, 72.52, 72.48, 70.80, 70.13, 70.08, 69.99, 66.92, 66.86, 60.90, 60.71, 60.67, 59.52, 52.54, 52.23, 16.63 (C-6 Rha).
ESI-HRMS m/z 811.3195 $[M+H]^+$ ($C_{30}H_{55}N_2O_{23}$ requires 811.3196).

RP-HPLC of 5mer-III-di-NH$_3^+$ (ELSD trace, Method B1, t_R = 17.7, 18.8 min)

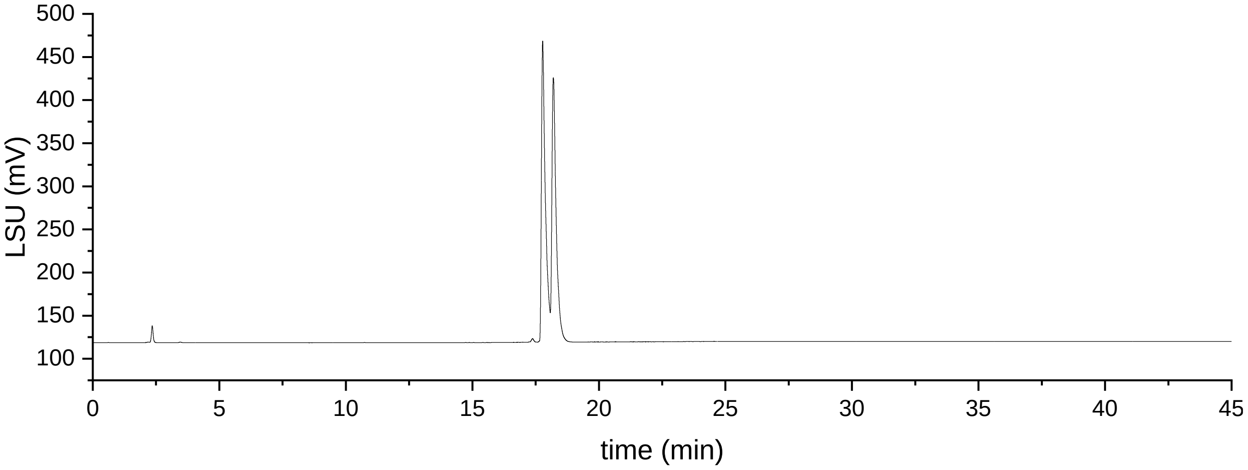

^{1}H NMR of 5mer-III-di-NH_3^+ (700 MHz, D_2O)

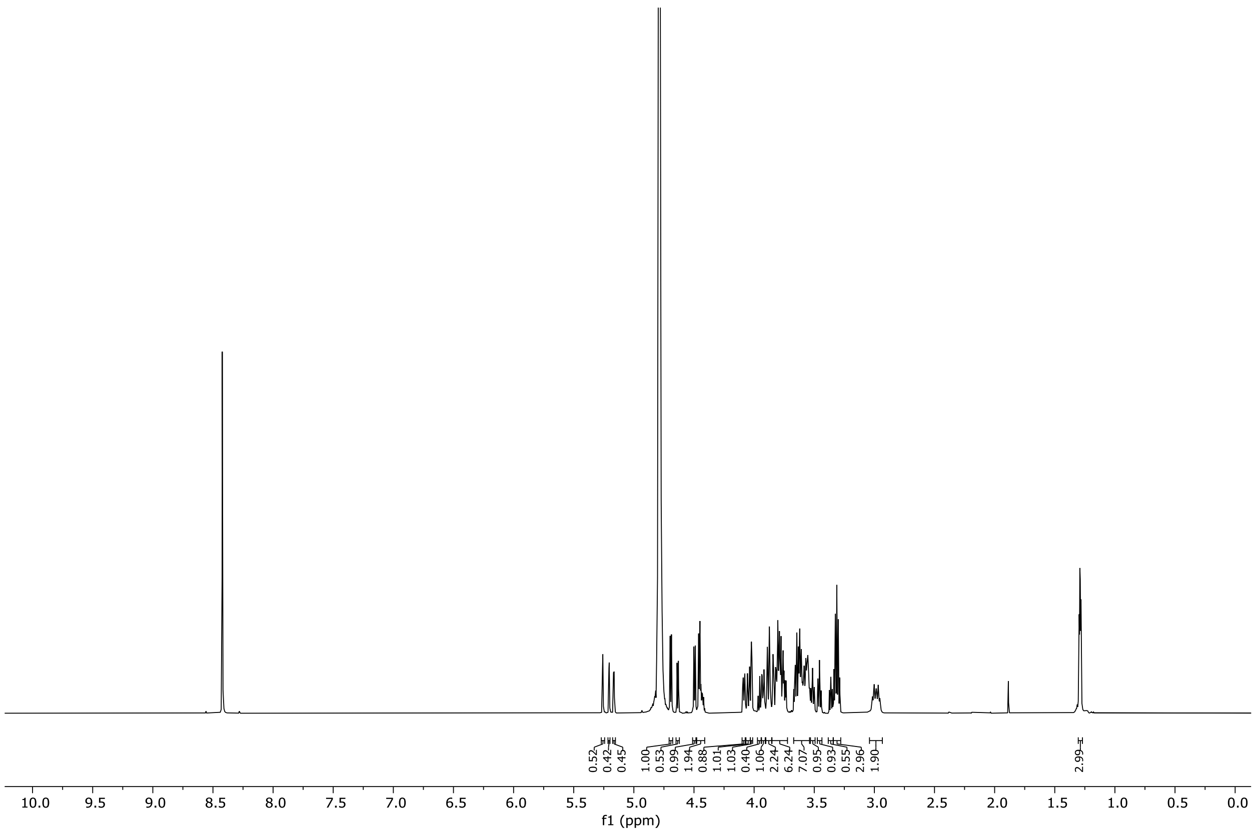

^{13}C NMR of 5mer-III-di-NH_3^+ (176 MHz, D_2O)

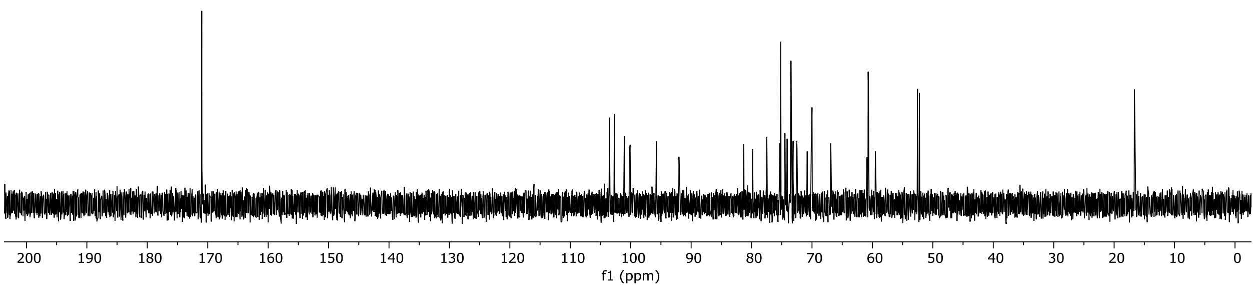

COSY NMR of 5mer-III-di-NH_3^+ (D_2O)

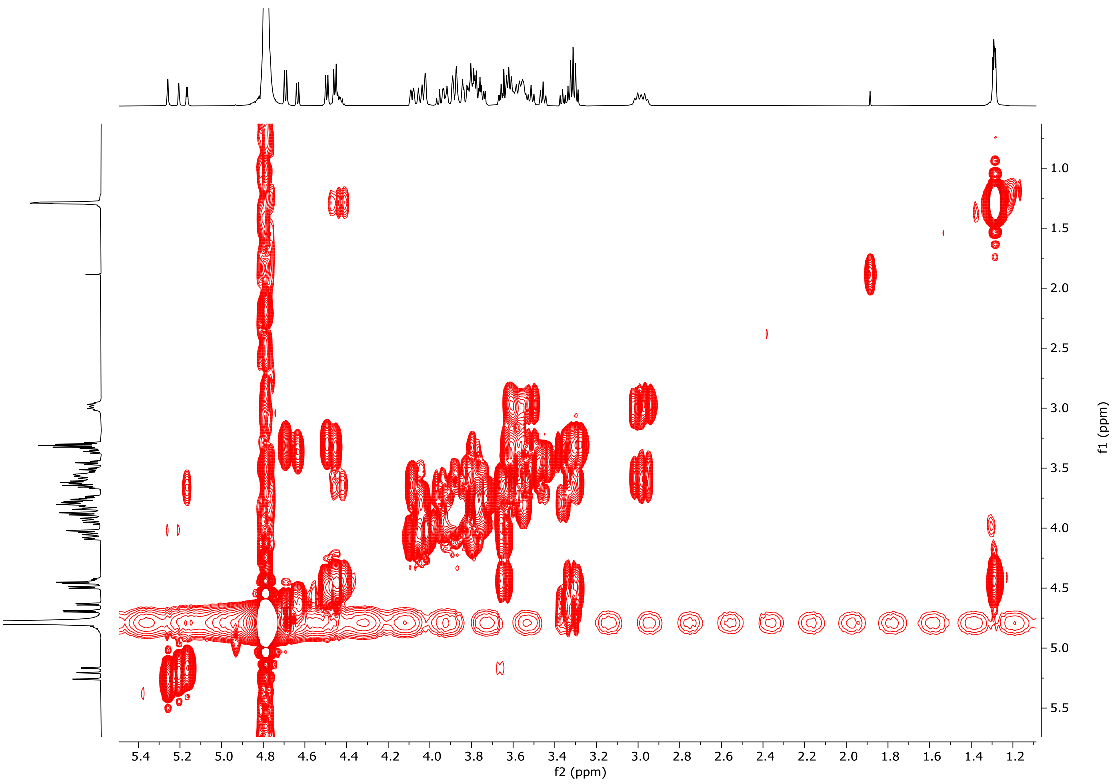

HSQC NMR of 5mer-III-di-NH_3^+ (D_2O)

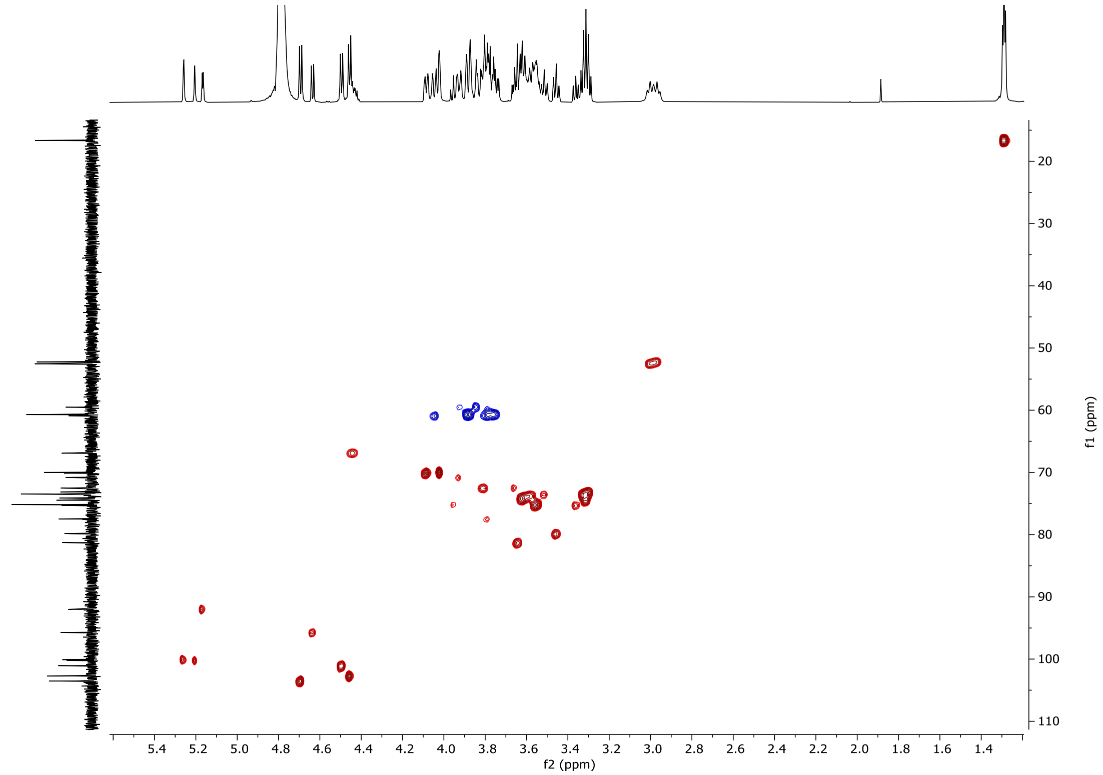

4 NMR studies

4.1 NMR characterization of 5mer-III-zwi

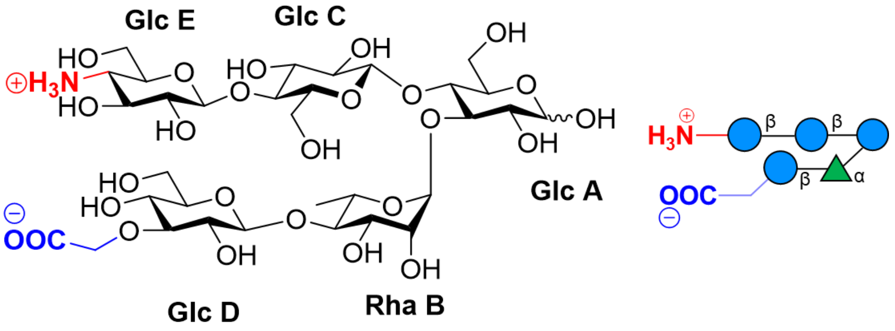

5mer-III-zwi

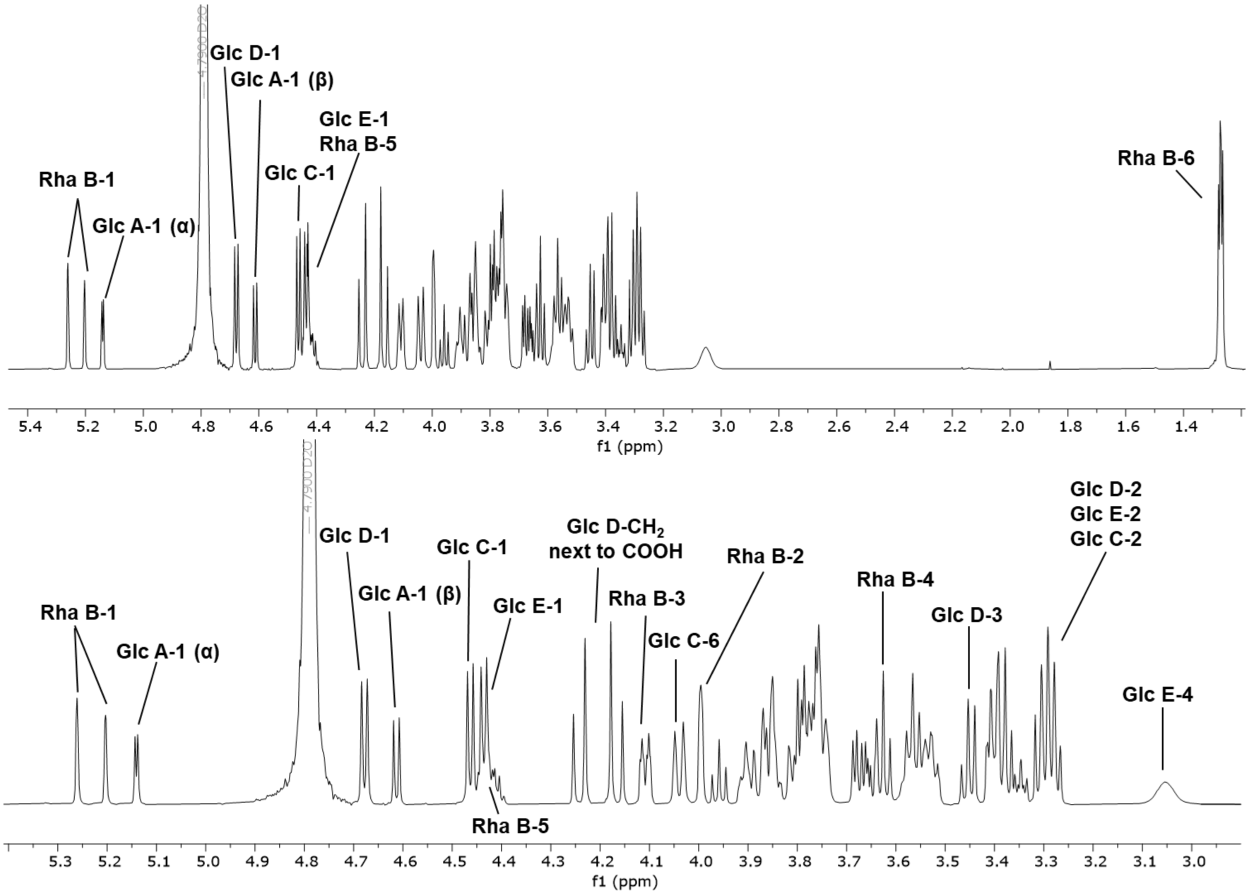

Figure S04

^{1}H NMR (700 MHz, D_2O) of **5mer-III-zwi** with assignments.

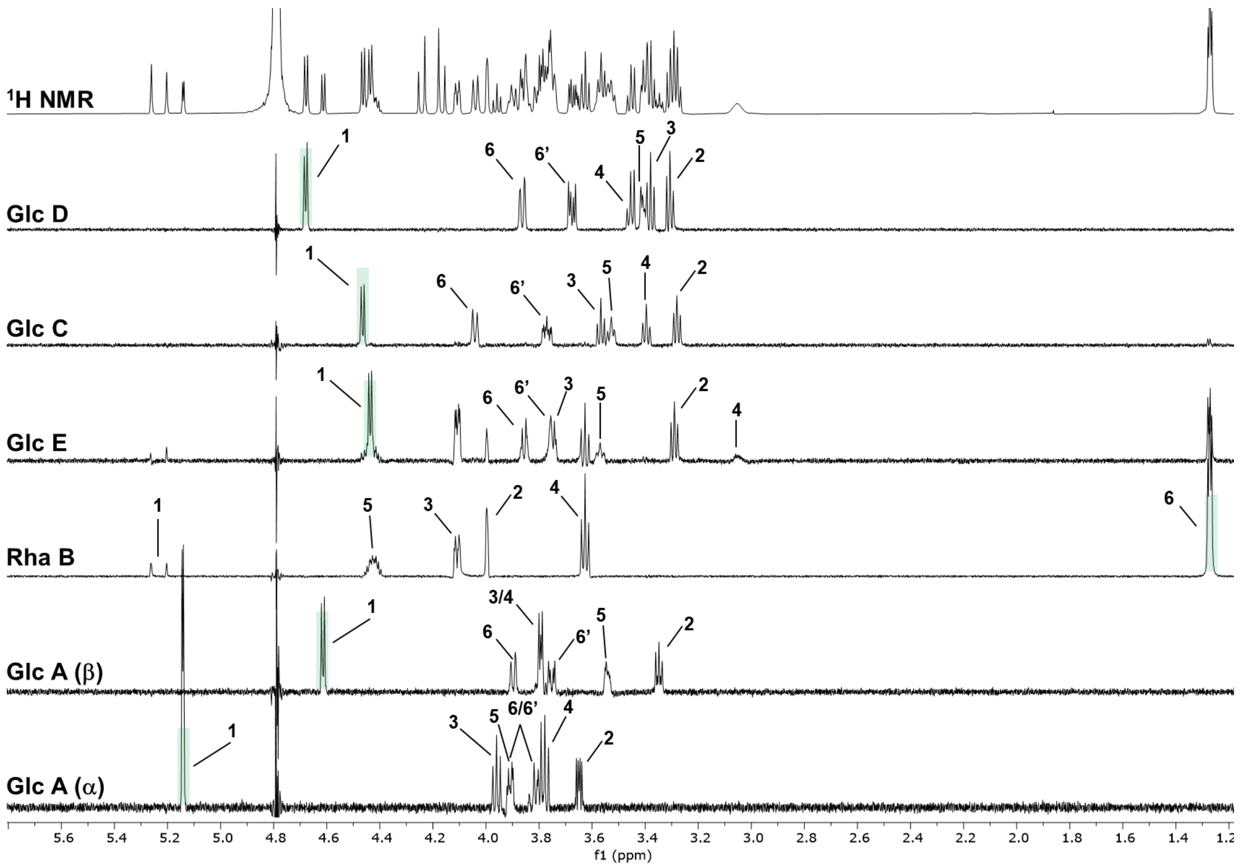

Figure S05
1D TOCSY (700 MHz, d9 200 ms, D_2O) of **5mer-III-zwi** with assignments. Resonances chosen for selective excitation are highlighted with green boxes.

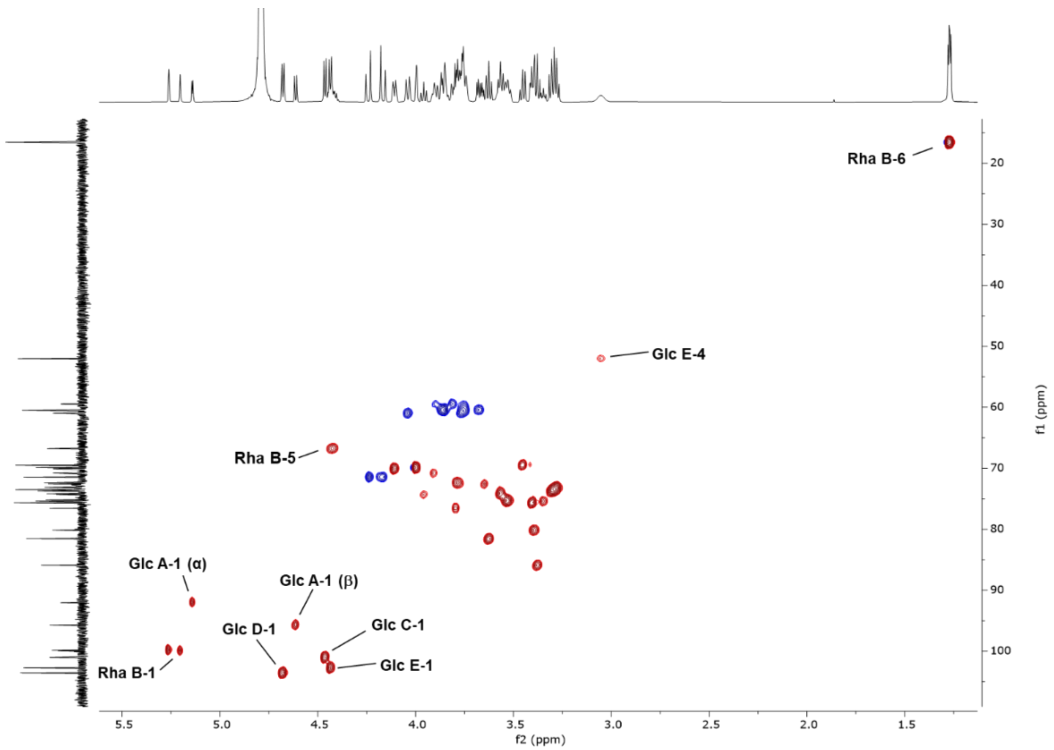

Figure S06
HSQC NMR (D_2O) of **5mer-III-zwi** with assignments.

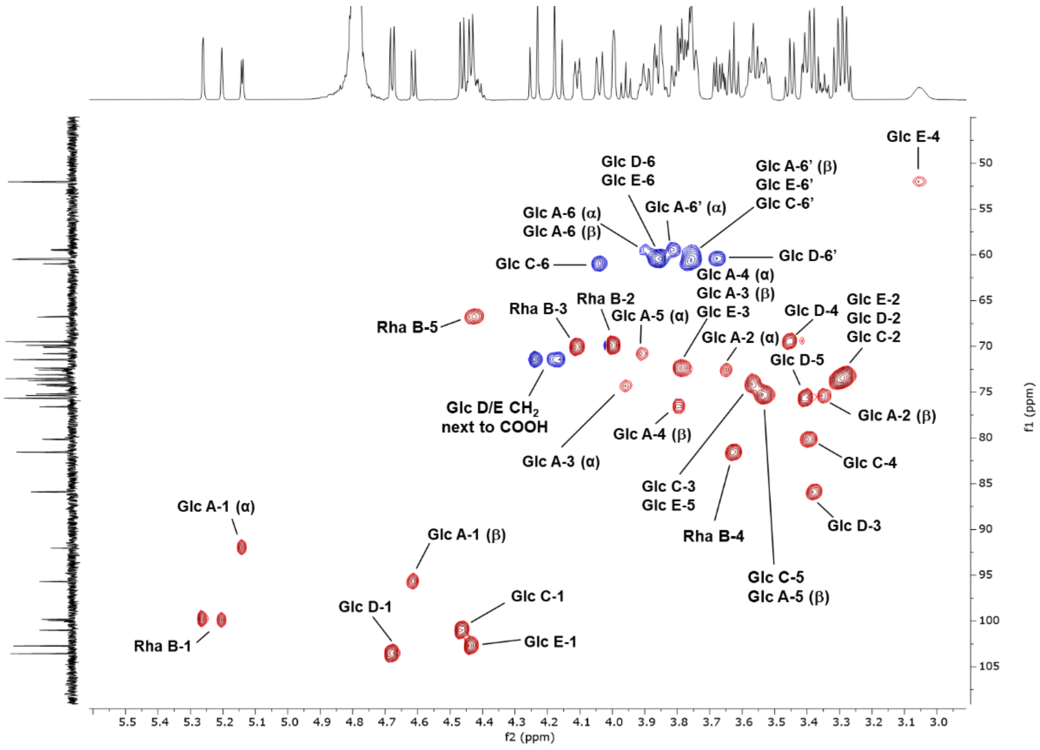

Figure S07
Excerpt of HSQC NMR (D_2O) of **5mer-III-zwi** with assignments.

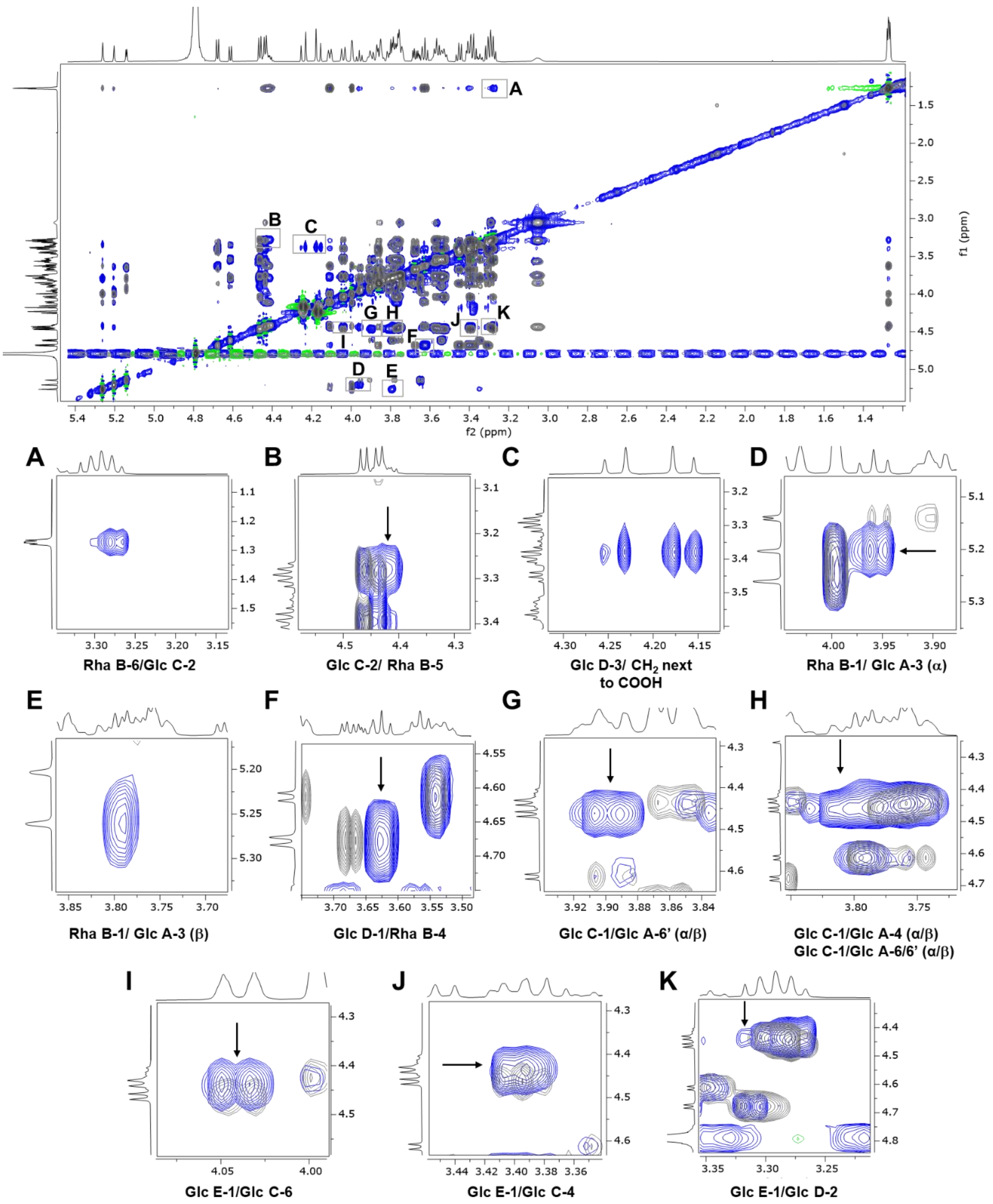

Figure S08

Superimposed 2D NOESY (green-blue, 700 MHz, d8 800 ms, D_2O) of **5mer-III-zwi** at pH 7.07 with assignments and 2D TOCSY spectrum (gray, 700 MHz, d9 150 ms, D_2O).

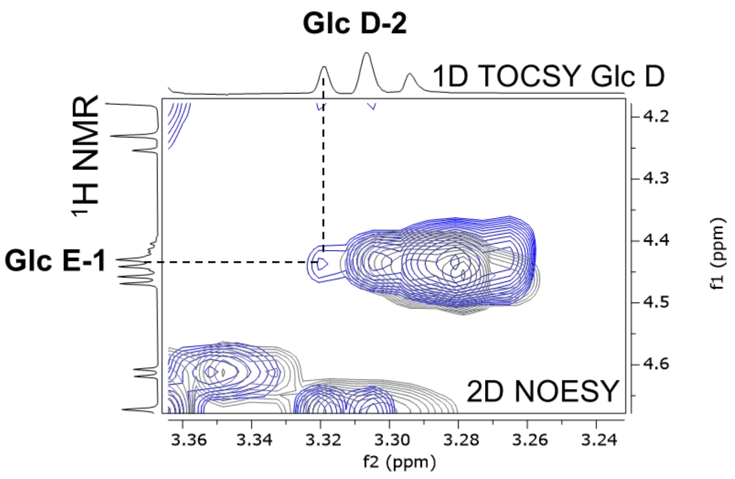

Figure S09
Excerpt of 2D ROESY (green-blue) and 2D TOCSY (gray) of **5mer-III-zwi** superimposed. The horizontal trace shows the 1D TOCSY with selective excitation of Glc D-1. The key NOE cross peak between Glc E-1 and Glc D-2 is marked with dashed lines.

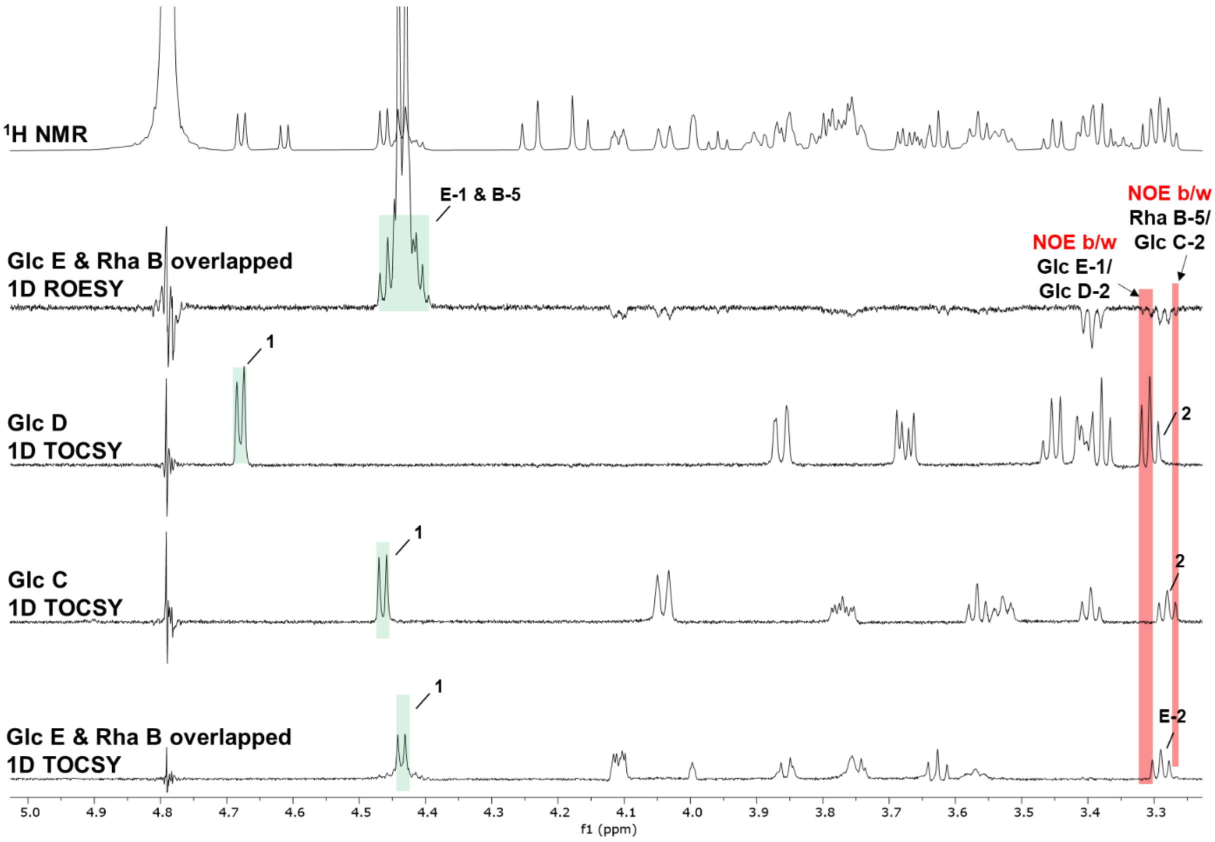

Figure S10
Overlay of 1D ROESY (700 MHz, p15 300 ms, D_2O) and 1D TOCSY (700 MHz, d9 200 ms, D_2O) of **5mer-III-zwi**. Key NOE signal between Glc E-1/Glc D-2 and Rha B-5/Glc C-2 (highlighted with red box) was observed.

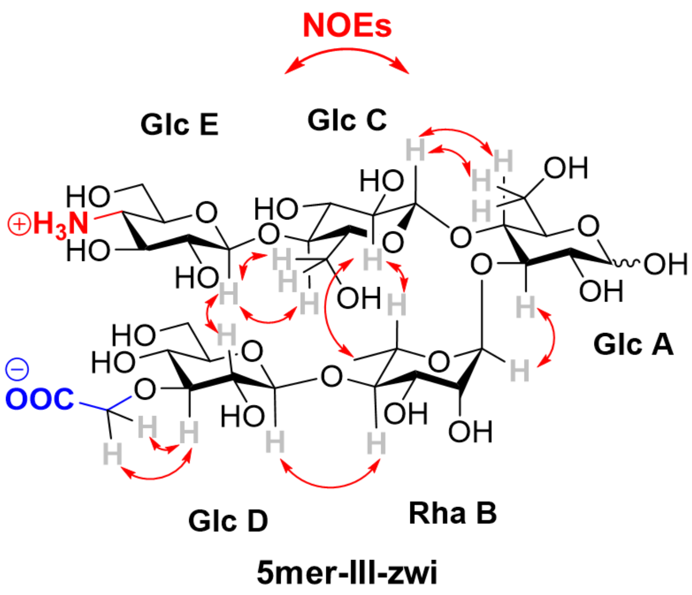

Figure S11
All experimentally observed NOEs (red arrows) of **5mer-III-zwi** at pH 7.07.

4.1.1 pH titration of 5mer-III-zwi

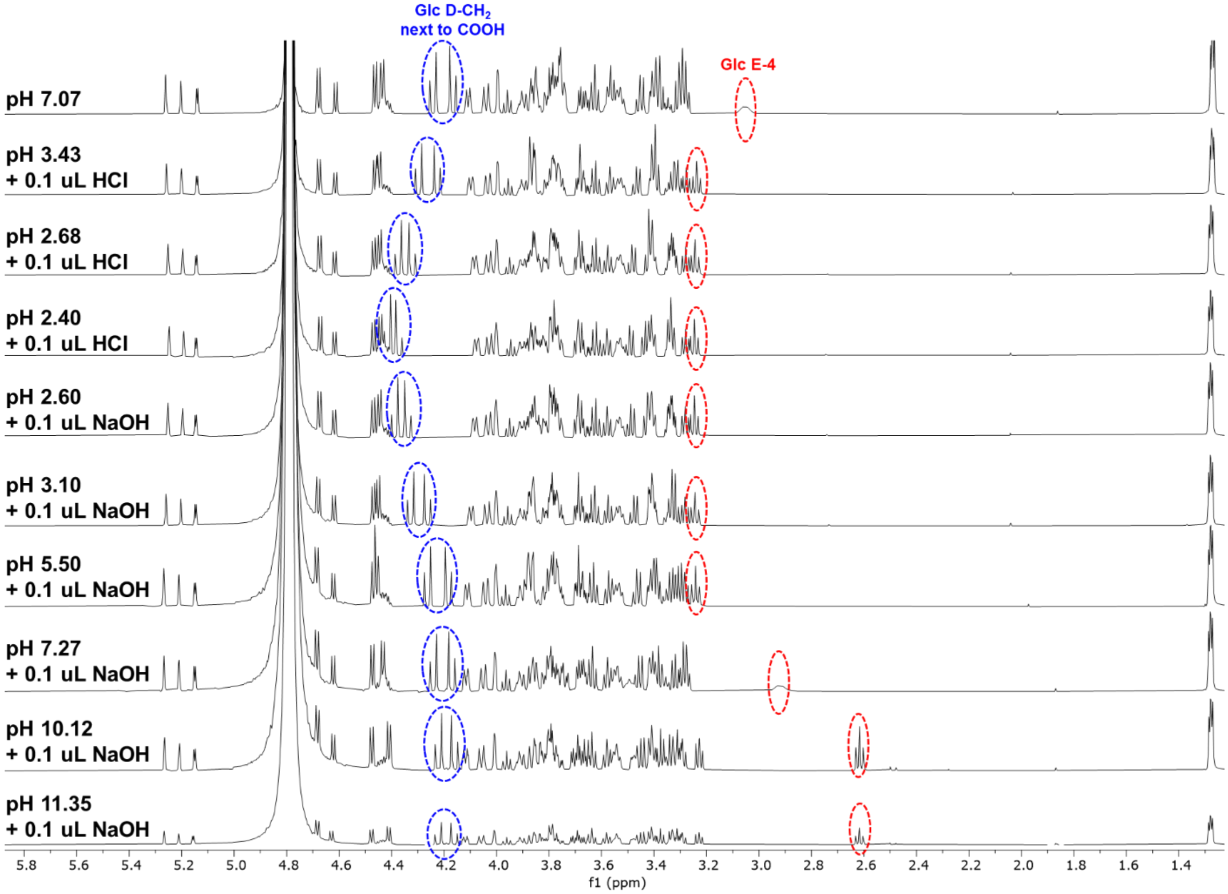

Figure S12

^{1}H NMR (700 MHz, D_2O) titration of **5mer-III-zwi**. The pH of the solution was adjusted using 1 M HCl and 1 M NaOH solutions. Changes in protonation state of the carboxylic acids and amino group can be followed by monitoring the chemical shift of Glc D-CH_2 next to COOH (blue) Glc E-4 (red). The shift of selected signals during the titration is highlighted with: blue circle (Glc D-CH_2 next to COOH) and red circle (Glc E-4).

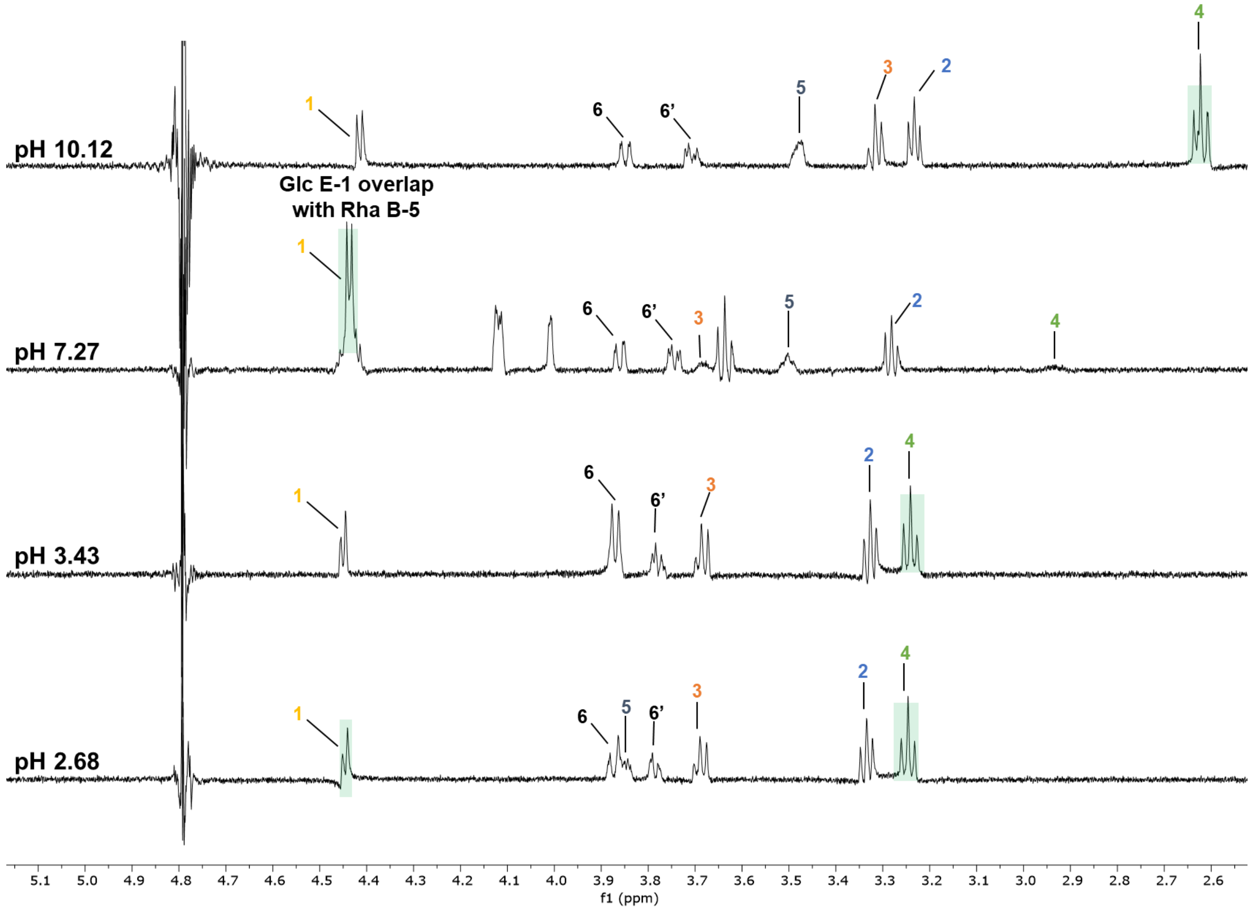

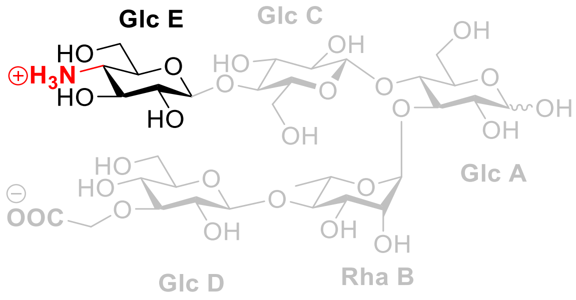

Figure S13
Selective 1D TOCSY (700 MHz, d9 200 ms, D2O) spectra of Glc E of **5mer-III-zwi** with assignments showing the peak shifts at different pH. Resonances chosen for selective excitation are highlighted with green boxes.

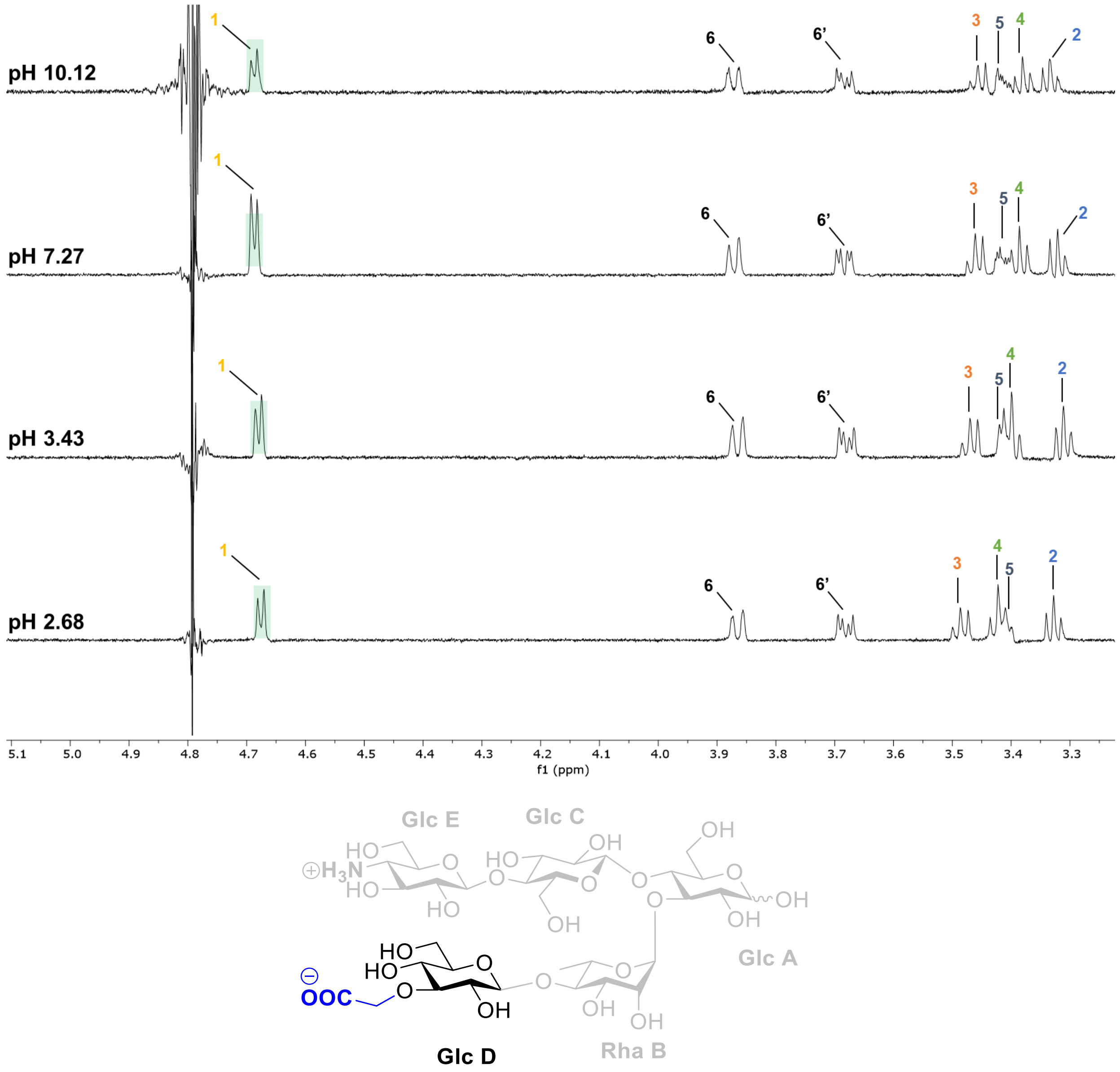

Figure S14
Selective 1D TOCSY (700 MHz, d9 200 ms, D_2O) spectra of **Glc D** of **5mer-III-zwi** with assignments showing the peak shifts at different pH. Resonances chosen for selective excitation are highlighted with green boxes.

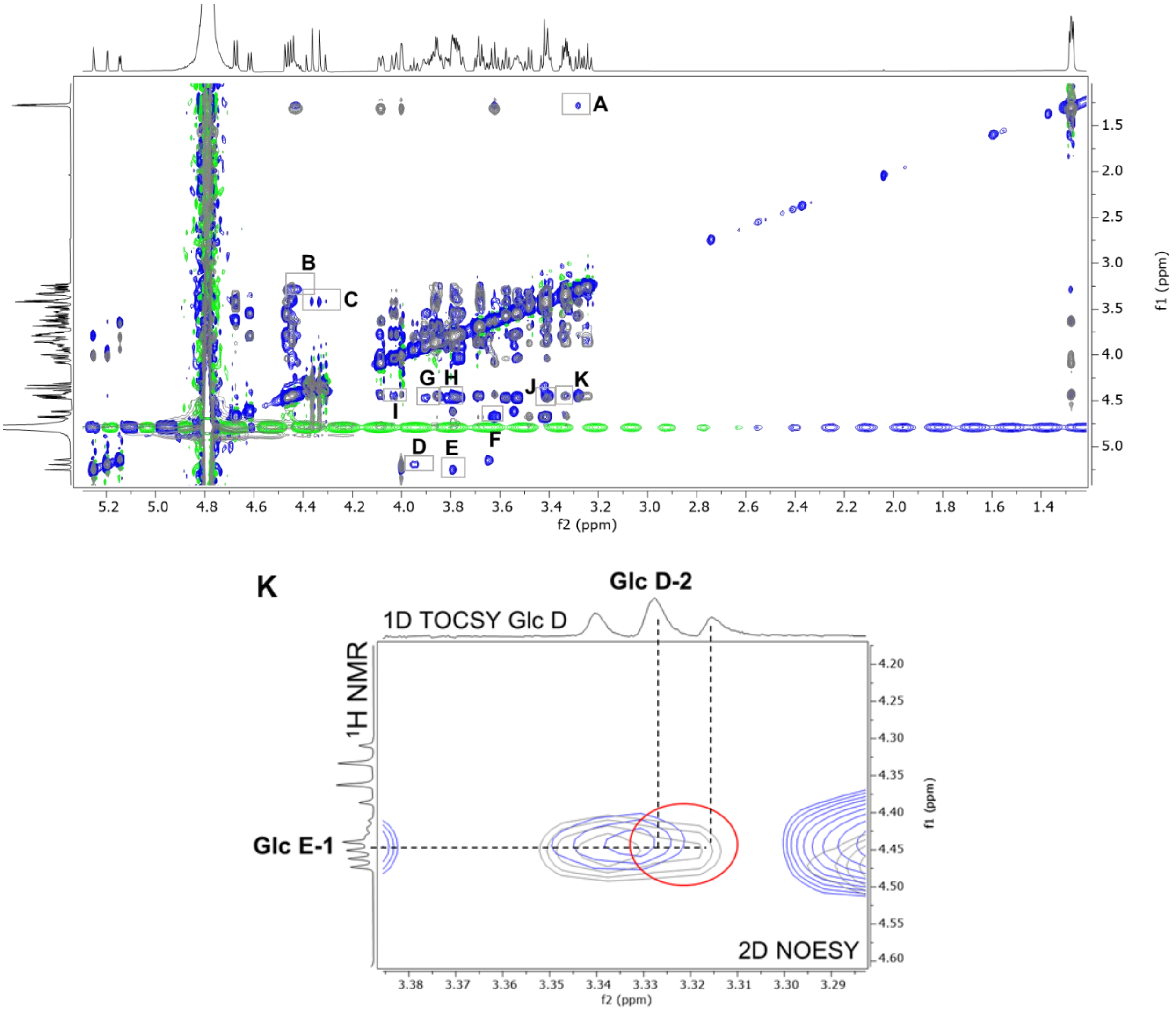

Figure S15

Superimposed 2D NOESY (green-blue, 700 MHz, d8 800 ms, D_2O) of **5mer-III-zwi** at **pH 2.68** with assignments and 2D TOCSY spectrum (gray, 700 MHz, d9 150 ms, D_2O). The pH was adjusted from **3.43** to **2.68** by adding 0.1 uL of 1M HCl solution. At pH 2.68, all the NOEs observed at **pH 7.07** were detected (**Figure S08**) except for the inter-strand NOE signal between the two strands Glc E-1/Glc D-2 (**K**).

.

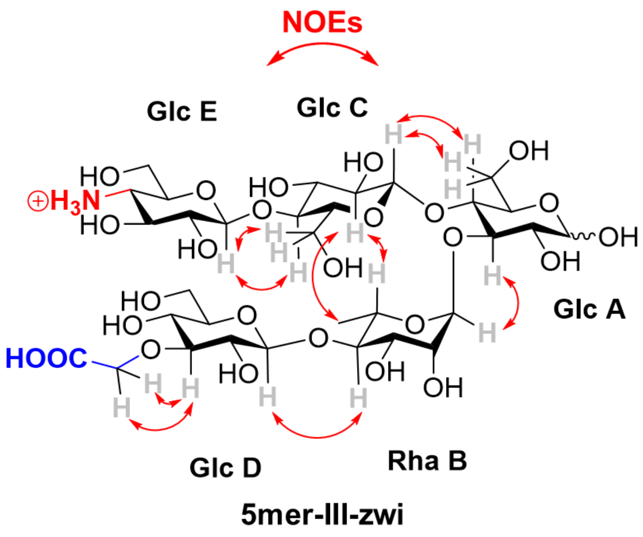

Figure S16
All experimentally observed NOEs (red arrows) **5mer-III-zwi** at **pH 2.68**.

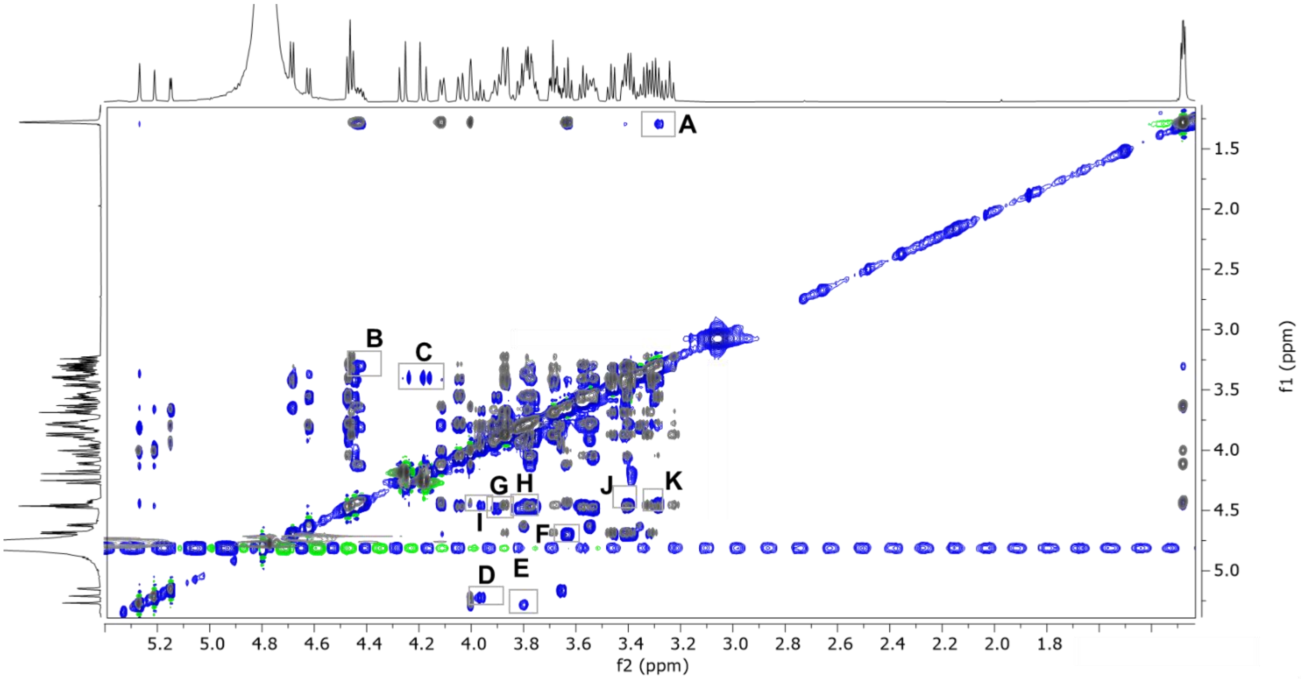

Figure S17
Superimposed 2D NOESY (green-blue, 700 MHz, d8 800 ms, D_2O) of **5mer-III-zwi** at **pH 5.5** with assignments and 2D TOCSY spectrum (gray, 700 MHz, d9 150 ms, D_2O). The pH was adjusted from **2.6** to **5.5** by adding 0.2 uL of a 1 M NaOH solution. At **pH 5.5**, all the NOEs observed at **pH 7.07** were detected (**Figure S08**).

•

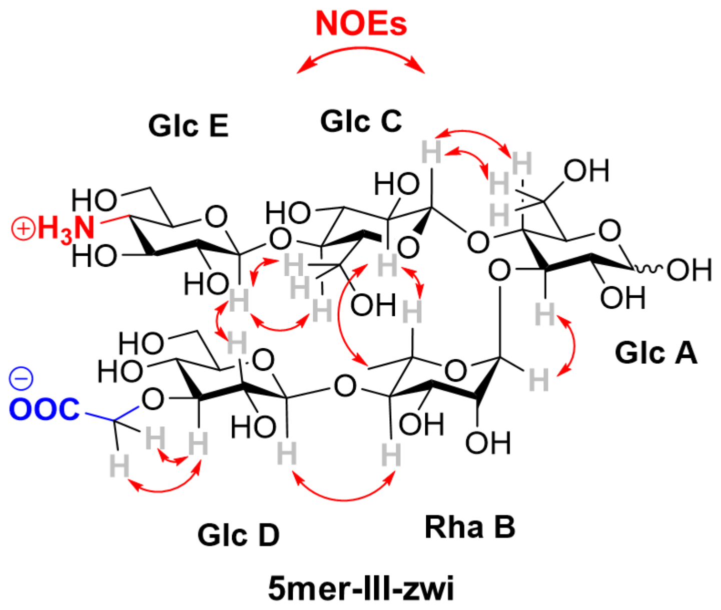

Figure S18
All experimentally observed NOEs (red arrows) of **5mer-III-zwi** at **pH 5.5**.

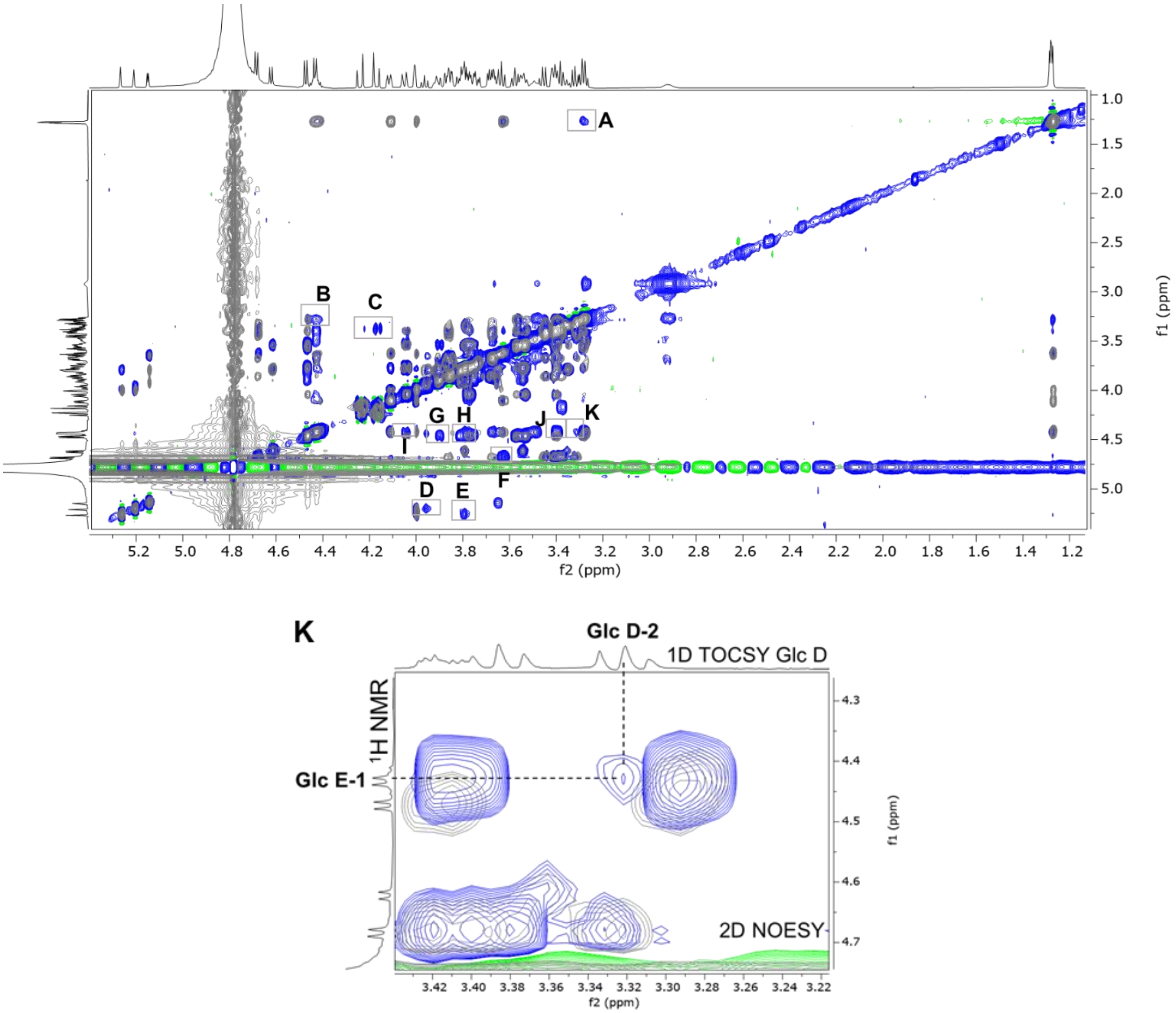

Figure S19

Superimposed 2D NOESY (green-blue, 700 MHz, d8 800 ms, D_2O) of **5mer-III-zwi** at **pH 7.27** with assignments and 2D TOCSY spectrum (gray, 700 MHz, d9 150 ms, D_2O). The pH was adjusted from **2.40** to **7.27** by adding 0.4 uL of a 1 M NaOH solution. At **pH 7.27**, all the NOEs observed at **pH 7.07** were detected (**Figure S08**) except for the inter-strand NOE signal between the two strands Glc E-1/Glc D-2 (**K**).

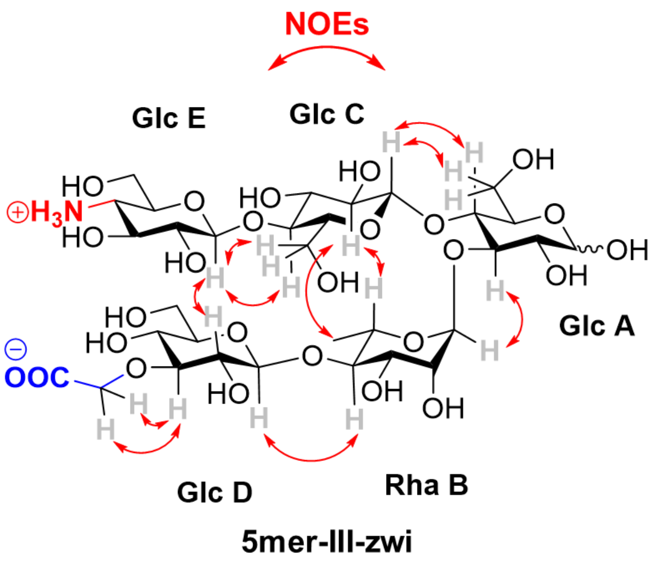

Figure S20
All experimentally observed NOEs (red arrows) **5mer-III-zwi** at **pH 7.27**.

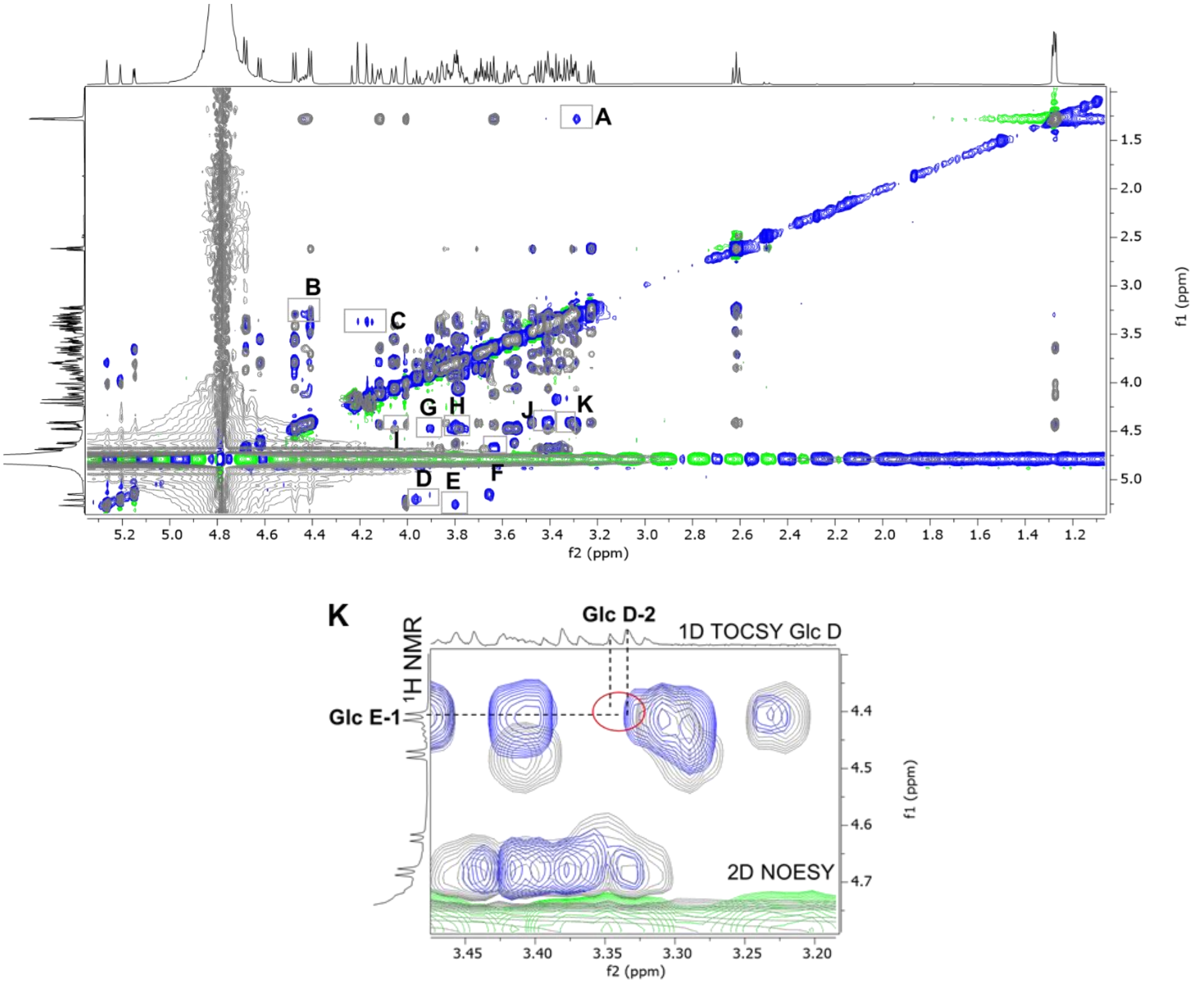

Figure S21

Superimposed 2D NOESY (green-blue, 700 MHz, d8 800 ms, D_2O) of **5mer-III-zwi** at **pH 10.12** with assignments and 2D TOCSY spectrum (gray, 700 MHz, d9 150 ms, D_2O). The pH was adjusted from **7.27** to **10.12** by adding 0.1 uL of a 1 M NaOH solution. At **pH 10.12**, all the NOEs observed at **pH 7.07** were detected (**Figure S08**) except for the inter-strand NOE signal between the two strands Glc E-1/Glc D-2 (**K**).

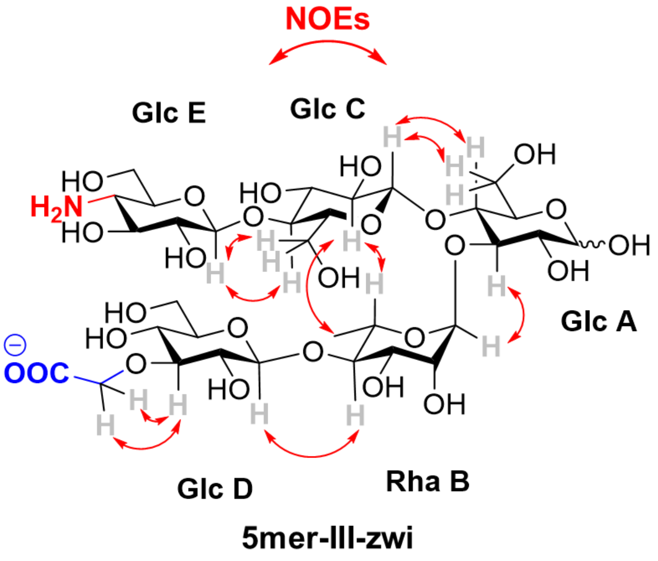

Figure S22
All experimentally observed NOEs (red arrows) **5mer-III-zwi** at **pH 10.12**.

pH	Protonation state	Glc E-1/GlcD-2 NOE signal
**10.12**	COO^- / NH_2	No
**7.27**	COO^- / NH_3^+	Yes
**3.43**	$COO(H)^-$ / NH_3^+	No
**2.68**	COOH / NH_3^+	No

Table S01
Overall analysis of inter-strand NOE signal Glc E-1/Glc D-2 of **5mer-III-zwi** at different pH.

4.2 NMR characterization of 5mer-III-di-SO_3^-

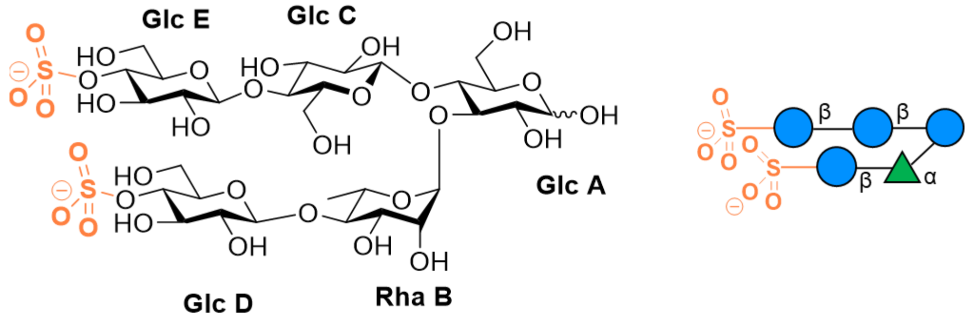

5mer-III-di-SO_3^-

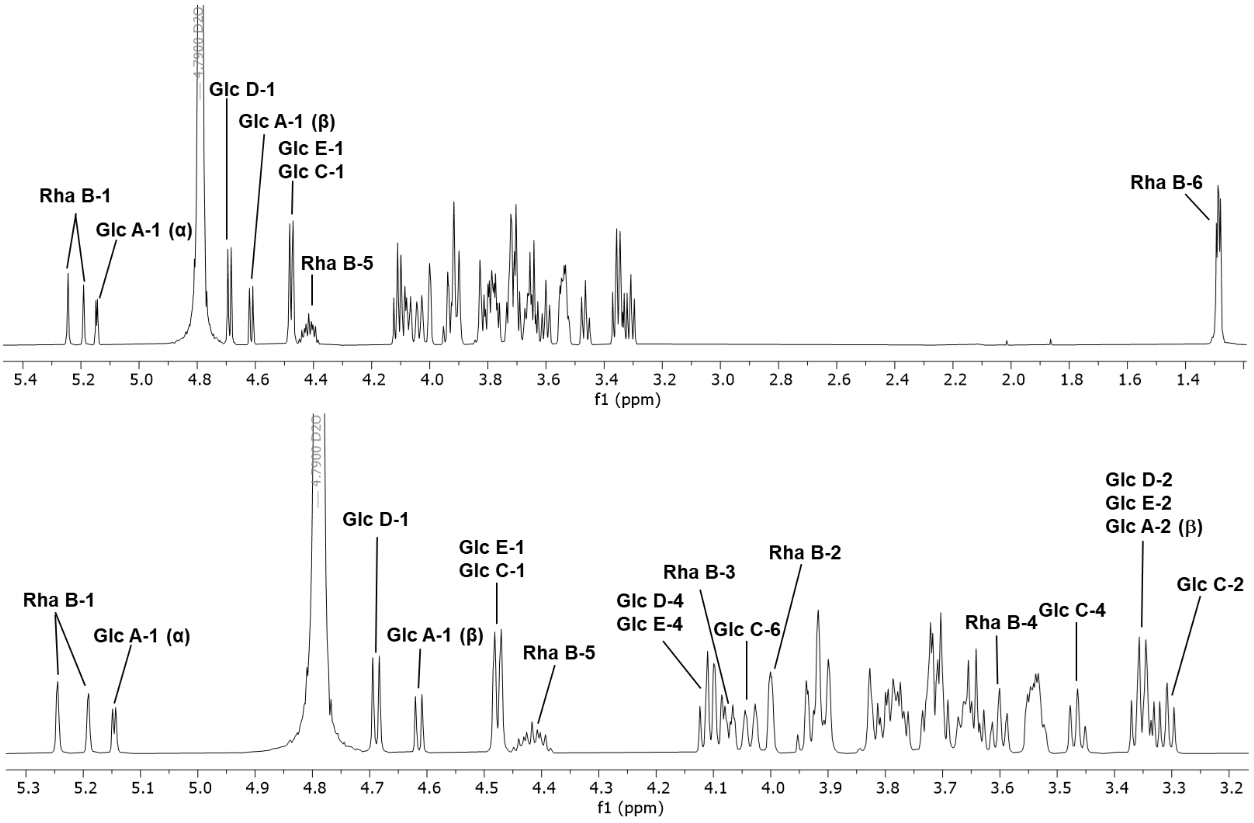

Figure S23
^{1}H NMR (700 MHz, D_2O) of **5mer-III-di-SO_3^-** with assignments.

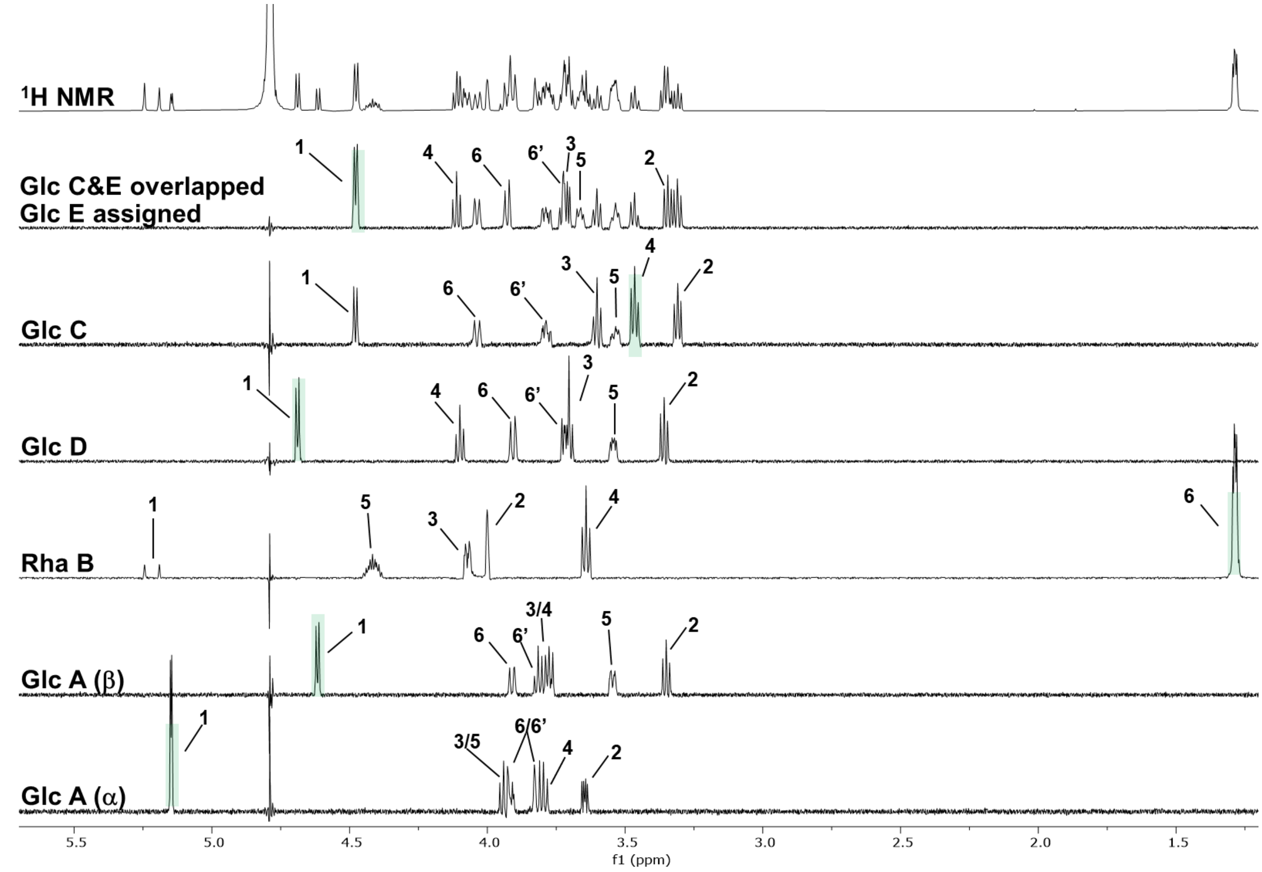

Figure S24
1D TOCSY (700 MHz, d9 200 ms, D_2O) of **5mer-III-di-SO_3^-** with assignments. Resonances chosen for selective excitation are highlighted with green boxes.

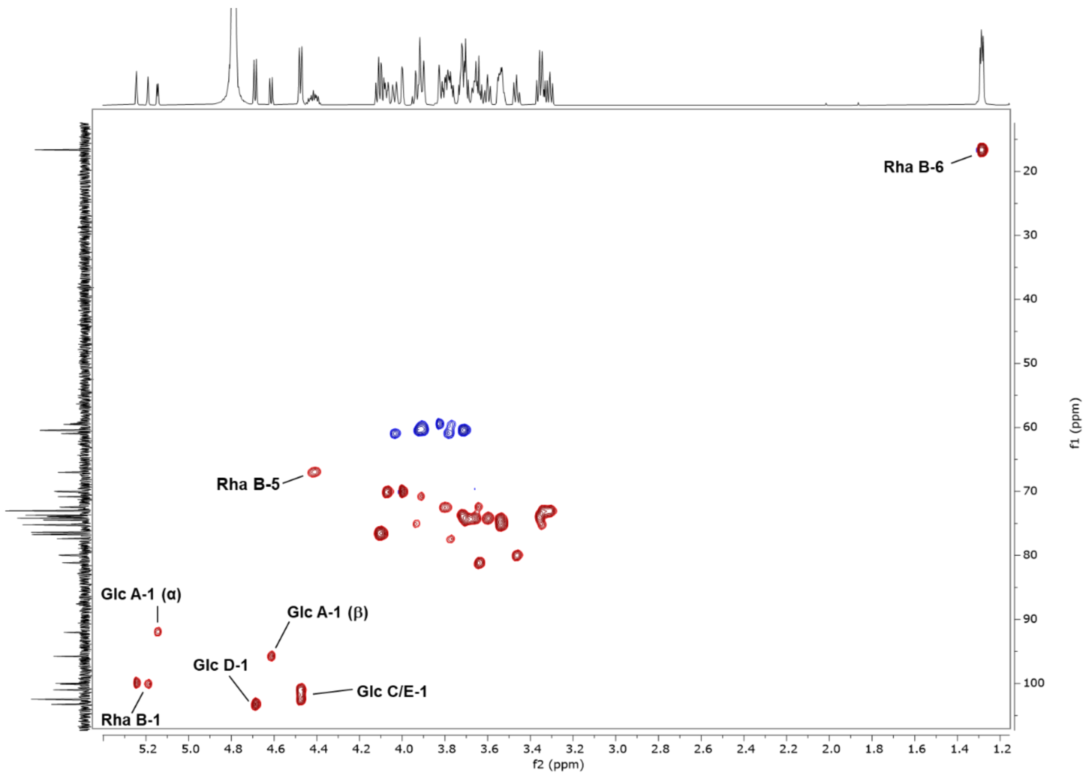

Figure S25
HSQC NMR (D_2O) of **5mer-III-di-SO_3^-** with assignments.

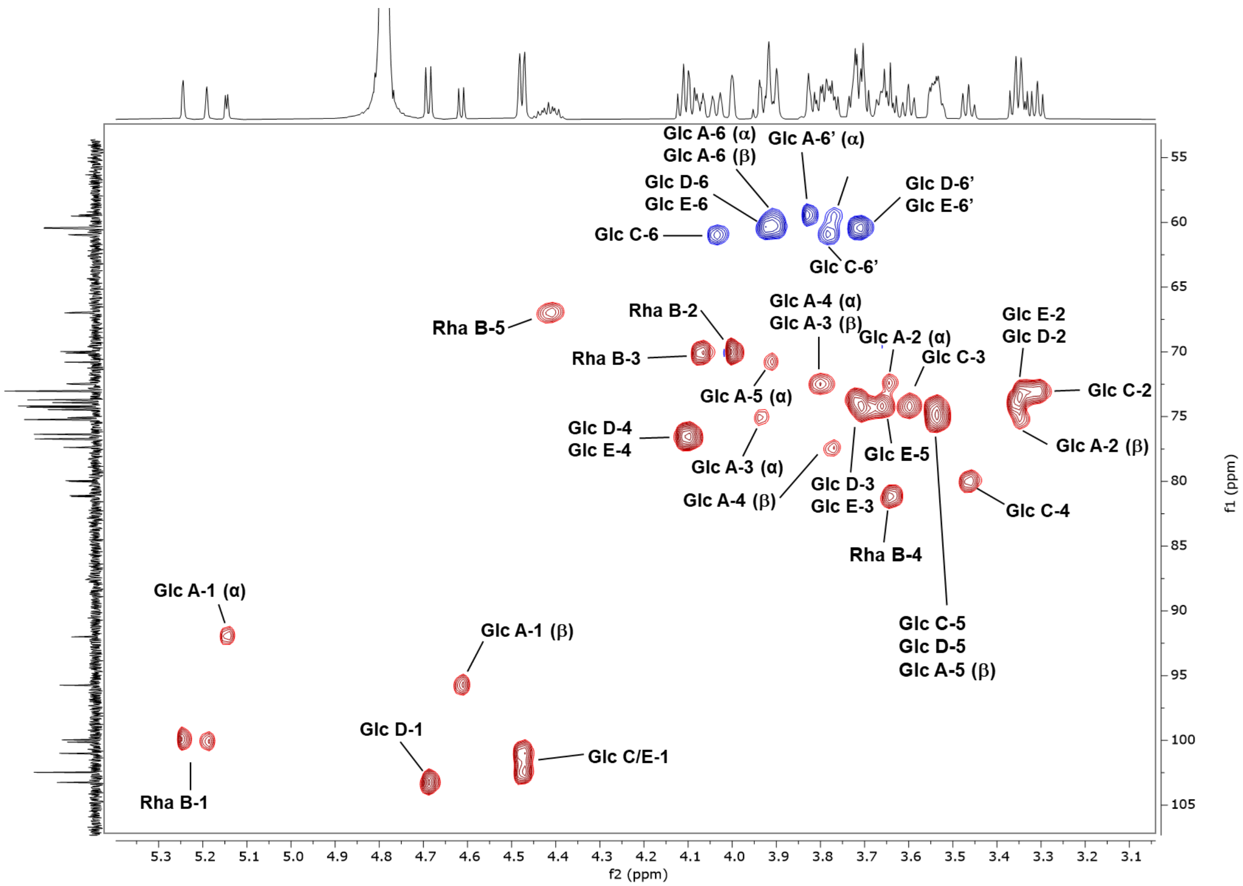

Figure S26

Excerpt of HSQC NMR (D_2O) of **5mer-III-di-SO_3^-** with assignments.

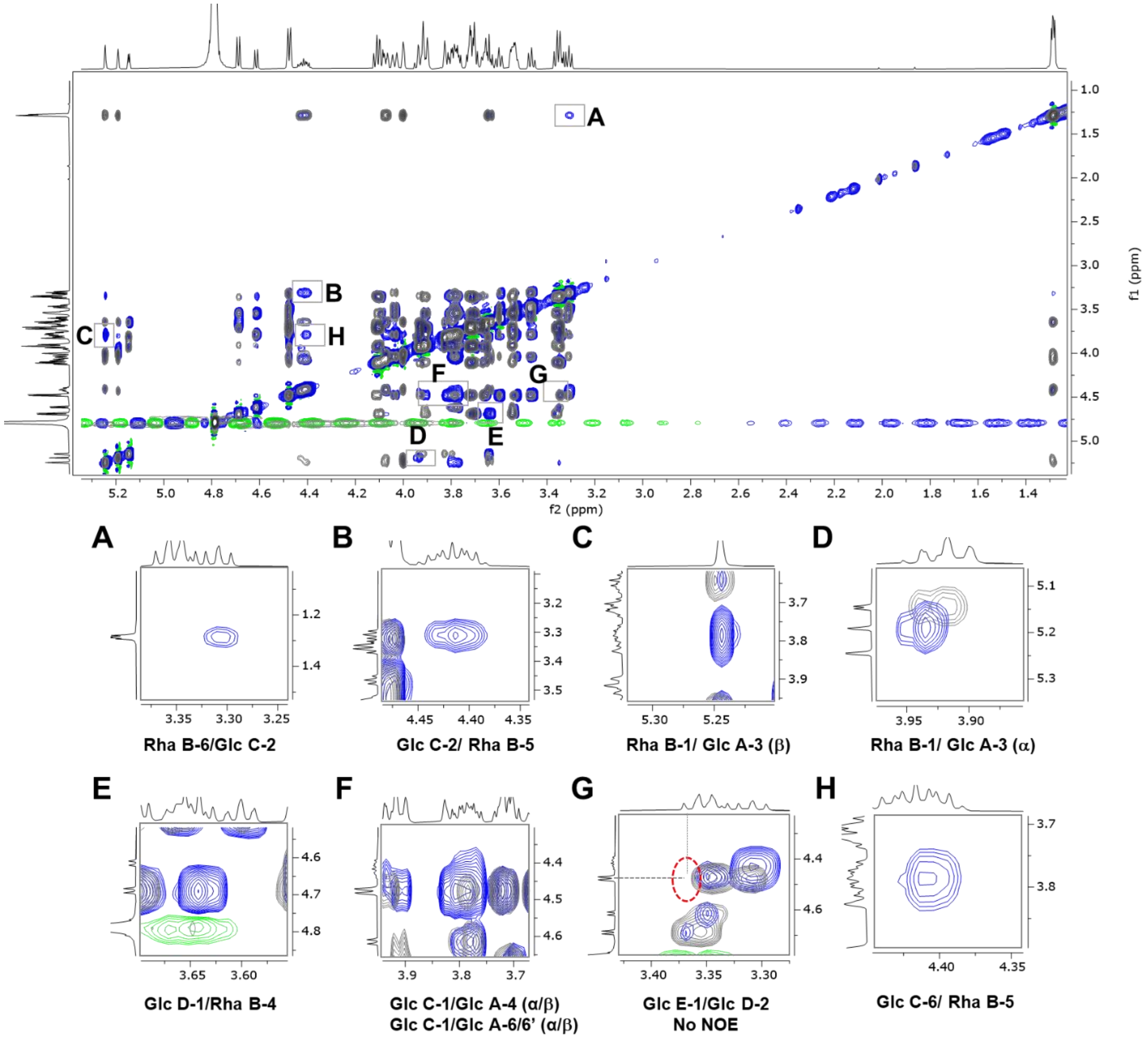

Figure S27
Superimposed 2D NOESY (green-blue, 700 MHz, d8 800 ms, D_2O) of **5mer-III-di-SO$_3^-$** with assignments and 2D TOCSY spectrum (gray, 700 MHz, d9 150 ms, D_2O). In panel G, the red cycle highlights the absence of the NOE signal between Glc E-1 and Glc D-2.

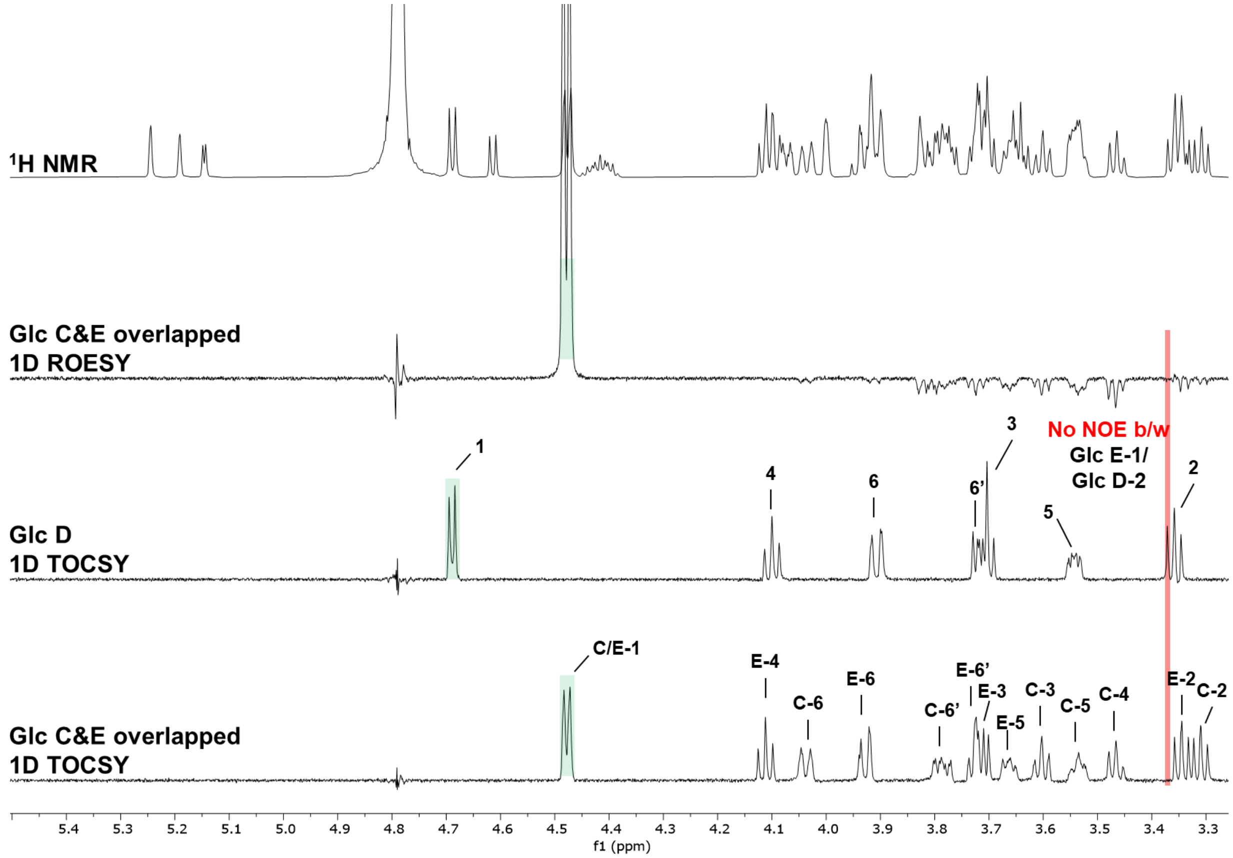

Figure S28
Overlay of 1D ROESY (700 MHz, p15 300 ms, D_2O) and 1D TOCSY (700 MHz, d9 200 ms, D_2O) of **5mer-III-di-SO_3^-**. No NOE signal between Glc E-1/Glc D-2 (highlighted with red box) was observed.

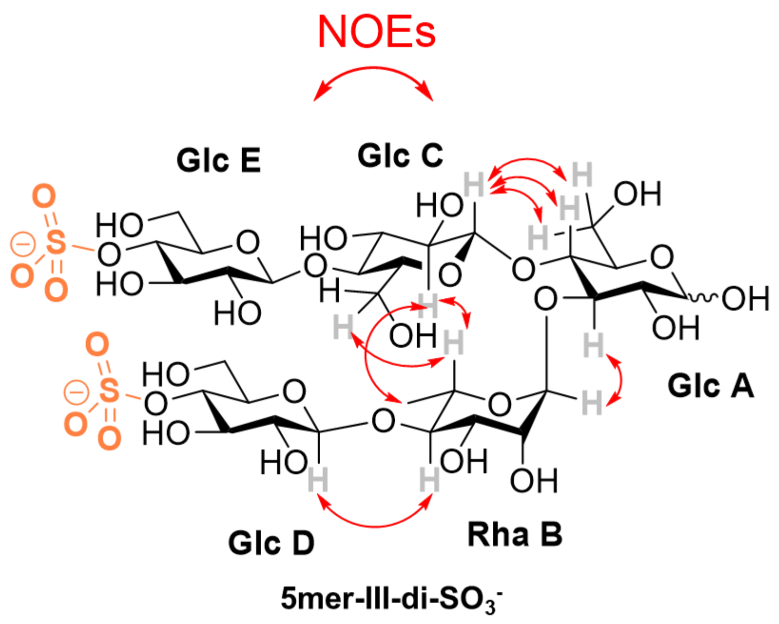

Figure S29
All experimentally observed NOEs (red arrows) of **5mer-III-di-SO$_3^-$**.

4.3 NMR characterization of 5mer-III-di-PO_3^{2-}

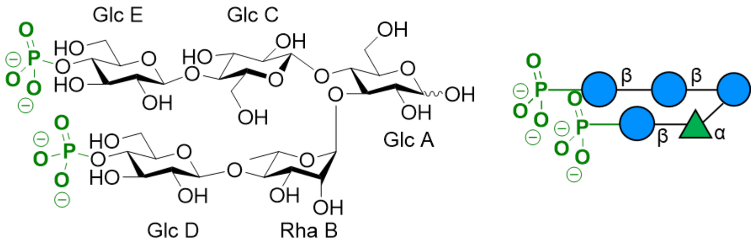

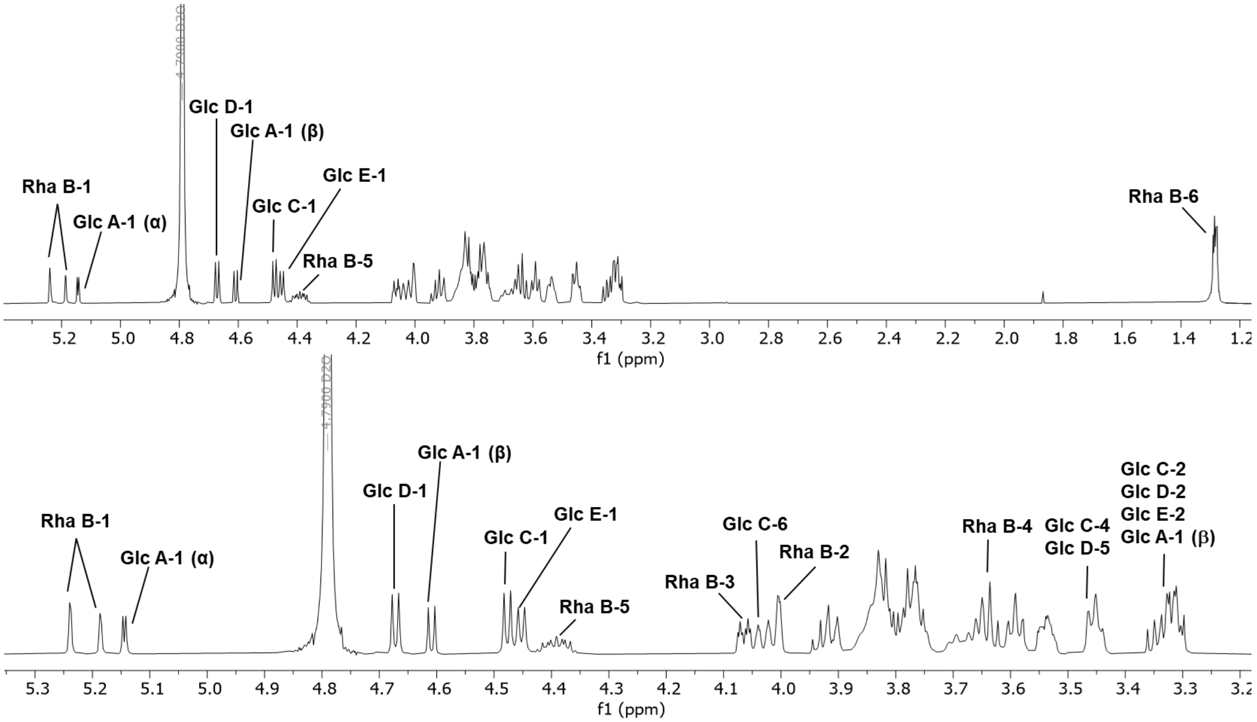

Figure S30
^{1}H NMR (700 MHz, D_2O) of **5mer-III-di-PO_3^{2-}** with assignments.

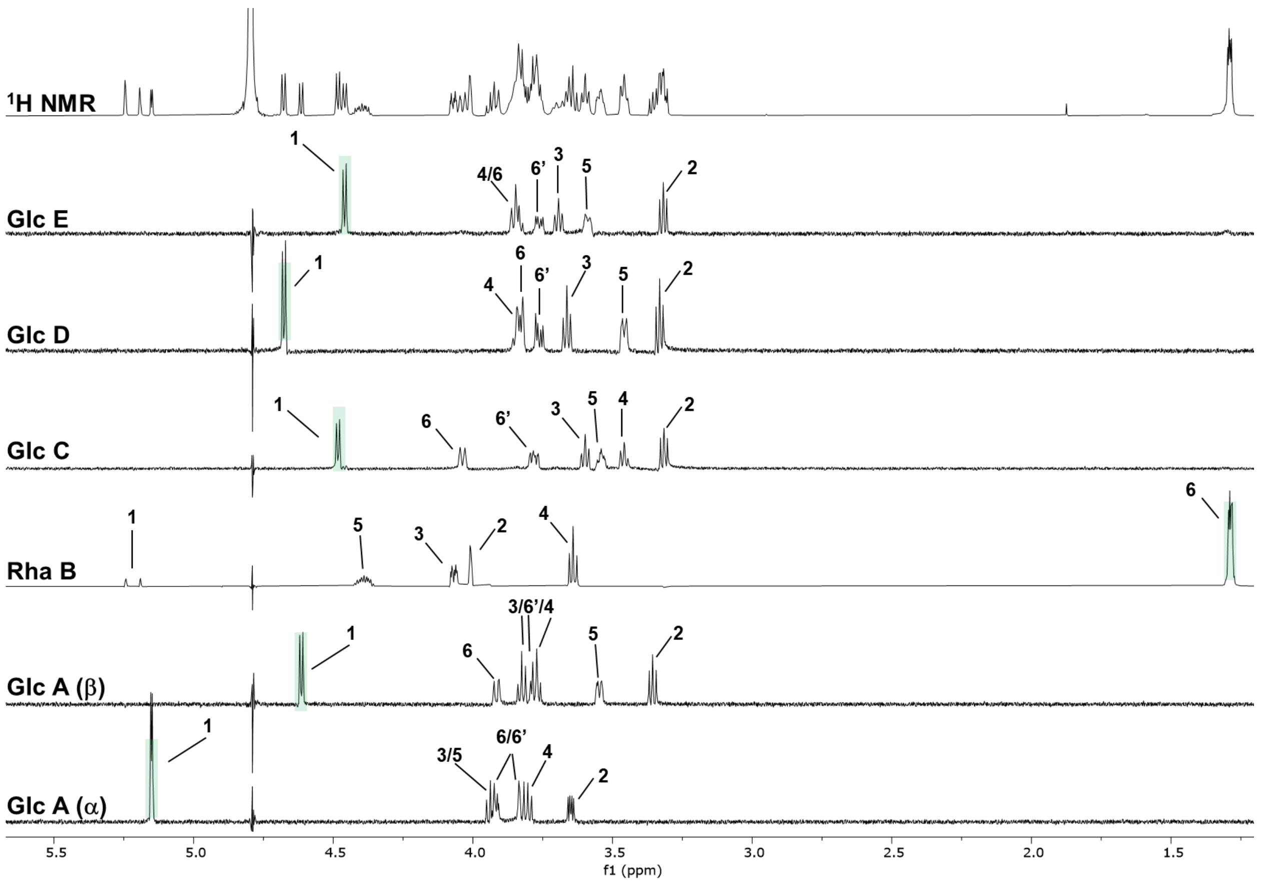

Figure S31
1D TOCSY (700 MHz, d9 200 ms, D_2O) of **5mer-III-di-PO$_3^{2-}$** with assignments. Resonances chosen for selective excitation are highlighted with green boxes.

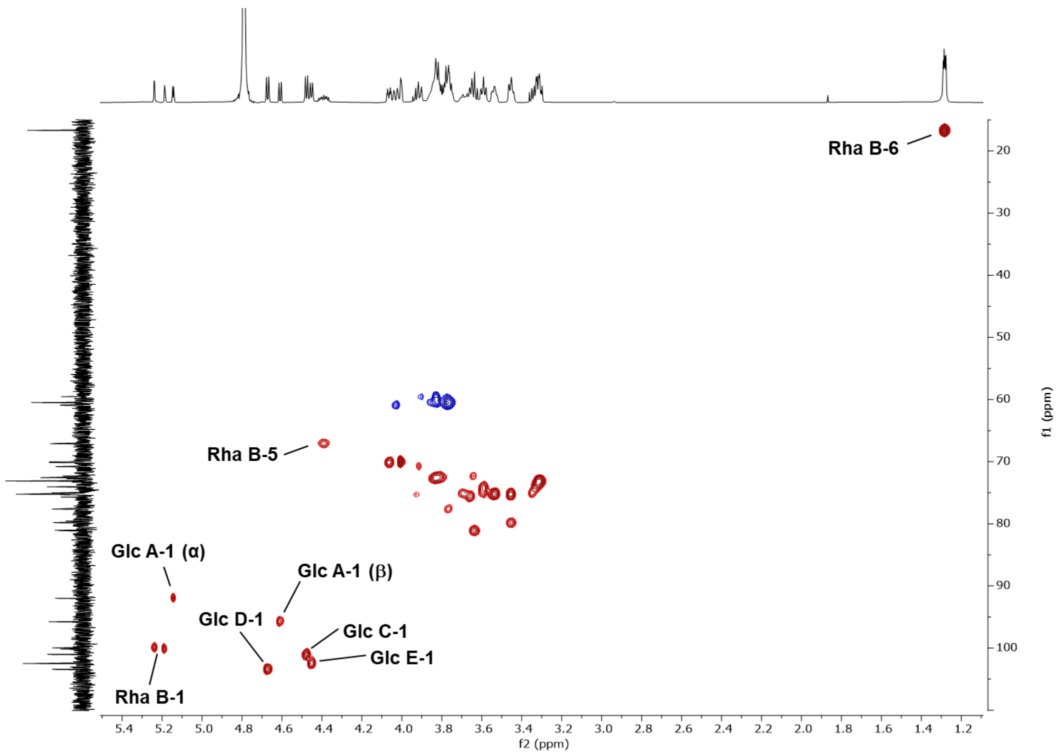

Figure S32
HSQC NMR (D_2O) of **5mer-III-di-PO_3^{2-}** with assignments.

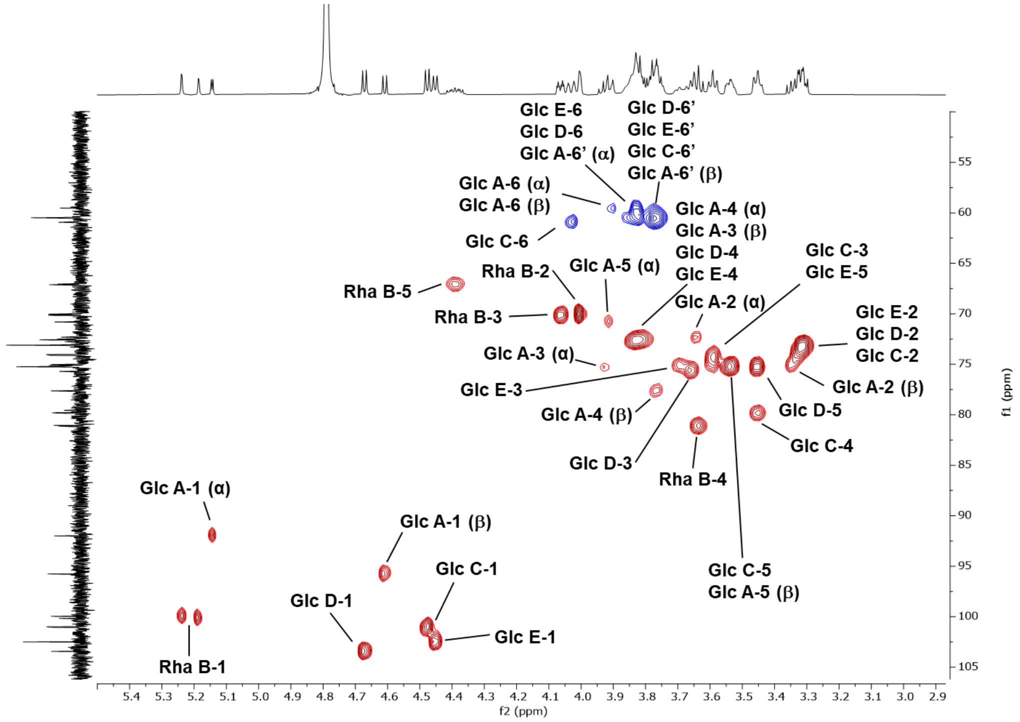

Figure S33
Excerpt of HSQC NMR (D_2O) of **5mer-III-di-PO_3^{2-}** with assignments.

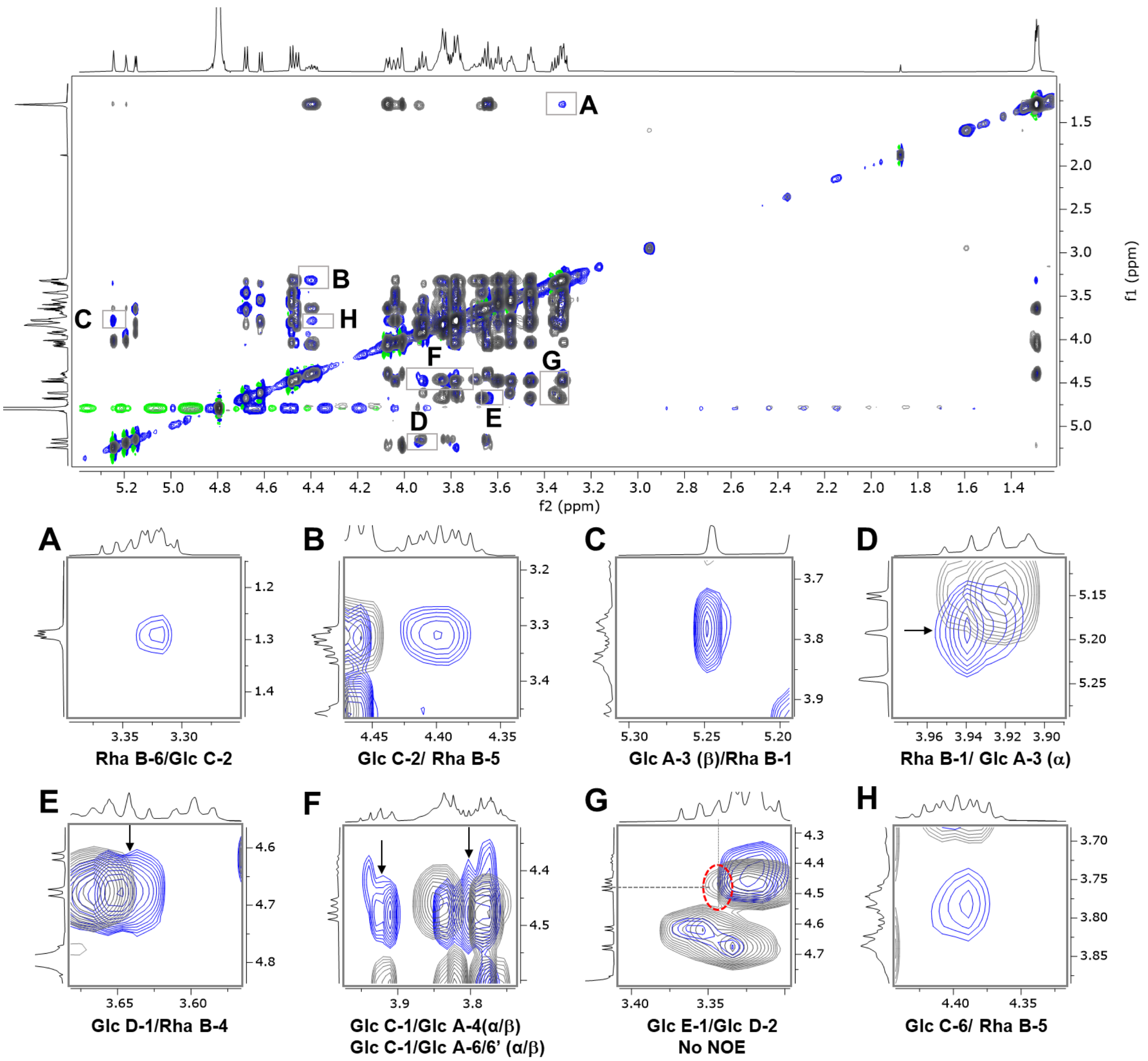

Figure S34
Superimposed 2D NOESY (green-blue, 700 MHz, d8 800 ms, D_2O) of **5mer-III-di-PO_3^{2-}** with assignments and 2D TOCSY spectrum (gray, 700 MHz, d9 150 ms). In panel G, the red cycle highlights the absence of the NOE signal between Glc E-1 and Glc D-2.

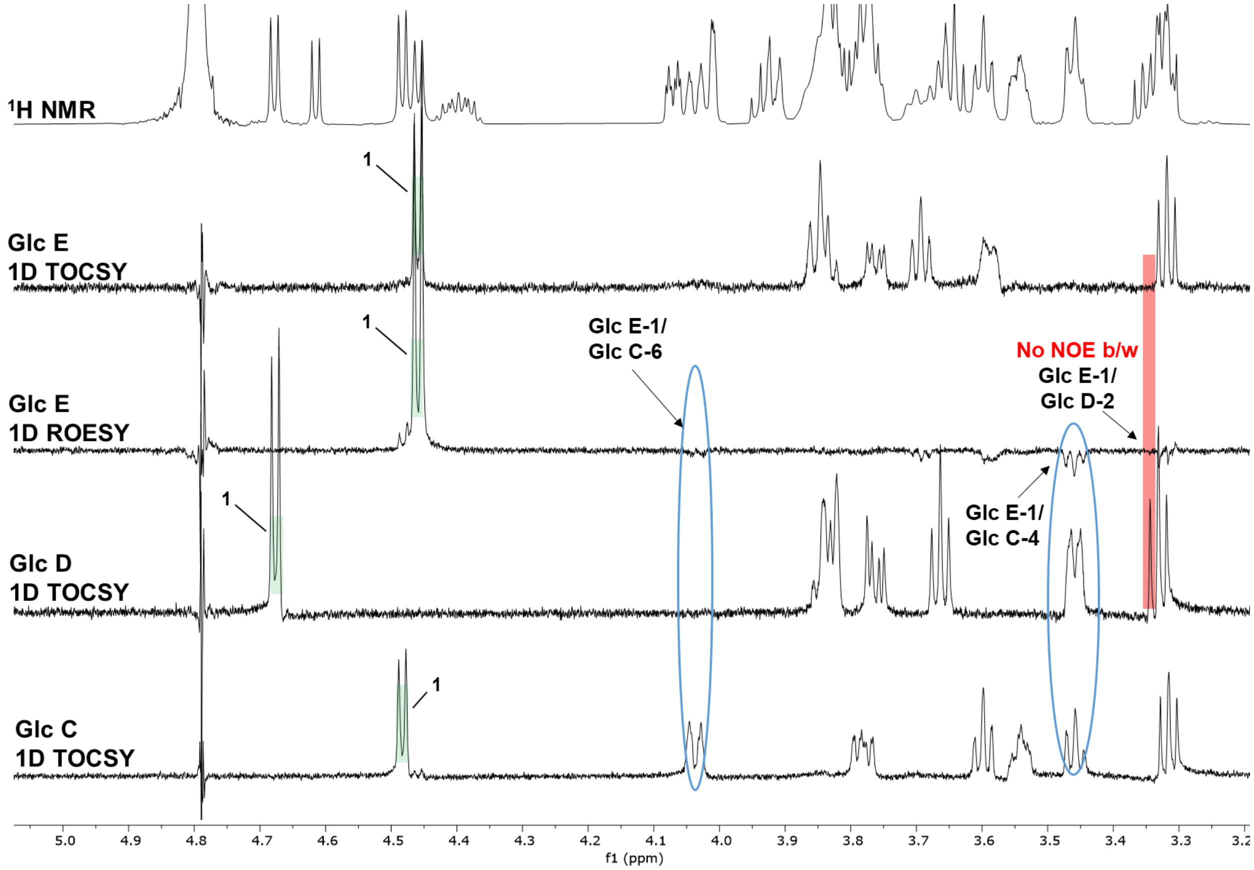

Figure S35
Overlay of 1D ROESY (700 MHz, p15 300 ms, D_2O) and 1D TOCSY (700 MHz, d9 200 ms) of **5mer-III-di-PO_3^{2-}**. The ROEs between Glc E-1/Glc C-6 and Glc E-1/Glc C-4 are highlighted with blue circles. No Key NOE signal was observed between Glc E-1/Glc D-2 (highlighted with red box).

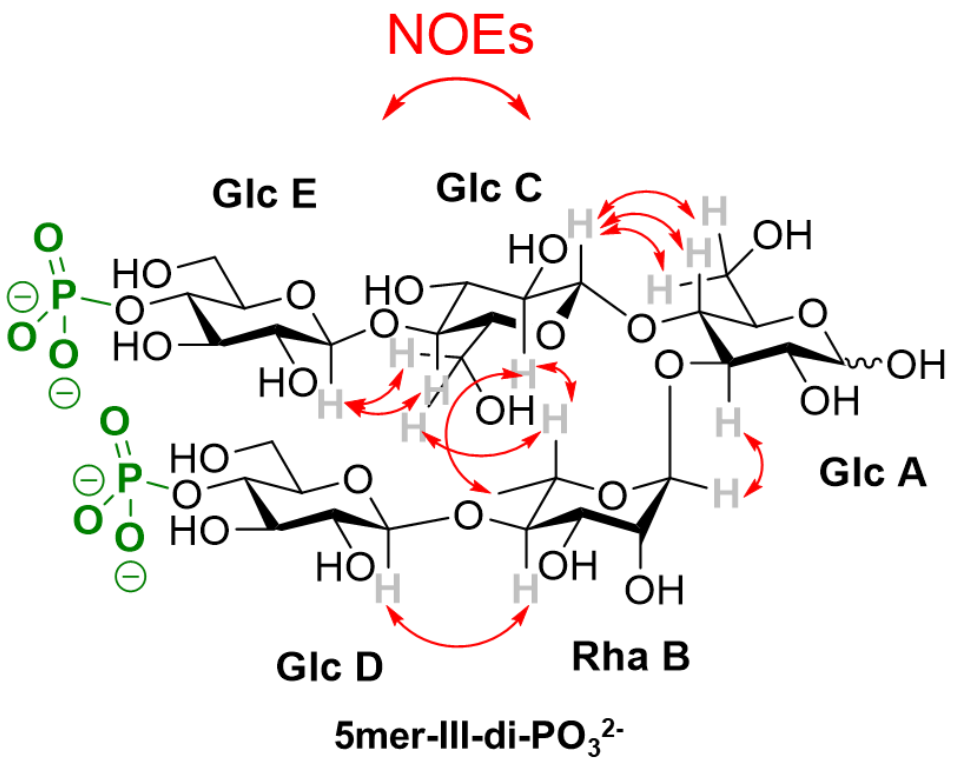

Figure S36
All experimentally observed NOEs (red arrows) of **5mer-III-di-PO$_3^{2-}$.**

4.3.1 Analysis of enzyme triggered dephosphorylation

Alkaline phosphatase (ALP) from bovine (calf) intestine (20 U/µL protein) was purchased from Sigma Aldrich. The phosphorylated compound (0.8 µmol, 1.6 M) (2.0% (w/w)) in DEA buffer was treated with ALP (100 U/µmol), final reaction volume 500 µL. The reaction was then incubated at 37°C for 5 h. The reaction progress was monitored by MALDI-ToF. Upon dephosphorylation, NMR analysis was performed to identify conformational differences between the phosphorylated and dephosphorylated 5mer.

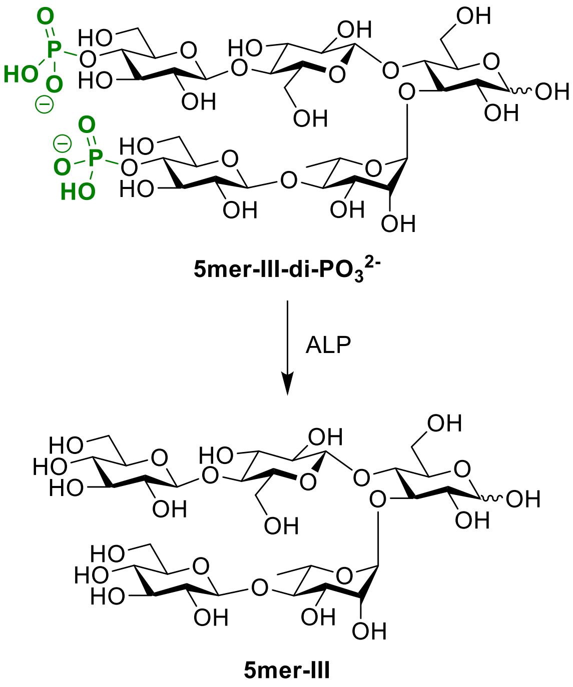

4.3.2 MALDI-TOF of 5mer-III-di-PO$_3^{2-}$ before and after dephosphorylation

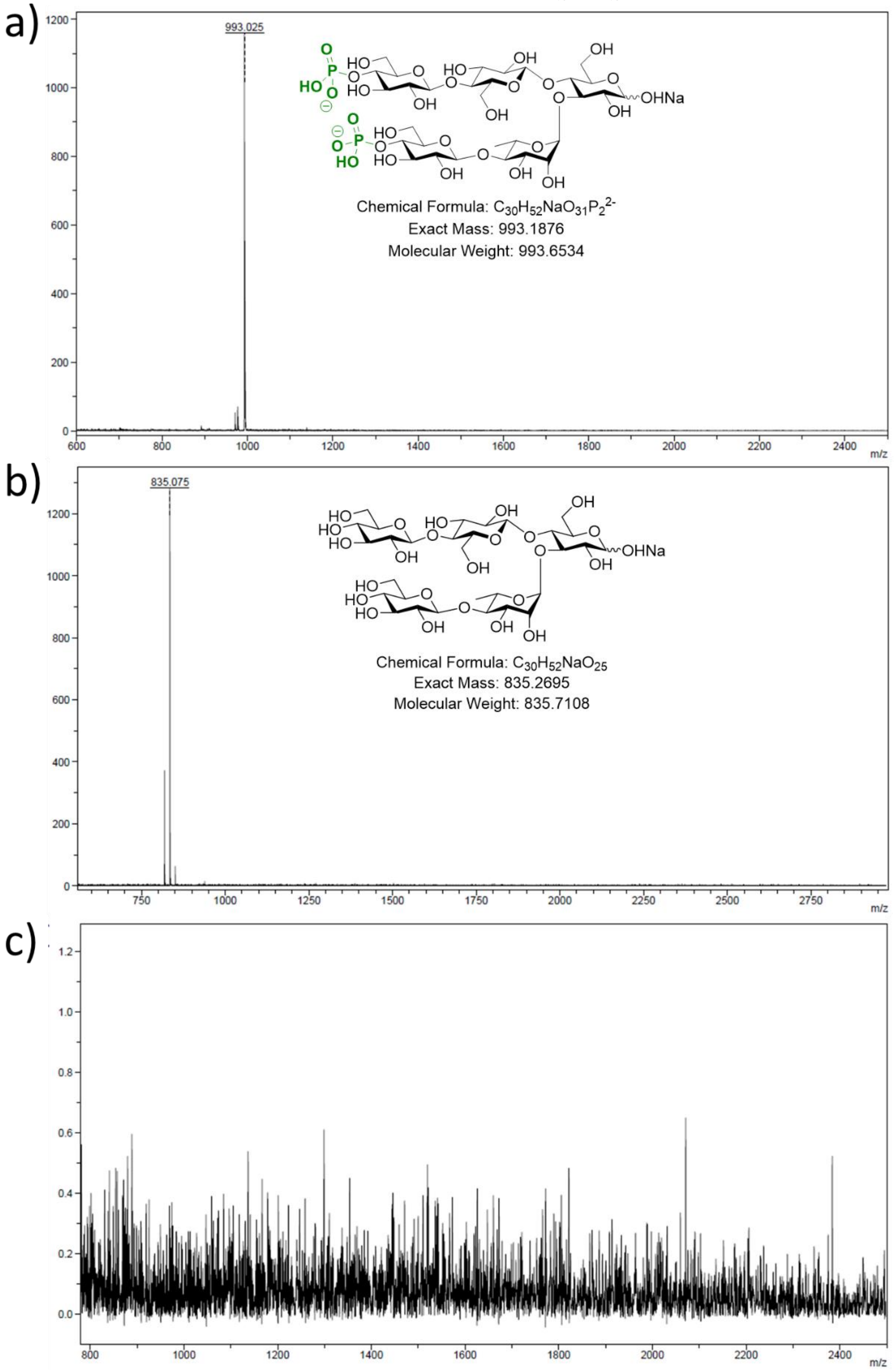

Figure S37
MALDI-ToF of **5mer-III-di-PO$_3^{2-}$** before (**a**-negative mode) and after (5 h, **b**-positive mode, **c**-negative mode) exposure to ALP.

4.3.3 NMR Studies after enzyme triggered dephosphorylation

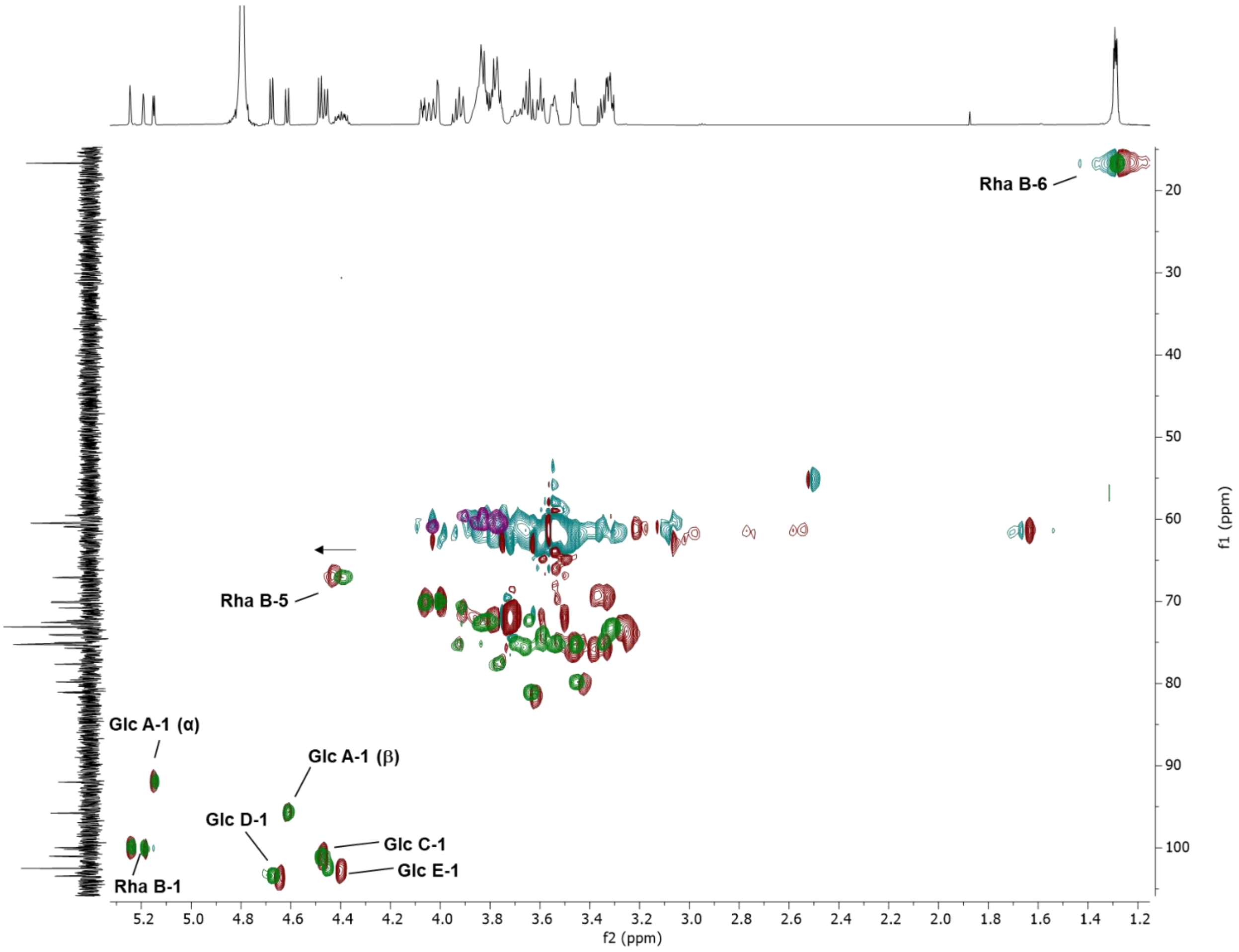

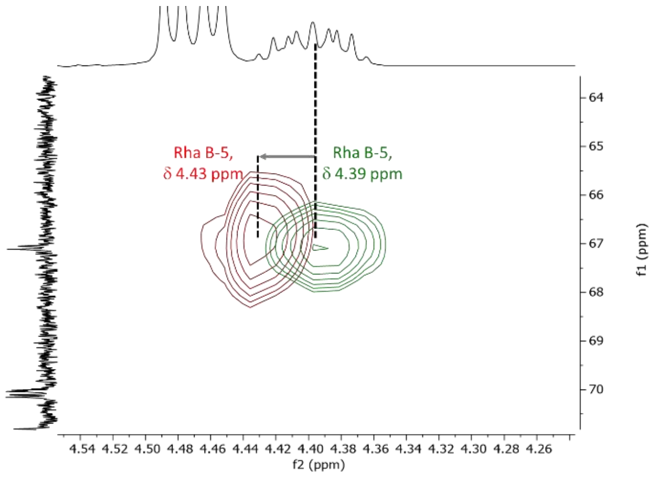

Figure S38
Superimposed 2D HSQC before (green-purple) and after (red-cyan) dephosphorylation of **5mer-III-di-PO$_3^{2-}$** with assignments. A downfield shift of Rha B-5 from 4.39 to 4.43 ppm after the cleavage of phosphate groups indicates the stabilization of the hairpin close conformation.

4.3.4 Inter-residue NOEs comparison before and after dephosphorylation

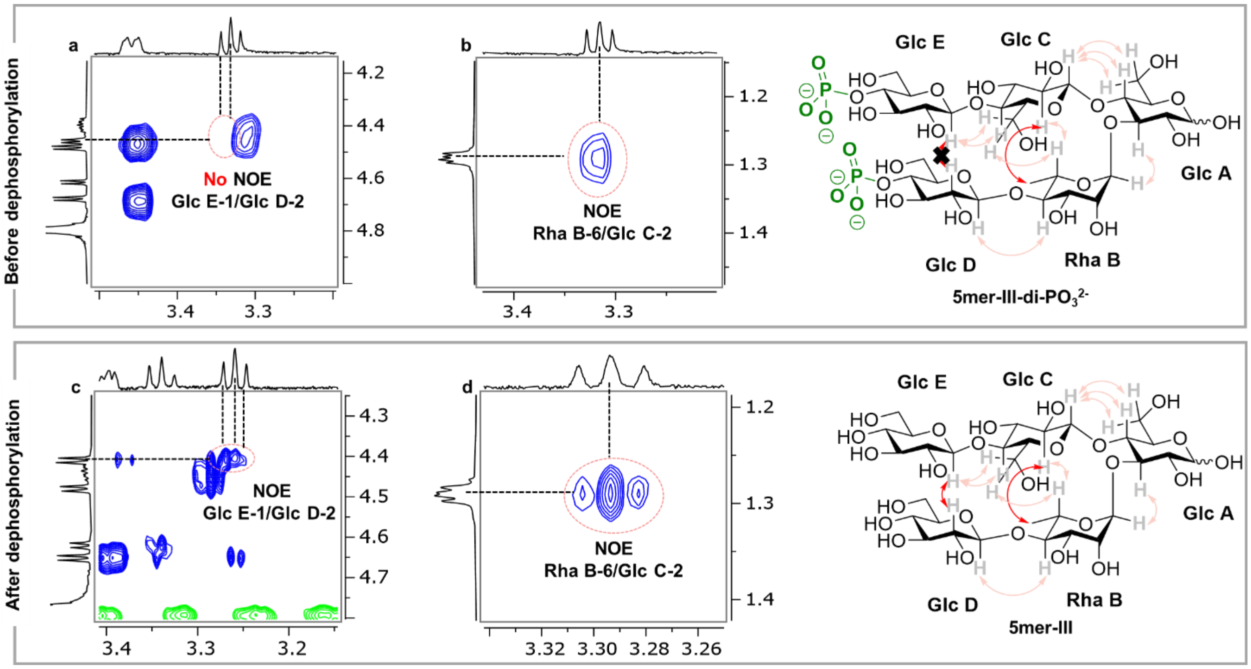

Figure S41

Comparison of the key inter-residue signals (Glc E-1/Glc D-2 and Rha B-6/Glc C-2) before (**5mer-III-di-**$\mathbf{PO_3^{2-}}$**)** and after dephosphorylation (**5mer-III**). The interstrand NOE was absent (**a**) in case of **5mer-III-di-**$\mathbf{PO_3^{2-}}$ and weak intensity was observed for Rha B-6/Glc C-2 (**b**) when compared it after dephosphorylation (**5mer-III**) (**d**). The key interstrand NOE signal was reinstalled (**c**) after dephosphorylation indicates the stabilization of the hairpin close conformation.

4.4 NMR characterization of 5mer-III-di-CO_2^-

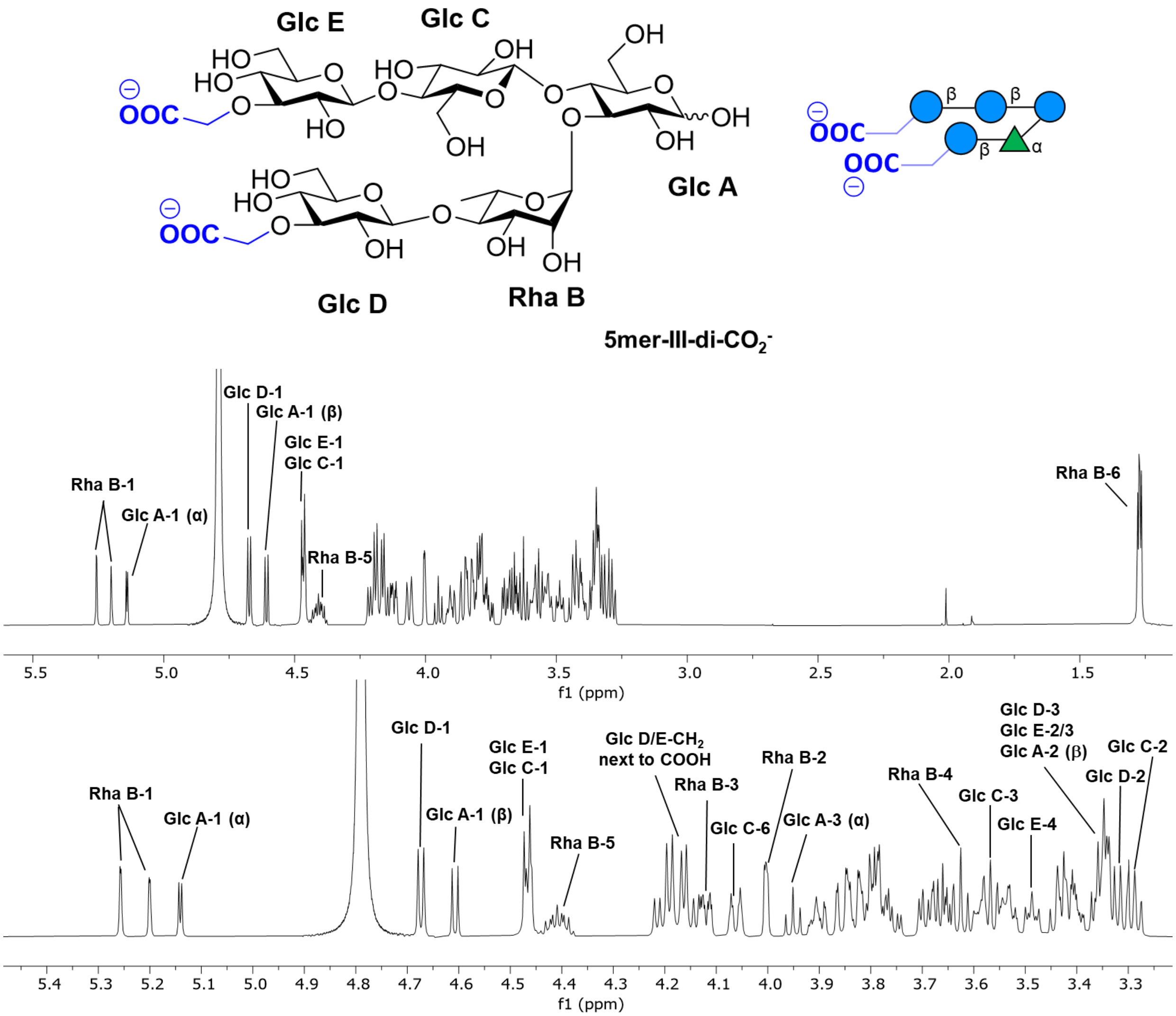

Figure S42
^{1}H NMR (700 MHz, D_2O) of **5mer-III-di-CO_2^-** with assignments.

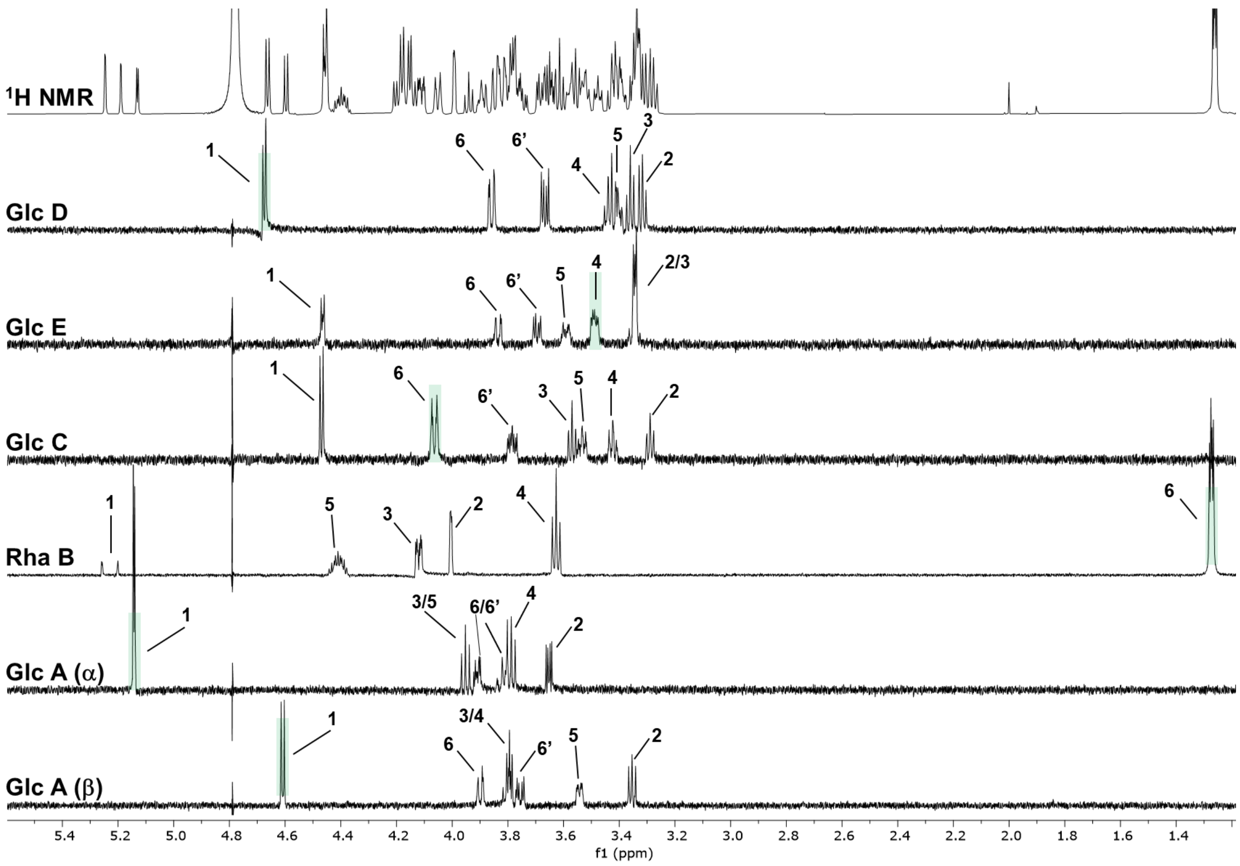

Figure S43
1D TOCSY (700 MHz, d9 200 ms, D_2O) of **5mer-III-di-CO_2^-** with assignments. Resonances chosen for selective excitation are highlighted with green boxes.

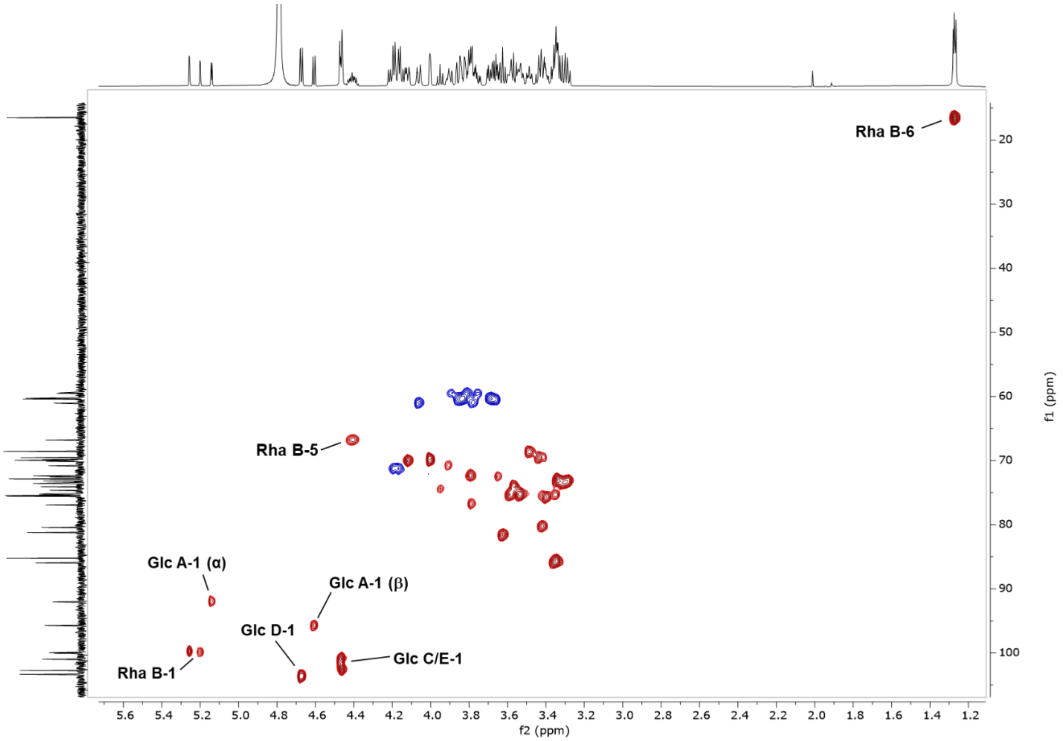

Figure S44
HSQC NMR (D_2O) of **5mer-III-di-CO_2^-** with assignments.

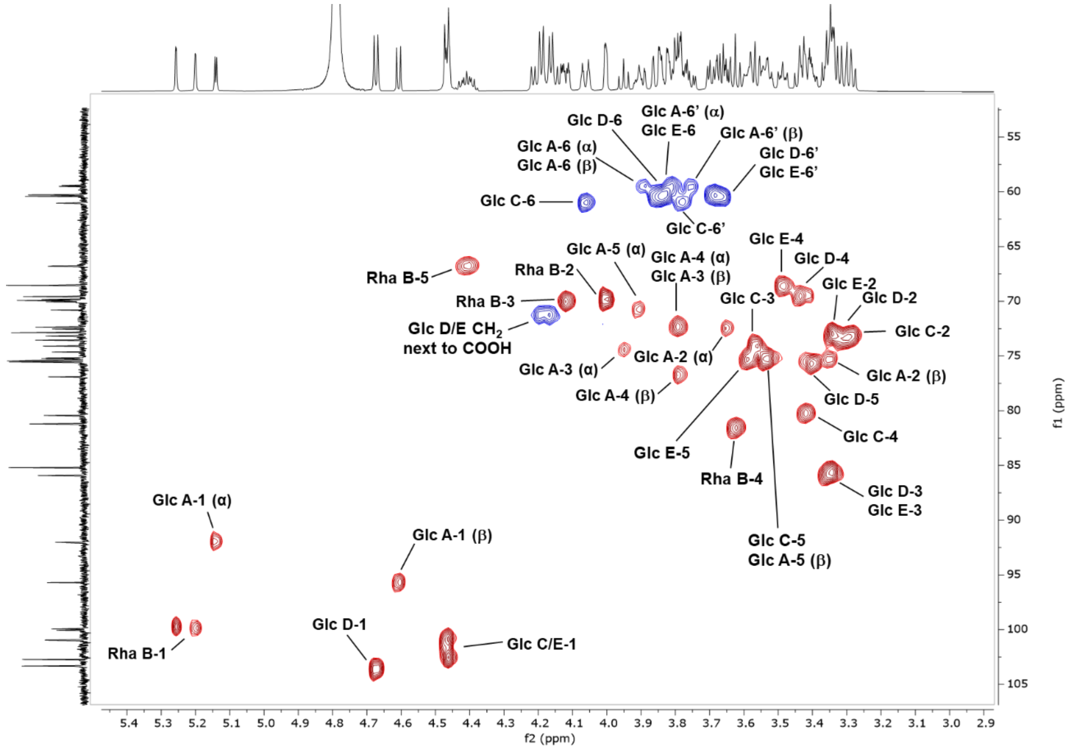

Figure S45
Excerpt of HSQC NMR (D_2O) of **5mer-III-di-CO_2^-** with assignments.

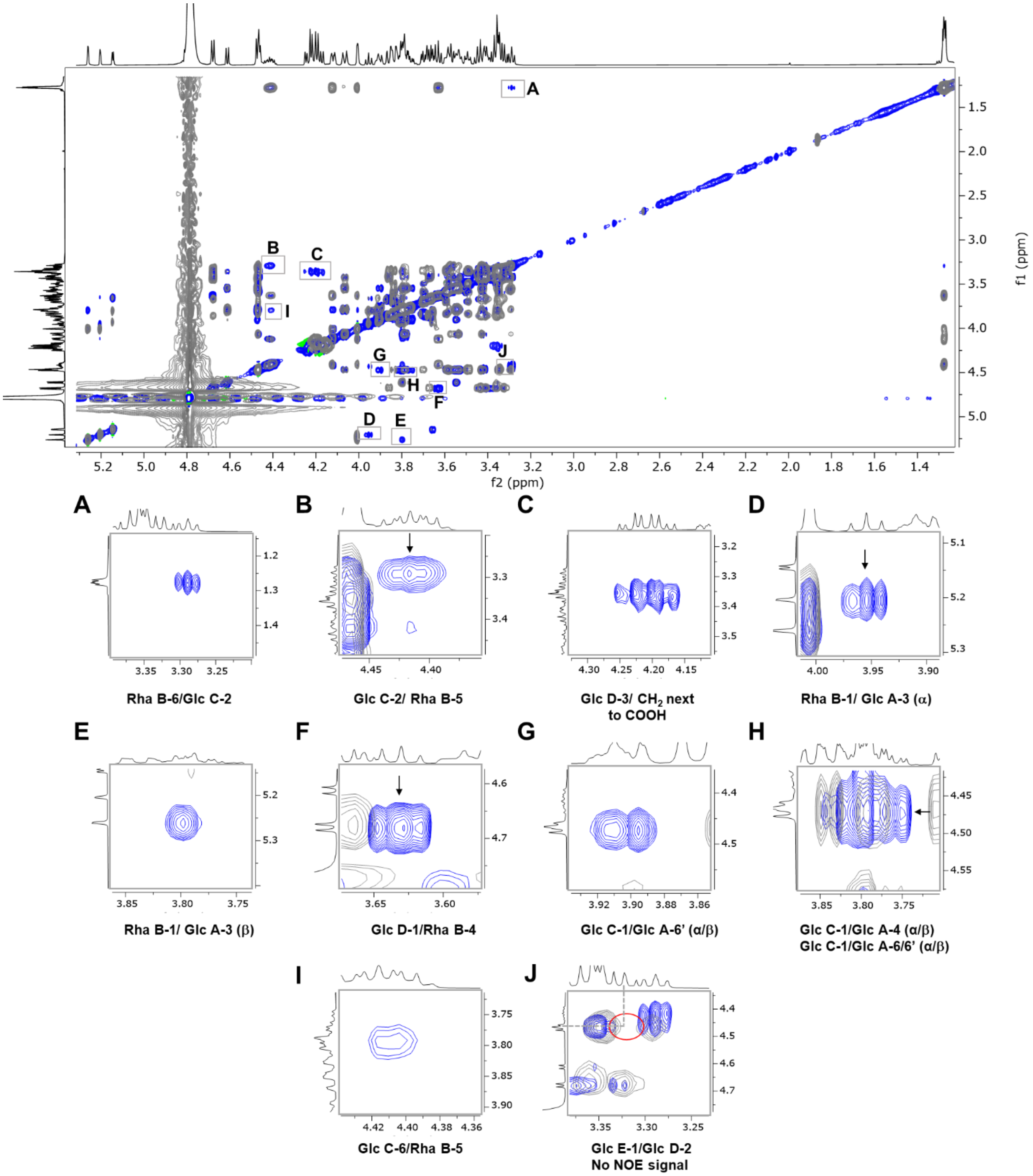

Figure S46

Superimposed 2D NOESY (green-blue, 700 MHz, d8 800 ms, D_2O) of **5mer-III-di-CO$_2^-$** at **pH 7.13** with assignments and 2D TOCSY spectrum (gray, 700 MHz, d9 150 ms, D_2O). In panel J, the red cycle highlights the absence of the NOE signal between Glc E-1 and Glc D-2.

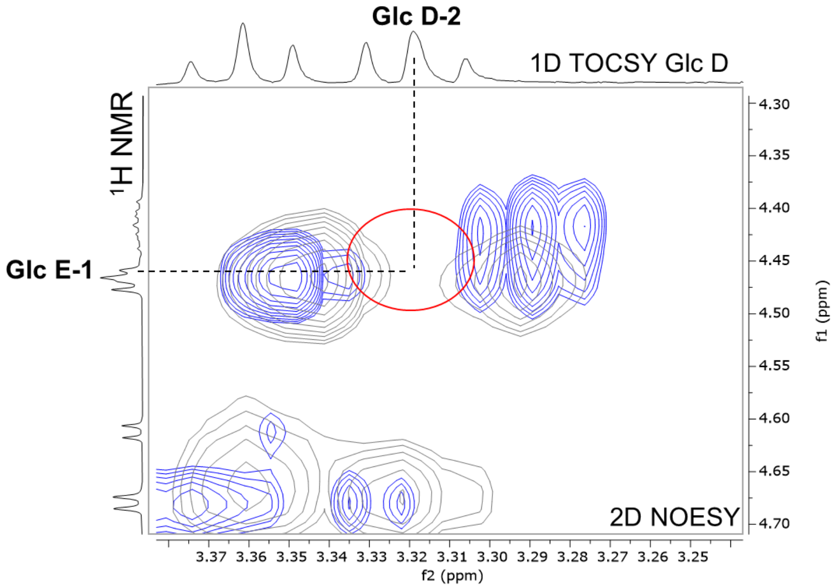

Figure S47
Excerpt of 2D NOESY (green-blue) and 2D TOCSY (gray) of **5mer-III-di-CO_2^-** overimposed. The horizontal trace shows the 1D TOCSY with selective excitation of Glc D-1. The inter-strand NOE cross peak was not observed at neutral pH (marked with dashed lines and red circle).

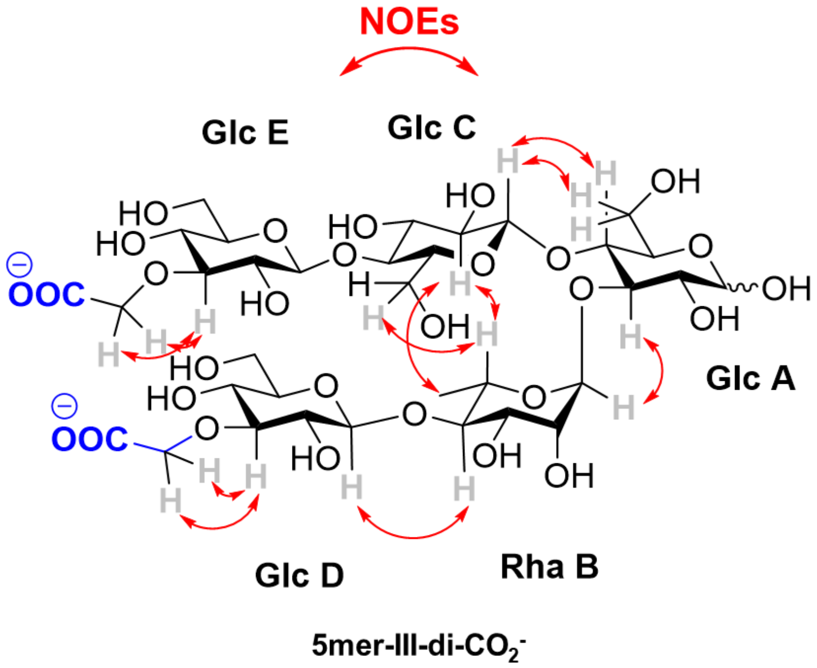

Figure S48
All experimentally observed NOEs (red arrows) of **5mer-III-di-CO_2^-** at **pH 7.13**.

4.4.1 pH titration of 5mer-III-di-CO_2^-

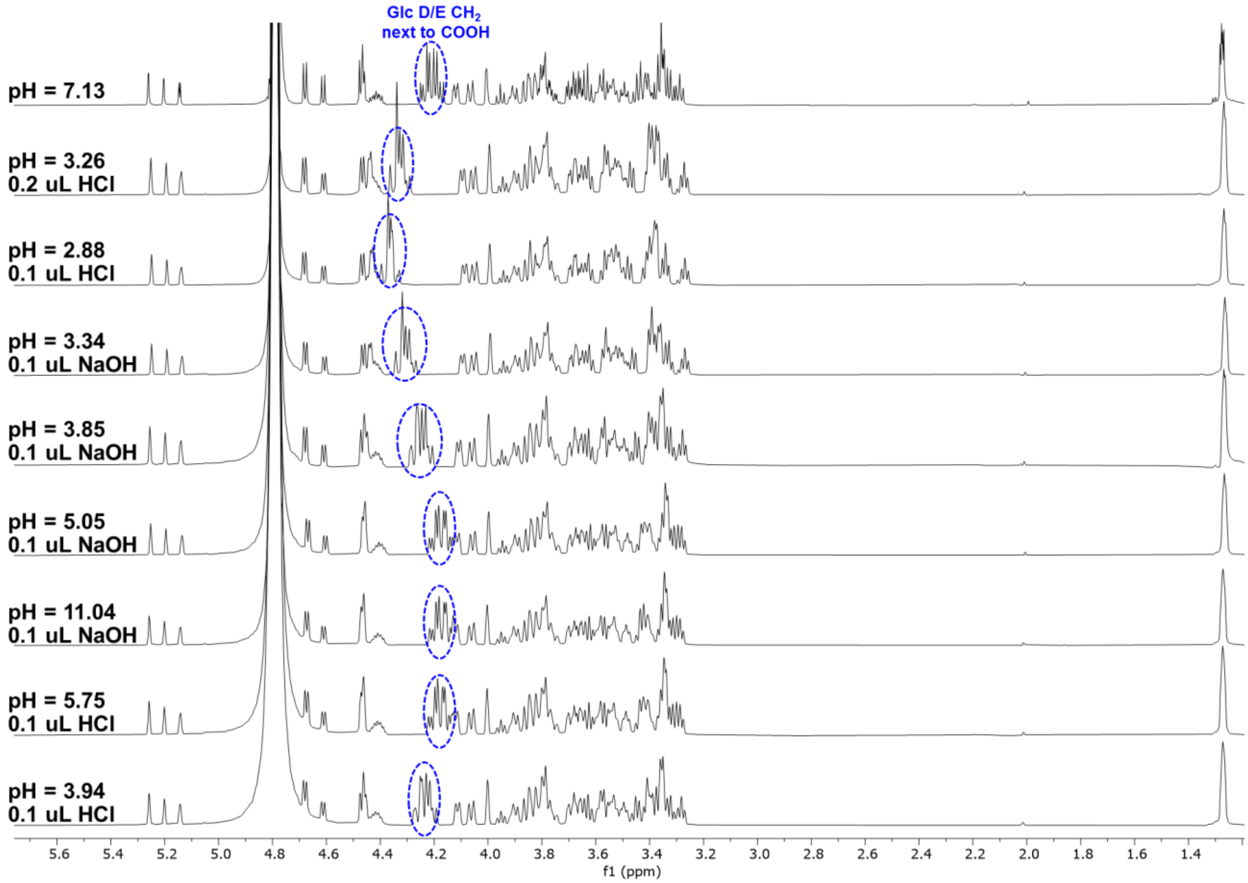

Figure S49 A.
^1H NMR (700 MHz, D_2O) titration of **5mer-III-di-CO_2^-**. pH of the sample was adjusted using 1M HCl and 1M NaOH solutions. The shift of selected signals during the titration is highlighted with: blue circle (Glc D/E-CH_2 next to COOH).

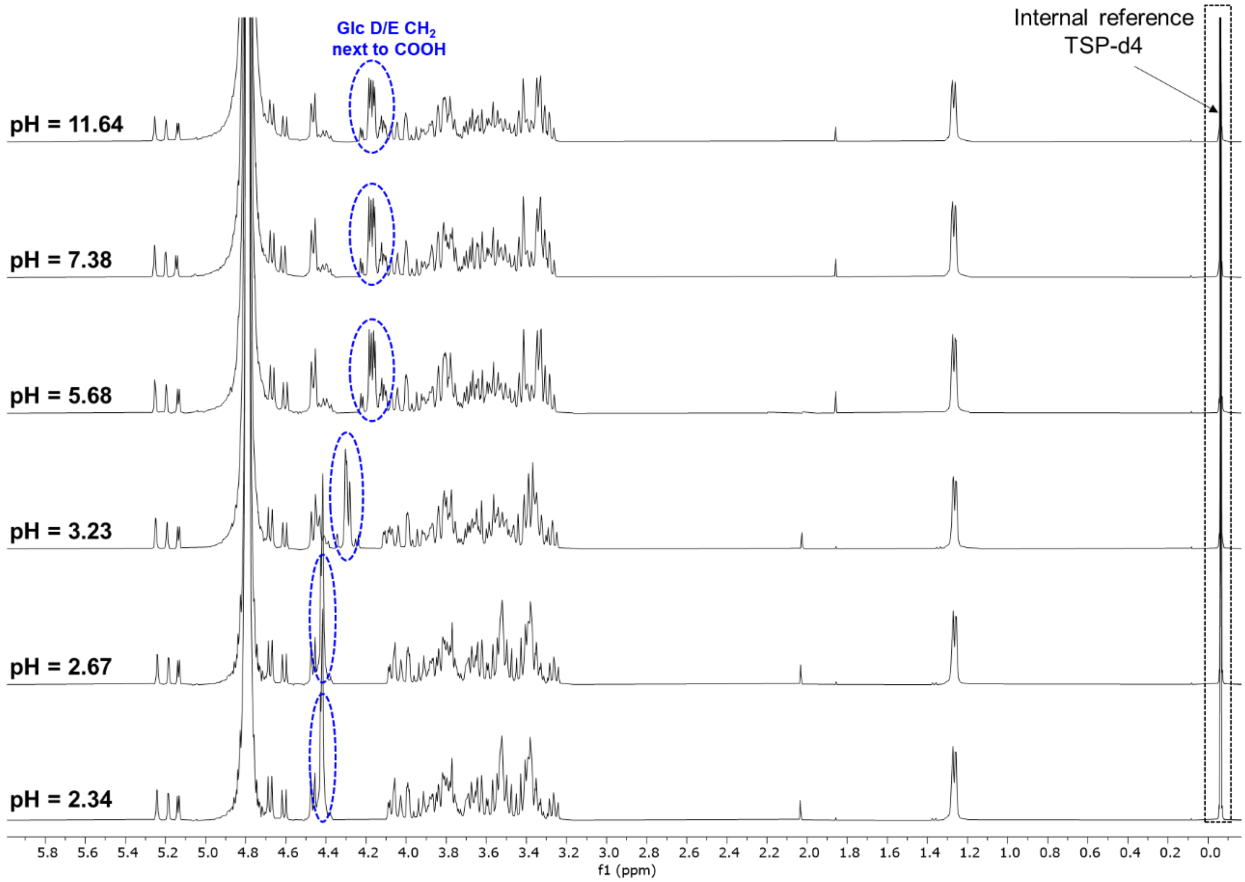

Figure S49 B

Titration repeated with internal reference (TSP-d4). Proton NMR analysis of **5mer-III-di-CO_2^-** in solution at different pH (D_2O, 700 MHz). To confirm whether upon changing the pH of the sample, the solvent peak changes or not. We repeated the titration on **5mer-III-di-CO_2^-** using an internal reference i.e. TSP-d4, which is commonly used for D_2O solvents in NMR. The concentration of the reference was half of the sample concentration. All the spectra recorded at different pH were stacked. There was no change observed in the solvent peak when the pH of the sample was changed.

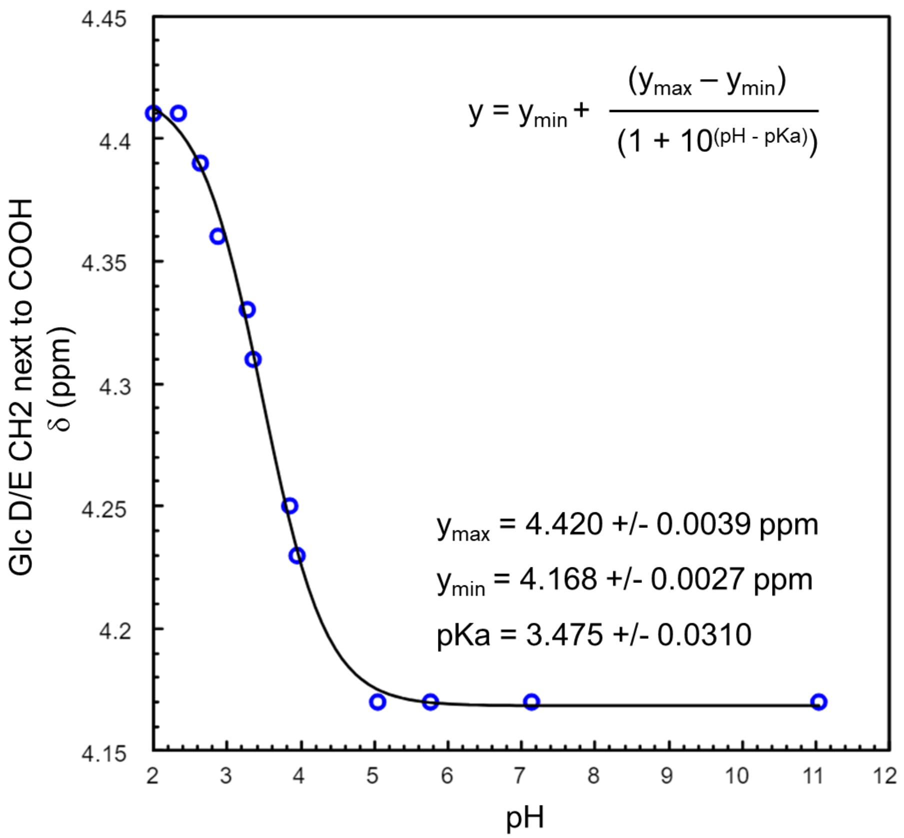

Figure S49 C

Plot of the chemical shifts of the Glc D/E CH_2 next to COOH as a function of pH for **5mer-III-di-CO_2^-**, showing *plateaus* above pH 5 that signify a fully ionic state, and plateaus below pH 3 that indicate a neutral state.

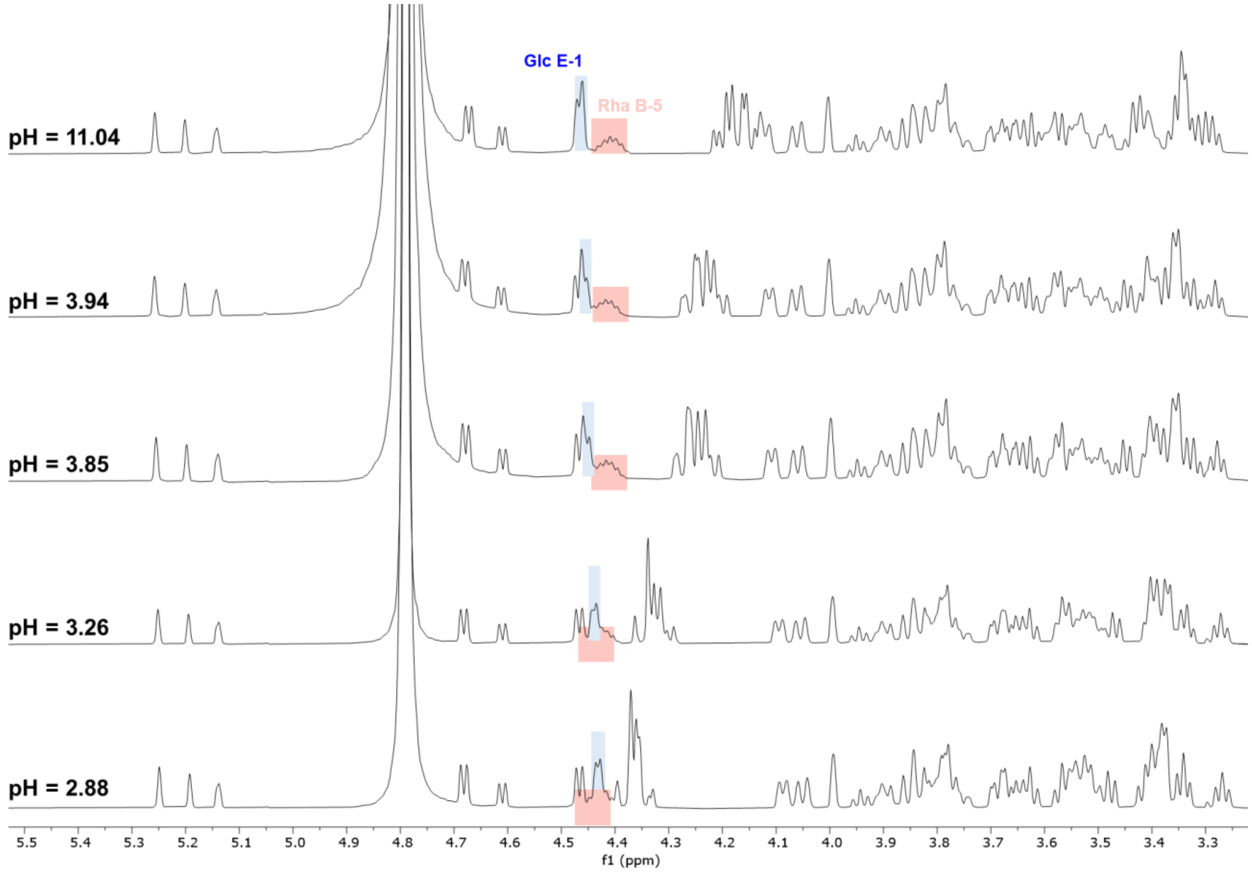

Figure S50
The shift of Glc E-1 (blue box) and Rha B-5 (red box) showing a linear correlation from high pH to low pH, indicating close proximity of strands at low pH. In our glycan hairpins, the close proximity between the two strands is encoded in the Glc E-1 chemical shift, since this nucleus may "feel" the presence of residue D. Indeed, upfield Glc E-1 shifts were observed only when Glc D on the opposite strands was present.[12] Herein, we have observed the same trend. For example, for **5mer-III-di-CO_2^-**, Glc E-1 moves upfield at low pH, highlighting the conformational changes induced by pH changes that modulate the proximity of Glc-D to Glc-E.

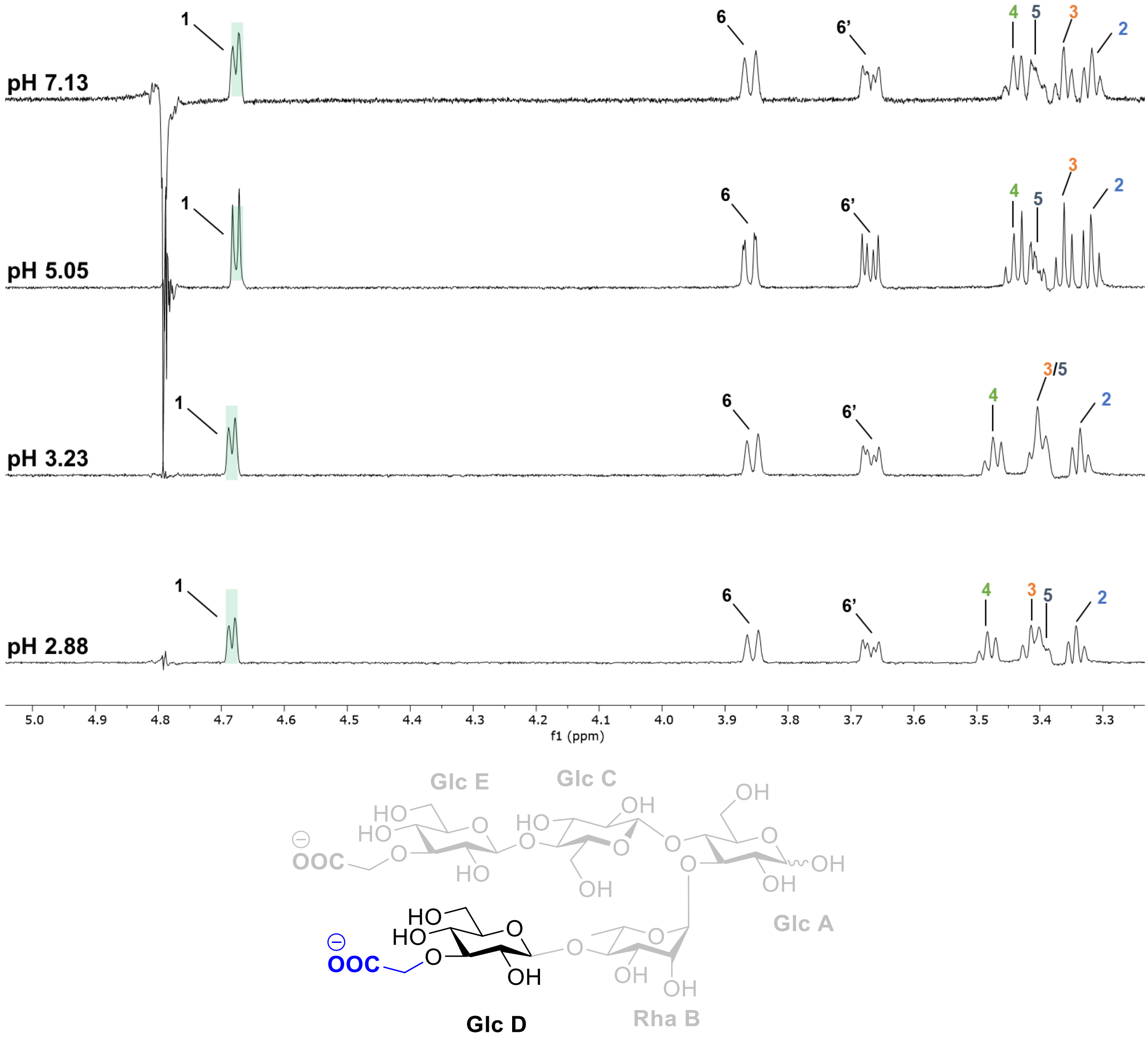

Figure S51
Selective 1D TOCSY (700 MHz, d9 200 ms, D_2O) spectra of **Glc D** of **5mer-III-di-CO_2^-** with assignments showing the peak shifts at different pH. Resonances chosen for selective excitation are highlighted with green boxes.

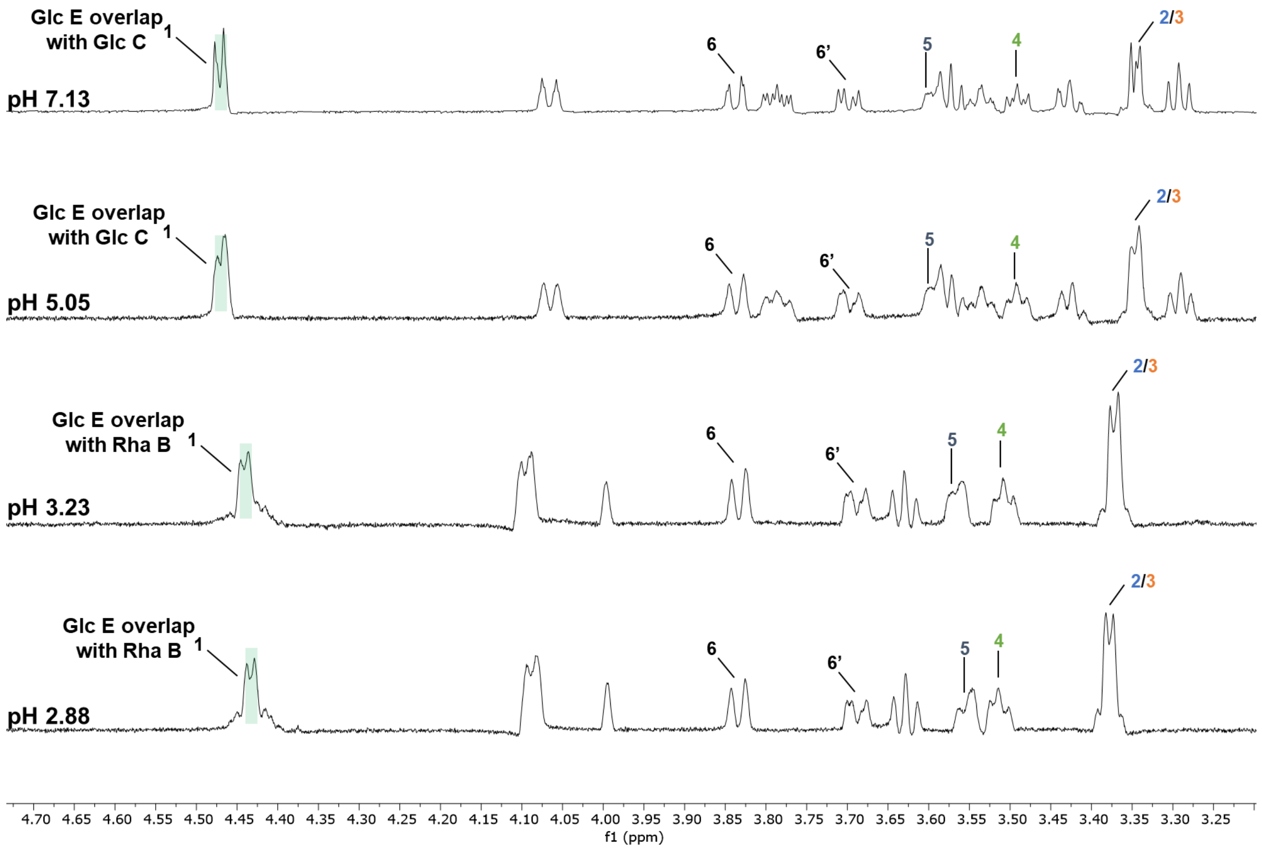

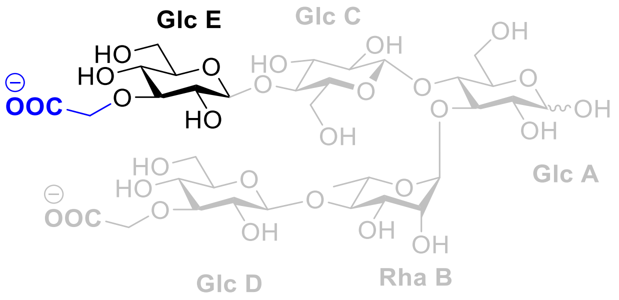

Figure S52
Selective 1D TOCSY (700 MHz, d9 200 ms, D_2O) spectra of **Glc E** of **5mer-III-di-CO_2^-** (**Glc E-1** overlaps with **Glc C-1** at high pH and with **Rha B-5** at low pH) with assignments of **Glc E** showing the peak shifts at different pH. Resonances chosen for selective excitation are highlighted with green boxes.

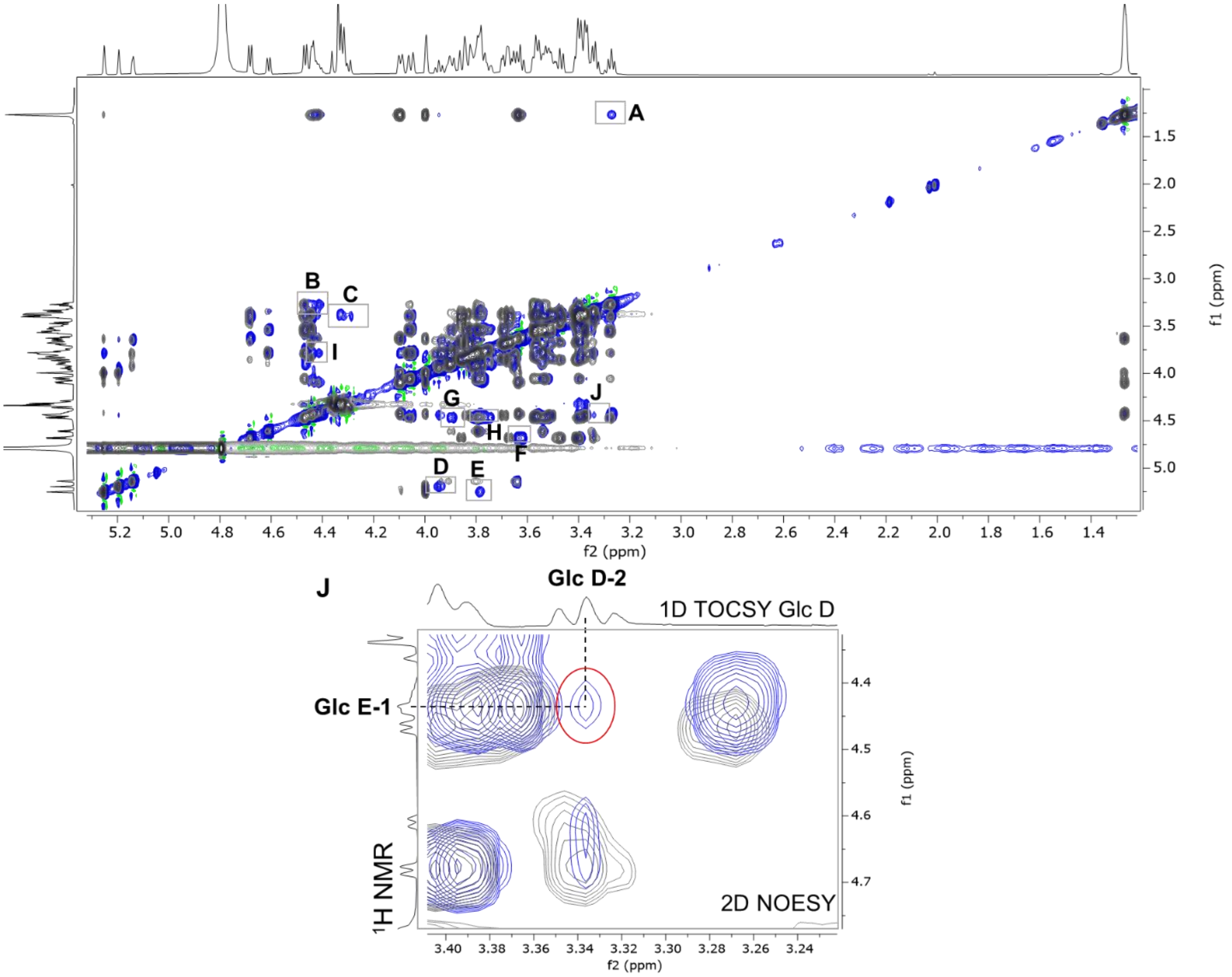

Figure S53

Superimposed 2D NOESY (green-blue, 700 MHz, d8 800 ms, D_2O) of **5mer-III-di-CO$_2^-$** at **pH 3.26** with assignments and 2D TOCSY spectrum (gray, 700 MHz, d9 150 ms, D_2O). The pH was adjusted from **7.13** to **3.26** by adding 0.2 uL of a 1 M HCl solution. At **pH 3.26**, all the NOEs observed at **pH 7.13** were detected (**Figure S46**) except for the inter-strand NOE signal between the two strands Glc E-1/Glc D-2 (**J**).

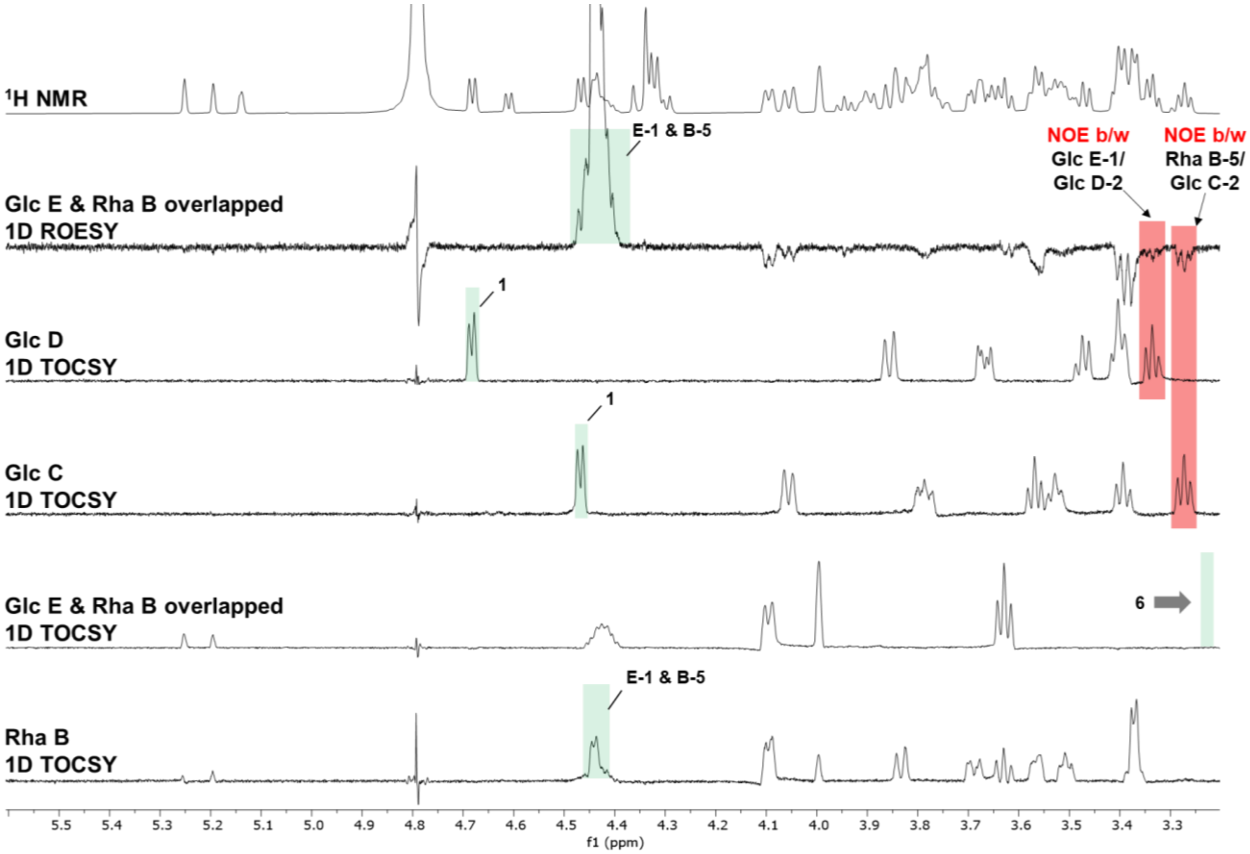

Figure S54
Overlay of 1D ROESY (700 MHz, p15 300 ms, D_2O) and 1D TOCSY (700 MHz, d9 200 ms, D_2O) of **5mer-III-di-CO_2^-**. Key NOE signal between Glc E-1/Glc D-2 and Rha B-5/Glc C-2 (highlighted with red box) was observed at **pH 3.26**.

.

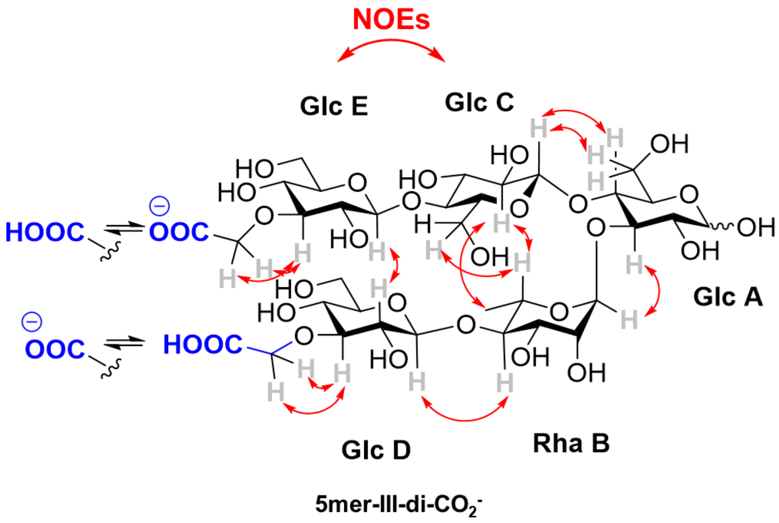

Figure S55
All experimentally observed NOEs (red arrows) of **5mer-III-di-CO_2^-** at **pH 3.26**.

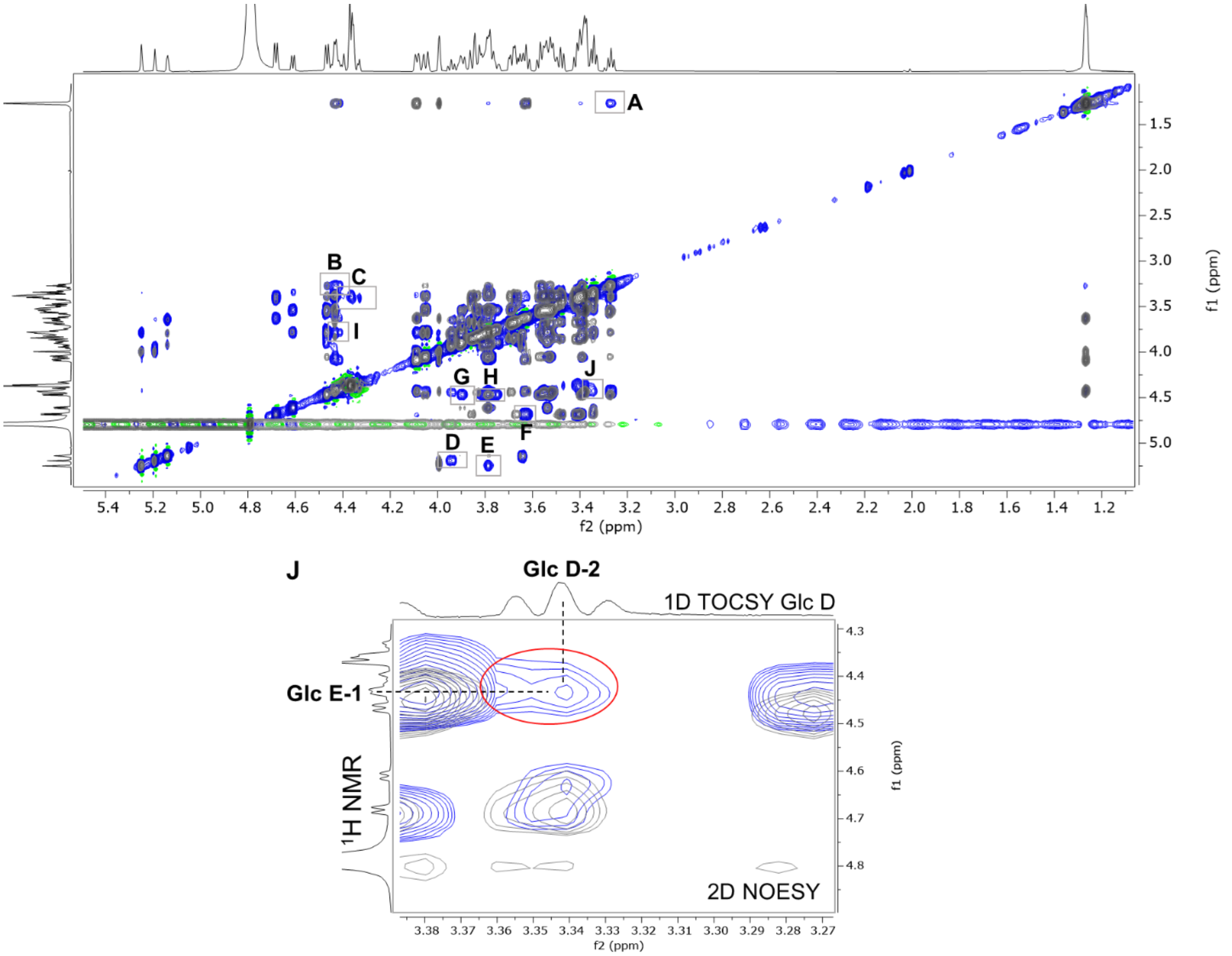

Figure S56

Superimposed 2D NOESY (green-blue, 700 MHz, d8 800 ms, D_2O) of **5mer-III-di-CO_2^-** at **pH 2.88** with assignments and 2D TOCSY spectrum (gray, 700 MHz, d9 150 ms, D_2O). The pH was adjusted from **3.26** to **2.88** by adding 0.1 uL of a 1 M HCl solution. At **pH 2.88**, all the NOEs observed at **pH 7.13** were detected (**Figure S46**) except for the inter-strand NOE signal between the two strands Glc E-1/Glc D-2 (**J**).

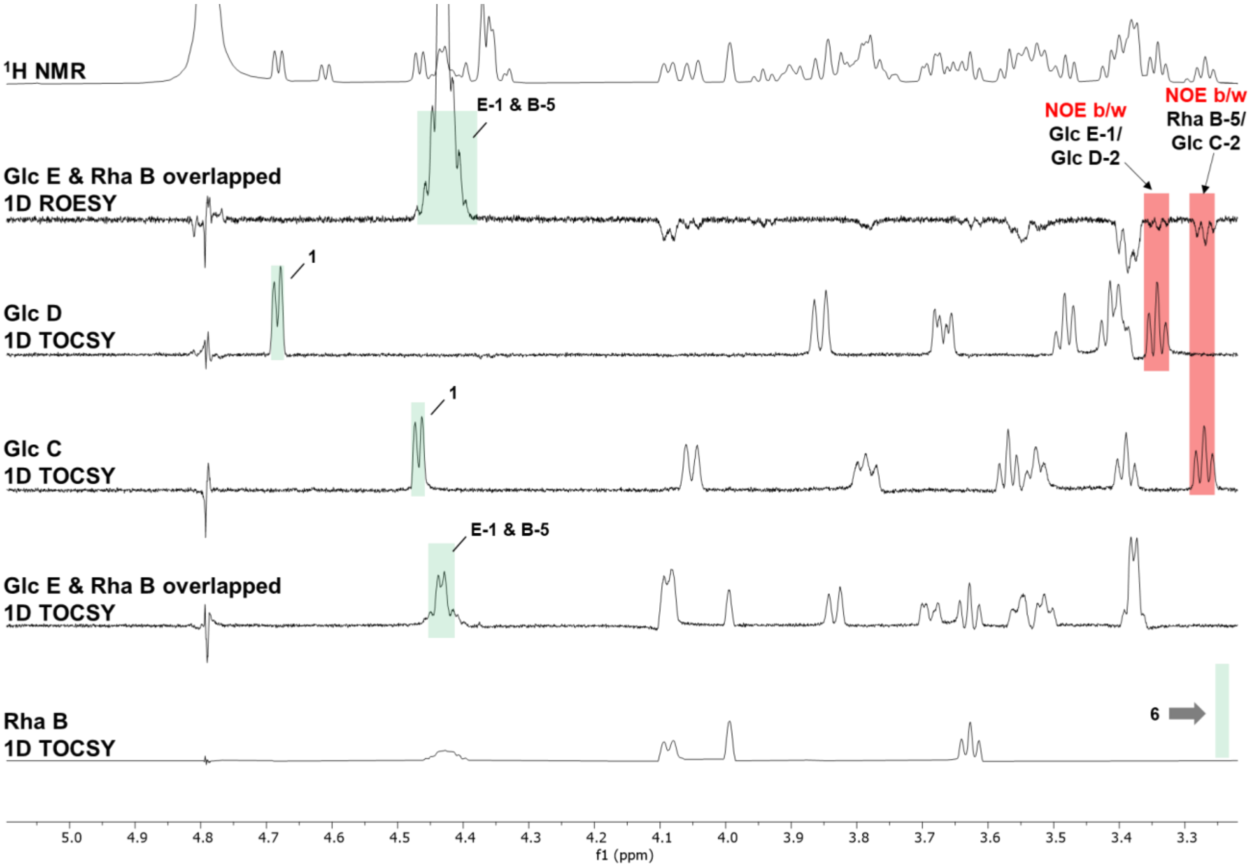

Figure S57
Overlay of 1D ROESY (700 MHz, p15 300 ms, D_2O) and 1D TOCSY (700 MHz, d9 200 ms, D_2O) of **5mer-III-di-CO_2^-**. Key NOE signal between Glc E-1/Glc D-2 and Rha B-5/Glc C-2 (highlighted with red box) was observed at **pH 2.88**.

.

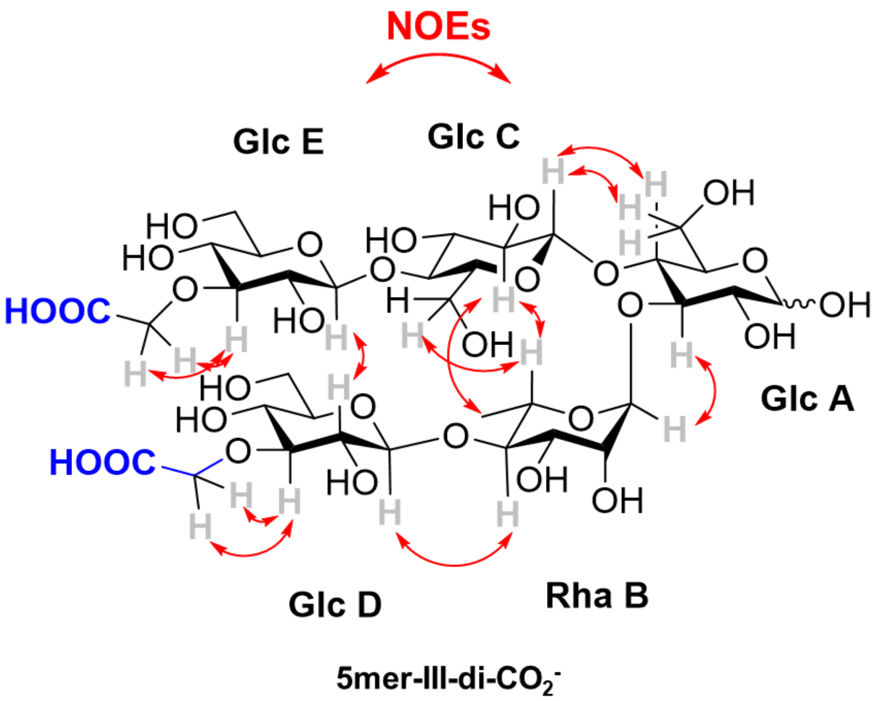

Figure S58
All experimentally observed NOEs (red arrows) of **5mer-III-di-CO$_2^-$** at **pH 2.88**.

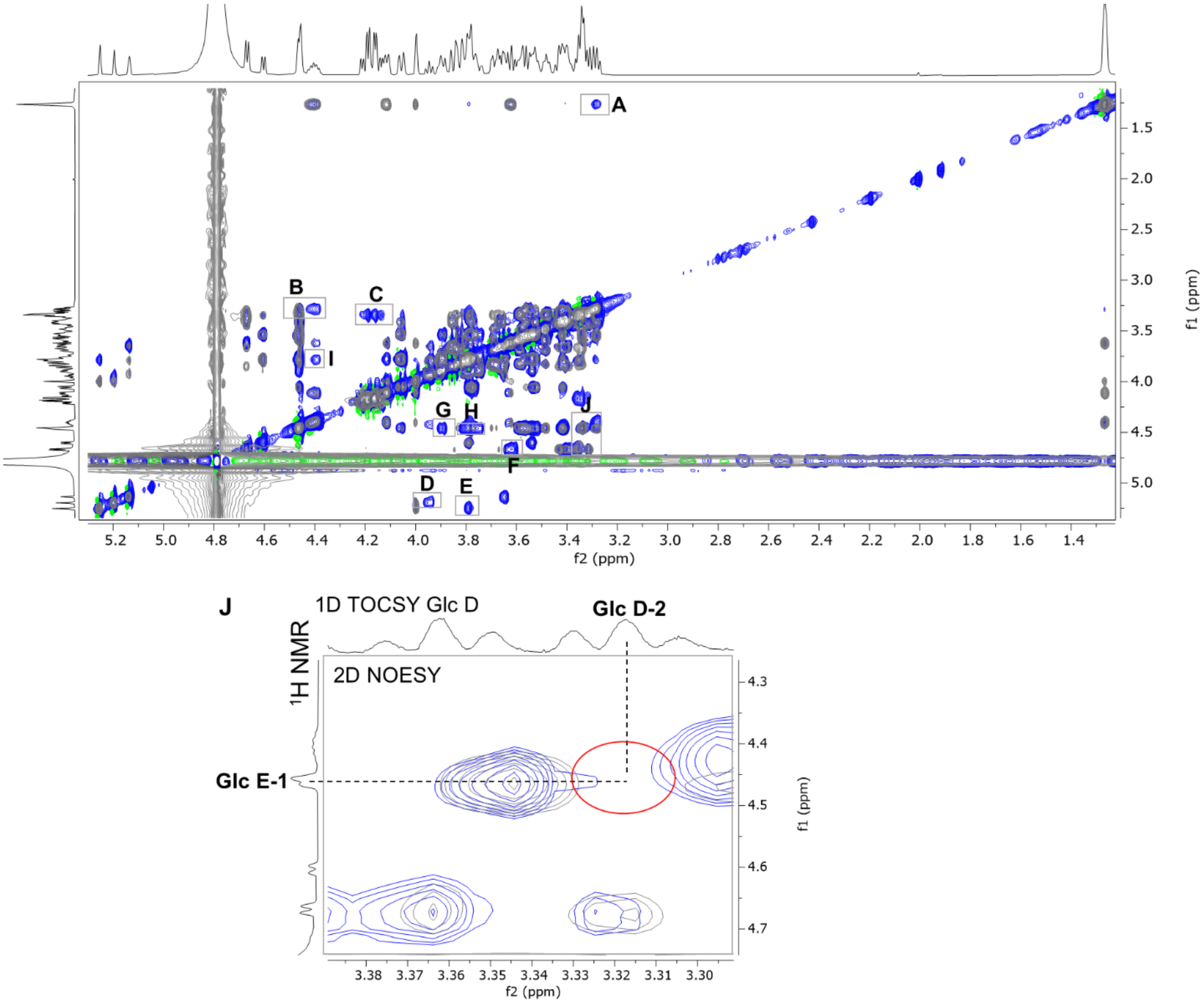

Figure S59

Superimposed 2D NOESY (green-blue, 700 MHz, d8 800 ms, D_2O) of **5mer-III-di-CO_2^-** at **pH 5.05** with assignments and 2D TOCSY spectrum (gray, 700 MHz, d9 150 ms, D_2O). The pH was adjusted from **2.88** to **5.05** by adding 0.3 uL of a 1 M NaOH solution. At **pH 5.05**, all the NOEs observed at **pH 7.13** were detected (**Figure S46**) except for the inter-strand NOE signal between the two strands Glc E-1/Glc D-2 (**J**).

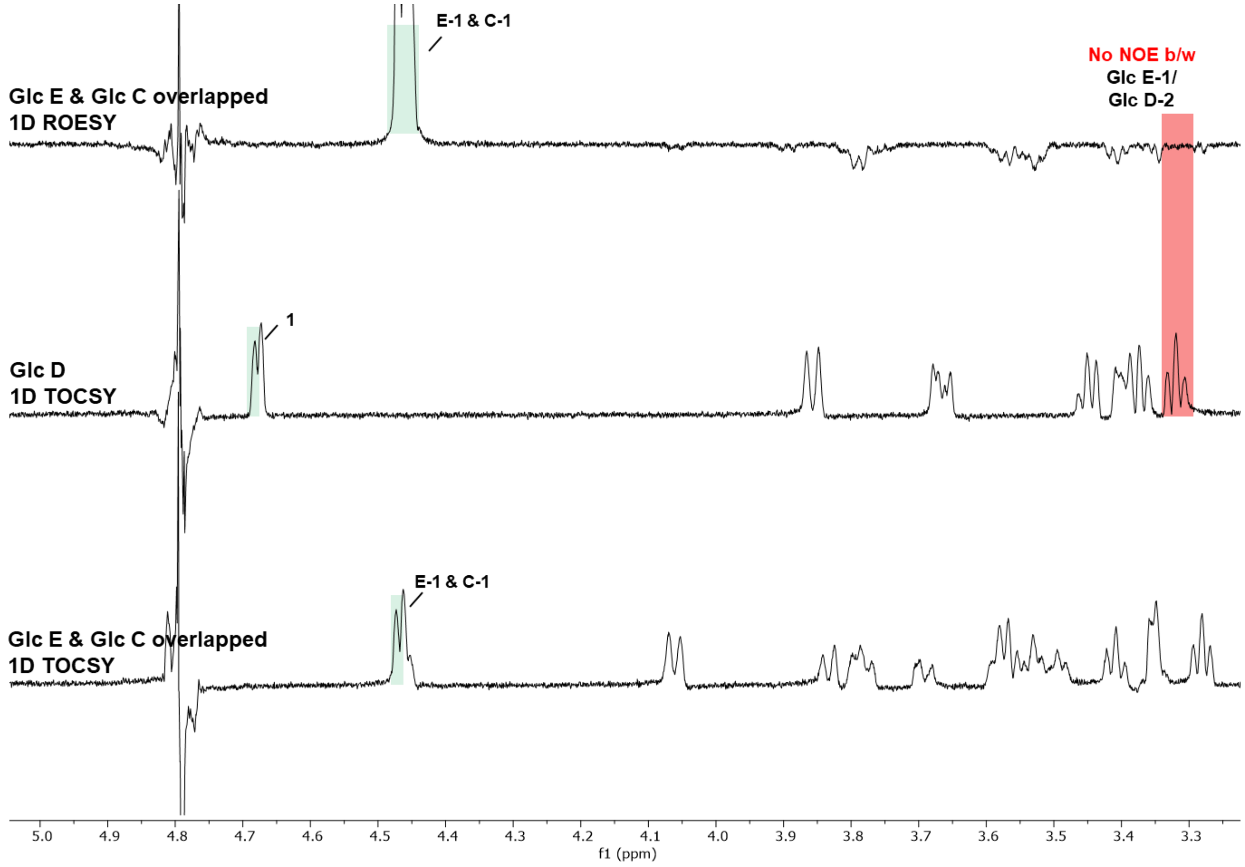

Figure S60
Overlay of 1D ROESY (700 MHz, p15 300 ms, D_2O) and 1D TOCSY (700 MHz, d9 200 ms, D_2O) of **5mer-III-di-CO_2^-**. No NOE signal between Glc E-1/Glc D-2 (highlighted with red box) was observed at **pH 5.05**.

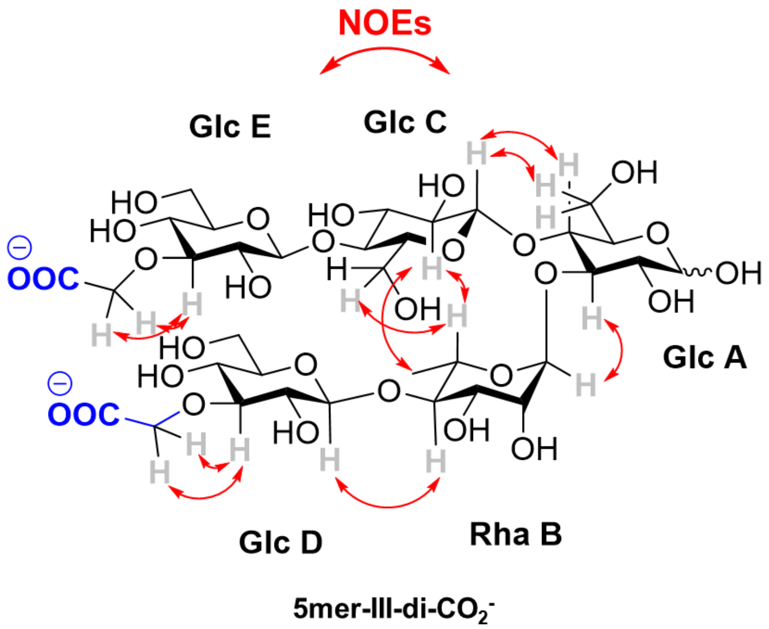

Figure S61
All experimentally observed NOEs (red arrows) of **5mer-III-di-CO_2^-** at **pH 5.05**.

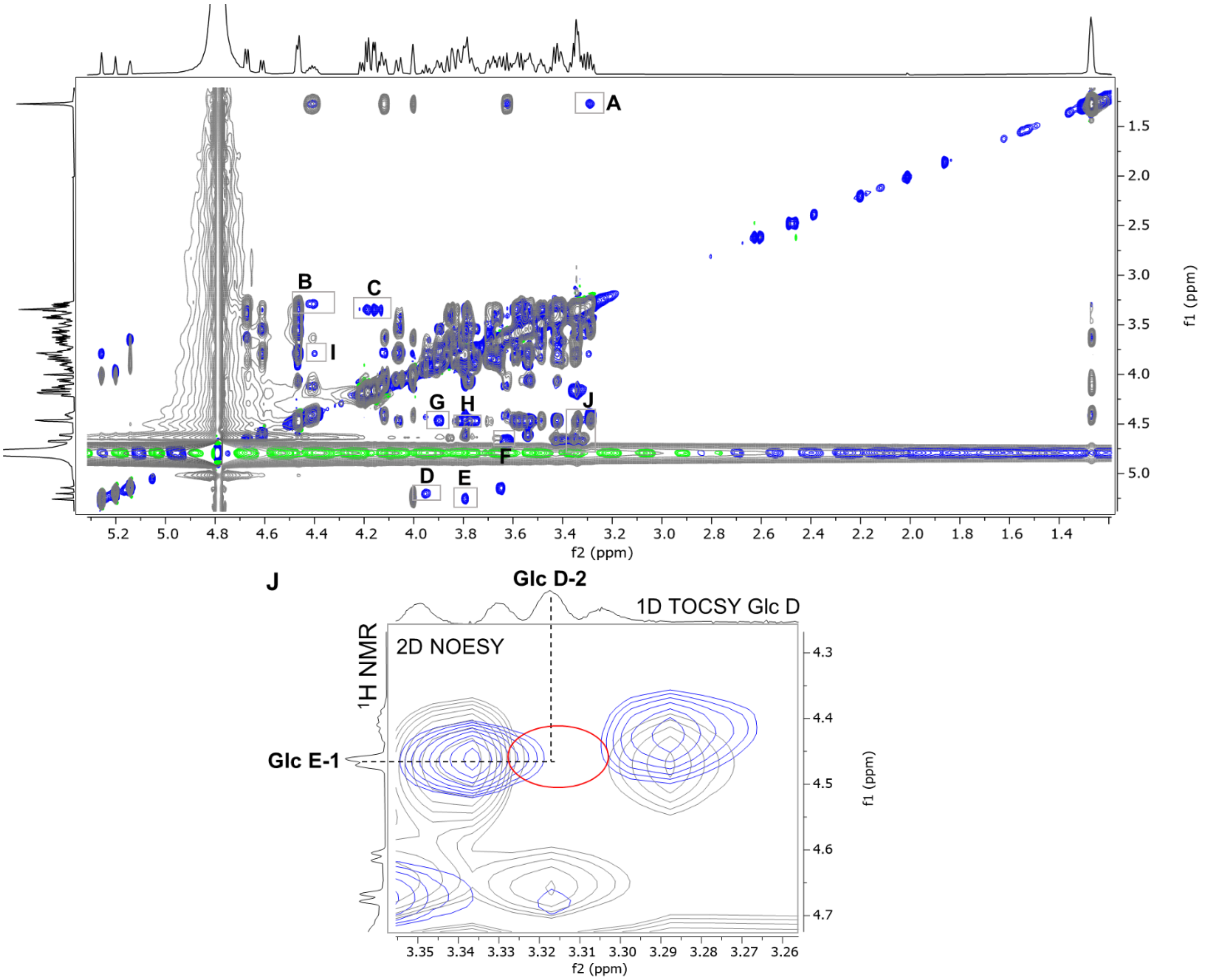

Figure S62

Superimposed 2D NOESY (green-blue, 700 MHz, d8 800 ms, D_2O) of **5mer-III-di-CO_2^-** at **pH 11.04** with assignments and 2D TOCSY spectrum (gray, 700 MHz, d9 150 ms, D_2O). The pH was adjusted from **5.05** to **11.04** by adding 0.1 uL of a 1 M NaOH solution. At **pH 11.04**, all the NOEs observed at **pH 7.13** were detected (**Figure S46**) except for the inter-strand NOE signal between the two strands Glc E-1/Glc D-2 (**J**).

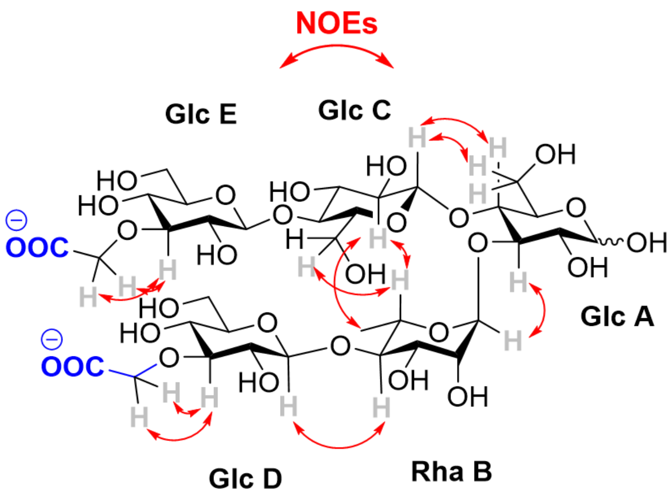

5mer-III-di-CO$_2^-$

Figure S63
All experimentally observed NOEs (red arrows) of **5mer-III-di-CO$_2^-$** at pH 11.04.

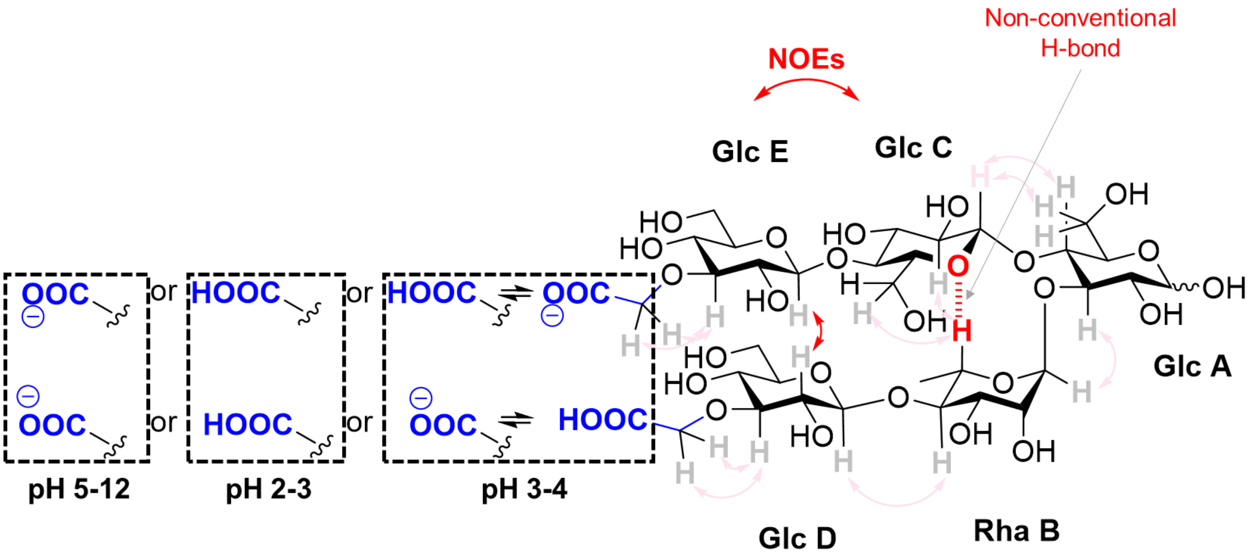

5mer-III-di-CO_2^-

Figure S64
Chemical structure representing different de(protonation) states of **5mer-III-di-CO_2^-**.

pH	Glc E-1/GlcD-2 NOE signal	δ Rha B-5 (ppm)
**7.13**	No	4.40
**3.26**	Yes	4.42
**2.88**	Yes	4.43
**3.34**	Yes	4.42
**3.85**	Yes (weak)	4.42
**5.05**	No	4.40
**11.04**	No	4.40

Table S02
Overall analysis of **5mer-III-di-CO_2^-** at different pH. Inter-strand NOE signal Glc E-1/Glc D-2 and Rha B-5 chemical shifts are shown.

4.4.2 Examination of scattering curves at different pH

SAXS experiment of the **5mer-III-di-CO_2^-** were obtained in the absence of salt and only was added portions of HCl or NaOH solutions to evaluation the conformation of this molecule with changes in pH. It was avoided high salt concentration because in our study we are especially interested in analyzing the influence of intra- and intermolecular Coulombic interaction in the overall conformation of this molecule. Subsequently, scattering is expected to originate from chain conformation mostly from Coulombic interactions that will be present or absent according to the degree of protonation of carboxylate groups.

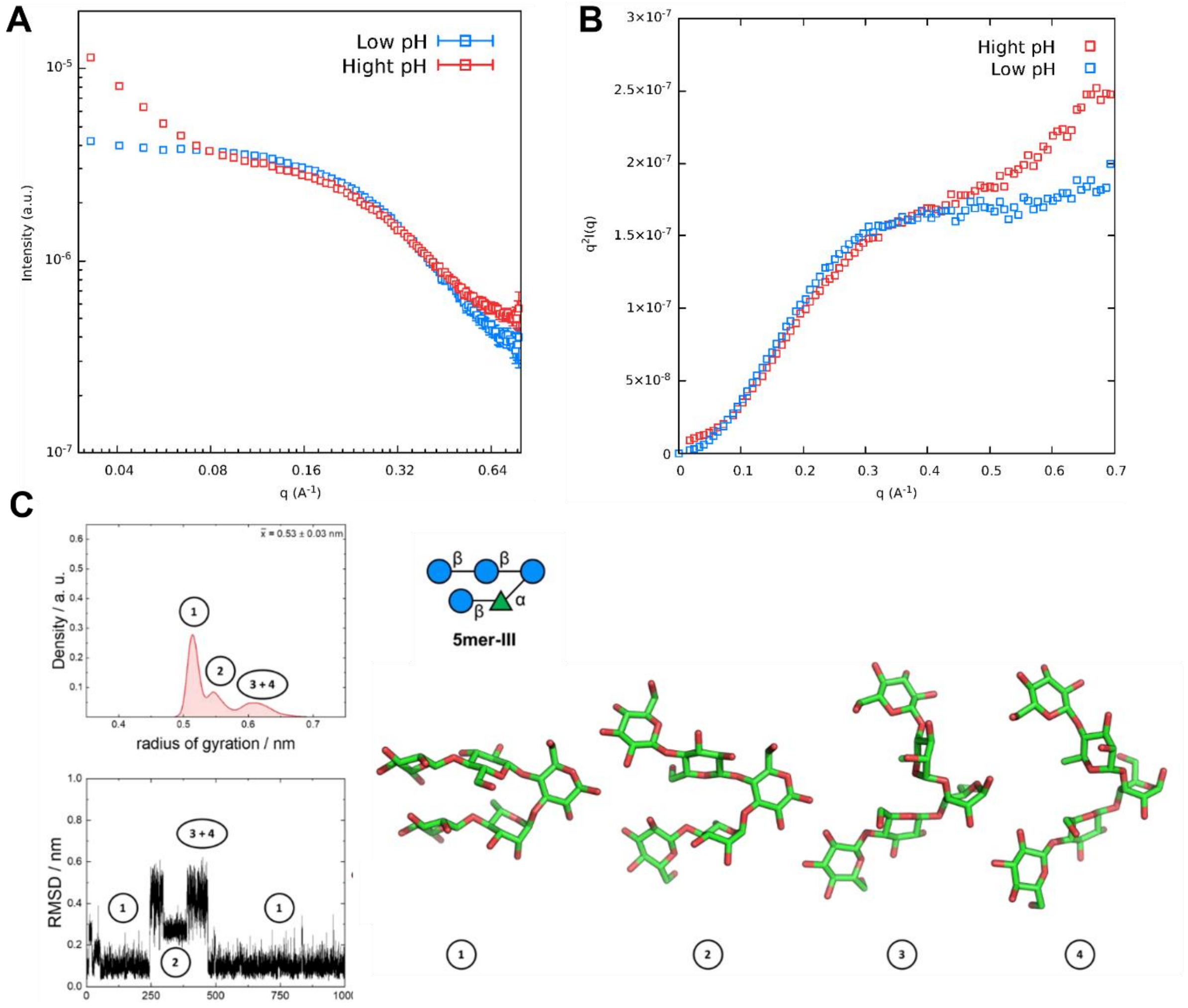

Figure S65

A. Small-angle X-ray scattering intensity plots of hairpin **5mer-III-di-CO_2^-** (10 mg/mL) at pH 2.9 (Low pH) and 11 (High pH). **B.** Kratky plots of hairpin **5mer-III-di-CO_2^-** (10 mg/mL) in both conditions. **C.** Predicted radius of gyration for different conformation of related neutral hairpin **5mer-III** using 1 μs MD simulation.

SAXS intensity curves at pH lower than 3 are characterized for a pristine tendency along y-axis at low-q region (Figure S65 A). This observation agrees with a monodispersed oligosaccharide solution where the Guinier regime is normally observed. The radius of gyration of dispersed glycan hairpin in this pH conditions is analyzed from the low-q region using Guinier law, $I(q) = I_0 \exp(-q^2 R_g^2/3)$, where I_0 is the zero-angle scattering at $q = 0$. The Guinier plots were fitted with linear functions as shown in (Figure S66) to estimate the radius of gyration, which yields 5.46 Å. Consequently, the radius of gyration of the fully protonated glycan hairpin **5mer-III-di-CO_2^-** is comparable with the radius of gyration predicted for a folded conformation of the neutral glycan hairpin

5mer-III (5.20 Å) using MD simulations, while being considerably smaller than the radius of gyration predicted for the most extended conformations (6.20 Å) (Figure S65 C). This observation is on accordance with the spatial proximity between the D-Glc residues of both non-reducing ends determined by NMR and are an indication that, as expected, it predominates attractive intramolecular hydrophobic interactions and van der Waals forces.

In the case of pH 11 the scattering intensity curve is characterized by two dominant features that represent two different length scales in solution of the samples (Figure S65 A). The low-q region ($q < 0.07$ Å^{-1}) show an asymptotic growth of the scattering cure along y-axis that it corresponds with large-size clusters, whereas the high-q regimen ($q > 0.07$ Å^{-1}) arise from the scattering of the monodisperse glycan hairpins similar to the behavior observed at acidic pH. The appropriate concentration for SAXS analysis of the glycan hairpins in both conditions is 1 % wt. Therefore, although no signs of aggregation are observed under the conditions in which the NMR experiments were carried out, at a concentration five timed higher used for SAXS samples it is not surprising that cluster appear in colloidal suspension. The aggregation of this glycan hairpin at basic pH but not at acidic pH agrees with some reports where it was suggested that formation of structures exhibiting strong clustering between chains of acidic polysaccharides required both hydrogen bonding and monovalent ion involvement. The differences between the conformations that the glycan hairpin adopts in both conditions when they are monodispersing can be studied qualitatively by comparing the high-q region of the scattering curve. In the Kratky plot these differences are more evident since the curve obtained in basic pH shows a constant increase along the y-axis in this region which corresponds to more flexible and extended conformation as a result of electrostatic repulsion present in the deprotonated molecule (Figure S65 B).

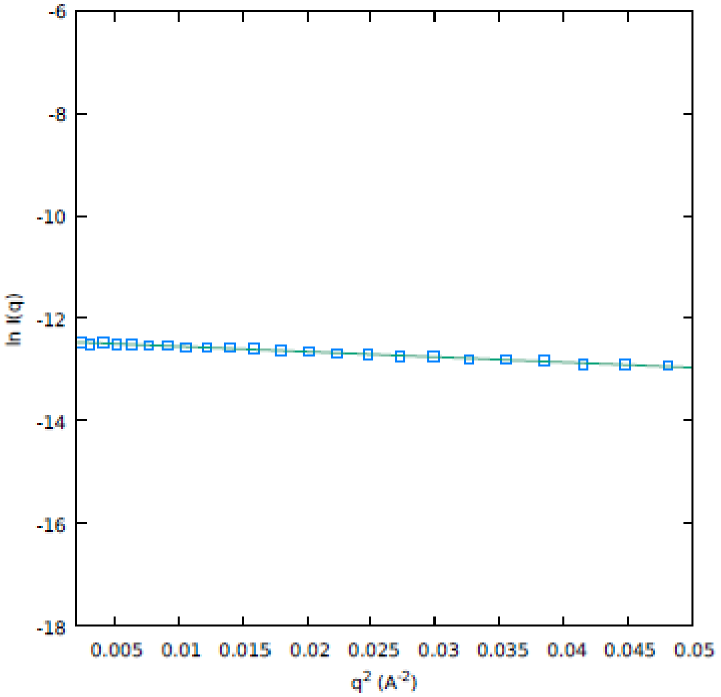

Figure S66
Natural logarithm of I(q) vs. Q^2 plots of Guinier law-SAXS measurements of **5mer-III-di-CO_2^-.**

4.5 NMR characterization of 5mer-III-di-NH_3^+

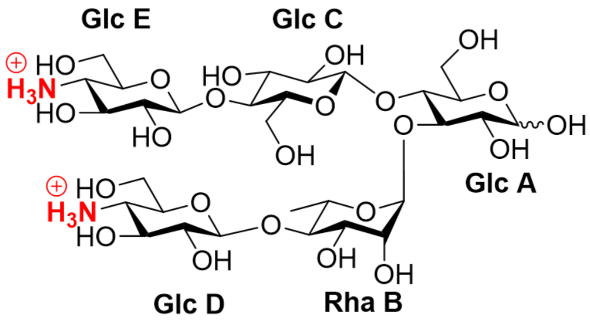

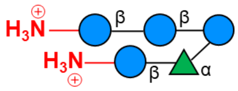

5mer-III-di-NH_3^+

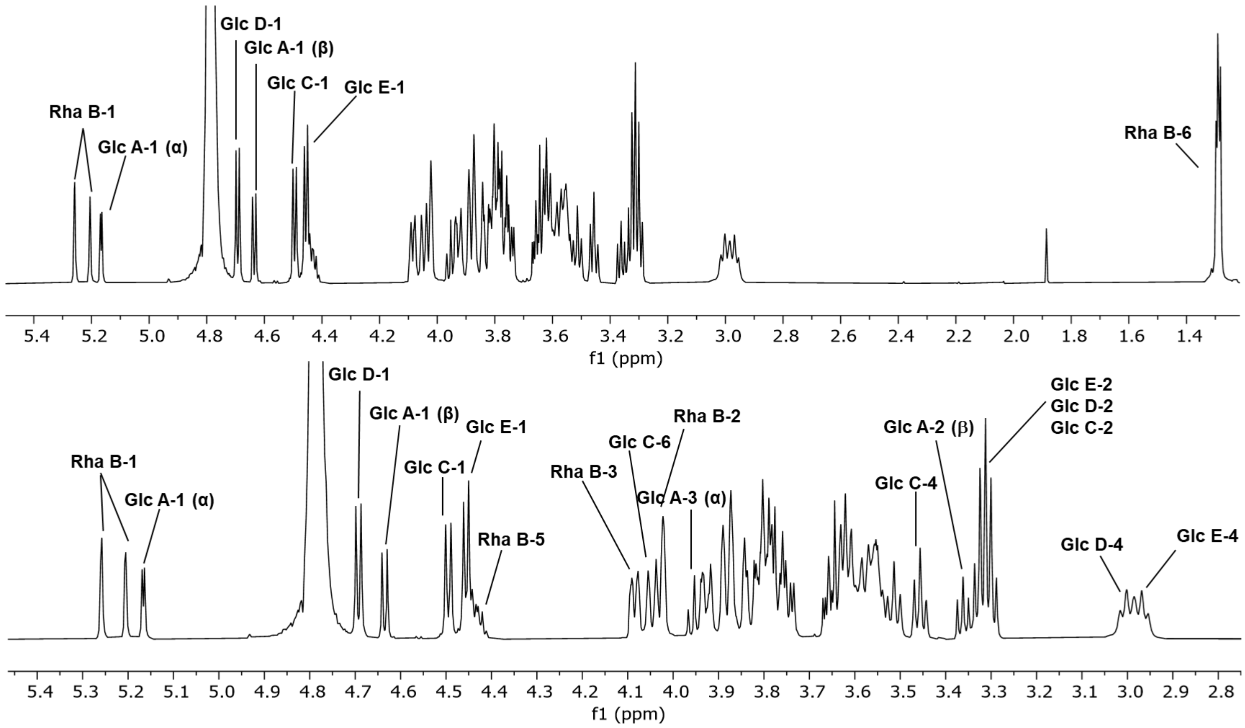

Figure S67
^{1}H NMR (700 MHz, D_2O) of **5mer-III-di-NH_3^+** with assignments.

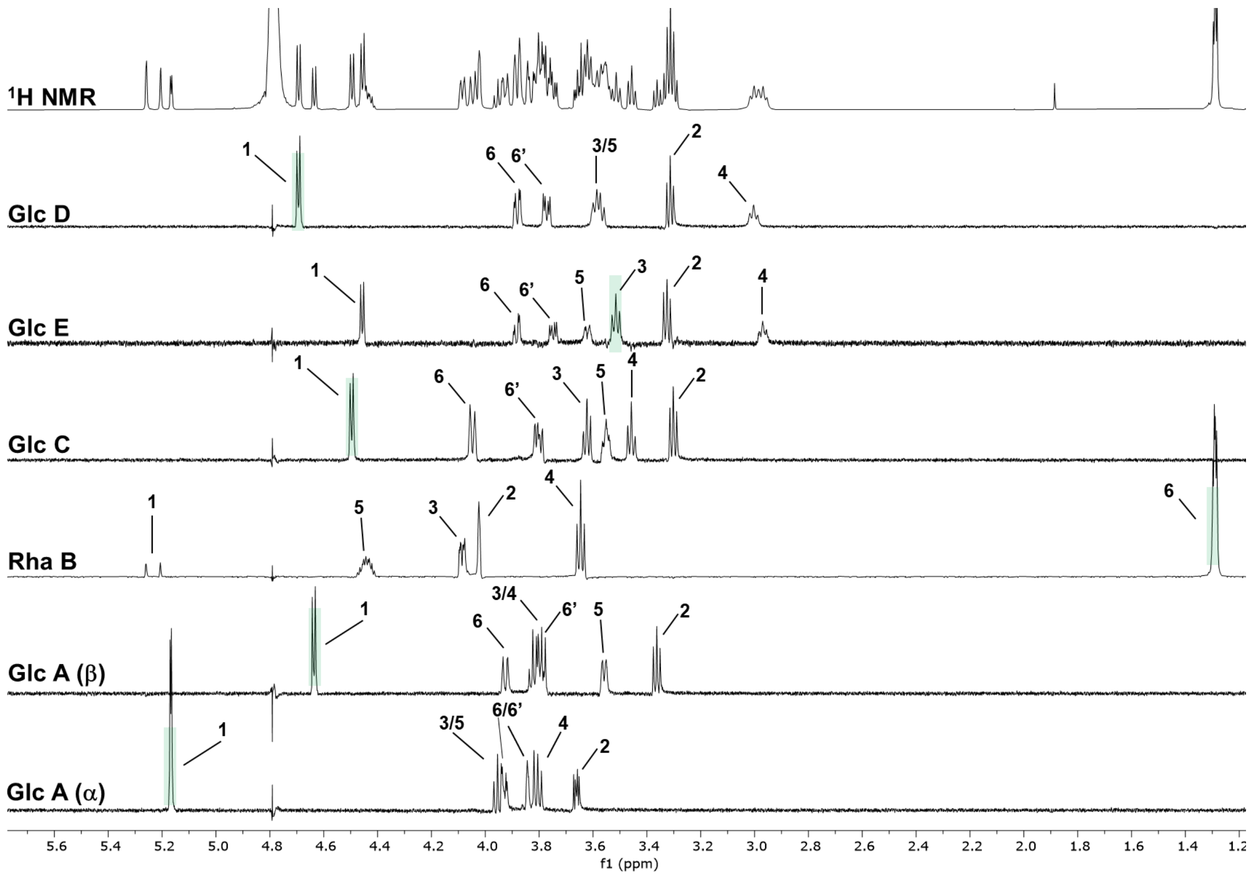

Figure S68
1D TOCSY (700 MHz, d9 200 ms, D_2O) of **5mer-III-di-NH_3^+** with assignments. Resonances chosen for selective excitation are highlighted with green boxes.

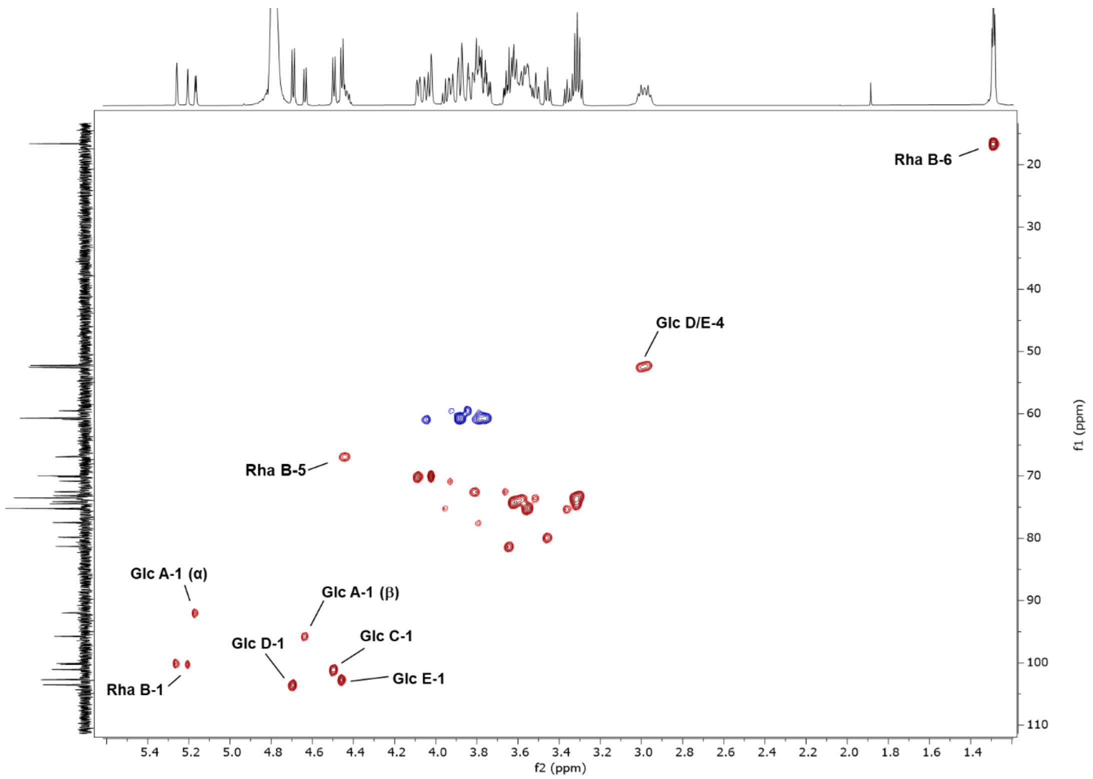

Figure S69
HSQC NMR (D_2O) of **5mer-III-di-NH$_3^+$** with assignments.

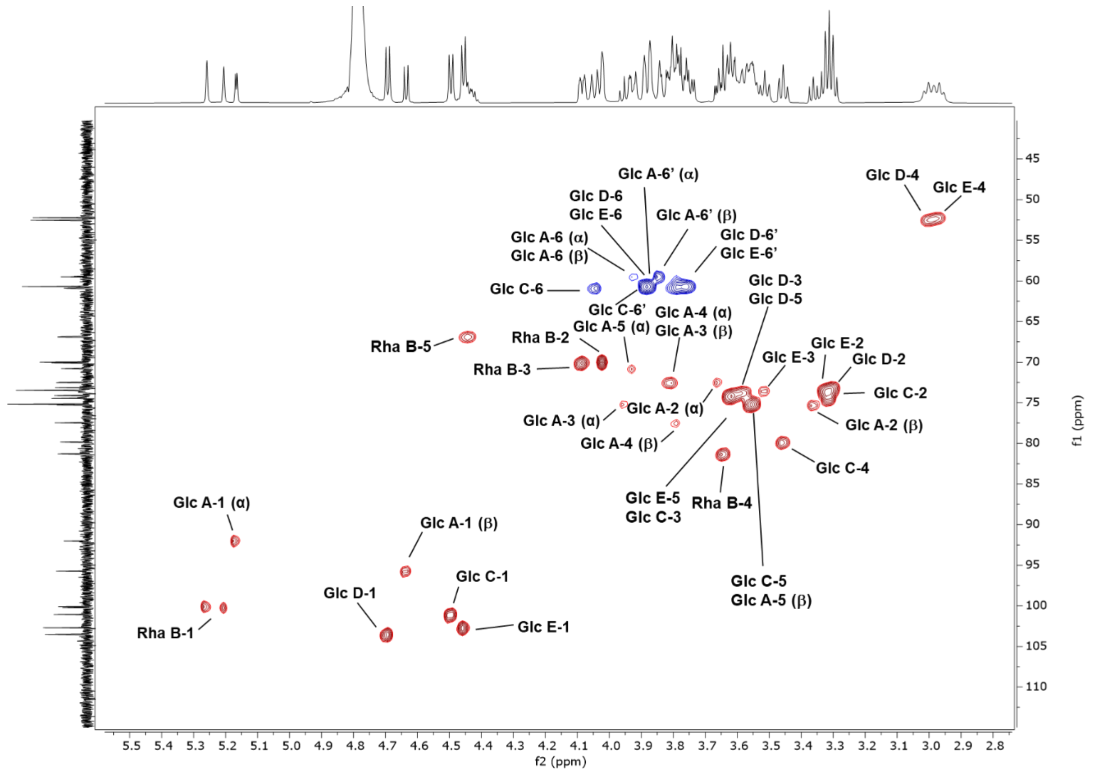

Figure S70
Excerpt of HSQC NMR (D_2O) of **5mer-III-di-NH$_3^+$** with assignments.

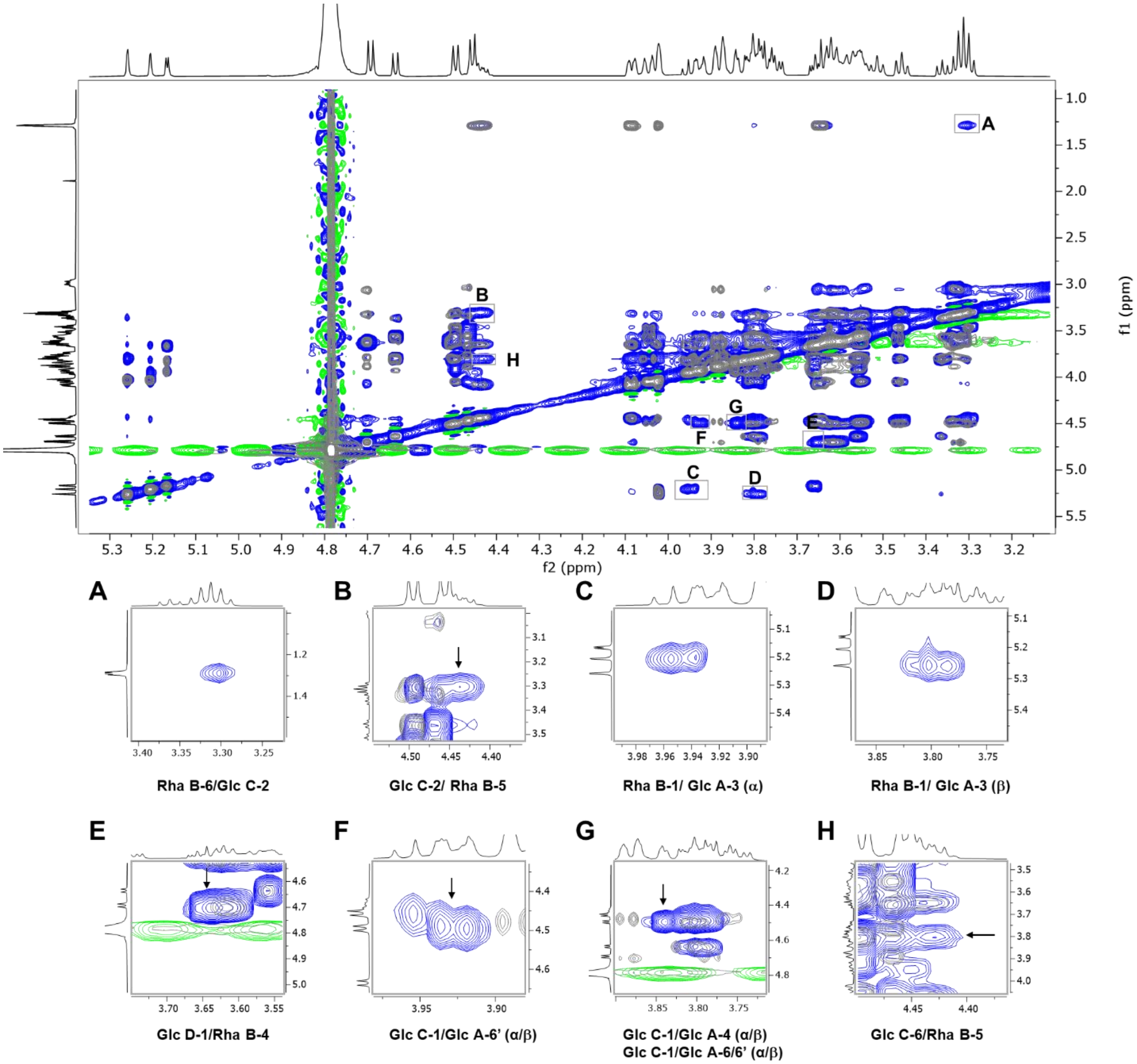

Figure S71
Superimposed 2D NOESY (green-blue, 700 MHz, d8 800 ms, D_2O) of **5mer-III-di-NH_3^+** at **pH 6.96** with assignments and 2D TOCSY spectrum (gray, 700 MHz, d9 150 ms, D_2O).

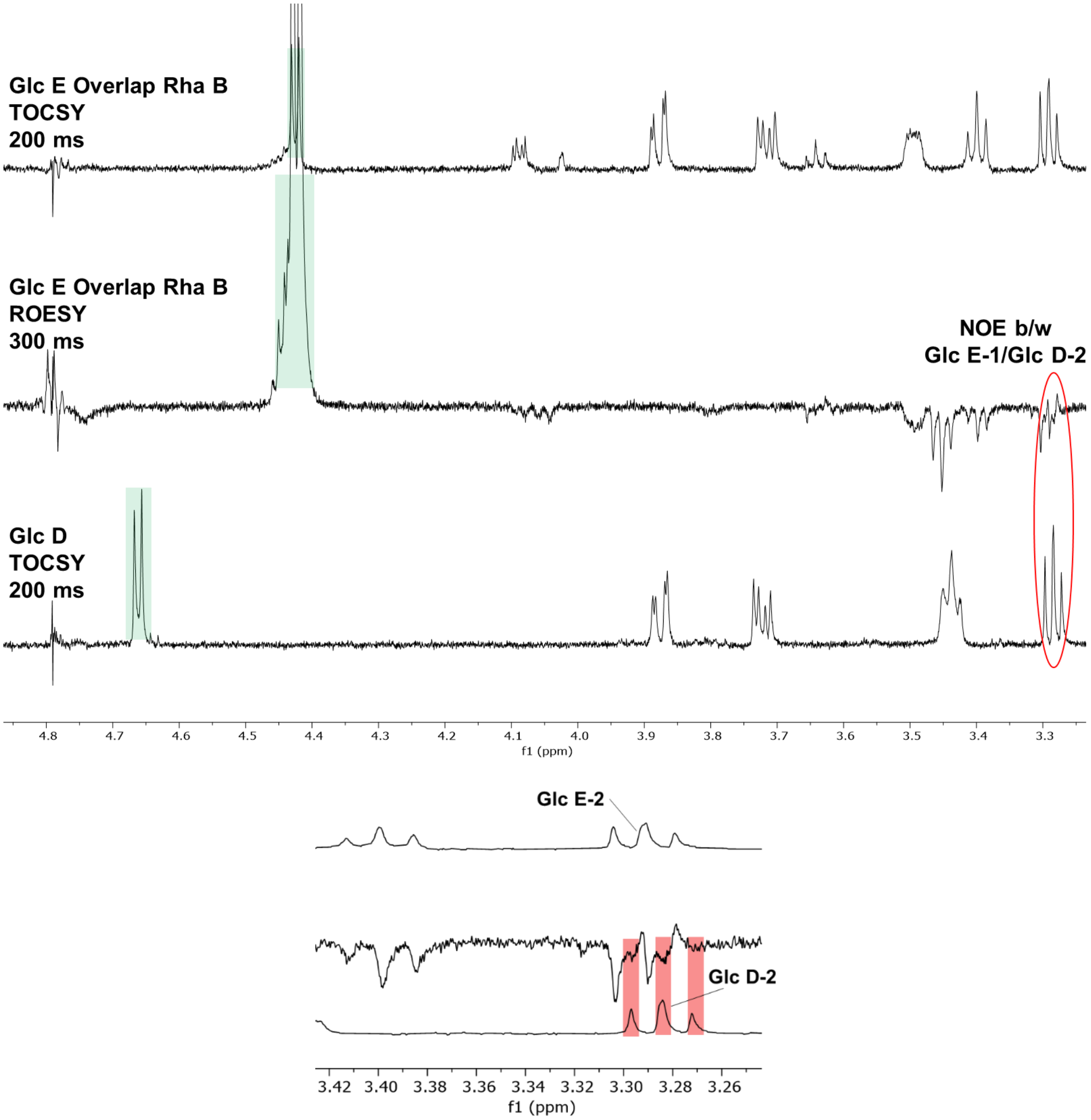

Figure S72
Overlay of 1D ROESY (700 MHz, p15 300 ms, D_2O) and 1D TOCSY (700 MHz, d9 200 ms, D_2O) of **5mer-III-di-NH_3^+**. Key NOE signal between Glc E-1/Glc D-2 (highlighted with red box and red circle) was observed at **pH 6.96**.

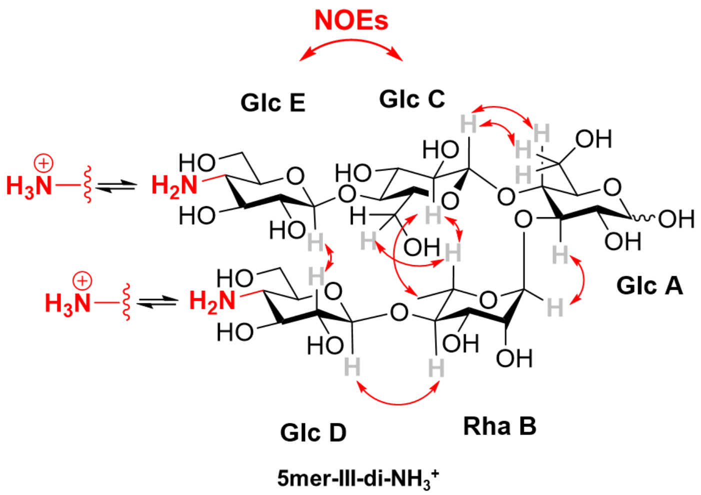

Figure S73
All experimentally observed NOEs (red arrows) of **5mer-III-di-NH_3^+** at **pH 6.96**.

4.5.1 pH titration of 5mer-III-di-NH_3^+

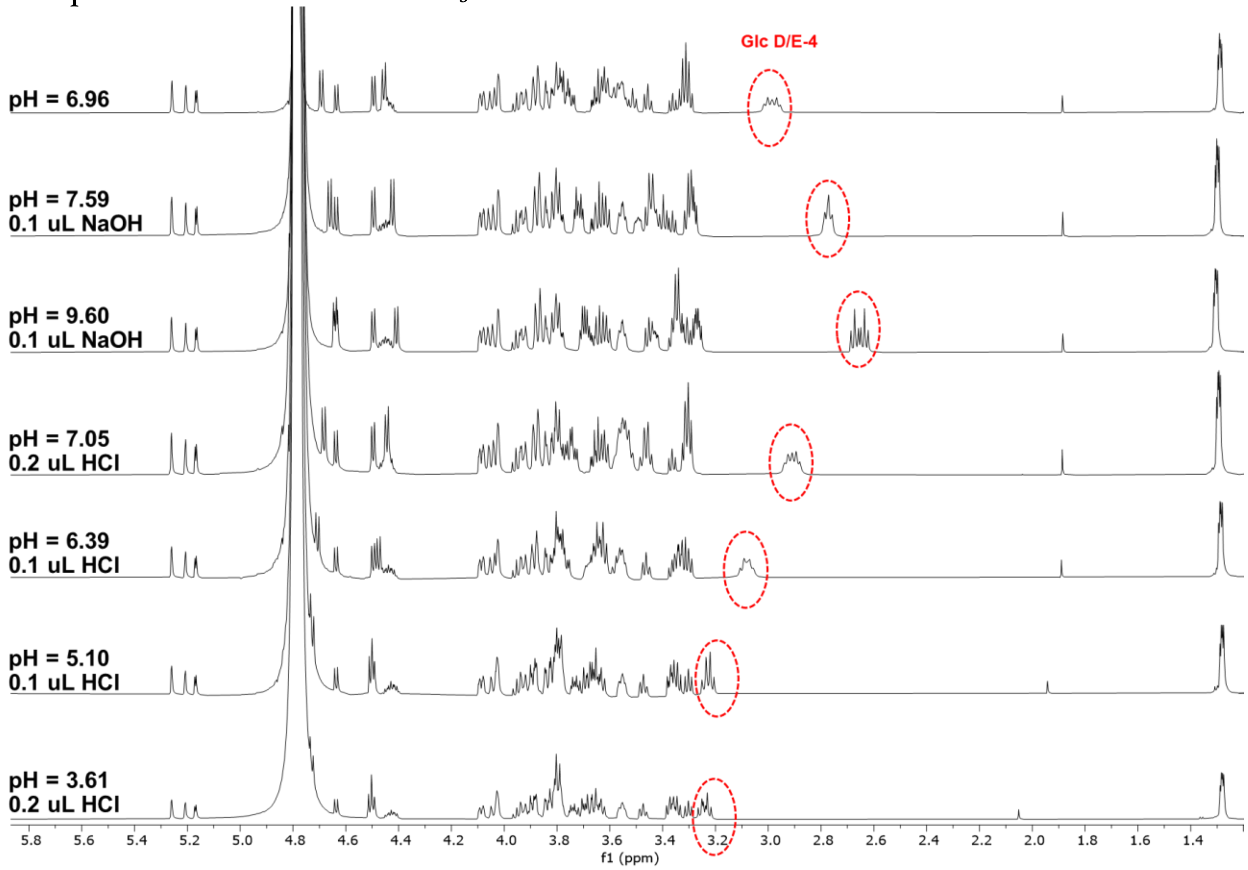

Figure S74

^1H NMR (700 MHz, D_2O) titration of **5mer-III-di-NH_3^+.** pH of the sample was adjusted using 1M HCl and 1M NaOH solutions. The shift of selected signals during the titration is highlighted with: Red circle (Glc D/E-4).

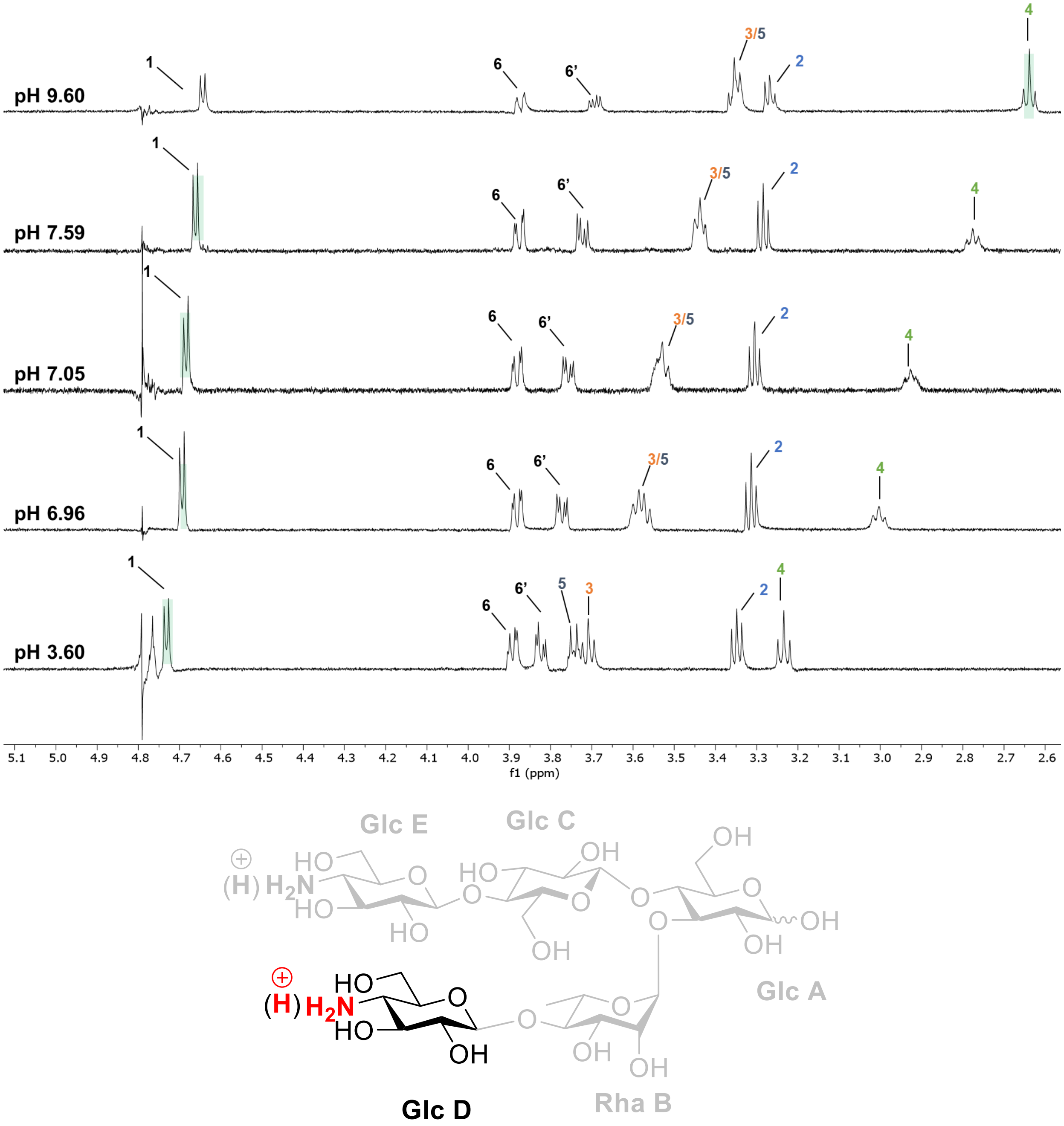

Figure S75
Selective 1D TOCSY (700 MHz, d9 200 ms, D_2O) spectra of **Glc D** of **5mer-III-di-NH_3^+** with assignments showing the peak shifts at different pH. Resonances chosen for selective excitation are highlighted with green boxes.

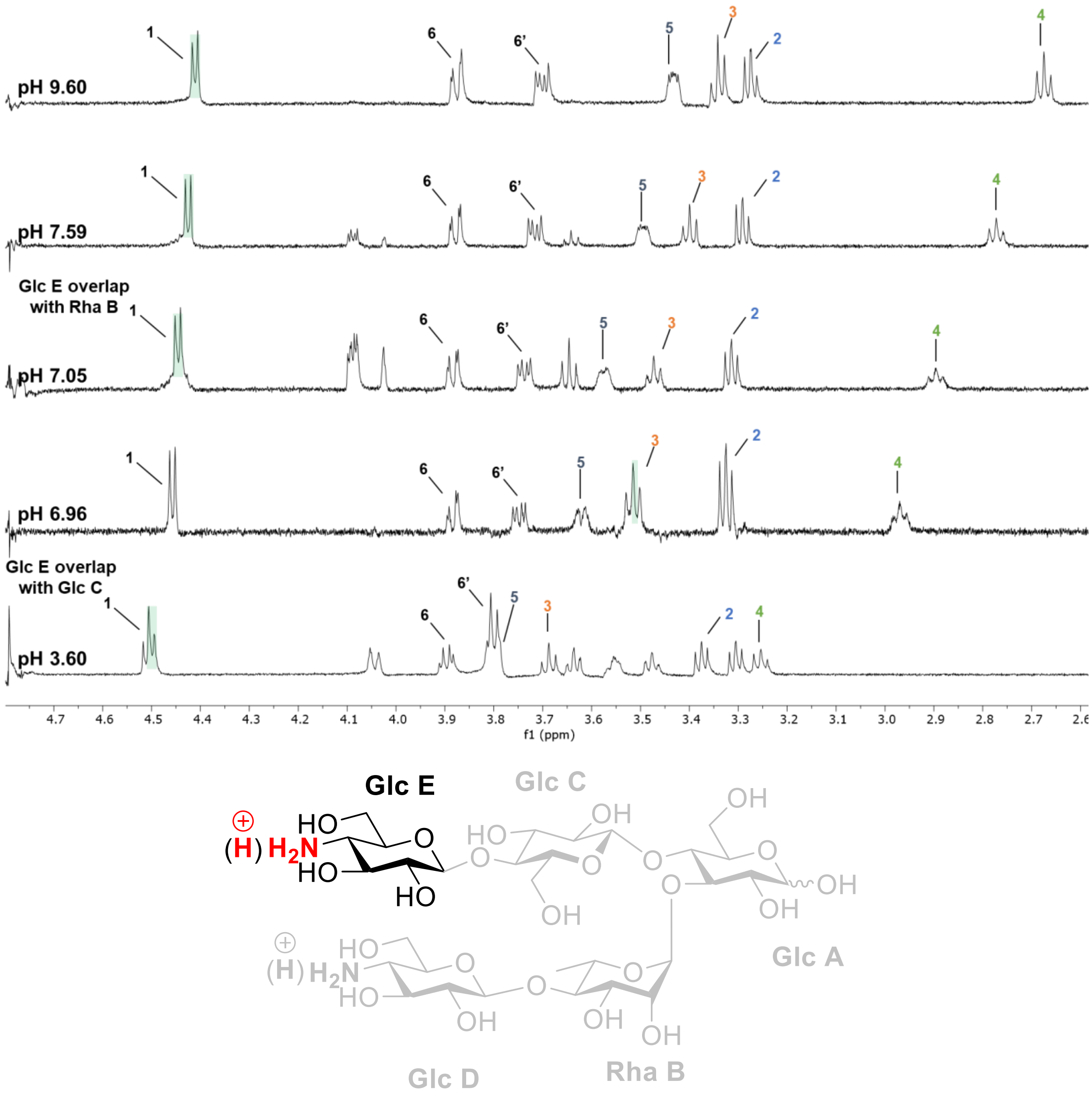

Figure S76

Selective 1D TOCSY (700 MHz, d9 200 ms, D_2O) spectra of **Glc E** of **5mer-III-di-NH$_3^+$** (**Glc E-1** overlaps with **Glc C-1** at low pH and with **Rha B-5** at neutral pH) with assignments of **Glc E** showing the peak shifts at different pH. Resonances chosen for selective excitation are highlighted with green boxes.

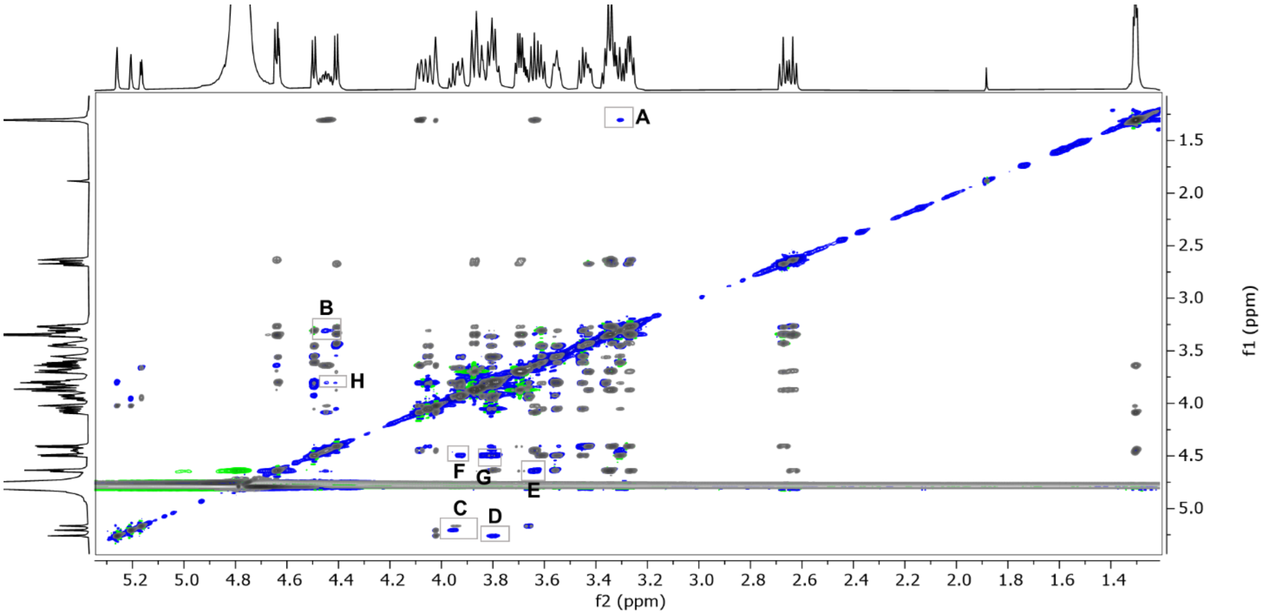

Figure S77
Superimposed 2D NOESY (green-blue, 700 MHz, d8 800 ms, D_2O) of **5mer-III-di-NH_3^+** at **pH 9.60** with assignments and 2D TOCSY spectrum (gray, 700 MHz, d9 150 ms, D_2O). The pH was adjusted from **6.96** to **9.60** by adding 0.3 uL of a 1 M NaOH solution. At **pH 9.60**, all the NOEs observed at **pH 6.96** were detected (**Figure S71**).

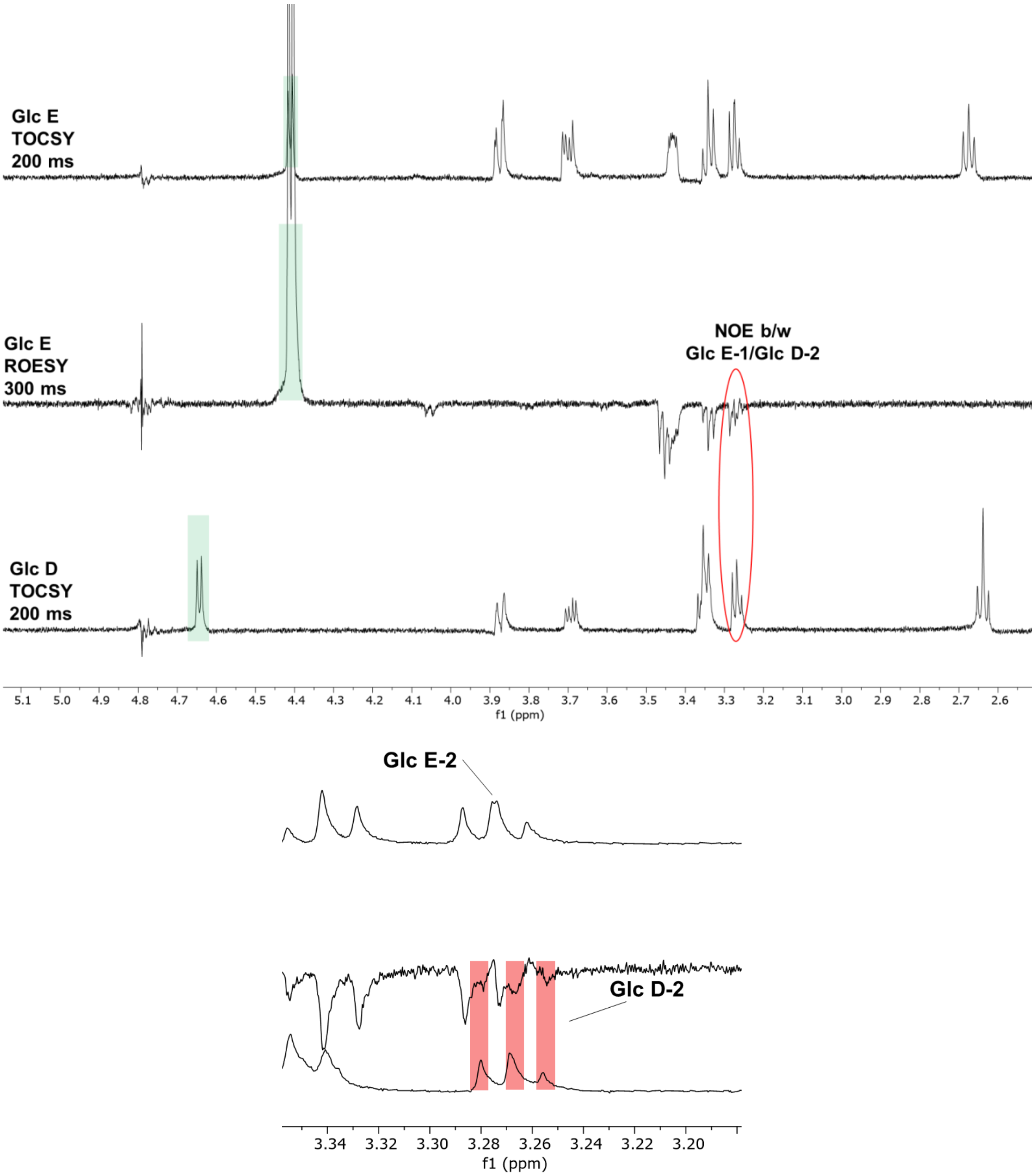

Figure S78
Overlay of 1D ROESY (700 MHz, p15 300 ms, D_2O) and 1D TOCSY (700 MHz, d9 200 ms, D_2O) of **5mer-III-di-NH_3^+**. Key NOE signal between Glc E-1/Glc D-2 (highlighted with red box and red circle) was observed at **pH 9.60**.

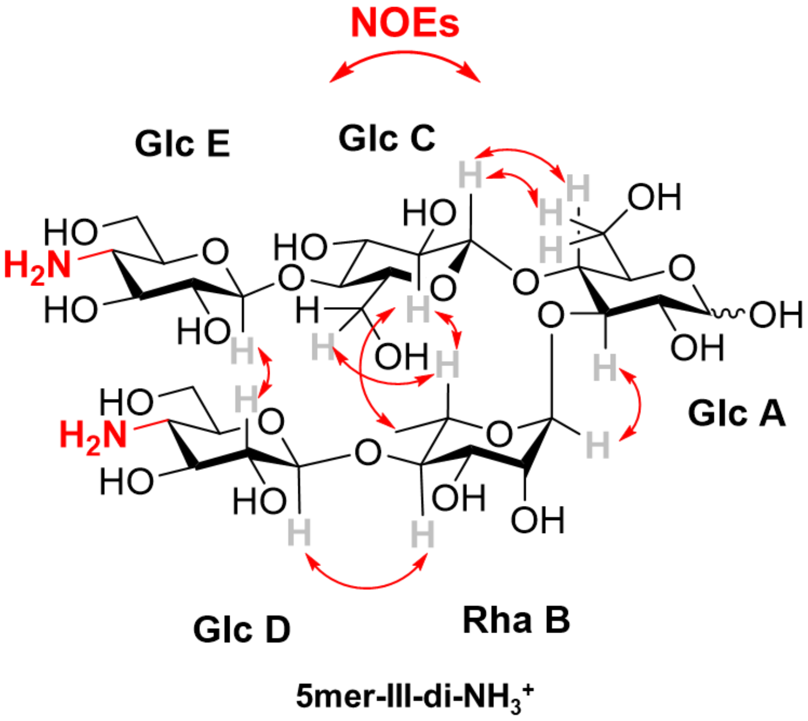

Figure S79
All experimentally observed NOEs (red arrows) of **5mer-III-di-NH$_3^+$**at **pH 9.60**

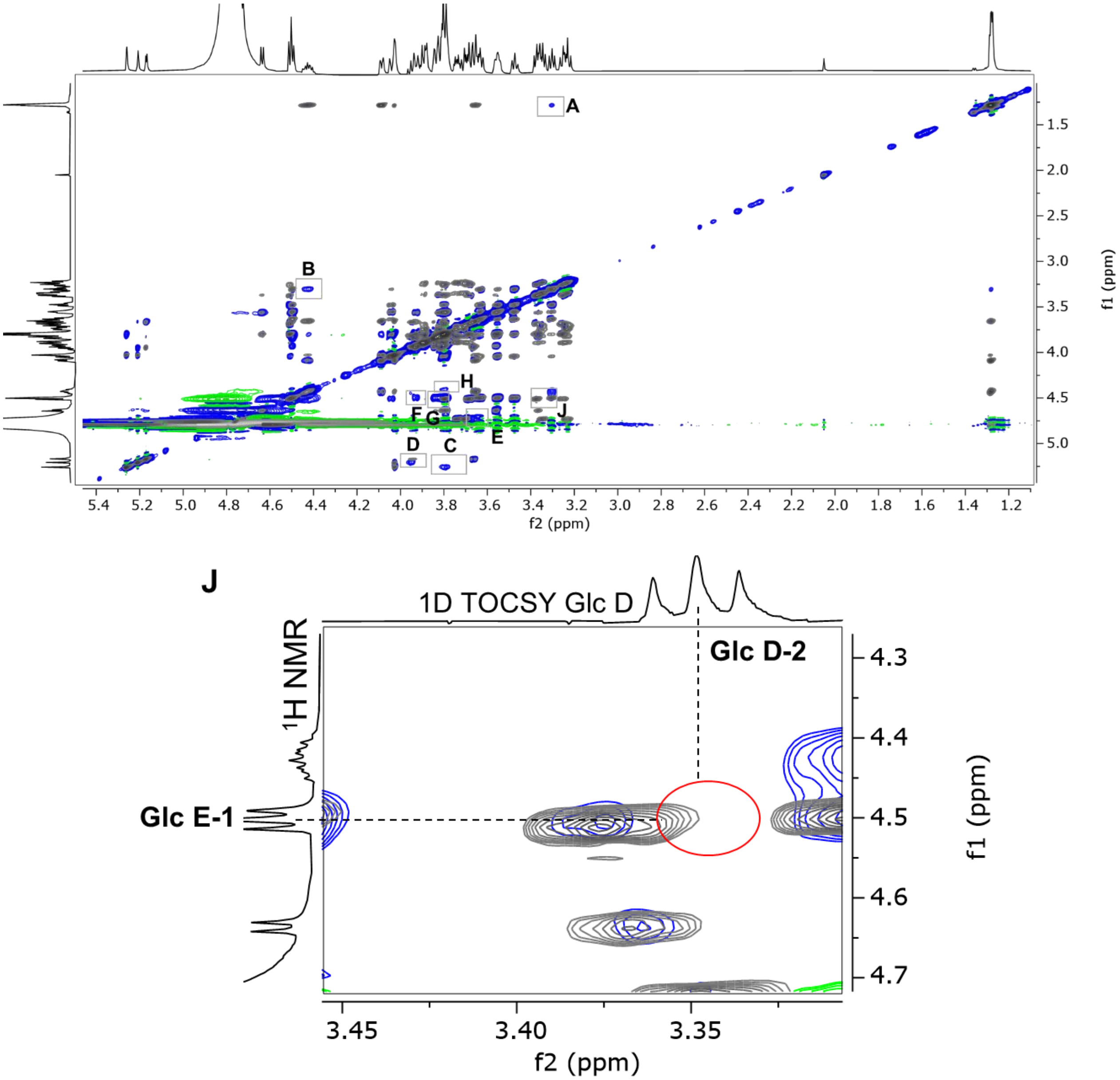

Figure S80
Overimposed 2D NOESY (green-blue, 700 MHz, d8 800 ms, D_2O) of **5mer-III-di-NH$_3^+$** at **pH 3.60** with assignments and 2D TOCSY spectrum (gray, 700 MHz, d9 150 ms, D_2O). The pH was adjusted from **5.10** to **3.61** by adding 0.1 uL of a 1 M HCl solution. At **pH 3.60**, all the NOEs observed at **pH 6.96** were detected (**Figure S71**) except for the inter-strand NOE signal between the two strands Glc E-1/Glc D-2 (**J**).

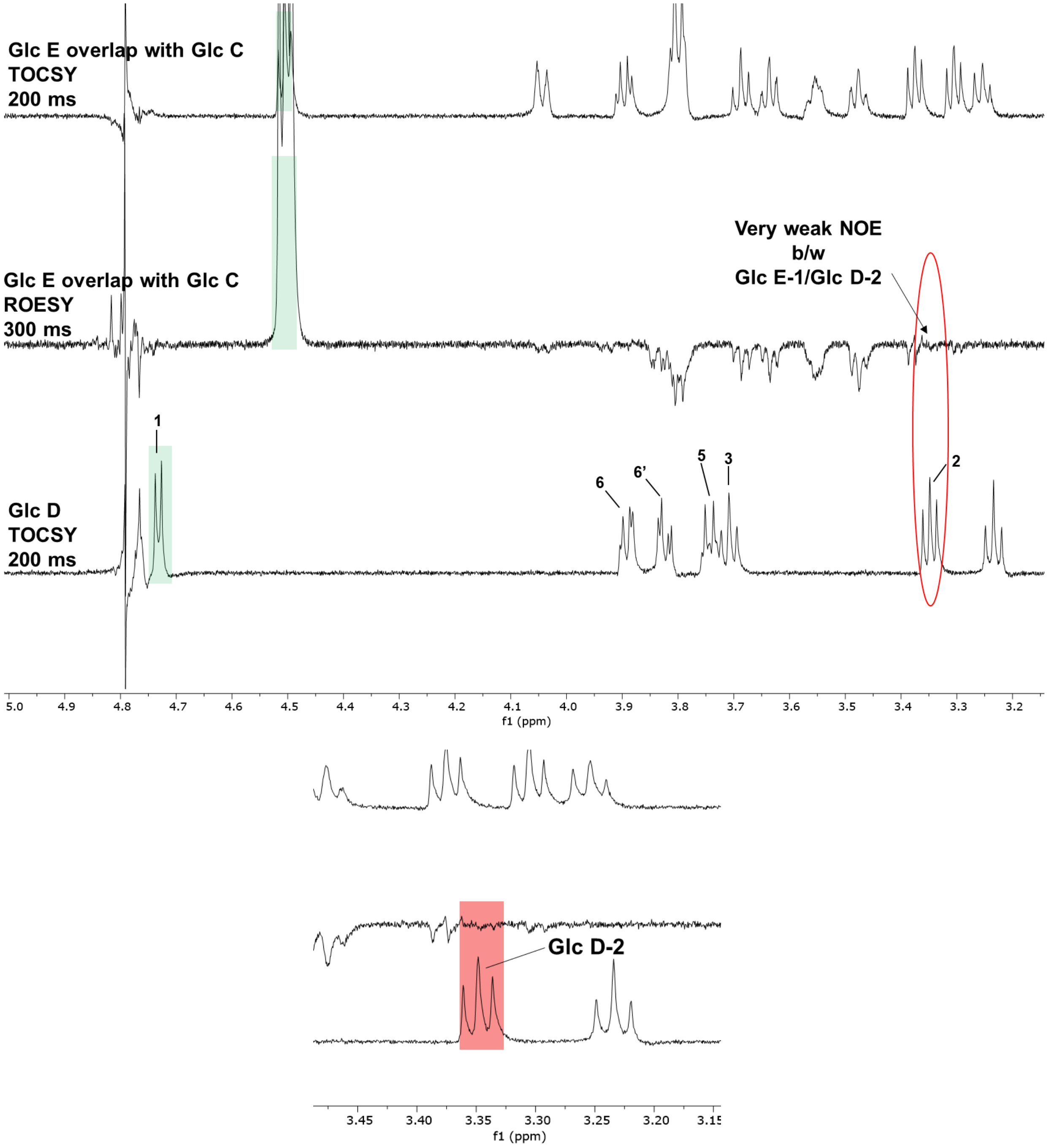

Figure S81

Overlay of 1D ROESY (700 MHz, p15 300 ms, D_2O) and 1D TOCSY (700 MHz, d9 200 ms, D_2O) of **5mer-III-di-NH_3^+**. Very weak key NOE signal between Glc E-1/Glc D-2 (highlighted with red box and red circle) was observed at **pH 3.61**.

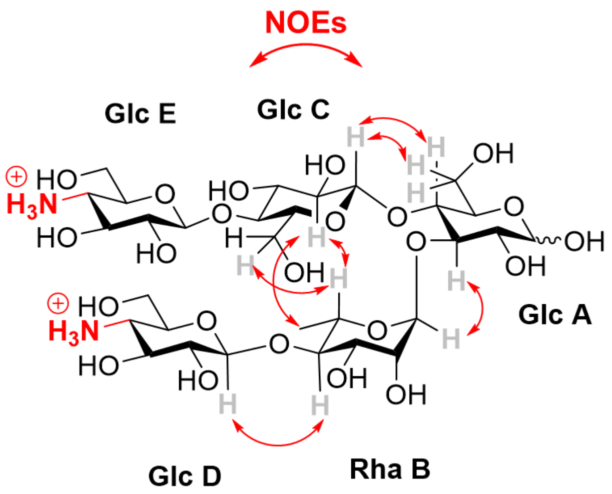

5mer-III-di-NH$_3^+$

Figure S82
All experimentally observed NOEs (red arrows) of **5mer-III-di-NH$_3^+$** at **pH 3.60**.

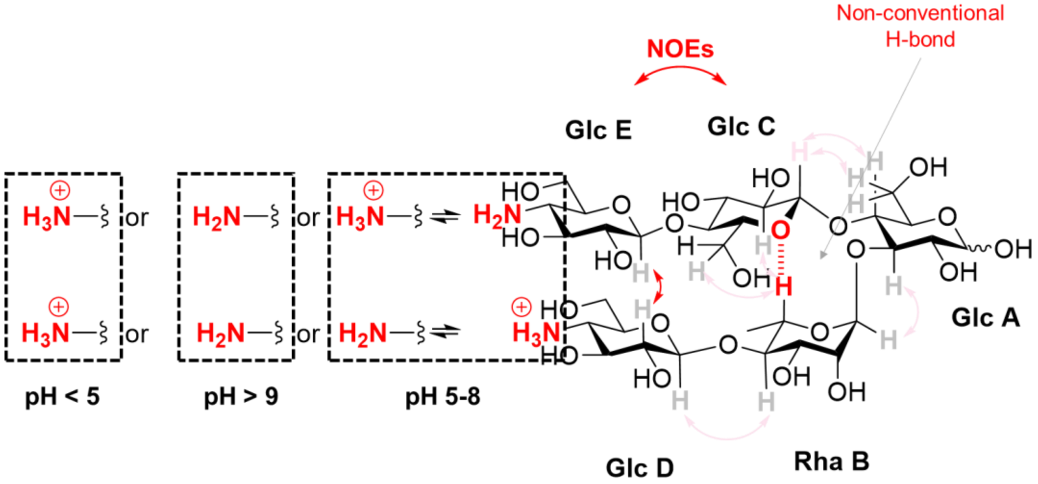

5mer-III-di-NH$_3^+$

Figure S83
Chemical structure representing different de(protonation) states of **5mer-III-di-NH$_3^+$**.

pH	Glc E-1/GlcD-2 NOE signal	δ Rha B-5 (ppm)
**9.60**	Yes	4.45
**7.59**	Yes	4.45
**6.96**	Yes (weak)	4.44
**3.60**	No	4.42

Table S03
Overall analysis of **5mer-III-di-NH$_3^+$** at different pH. Inter-strand NOE signal Glc E-1/Glc D-2 and Rha B-5 chemical shifts are shown.

4.6 pH titration of 5mer-III

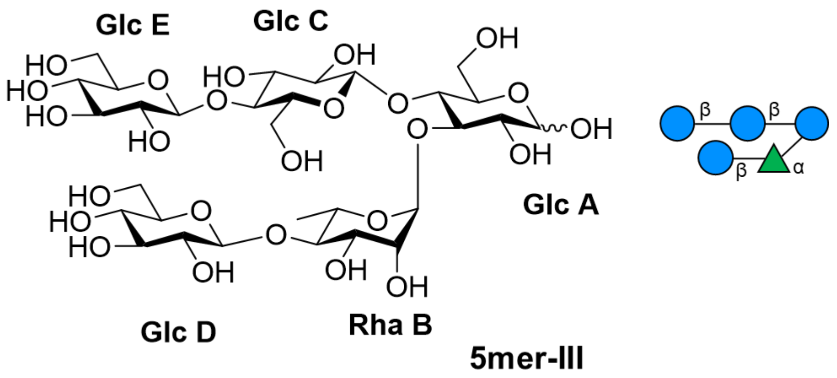

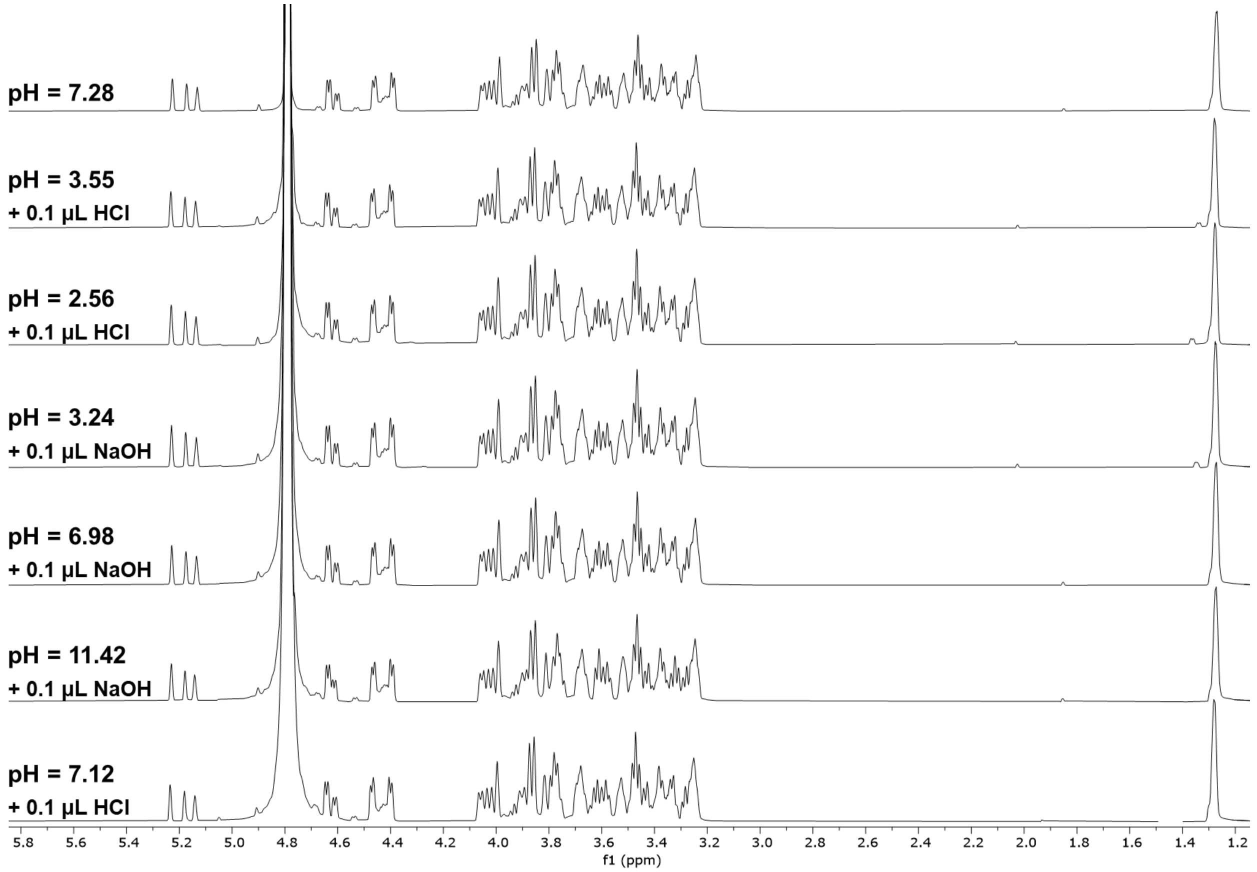

Figure S84
^{1}H NMR (700 MHz, D_2O) titration of **5mer-III**. pH was adjusted using 1M HCl and 1M NaOH solutions. Control experiments showing that **5mer-III** (lacking ionic functional groups) is not affected by pH.

4.7 Evaluation of the non-conventional H-bond at neutral pH for all 5mer

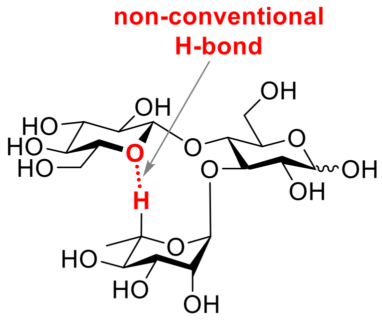

3mer-III

Compound	δ Rha B-5 (ppm)	Δδ (ppm)*
**5mer-III**	4.43	0.37
**5mer-III-di-SO_3^-**	4.41	0.35
**5mer-III-zwi**	4.43	0.37
**5mer-III-di-CO_2^-**	4.41	0.35
**5mer-III-di-NH_3^+**	4.44	0.38
**5mer-III-di-PO_3^{2-}**	4.39	0.33
**3mer-V**	4.06	0

Table S04
Chemical shifts of Rha-5 extracted from 2D HSQC spectra at neutral pH for all the compounds synthesized in this work. The results are compared with the **3mer-V** (lacking the non-conventional H-bond) synthesized in previous work.[3] ***Δδ** was calculated as follows:

$$\Delta\delta = \delta_{(Rha-5,\boldsymbol{compound})} - \delta_{(Rha-5,\boldsymbol{3me-V})}$$

The chemical shift of Rha-5 for all 5mers show a substantial downfield shift (Δδ ≈ 0.3-0.4 ppm) when compared to **3mer-V**, indicating the presence of the non-conventional H-bond in the turn unit.[13a, 13b] Still, Rha B-5 in all the 5mer with the same ionic groups on the hairpin strands show a upfield shift suggesting the weakening of the non-conventional H-bond due to electrostatic repulsion. In contrast, the downfield shift of Rha B-5 of **5mer-III-zwi** indicates a closer hairpin conformation due to the formation of an ionic bridge.

4.8 STEP-NOESY/t-ROESY inter-residue distance estimation

To determine the distance between turn residues Rha B and Glc C, NOEs from Rha B-5 were estimated. As this proton shows overlap in all the structures, the STEP-NOESY/t-ROESY strategy was employed. Isotropic mixing from Rha B-6 was optimized to obtain the highest signal at Rha B-5 at the possible lower mixing time, which was 40ms. Selective irradiation of Rha B-6 followed by TOCSY transfer of magnetization to Rha B-5 and then NOESY/t-ROESY from Rha B-5 permitted us to measure the intra-residue Rha B-5/Rha B-3 (as reference) and the inter-residue Rha B-5/Glc C-2 NOEs/ROEs for all 5mers. To determine the distance between strand residues Glc D and Glc E, NOEs from Glc D-2 were measured. Selective irradiation of Glc D-1 followed by TOCSY transfer of magnetization to Glc D-2 and then NOESY/t-ROESY from Glc D-2 permitted us to measure the intra-residue Glc D-2/Glc D-4 (as reference) and the inter-residue Glc D-2/Glc E-1 NOEs/ROEs for all 5mers.The results are shown in Figure S84-87. The intra-residue distance calculations for the corresponding OMe derivatives obtained with Maestro Software:

1. Minimization with MacroModel (OPLS4 force field, water)
2. Energy Optimization with Jaguar (B3LYP-D3_6-31G**, DFT, solvent=water_PBF)

	r ref D2-D4	r ref B5-B3
**L-Rha α-OMe**		2.5
**Glc-β-Ome_**	2.5	
**Glc-β-Ome_SO_3^-**	2.6	
**Glc-β-Ome_PO_3^{2-}**	2.6	
**Glc-β-Ome_zwi (CH_2-CO_2^-) gg**	2.6	

The equation used to estimate the inter-residue distance is given below; where r_{ij} is estimated distance, r_{ref} is reference distance, V_{ref} is integral of NOE peak between reference proton pair and V_{ij} is integral of NOE peak between target proton pair.

$$r_{ij} = r_{ref} \times \left(\frac{V_{ref}}{V_{ij}}\right)^{1/6}$$

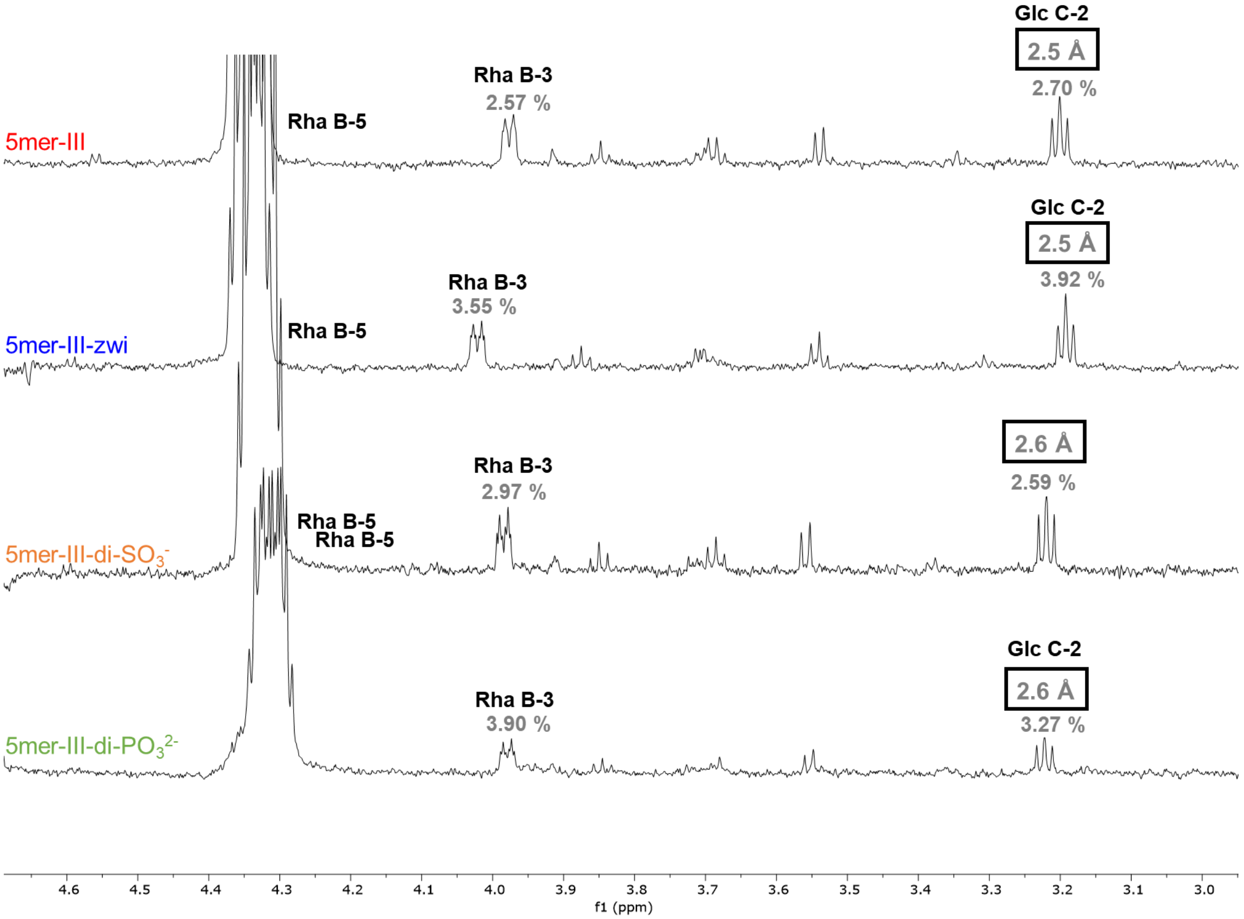

Figure S85
STEP-NOESY (400 ms) experiments to estimate the inter-residue Rha B-5/Glc C-2 NOE distance for **5mer-III**, **5mer-III-zwi**, **5mer-III-di-SO$_3^-$ and 5mer-III-di-PO$_3^{2-}$** (293 K, D_2O, 800 MHz) at neutral pH.

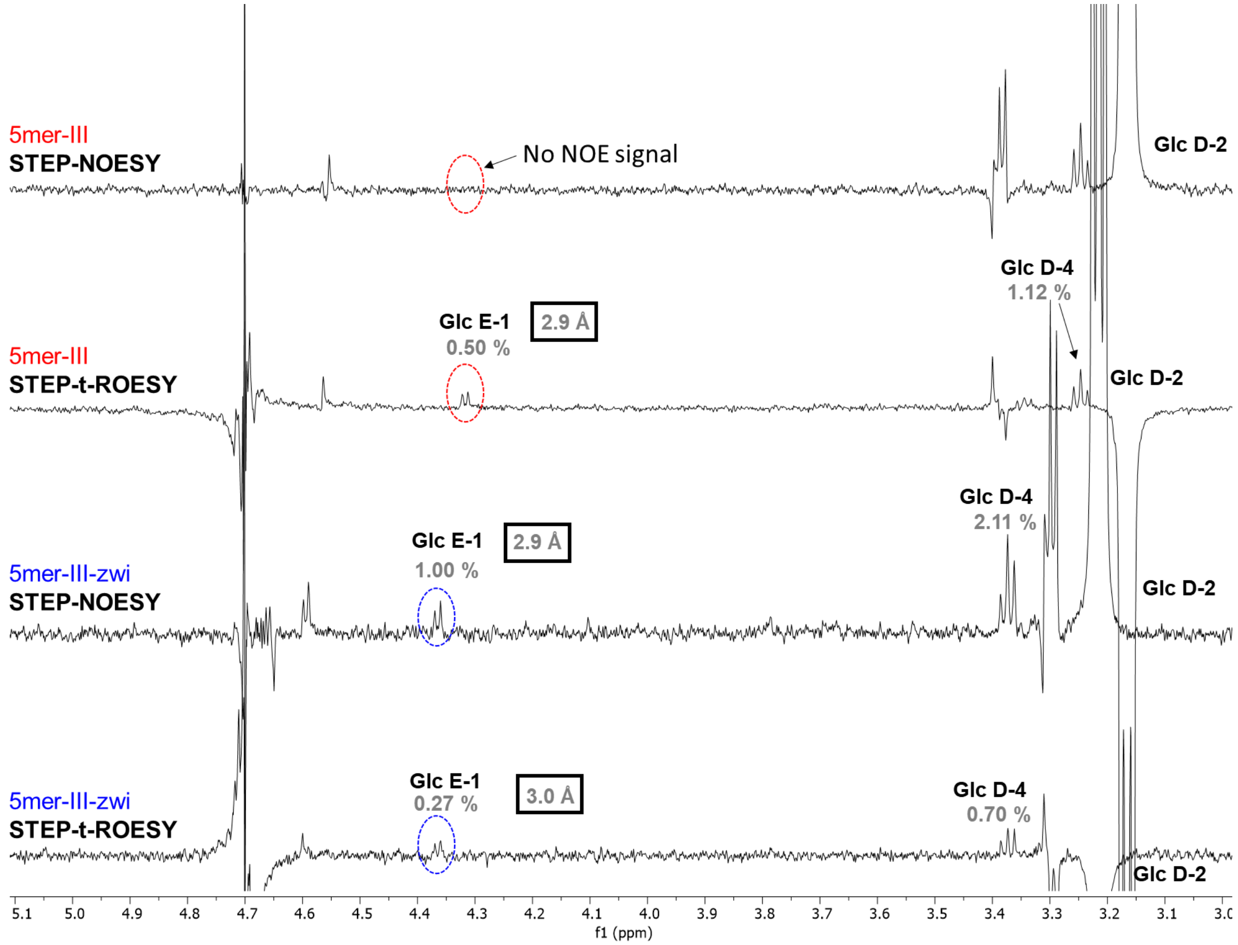

Figure S86
STEP-NOESY (400 ms)/ROESY (200 ms) experiments to estimate the interstrand Glc D-2/Glc E-1 NOE distance for **5mer-III** and **5mer-III-zwi** (293 K, D_2O, 800 MHz) at pH 5.5. No NOE signal was detected in STEP-NOESY experiments in **5mer-III** (red circle) whereas NOE signal detected in both experiments in **5mer-III-zwi** (blue circle).

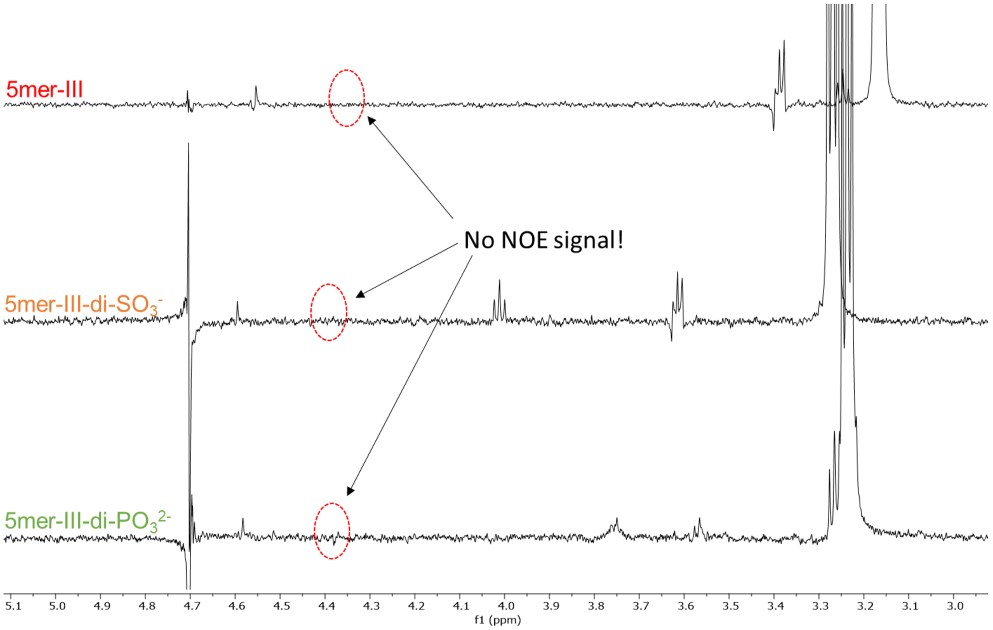

Figure S87
STEP-NOESY (400 ms) experiments to estimate the interstrand Glc D-2/Glc E-1 NOE distance for **5mer-III**, **5mer-III-di-PO$_3^{2-}$ and 5mer-III-di-SO$_3^-$** (293 K, D_2O, 800 MHz). No NOE signal was detected in STEP-NOESY experiments in all the 5mers (red circle). This could be due to longer D2-E1 distances or to NOE zero arising from different local correlation times.

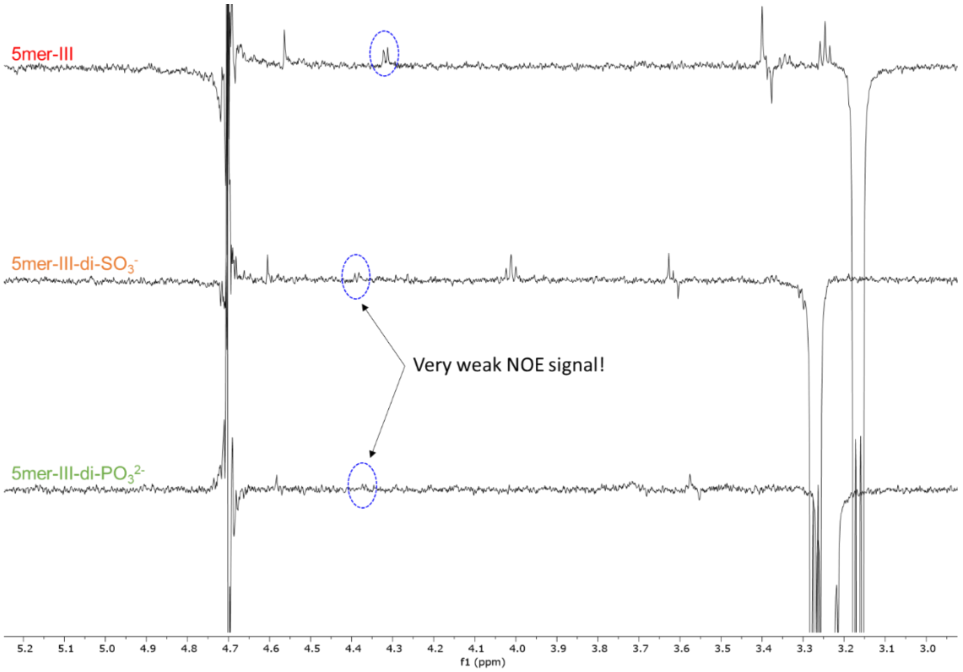

Figure S88
STEP-t-ROESY (200 ms) experiments to estimate the interstrand Glc D-2/Glc E-1 NOE distance for **5mer-III**, **5mer-III-di-PO$_3^{2-}$ and 5mer-III-di-SO$_3^-$** (293 K, D_2O, 800 MHz). NOE signal was detected in STEP-t-ROESY experiments in all the 5mers (blue circle), but the intensities of the peaks were weaker for **5mer-III-di-PO$_3^{2-}$ and 5mer-III-di-SO$_3^-$** in comparison to **5mer-III**, indicating open conformation of both anionic hairpins.

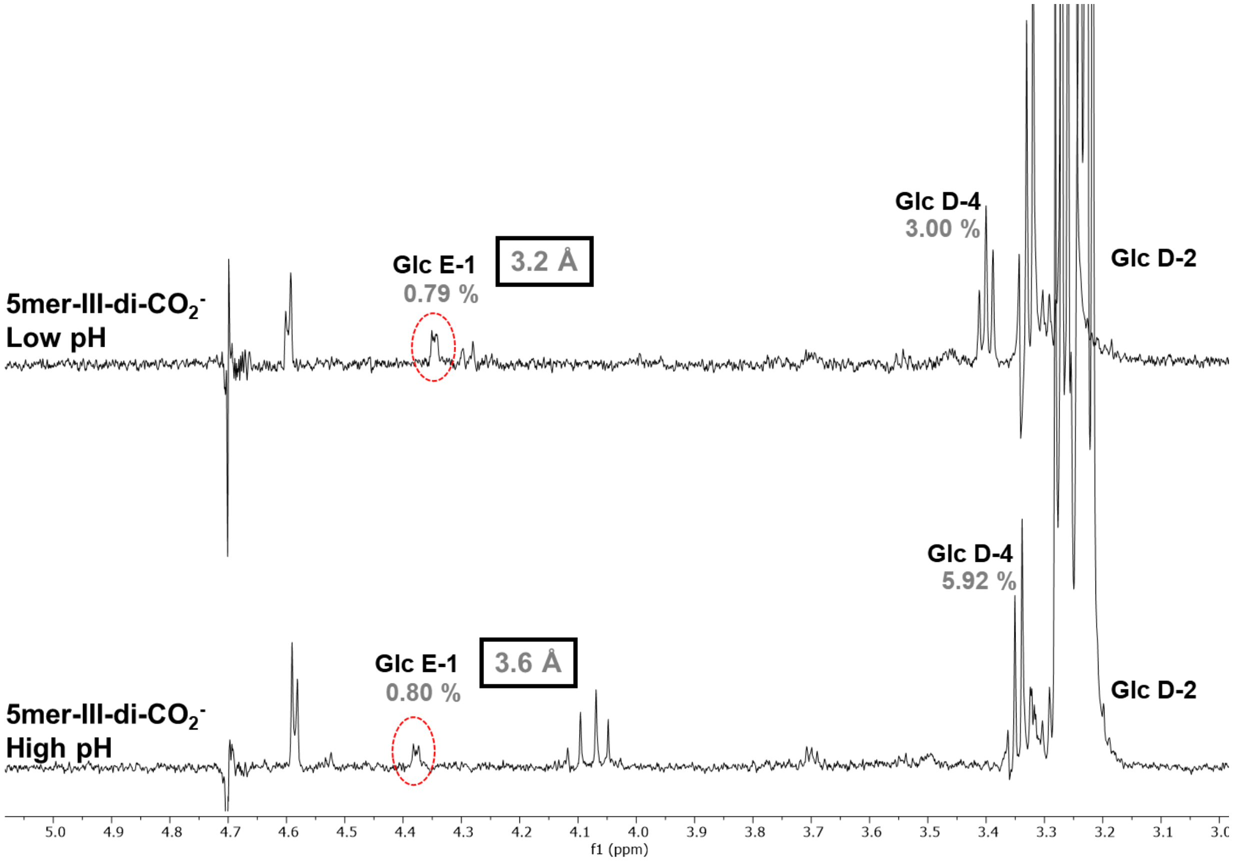

Figure S89
STEP-NOESY (400 ms) experiments to estimate the interstrand Glc D-2/Glc E-1 NOE distance for **5mer-III-di-CO_2^-** (293 K, D_2O, 800 MHz) at low and high pH (293 K, D_2O, 800 MHz).

STEP-t-ROESY[#]	rD2-E1	rB5-C2	STEP-NOESY[14]	rD2-E1	rB5-C2
**5mer-III**	2.9	2.5	1D-t-ROESY	(**),<br>2.9 (***)	2.5
**5mer-III-zwi**	3	2.5		2.95	2.5
**5mer-III-di-SO_3^-**	>3.3 (*)	2.6		(**)	2.6
**5mer-III-di-PO_3^{2-}**	>3.3 (*)	2.6		(**)	2.6
**5mer-III-di-CO_2^- Low pH**				3.2	2.5
**5mer-III-di-CO_2^- High pH**				3.6	2.5

(*) NOE detectable but not quantifiable, (**) NOE not detectable, (***) from 1D-t-ROESY

(#) The STEP-t-ROESY was adapted/written in house from 1D-t-ROESY and STEP-NOESY standard Bruker pulse sequences. Thanks to Tammo Diercks.

Table S05

Estimated inter-residue distances using Isolated Spin Pair Approximation (ISPA).

4.9 Estimation of the inter-residue NOEs and ROEs using full matrix relaxation approach (MSpin software)

4.9.1 Estimation for the closed form of 5mer-III

tc+25%	NOESY	dist (Å)	mixing (ms)	dist (Å)	mixing (ms)	dist (Å)	mixing (ms)	dist (Å)	mixing (ms)	dist (Å)	mixing (ms)	dist (Å)
1015.90	100 ms (Intensity)		200 ms (Intensity)		300 ms (Intensity)		400 ms (Intensity)		600 ms (Intensity)		800 ms (Intensity)	
B5-C2	2.88	2.31	5.71	2.31	8.57	2.30	11.46	2.31	17.10	2.30	22.63	2.31
B5-B3	1.77	2.50	3.51	2.50	5.23	2.50	7.14	2.50	10.44	2.50	13.98	2.50
E1-D2	5.47	2.72	10.80	2.72	16.27	2.72	21.15	2.72	32.56	2.72	41.78	2.72
D2-D4	7.19	2.60	14.04	2.60	21.51	2.60	27.44	2.60	42.43	2.60	54.76	2.60
tc	NOESY											
812.72												
B5-C2	2.23	2.30	4.45	2.31	6.67	2.30	8.88	2.29	13.28	2.29	17.62	2.30
B5-B3	1.35	2.50	2.78	2.50	4.01	2.50	5.23	2.50	7.89	2.50	10.67	2.50
E1-D2	4.23	2.72	8.44	2.73	12.63	2.71	16.77	2.73	24.86	2.73	32.61	2.71
D2-D4	5.56	2.60	11.23	2.60	16.36	2.60	22.65	2.60	32.96	2.60	41.45	2.60
tc-25%	NOESY											
609.54												
B5-C2	1.55	2.30	3.09	2.29	4.64	2.31	6.18	2.30	9.25	2.29	12.31	2.30
B5-B3	0.94	2.50	1.83	2.50	2.87	2.50	3.74	2.50	5.52	2.50	7.56	2.50
E1-D2	2.94	2.72	5.87	2.73	8.80	2.71	11.71	2.72	17.42	2.71	23.10	2.73
D2-D4	3.87	2.60	7.95	2.60	11.34	2.60	15.40	2.60	22.53	2.60	30.67	2.60

Table S06 Estimation of the inter-residue NOEs at different mixing and correlation times for the closed form of **5mer-III**.

tc+25%	ROESY	dist (Å)	mixing (ms)	dist (Å)	mixing (ms)	dist (Å)	mixing (ms)	dist (Å)	mixing (ms)	dist (Å)
1015.90	50 ms (Intensity)		100 ms (Intensity)		150 ms (Intensity)		200 ms (Intensity)		300 ms (Intensity)	
B5-C2	-3.19	2.30	-6.43	2.31	-9.63	2.31	-12.81	2.31	-19.01	2.31
B5-B3	-1.95	2.50	-4.01	2.50	-6.03	2.50	-7.90	2.50	-11.89	2.50
E1-D2	-6.21	2.72	-12.34	2.72	-18.30	2.73	-24.02	2.72	-35.18	2.73
D2-D4	-8.10	2.60	-16.24	2.60	-24.45	2.60	-31.67	2.60	-46.78	2.60
tc	ROESY									
812.72										
B5-C2	-2.65	2.30	-5.29	2.31	-7.93	2.31	-10.56	2.31	-15.76	2.30
B5-B3	-1.60	2.50	-3.32	2.50	-4.89	2.50	-6.65	2.50	-9.55	2.50
E1-D2	-5.03	2.72	-10.03	2.72	-14.99	2.73	-19.87	2.73	-29.32	2.72
D2-D4	-6.60	2.60	-13.11	2.60	-20.23	2.60	-26.43	2.60	-38.65	2.60
tc-25%	ROESY									
609.54										
B5-C2	-2.10	2.30	-4.19	2.32	-6.28	2.30	-8.36	2.31	-12.50	2.31
B5-B3	-1.27	2.50	-2.65	2.50	-3.78	2.50	-5.22	2.50	-7.76	2.50
E1-D2	-3.98	2.72	-7.95	2.73	-11.90	2.71	-15.80	2.72	-23.46	2.73
D2-D4	-5.26	2.60	-10.66	2.60	-15.34	2.60	-20.77	2.60	-31.32	2.60

Table S07 Estimation of the inter-residue ROEs at different mixing and correlation times for the closed form of **5mer-III**.

For the 1 ns correlation time	100 ms	200 ms	300 ms	400 ms	600 ms	800 ms
Ratio B5-C2/B5-B3 NOESY (Intensities)	1.63	1.63	1.64	1.61	1.64	1.62
Ratio E1-D2/D2-D4 NOESY (Intensities)	0.76	0.77	0.76	0.77	0.77	0.76
dist B5-C2 NOESY (Å)	2.31	2.31	2.30	2.31	2.30	2.31
dist E1-D2 NOESY (Å)	2.72	2.71	2.72	2.71	2.72	2.72

Table S08 Ratios of the estimated inter and intra-residue NOE intensities for the closed form of **5mer-III** at 1 ns correlation time.

For the 1 ns correlation time	50 ms	100 ms	150 ms	200 ms	300 ms
Ratio B5-C2/B5-B3 ROESY (Intensities)	1.64	1.60	1.60	1.62	1.60
Ratio E1-D2/D2-D4 ROESY (Intensities)	0.77	0.76	0.75	0.76	0.75
dist B5-C2 ROESY (Å)	2.30	2.31	2.31	2.31	2.31
dist E1-D2 ROESY (Å)	2.73	2.73	2.73	2.72	2.73

Table S09 Ratios of the estimated inter and intra-residue ROE intensities for the closed form of **5mer-III** at 1ns correlation time.

4.9.2 Estimation for the 80:20 conformational equilibrium of the open form of 5mer-III

tc+25%	NOESY	dist (Å)	mixing (ms)	dist (Å)	mixing (ms)	dist (Å)	mixing (ms)	dist (Å)	mixing (ms)	dist (Å)	mixing (ms)	dist (Å)
1015.90	100 ms (Intensity)		200 ms (Intensity)		300 ms (Intensity)		400 ms (Intensity)		600 ms (Intensity)		800 ms (Intensity)	
B5-C2	2.40	2.36	4.8	2.36	7.19	2.36	9.58	2.36	14.33	2.36	19.03	2.36
B5-B3	1.70	2.50	3.41	2.50	5.11	2.50	6.82	2.50	10.23	2.50	13.63	2.50
E1-D2	0.75	2.84	1.51	2.84	2.26	2.84	3.02	2.83	4.53	2.83	6.04	2.83
D2-D4	1.27	2.60	2.54	2.60	3.80	2.60	5.07	2.60	7.60	2.60	10.13	2.60
tc	NOESY											
812.72												
B5-C2	1.86	2.36	3.71	2.36	5.56	2.36	7.41	2.36	11.11	2.36	14.78	2.36
B5-B3	1.32	2.50	2.63	2.50	3.95	2.50	5.27	2.50	7.91	2.50	10.56	2.50
E1-D2	0.58	2.84	1.17	2.84	1.75	2.84	2.33	2.83	3.50	2.83	4.67	2.83
D2-D4	0.98	2.60	1.96	2.60	2.94	2.60	3.92	2.60	5.88	2.60	7.84	2.60
tc-25%	NOESY											
609.54												
B5-C2	1.29	2.36	2.58	2.36	3.87	2.36	5.15	2.36	7.73	2.36	10.30	2.36
B5-B3	0.92	2.50	1.83	2.50	2.75	2.50	3.66	2.50	5.50	2.50	7.34	2.50
E1-D2	0.41	2.83	0.81	2.84	1.22	2.84	5.15	2.34	2.43	2.83	3.25	2.83
D2-D4	0.68	2.60	1.36	2.60	2.04	2.60	2.73	2.60	4.09	2.60	5.45	2.60

Table S10 Estimation of the inter-residue NOEs at different mixing and correlation times for the 80:20 conformational equilibrium of the open form of **5mer-III**.

tc+25%	ROESY	dist (Å)	mixing (ms)	dist (Å)	mixing (ms)	dist (Å)	mixing (ms)	dist (Å)	mixing (ms)	dist (Å)
1015.90	50 ms (Intensity)		100 ms (Intensity)		150 ms (Intensity)		200 ms (Intensity)		300 ms (Intensity)	
B5-C2	-2.68	2.36	-5.36	2.36	-8.03	2.36	-10.70	2.36	-15.98	2.36
B5-B3	-1.90	2.50	-3.81	2.50	-5.71	2.50	-7.61	2.50	-11.39	2.50
E1-D2	-0.84	2.84	-1.69	2.84	-2.53	2.84	-3.37	2.84	-5.06	2.83
D2-D4	-1.42	2.60	-2.84	2.60	-4.26	2.60	-5.67	2.60	-8.50	2.60
tc	ROESY									
812.72										
B5-C2	-2.20	2.36	-4.41	2.36	-6.60	2.36	-8.81	2.36	-8.81	2.36
B5-B3	-1.56	2.50	-3.1	2.50	-4.69	2.50	-6.26	2.50	-6.26	2.50
E1-D2	-0.69	2.84	-1.4	2.83	-2.07	2.84	-2.77	2.84	-2.77	3.03
D2-D4	-1.17	2.60	-2.33	2.60	-3.50	2.60	-4.67	2.60	-6.99	2.60
tc-25%	ROESY									
609.54										
B5-C2	-1.75	2.36	-3.49	2.36	-5.23	2.36	-6.97	2.36	-10.44	2.36
B5-B3	-1.24	2.50	-2.48	2.50	-3.72	2.50	-4.95	2.50	-7.42	2.50
E1-D2	-0.55	2.84	-1.10	2.84	-1.65	2.84	-2.20	2.84	-3.29	2.84
D2-D4	-0.92	2.60	-1.85	2.60	-2.77	2.60	-3.69	2.60	-5.54	2.60

Table S11 Estimation of the inter-residue ROEs at different mixing and correlation times for the 80:20 conformational equilibrium of the open form of **5mer-III**.

For the 1 ns correlation time	100 ms	200 ms	300 ms	400 ms	600 ms	800 ms
Ratio B5-C2/B5-B3 NOESY (Intensities)	1.41	1.41	1.41	1.40	1.40	1.40
Ratio E1-D2/D2-D4 NOESY (Intensities)	0.59	0.59	0.59	0.60	0.60	0.60
dist B5-C2 NOESY (Å)	2.36	2.36	2.36	2.36	2.36	2.36
dist E1-D2 NOESY (Å)	2.84	2.84	2.84	2.83	2.83	2.83

Table S12 Ratios of the estimated inter and intra-residue NOE intensities for the for the 80:20 conformational equilibrium of the open form of **5mer-III** at 1 ns correlation time.

For the 1 ns correlation time	50 ms	100 ms	150 ms	200 ms	300 ms
Ratio B5-C2/B5-B3 ROESY (Intensities)	1.41	1.41	1.41	1.41	1.40
Ratio E1-D2/D2-D4 ROESY (Intensities)	0.59	0.59	0.59	0.59	0.60
dist B5-C2 ROESY (Å)	2.36	2.36	2.36	2.36	2.36
dist E1-D2 ROESY (Å)	2.84	2.84	2.84	2.84	2.83

Table S13 Ratios of the estimated inter and intra-residue NOE intensities for the for the 80:20 conformational equilibrium of the open form of **5mer-III** at 1 ns correlation time.

4.10 ^{1}H and ^{13}C chemical shifts assignment for all the compounds

		Chemical shifts δ (ppm)									
Residues	Proton labels	5mer-III-zwi		5mer-III-di-SO_3^-		5mer-III-di- PO_3^{2-}		5mer-III-di-CO_2^-		5mer-III-di-NH_3^+	
		^{1}H	^{13}C	^{1}H	^{13}C	^{1}H	^{13}C	^{1}H	^{13}C	^{1}H	^{13}C
Glc A (α)	Glc A-1 (α)	5.14	91.93	5.14	91.95	5.14	91.93	5.14	91.92	5.17	92.09
	Glc A-2 (α)	3.64	72.58	3.64	72.44	3.64	72.42	3.64	72.41	3.66	72.58
	Glc A-3 (α)	3.96	74.36	3.94	75.02	3.94	75.32	3.94	74.34	3.95	75.32
	Glc A-4 (α)	3.78	72.42	3.80	72.44	3.80	72.58	3.79	72.25	3.80	72.58
	Glc A-5 (α)	3.91	70.81	3.91	70.83	3.91	70.81	3.90	70.80	3.92	70.81
	Glc A-6 (α)	3.89	59.36	3.92	60.35	3.92	59.52	3.92	59.51	3.92	59.52
	Glc A-6' (α)	3.82	59.36	3.83	59.38	3.83	60.17	3.81	59.51	3.84	60.65
Glc A (β)	Glc A-1 (β)	4.61	95.64	4.61	95.66	4.61	95.80	4.61	95.63	4.63	95.64
	Glc A-2 (β)	3.35	75.32	3.35	73.41	3.35	75.16	3.35	75.31	3.36	75.32
	Glc A-3 (β)	3.78	72.42	3.81	72.44	3.82	72.58	3.80	72.25	3.82	72.58
	Glc A-4 (β)	3.80	76.61	3.77	77.44	3.77	77.58	3.79	76.76	3.79	77.58
	Glc A-5 (β)	3.53	75.32	3.54	74.86	3.54	75.16	3.54	75.31	3.55	75.16
	Glc A-6 (β)	3.89	59.36	3.91	60.35	3.91	59.52	3.90	59.51	3.93	59.52
	Glc A-6' (β)	3.75	60.65	3.77	59.67	3.79	60.49	3.75	59.58	3.79	59.52
Rha B	Rha B-1	5.26, 5.20	99.83, 99.83	5.24, 5.19	100.01, 99.85	5.24, 5.19	100.15, 99.83	5.26, 5.20	99.82, 99.66	5.26, 5.20	100.31, 100.15
	Rha B-2	3.99	69.84	4.00	70.02	4.00	70.00	4.00	69.83	4.02	70.00
	Rha B-3	4.11	70.00	4.06	70.02	4.06	70.16	4.12	69.99	4.08	70.16
	Rha B-4	3.63	81.61	3.64	81.15	3.64	81.13	3.63	81.60	3.64	81.45
	Rha B-5	4.43	66.78	4.41	66.96	4.39	67.10	4.40	66.76	4.44	66.94
	Rha B-6	1.27	16.47	1.29	16.65	1.28	16.63	1.27	16.46	1.28	16.63
Glc C	Glc C-1	4.47	100.96	4.47	100.98	4.47	100.96	4.47	100.79	4.49	101.12
	Glc C-2	3.28	73.39	3.31	73.41	3.31	73.39	3.29	73.21	3.30	73.71
	Glc C-3	3.57	74.19	3.60	74.21	3.60	74.19	3.57	74.02	3.62	74.19
	Glc C-4	3.39	80.16	3.46	80.02	3.46	79.84	3.42	80.15	3.46	80.00
	Glc C-5	3.53	75.32	3.53	74.86	3.54	75.16	3.53	75.31	3.55	75.16
	Glc C-6	4.04	60.97	4.03	60.99	4.03	60.81	4.06	60.96	4.04	60.97
	Glc C-6'	3.77	60.65	3.78	60.99	3.78	60.49	3.78	60.96	3.80	60.65

Glc D	Glc D-1	4.67	103.54	4.68	103.24	4.68	103.38	4.67	103.69	4.69	103.54
	Glc D-2	3.30	73.39	3.36	73.41	3.33	73.39	3.32	73.21	3.31	73.71
	Glc D-3	3.38	85.96	3.70	74.21	3.66	75.65	3.36	85.63	3.58	73.81
	Glc D-4	3.45	69.36	4.09	76.47	3.82	72.58	3.44	69.51	3.00	52.43
	Glc D-5	3.40	75.64	3.54	74.86	3.45	75.16	3.40	75.63	3.57	73.81
	Glc D-6	3.86	60.49	3.90	60.35	3.84	60.17	3.86	60.32	3.88	60.65
	Glc D-6’	3.67	60.33	3.72	60.35	3.76	60.49	3.67	60.32	3.77	60.65
Glc E	Glc E-1	4.44	102.73	4.47	102.43	4.46	102.41	4.46	102.56	4.46	102.73
	Glc E-2	3.29	73.39	3.34	73.41	3.32	73.39	3.34	73.21	3.32	73.71
	Glc E-3	3.63	72.42	3.71	74.21	3.69	75.00	3.34	85.63	3.51	73.55
	Glc E-4	3.05	51.94	4.11	76.47	3.84	72.58	3.49	68.54	2.97	52.27
	Glc E-5	3.57	74.19	3.66	74.21	3.59	74.19	3.59	75.31	3.62	74.19
	Glc E-6	3.85	60.49	3.92	60.35	3.82	60.17	3.83	59.51	3.88	60.65
	Glc E-6’	3.75	60.65	3.71	60.35	3.76	60.49	3.69	60.32	3.75	60.65
Glc D/E	CH_2 next to COOH	4.24, 4.16	71.45					4.17	71.28		

Table S14 ^{1}H and ^{13}C chemical shift values assigned at neutral pH from 2D HSQC and 1D TOCSY for all synthesized compounds in this work.

5 References

(1) Eller, S.; Collot, M.; Yin, J.; Hahm, H. S.; Seeberger, P. H. Automated solid-phase synthesis of chondroitin sulfate glycosaminoglycans. *Angew. Chem. Int. Ed.* **2013**, *52* (22), 5858-5861. https://doi.org/10.1002/anie.201210132.

(2) Suzuki, T.; Yanaka, S.; Watanabe, T.; Yan, G.; Satoh, T.; Yagi, H.; Yamaguchi, T.; Kato, K. Remodeling of the Oligosaccharide Conformational Space in the Prebound State To Improve Lectin-Binding Affinity. *Biochemistry* **2020**, *59* (34), 3180-3185. https://doi.org/10.1021/acs.biochem.9b00594.

(3) Fittolani, G.; Tyrikos-Ergas, T.; Poveda, A.; Yu, Y.; Yadav, N.; Seeberger, P. H.; Jiménez-Barbero, J.; Delbianco, M. Synthesis of a glycan hairpin. *Nat. Chem.* **2023**, *15* (10), 1461-1469. https://doi.org/10.1038/s41557-023-01255-5.

(4) Zhu, Y.; Tyrikos-Ergas, T.; Schiefelbein, K.; Grafmüller, A.; Seeberger, P. H.; Delbianco, M. Automated access to well-defined ionic oligosaccharides. *Org. Biomol. Chem.* **2020**, *18* (7), 1349-1353. https://doi.org/10.1039/D0OB00137F.

(5) Le Mai Hoang, K.; Pardo-Vargas, A.; Zhu, Y.; Yu, Y.; Loria, M.; Delbianco, M.; Seeberger, P. H. Traceless Photolabile Linker Expedites the Chemical Synthesis of Complex Oligosaccharides by Automated Glycan Assembly. *J. Am. Chem. Soc.* **2019**, *141* (22), 9079-9086. https://doi.org/10.1021/jacs.9b03769.

(6) Hofmann, J.; Hahm, H. S.; Seeberger, P. H.; Pagel, K. Identification of carbohydrate anomers using ion mobility-mass spectrometry. *Nature* **2015**, *526* (7572), 241-244. https://doi.org/10.1038/nature15388.

(7) Gude, M.; Ryf, J.; White, P. D. An accurate method for the quantitation of Fmoc-derivatized solid phase supports. *Lett. Pept. Sci.* **2002**, *9* (4), 203-206. https://doi.org/10.1023/A:1024148619149.

(8) Tyrikos-Ergas, T.; Sletten, E. T.; Huang, J.-Y.; Seeberger, P. H.; Delbianco, M. On resin synthesis of sulfated oligosaccharides. *Chem. Sci.* **2022**, *13* (7), 2115-2120. https://doi.org/10.1039/D1SC06063E.

(9) Sletten, E. T.; Fittolani, G.; Hribernik, N.; Dal Colle, M. C. S.; Seeberger, P. H.; Delbianco, M. Phosphates as Assisting Groups in Glycan Synthesis. *ACS Cent. Sci.* **2024**, *10* (1), 138-142. https://doi.org/10.1021/acscentsci.3c00896.

(10) Hurevich, M.; Kandasamy, J.; Ponnappa, B. M.; Collot, M.; Kopetzki, D.; McQuade, D. T.; Seeberger, P. H. Continuous photochemical cleavage of linkers for solid-phase synthesis. *Org. Lett.* **2014**, *16* (6), 1794-1797. https://doi.org/10.1021/ol500530q.

(11) Yu, Y.; Tyrikos-Ergas, T.; Zhu, Y.; Fittolani, G.; Bordoni, V.; Singhal, A.; Fair, R. J.; Grafmüller, A.; Seeberger, P. H.; Delbianco, M. Systematic Hydrogen-Bond Manipulations To Establish Polysaccharide Structure–Property Correlations. *Angew. Chem. Int. Ed.* **2019**, *58* (37), 13127-13132. https://doi.org/10.1002/anie.201906577.

(12) Yadav, N.; Djalali, S.; Poveda, A.; Ricardo, M. G.; Seeberger, P. H.; Jiménez-Barbero, J.; Delbianco, M. Dissecting the Conformational Stability of a Glycan Hairpin. *J. Am. Chem. Soc.* **2024**, *146* (9), 6369-6376. https://doi.org/10.1021/jacs.4c00423.

(13) (a)Aeschbacher, T.; Zierke, M.; Smieško, M.; Collot, M.; Mallet, J. M.; Ernst, B.; Allain, F. H.; Schubert, M. A Secondary Structural Element in a Wide Range of Fucosylated Glycoepitopes. *Chem. Eur. J.* **2017**, *23* (48), 11598-11610. https://doi.org/10.1002/chem.201701866. (b)Zhang, Y.; Gómez-Redondo, M.; Jiménez-Osés, G.; Arda, A.; Overkleeft, H. S.; van der Marel, G. A.; Jiménez-Barbero, J.; Codée, J. D. C. Synthesis and Structural Analysis of Aspergillus fumigatus Galactosaminogalactans Featuring α-Galactose, α-Galactosamine and α-N-Acetyl Galactosamine Linkages. *Angew. Chem. Int. Ed.* **2020**, *59* (31), 12746-12750. https://doi.org/10.1002/anie.202003951.

(14) Neuhaus, D.; Williamson, M. P. *The nuclear Overhauser effect in structural and conformational analysis*; New York (N.Y.) : Wiley, 2000.
